# The *Drosophila melanogaster* Neprilysin Nepl15 is involved in lipid and carbohydrate storage

**DOI:** 10.1038/s41598-021-81165-z

**Published:** 2021-01-22

**Authors:** Surya Banerjee, Christine Woods, Micheal Burnett, Scarlet J. Park, William W. Ja, Jennifer Curtiss

**Affiliations:** 1 New Mexico State University, Las Cruces, NM USA; 2 The Scripps Research Institute Florida, Jupiter, FL USA

**Keywords:** Homeostasis, Endocrine system and metabolic diseases

## Abstract

The prototypical M13 peptidase, human Neprilysin, functions as a transmembrane “ectoenzyme” that cleaves neuropeptides that regulate e.g. glucose metabolism, and has been linked to type 2 diabetes. The M13 family has undergone a remarkable, and conserved, expansion in the *Drosophila* genus. Here, we describe the function of *Drosophila melanogaster* Neprilysin-like 15 (Nepl15). Nepl15 is likely to be a secreted protein, rather than a transmembrane protein. Nepl15 has changes in critical catalytic residues that are conserved across the Drosophila genus and likely renders the Nepl15 protein catalytically inactive. Nevertheless, a knockout of the *Nepl15* gene reveals a reduction in triglyceride and glycogen storage, with the effects likely occurring during the larval feeding period. Conversely, flies overexpressing *Nepl15* store more triglycerides and glycogen. Protein modeling suggests that Nepl15 is able to bind and sequester peptide targets of catalytically active *Drosophila* M13 family members, peptides that are conserved in humans and *Drosophila*, potentially providing a novel mechanism for regulating the activity of neuropeptides in the context of lipid and carbohydrate homeostasis.

## Introduction

Neprilysin is the founding member of the M13 family of zinc metalloendopeptidases that typically function as “ecto-enzymes”. M13 family members typically contain a transmembrane domain and a C-terminal extracellular catalytic domain that cleaves secreted peptides^1–3^. Mammalian Neprilysin has a number of potential peptide targets involved in regulating neuronal function, appetite, metabolism, energy homeostasis and inflammation (e.g. tachykinins such as Substance P, galanin, cholecystokinin and neuropeptide Y)^4–8^. Recently, mammalian Neprilysin has been studied as a potential therapeutic target for treating type 2 diabetes^9^.

*Drosophila melanogaster* (*D. melanogaster*) behave remarkably similarly to mammals in terms of nutrient metabolism and energy homeostasis and have become a powerful model organism to study these processes^10–12^. Like mammals, *D. melanogaster* have organs for digestion and nutrient absorption (the midgut). They have a circulatory system (the hemolymph) that conveys lipids and other nutrients from one organ to another. The fat body stores carbohydates as glycogen and lipids as triacylglycerides (TAG), and thus combines functions of mammalian liver and adipose tissue. *Drosophila* use conserved pathways to control metabolism and energy homeostasis. For example, Insulin Like Peptides (ILPs) are released from Insulin Producing Cells (IPCs) in the brain, and signal via a conserved Insulin Receptor pathway that promotes nutrient uptake by cells. Adipokinetic Hormone (AKH), a *Drosophila* glucagon counterpart, is released from Corpora Cadiaca (CC) cells and signals via a conserved G Coupled Protein Receptor (GCPR) pathway that promotes nutrient mobilization. In addition, a number of peptide targets of mammalian Neprilysins that are known to be involved in feeding behavior, appetite regulation, metabolism and energy homeostasis, have *D. melanogaster* counterparts that are linked to similar processes^10,13–15^.

Here we show that the *D. melanogaster* M13 family member, Nepl15, is likely to be secreted and to be catalytically inactive. Nevertheless, we demonstrate that Nepl15 has a role in lipid and carbohydrate storage. *Nepl15* is strongly expressed in the fat body, and is differentially expressed between males and females. Knock-out mutations in *Nepl15* result in reduced levels of triglycerides and glycogen in adult males but not in females. Further, *Nepl15* mutants show an increase in larval developmental time that is consistent with a reduced ability to store nutrients needed to complete metamorphosis. Conversely, over-expression of Nepl15 in certain tissues results in increases in triglyceride and glycogen storage in males. Our results are consistent with a role for Nepl15 in regulating neuropeptide cleavage in the context of nutrient metabolism.

## Results

### Nepl15 is likely a secreted, catalytically dead member of the peptidase M13 family

**Figure 1:**
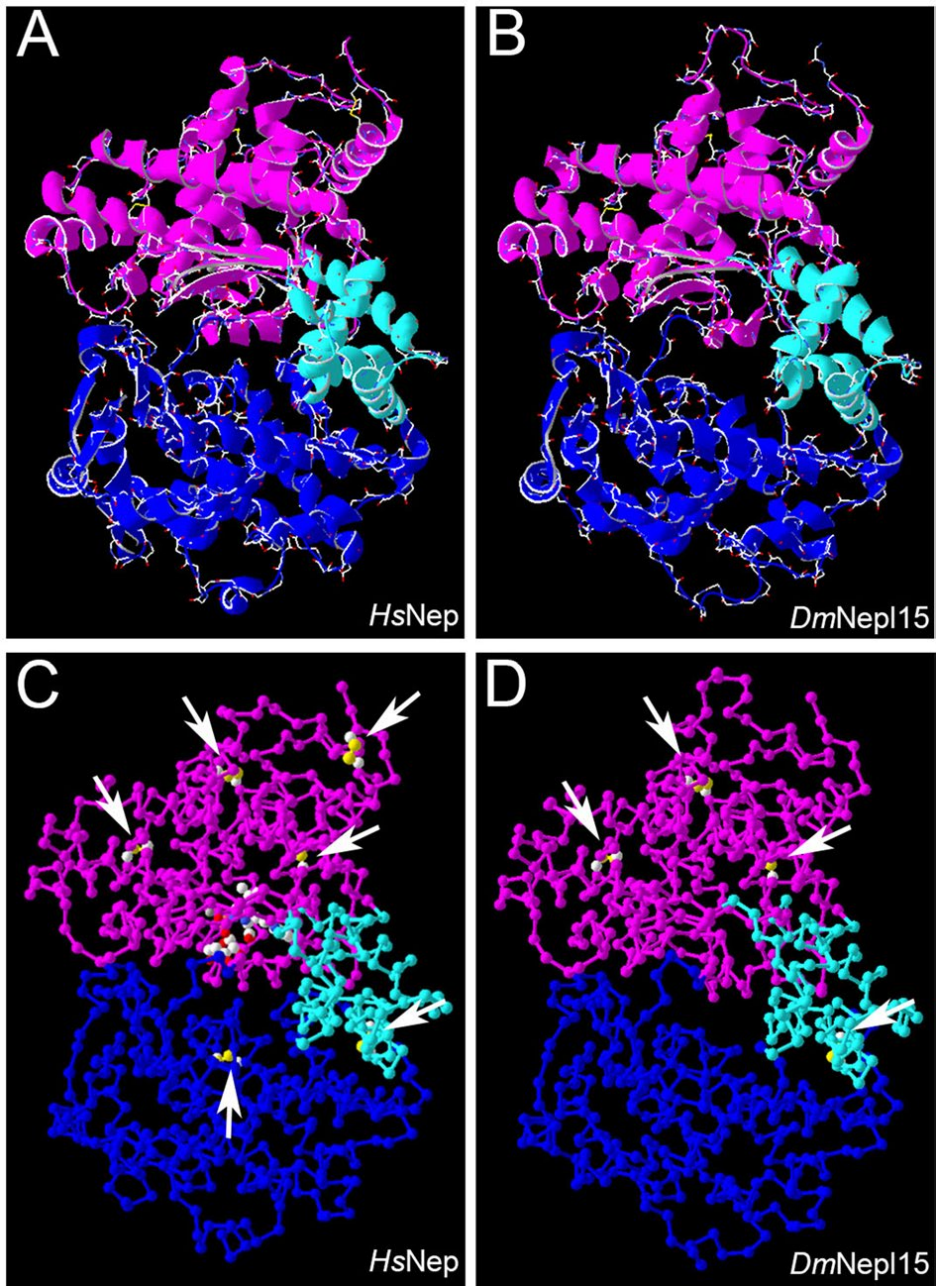
The domain structure of *Dm*Nepl15 is similar to that of other M13 family members. Ribbon diagrams (A,B) and backbone diagrams (C,D) of a model of a crystal structure of *Hs*Neprilysin (*Hs*Nep) in complex with the inhibitor phosphoramidon (1dmt)^16^ (A,C), and a homology model of *Dm*Nepl15 based on the 1dmt model (B,D). Magenta, blueberry and turquoise colors denote domains 1, 2 and 3, respectively. Arrows in C and D indicate disulfide bonds. These drawings were made using the DeepView—Swiss-PdbViewer (version 4.1.1)^114^ (http://www.expasy.org/spdbv/).

*Drosophila melanogaster* Nepl15 (formerly CG4721) is a member of a *Drosophila*-specific clade of the Peptidase M13 family, most of which are membrane-bound Zn(II) metalloendopeptidases that function as ectoenzymes^3^. Alignments of *Drosophila* Nepl15 orthologs with other M13 family members (Figure S1), as well as homology modeling of *D. melanogaster* Nepl15 (*Dm*Nepl15) (Figure S2,3), suggest that the Nepl15 domain structure is similar to that of both *Homo sapiens* Neprilysin (*Hs*Neprilysin) and *Hs*ECE-I. Domains 1 and 2 surround a central cavity and are linked by domain 3 (Fig. 1). Accordingly, most of the conserved Cys residues involved in disulfide bonds in *Hs*Neprilysin and in *Hs*ECE-1 are also found in the *Drosophila* Nepl15 orthologs, suggesting that they contribute to promoting a similar tertiary structure^3,16–21^.

**Figure 2:**
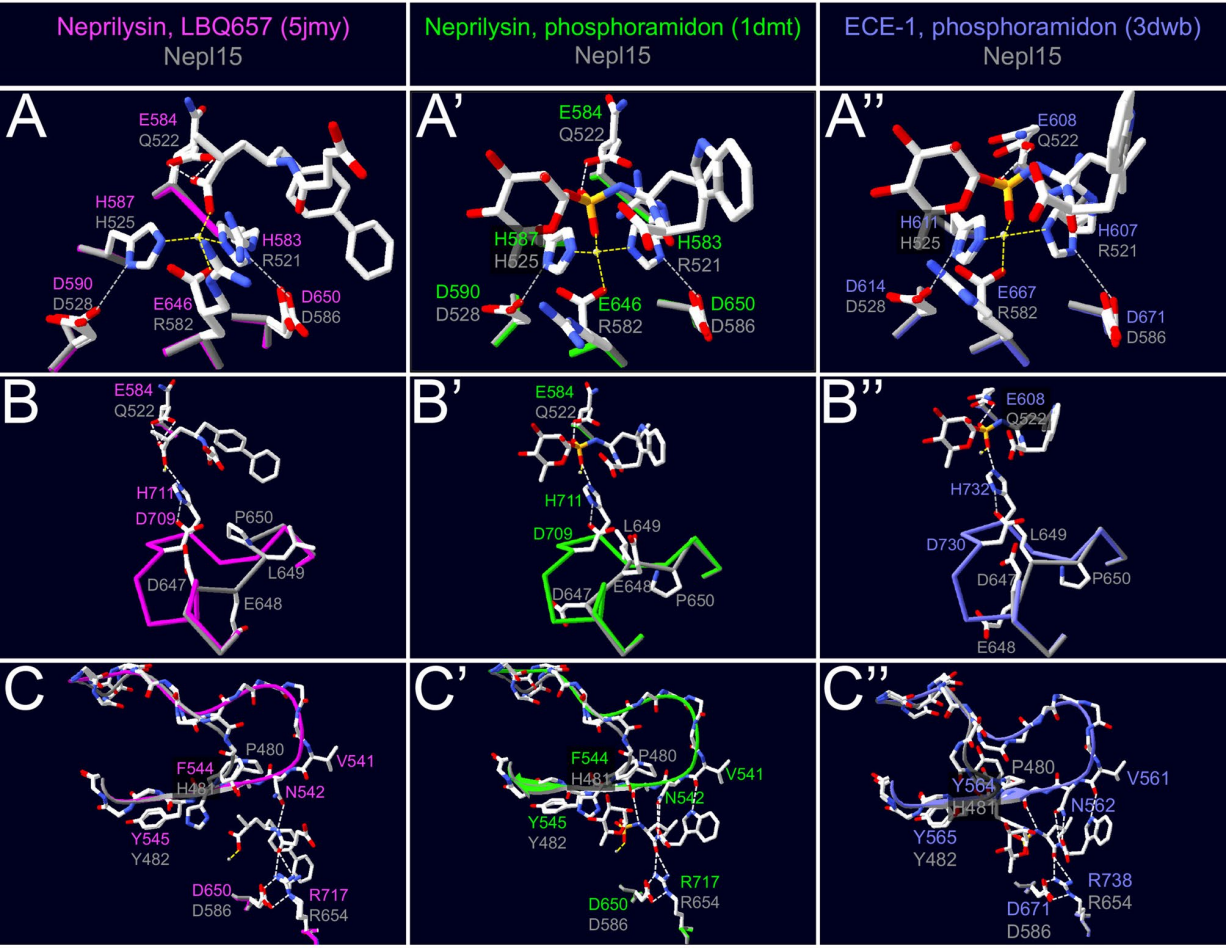
*Dm*Nepl15 lacks critical catalytic residues. Homology models of *Dm*Nepl15 (gray) based on models of crystal structures of *Hs*Neprilysin in complex with (left, magenta) LBQ657, the active metabolite of sacubitril (5jmy)^21^; and (center, green) with the inhibitor phosphoramidon (1dmt)^16^; (right, blue) homology model of *Dm*Nepl15 based on a model of a crystal structure of *Hs*ECE-1 in complex with the inhibitor phosphoramidon (3dwb)^17^. Yellow, white and gray dotted lines denote interactions between Zn(II) and its coordinators, hydrogen bonds, and other interactions described in the text, respectively. **(A–A”)** Two of the three residues involved in Zn(II) coordination in *Hs*Neprilysin (His583, His587 and Glu646) and in *Hs*ECE-1 (His607, His611, and Glu667) are replaced by Arg in *Dm*Nepl15. Asp residues involved in Asp-His-Zinc triads in *Hs*Neprilysin (Asp590 and Asp650) and in *Hs*ECE-1 (Asp614 and Asp671)^16–18,27,115^ are present at these positions in *Dm*Nepl15. The base that deprotonates water in *Hs*Neprilysin (Glu584) and in *Hs*ECE-1 (Glu608) is replaced by Gln in *Dm*Nepl15. **(B–B”)** In *Hs*Neprilysin, His711 is thought to help stabilize the transition state during catalysis; Asp709 interacts with His711 and is crucial for catalysis^16,21,28–31^. The corresponding residues are Asp730 and His732 in *Hs*ECE-1^17,27^. There appears to be no counterpart to *Hs*Neprilysin His711/732 in the *Drosophila* Nepl15 orthologs. **(C–C”)** In the *Hs*Neprilysin and *Hs*ECE-1 crystal structures the N-A-Ar–Ar motif includes the Asn542/Asn562 side chains and backbone CO groups that hydrogen bond with backbone elements of the LBQ657 and phosphoramidon inhibitors. There does not appear to be a *Dm*Nepl15 counterpart to Asn542/Asn562. Ala543/Ala563 in *Hs*Neprilysin appears to be replaced with Pro in the *Drosophila* Nepl15 orthologs, and Phe544/Tyr565 is replaced by His. *Hs*Neprilysin Arg717 and its *Hs*ECE-1 Arg738 counterpart hydrogen bond with backbone CO groups on the LBQ657 and phosphoramidon inhibitors, participate in salt bridges with Asp650/Asp671 and are important for catalytic activity^16,17,21,24,46,47^. *Drosophila* Nepl15 counterparts of these residues are Arg654 and Asp586. These drawings were made using the DeepView—Swiss-PdbViewer (version 4.1.1)^114^ (http://www.expasy.org/spdbv/).

Although the overall tertiary structure of *Drosophila* Nepl15 orthologs is likely to be similar to other M13 family members, Nepl15 appears to differ in several important respects. First, most of the *Drosophila* Nepl15 orthologs are likely to be secreted rather than transmembrane proteins (Figures S4-S6). Second, Nepl15s are likely to be catalytically inactive. For instance, two of the three His and Glu residues involved in Zn(II) coordination in M13 family members are changed to Arg in the *Drosophila* Nepl15 orthologs^16,21–25^. In addition, the Glu residue that helps deprotonate the hydrolytic water in other M13 family members is replaced by Gln^17,21,24,26,27^. Finally, a His residue thought to help stabilize the transition state during catalysis^16,17,21,27–31^ does not appear to have a counterpart in the *Drosophila* Nepl15 orthologs (Fig. 2A,A’,A”,B,B’,B”; Figure S1).

### Nepl15 may still be able to bind to peptide substrates

Substrate binding in peptidases is based partly on interactions between the amino acids in the active site of the enzyme with the backbone of the peptide substrate, and partly on the amino acid sequence of the peptide substrate. Based on a nomenclature system in general use for peptidases^32^, the amino acids in the peptide substrate are labeled e.g. P1, P1′ and P2′, with the C-terminus on the prime side, and cleavage occurs between the P1 and P1′ amino acids; the corresponding subsites are labeled S1, S1′ and S2’.

Most M13 family members share an N–A–Ar–Ar motif (“Ar” for aromatic amino acid)^16,17,28,33–37^ in which the Asn and Ala residues in the motif are involved in binding to the peptide backbone and the two aromatic amino acids form the S1 and S2 subsites. There does not appear to be a *Dm*Nepl15 Asn counterpart, and Ala appears to be replaced with Pro in the *Drosophila* Nepl15 orthologs. Moreover, the aromatic amino acid in the motif that lines the S1 subsite is replaced by a His (Fig. 2C,C’,C”; Figure S1), though the S1 subsite appears to be less important for specificity in M13 family members compared to other subsites^3,17,38–45^.

However, other aspects of the *Drosophila* Nepl15 sequences and the *Dm*Nepl15 homology model structure suggest that it could interact with substrates, albeit perhaps through a different mechanism than typical M13 family members. For instance, an Arg residue that binds backbone CO groups and is important for catalytic activity^16,17,21,24,46,47^ is present in the *Drosophila* Nepl15s (Fig. 2C; Figure S1).

**Figure 3:**
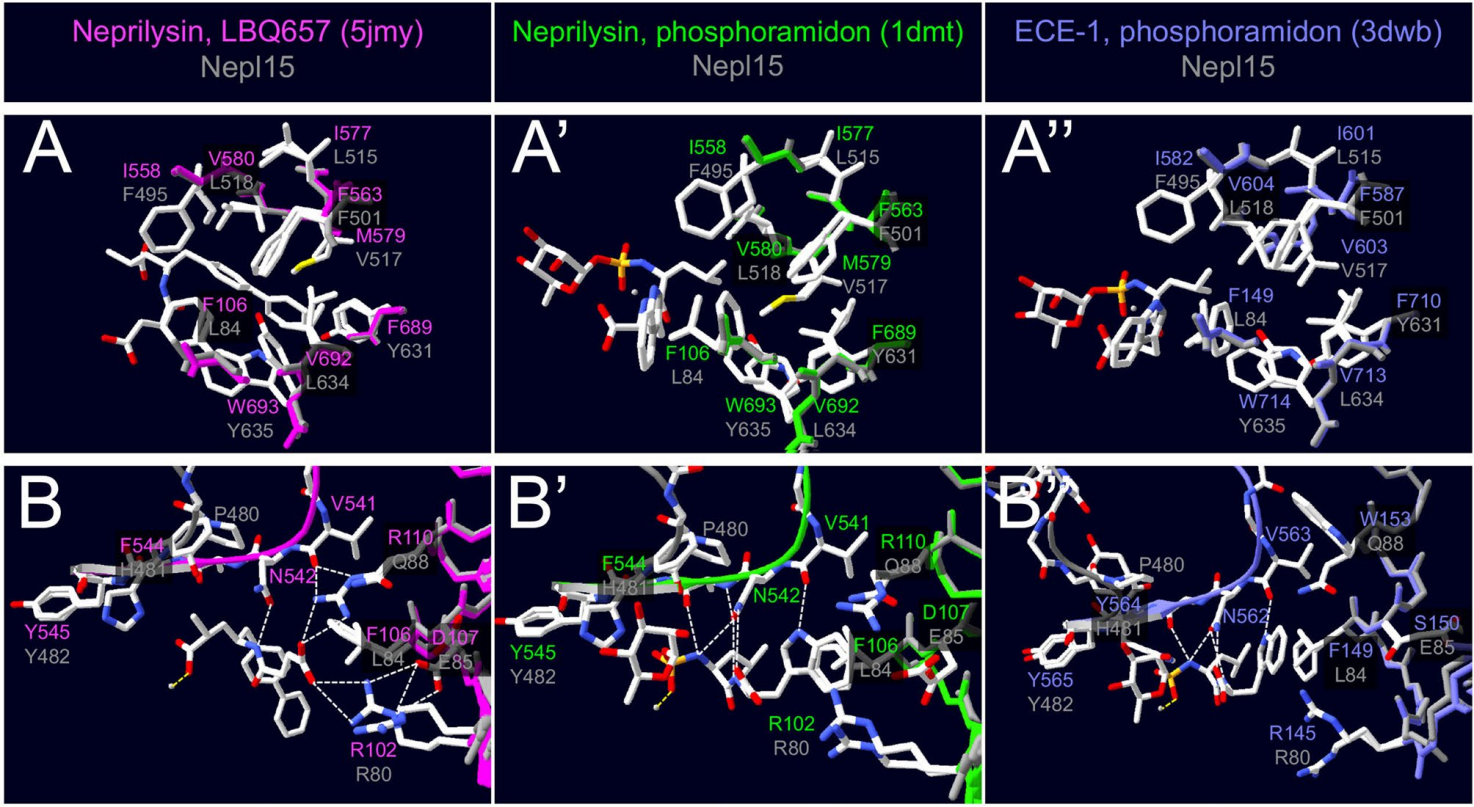
Nepl15 may be able to bind peptide substrates. Homology models of *Dm*Nepl15 (gray) based on models of crystal structures of *Hs*Neprilysin in complex with (left, magenta) LBQ657, the active metabolite of sacubitril (5jmy)^21^; and (center, green) with the inhibitor phosphoramidon (1dmt)^16^; (right, blue) homology model of *Dm*Nepl15 based on a model of a crystal structure of *Hs*ECE-1 in complex with the inhibitor phosphoramidon (3dwb)^17^. Yellow, white and gray dotted lines denote interactions between Zn(II) and its coordinators, hydrogen bonds, and other interactions described in the text, respectively **(A–A”)** Hydrophobic residues line the S1′ subsite in DmNepl15 as they do in *Hs*Neprilysin and in *Hs*ECE-1. **(B–B”)** Interactions between the N–A–Ar–Ar motif, the S2′ subsite and the inhibitors reveal flexibility in binding to peptide substrates, as well as differences between the S2′ subsites of *Hs*Neprilysin, *Hs*ECE-1 and *Dm*Nepl15. In particular, *Hs*Neprilysin Val541, Arg110 and Arg102 are involved in positioning the terminal -COOH group that enables the dipeptidylcarboxypeptidase actvitiy of which *Hs*Neprilysin is capable. Whereas Val563 appears to be the HsECE-1 counterpart to *Hs*Neprilysin Val541, there is no DmNepl15 counterpart to this residue. An Arg is present in the *Hs*ECE-1 and *Dm*Nepl15 counterparts of *Hs*Neprilysin Arg102. However, the *Hs*Neprilysin Arg110 counterparts are Trp153 in *Hs*ECE-1 and Gln88 in *Dm*Nepl15. These drawings were made using the DeepView—Swiss-PdbViewer (version 4.1.1)^114^ (http://www.expasy.org/spdbv/).

The hydrophobic residues of the S1′ subsite interact preferentially with bulky hydrophobic residues at the P1′ site are generally the most important drivers of specificity in M13 family members^1,3,16,17,48^. The corresponding S1′ site residues are also hydrophobic in the *Drosophila* Nepl15s (Fig. 3A; Figure S1).

The S2′ subsites in M13 family members vary considerably in specificity and appear to be somewhat flexible^3^. For instance, two Arg residues (Arg 102 and Arg 110) present in the *Hs*Neprilysin S2′ subsite are thought to enable this enzyme to function as a dipeptidylcarboxypeptidase in addition to its endopeptidase activity^16,21,34,47–51^. In the *Drosophila* Nepl15 orthologs Arg or Lys are found in the corresponding position to Arg102, but Arg110 is replaced with Gln (Fig. 3B, Figure S1). Taken together, these results suggest that the *Drosophila* Nepl15 orthologs are likely able to bind to peptides containing a hydrophobic amino acid in the P1′ position. The contributions of the S1 and S2′ subsites to specificity are less certain.

Based on published estimates of divergence dates among these species^52^, for the most part these similarities and differences in *Drosophila* Nepl15 orthologs compared to other M13 family members have been conserved over ~ 35 million years of evolution^53,54^. Thus, despite the probable lack of catalytic activity, the differences are likely to be important for *Drosophila* Nepl15′s unique function.

### *D. melanogaster Nepl15* transcript expression is enriched in the fat body

To further elucidate the function of the *Nepl15* gene, we first checked the *Nepl15* mRNA expression profile based on genome-wide microarray^55^ and RNAseq data^56^ (Figure S7). In both microarray data and RNAseq data the larval fat body had the highest levels of *Nepl15* expression among the different larval tissues tested. Both microarray and RNAseq data also showed that the adult fat body has among the highest levels of expression of *Nepl15* compared to other adult tissues. We also noted from the RNAseq data that *Nepl15* expression levels are more than four-fold higher in whole adult males compared to whole adult females, despite comparable levels between adult male and female in most tissues (Figure S7). The 1.6-fold difference in fat body expression levels between males and females suggests it is one of the primary contributors to the difference in *Nepl15* levels in adult males versus females.

**Figure 4:**
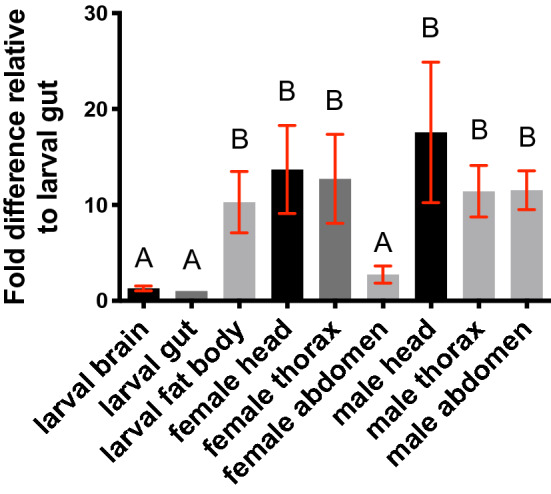
Male and female adults have different *Nepl15* transcript expression patterns. RT-qPCR data to show relative abundance of *Nepl15* transcripts in different organs of the wild type (*w*^*1118*^) third instar larvae and in different body parts of adult female and male flies. Data are displayed as fold differences compared to the larval gut. ΔCt values were analyzed using a one-way ANOVA. Fold differences in different organs and body segments were assigned to statistical groups using Tukey’s multiple comparison test. Groups sharing at least one letter are not significantly different; groups not sharing any letter are significantly different (*P* < 0.05). Error bars represent the standard error of mean (SEM). Larval tissues and adult female tissues: n = 4 (3 technical replicates each). Adult male tissues: n = 3 (3 technical replicates each).

To corroborate the *Nepl15* transcript expression data available from the genome-wide studies described above, we performed RT-qPCR to quantify the relative abundance of *Nepl15* transcripts in the third instar larval central nervous system, gut and fat body, and in the head, thorax and abdomen of *w*^*1118*^, 2–3 days old, adult female and male flies (Fig. 4). Consistent with the genome-wide data described above, *Nepl15* transcript levels in the larval fat body were significantly higher compared to levels in larval brain and gut. *Nepl15* transcript levels in the adult female head and thorax, as well as the adult male head, thorax and abdomen, were similar to each other and to levels in the larval fat body. Strikingly, however, *Nepl15* transcript levels in the abdomens of adult female flies were significantly lower than those in abdomens of adult male flies and were comparable to the much lower levels of *Nepl15* transcripts observed in the larval gut and brain.

The difference in *Nepl15* transcript levels in the adult male versus female abdomen are interesting, but are not easily compared to the genome-wide studies, which looked at individual organs. Furthermore, there is substantial variability in *Nepl15* transcript levels among biological replicates. This variability is likely to result from variability in expression rather than variability due to experimental technique, because we were careful to follow MIQE guidelines that stipulate that standard deviations between technical replicates be $\le$ 0.3, and that standard deviations between biological replicates be $\le$ 0.5 for the *RpL32* control (see “Methods”). Nevertheless, the higher *Nepl15* transcript levels in the larval and adult fat body suggest a role for *Nepl15* expression in the fat body.

### *Nepl15* knockout (*w*^*1118*^*; Nepl15*^*ko*^) flies have reduced lipids

To study the function of Nepl15, we generated knockout mutants (*w*^*1118*^*; Nepl15*^*ko*^) by ends-out homologous recombination^57^. We have confirmed the knockouts by performing genomic PCR followed by sequencing as well as by RT-qPCR (see Materials and Methods). Homozygous knockout flies are viable and fertile. Following our initial observation that *Nepl15* is highly expressed in the larval and adult fat body compared to other tissues, we assessed whether *Nepl15* knockout mutants have defects in fat body function.

**Figure 5:**
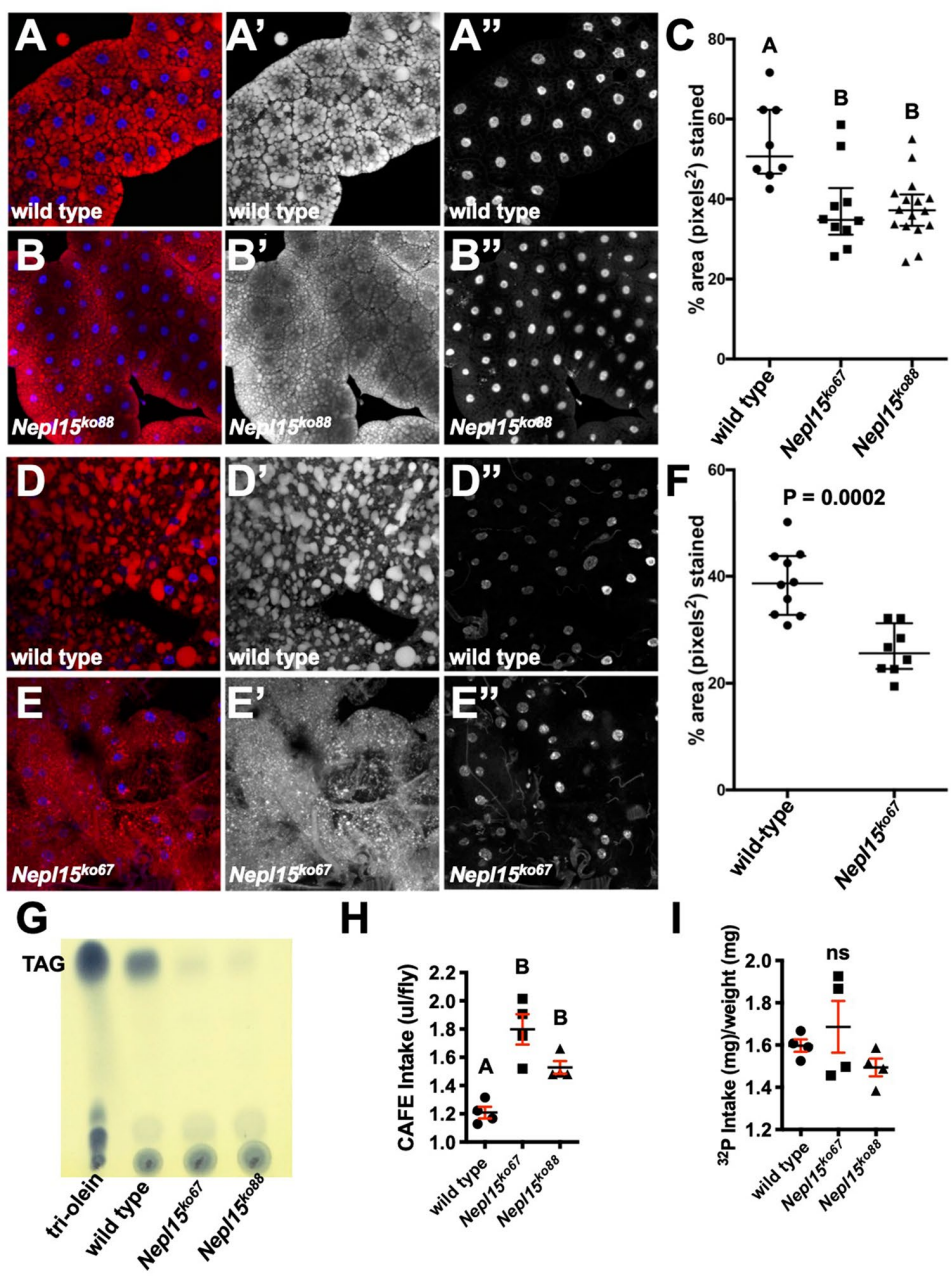
*Nepl15*^*ko*^ flies have reduced triacylglycerides. Fluorescent images of Nile Red stainings in larval fat bodies from wild-type and *Nepl15*^*ko*^ larvae **(A,B)**; nuclei are marked by Hoechst 33342. Merges in the left-hand panels, Nile Red in the middle panels, Hoechst in the right-hand panels. Quantification of Nile Red stainings in larval fat bodies **(C)**. Nile Red stainings in wild-type and *Nepl15*^*ko*^ adult fat bodies **(D,E)**; nuclei are marked by Hoechst 33342. Merges in the left-hand panels, Nile Red in the middle panels, Hoechst in the right-hand panels. Quantification of Nile Red stainings in adult fat bodies **(F)**. **(G)** Uncropped image of a thin layer chromatography plate used to separate triacylglycerides (TAG) in lysates from adult male wild-type flies and from adult males from two independent knock-out isolate strains (*Nepl15*^*ko67*^ and *Nepl15ko*^*88*^). Tri-olein was used as a standard. **(H,I)** Food intake was measured by CAFE assay (**H**; n = 4 for all genotypes), and by food labeling by radioactive tracer (**I**; n = 4 for all genotypes). In panel **(I)** radioactive tracer data from panel **(H)** was normalized to body weight. Values in **(C,H,I)** were analyzed using a one-way ANOVA and were assigned to statistical groups using Tukey’s multiple comparison test. Groups sharing at least one letter are not significantly different; groups not sharing any letter are significantly different (*P* < 0.05). Values in F were analyzed using a two-tailed student’s t test and the P value is shown. Error bars represent the standard error of mean (SEM).

One major role of the fat body is storing lipids in the form of triacylglycerides (TAG) in lipid droplets. Therefore, we stained the larval (Fig. 5A-C) and adult fat bodies (Fig. 5D-F) from wild type and *Nepl15*^*ko*^ flies with the red fluorescent dye Nile Red to observe lipid droplets. Lipid droplets in the larval and adult fat body of the *Nepl15*^*ko*^ flies appeared smaller than those in the wild type fat body and the % area of Nile Red staining was significantly less in *Nepl15*^*ko*^ larvae and adult fat bodies compared to wild type. The images shown in Fig. 5A-F are representative of the images used for the quantification, and we did not observe obvious differences between different regions of the fat body. We also stained the larval fat body with periodic acid–Schiff (PAS) (Figure S8A,B) to detect polysaccharides, including glycogen^58^. Under bright-field illumination, the stain results in accumulation of a dark pink pigment in areas with high polysaccharide concentrations. *Nepl15*^*ko*^ larval fat body showed less of this dark pigment, suggesting reduced concentrations of glycogen.

To confirm the Nile Red stainings, we performed thin layer chromatography (TLC) to determine TAG levels in lysates generated from whole adult wild type and two independent *Nepl15*^*ko*^ isolates. Levels of TAG were significantly reduced in *w*^*1118*^*; Nepl15*^*ko*^ flies compared to *w*^*1118*^ flies (Fig. 5G). Taken together, our results suggest that *Nepl15* is required for TAG and glycogen storage in the fat body during both larval and adult stages.

One possibility behind the reduced levels of stored nutrients is impaired capacity of food intake by *Nepl15*^*ko*^ mutant flies compared to wild type. To examine any changes in feeding behavior, we first monitored the amount of liquid food ingested by these adult flies using the capillary feeding (CAFE) assay^59^. Interestingly, food intake was significantly increased in the adult *Nepl15*^*ko*^ flies compared to the adult wild type flies (Fig. 5H). To substantiate this finding, we also measured food ingestion via radioisotope labeling^60^ and observed a similar outcome, though the results were not statistically significant when normalized to fly weight (Fig. 5I). These results suggest that reduced storage of nutrients in the *Nepl15*^*ko*^ flies does not result from reductions in food intake.

### Adult *Nepl15*^*ko*^ males but not females have reduced glycerolipids and glycogen

To avoid the possibility that the reductions in stored TAG and glycogen observed in the *w*^*1118*^*; Nepl15*^*ko*^ flies arose from background effects or differences in population densities, we back-crossed *w*^*1118*^; *Nepl15*^*ko*^ flies to our *w*^*1118*^; + (hereafter *w*^*1118*^) lab strain for 6 generations. In addition, we raised flies at similar densities (see Materials and Methods). Intrigued by the differences in expression of *Nepl15* in males versus females, we also began to separate males and females during experiments. Using these methods, we repeated the food ingestion via radioisotope labeling experiments, with similar results: in two trials, *w*^*1118*^*; Nepl15*^*ko*^ males and females consumed approximately the same amount of food compared to their *w*^*1118*^ counterparts (results of one trial shown in Figure S8C). We also weighed *w*^*1118*^ and *w*^*1118*^*; Nepl15*^*ko*^ adult males and females. As expected, female *D. melanogaster* of both genotypes are larger than males of both genotypes, and therefore weigh more. In multiple trials and at different ages, there were no consistent differences in weight between *w*^*1118*^ and *w*^*1118*^*; Nepl15*^*ko*^ males or between *w*^*1118*^ and *w*^*1118*^*; Nepl15*^*ko*^ females. Two representative trials conducted independently in the Curtiss and Ja labs, respectively are shown in Figure S8D,E. For the experiments in Figures S8C & E that were performed in the Ja lab, flies were raised for several generations in the Ja lab after being shipped from the Curtiss lab, prior to conducting the experiments. These data provide strong evidence that there are no differences between *w*^*1118*^ and *w*^*1118*^*; Nepl15*^*ko*^ adult in terms of feeding or ability to reach and maintain a normal body weight.

**Figure 6:**
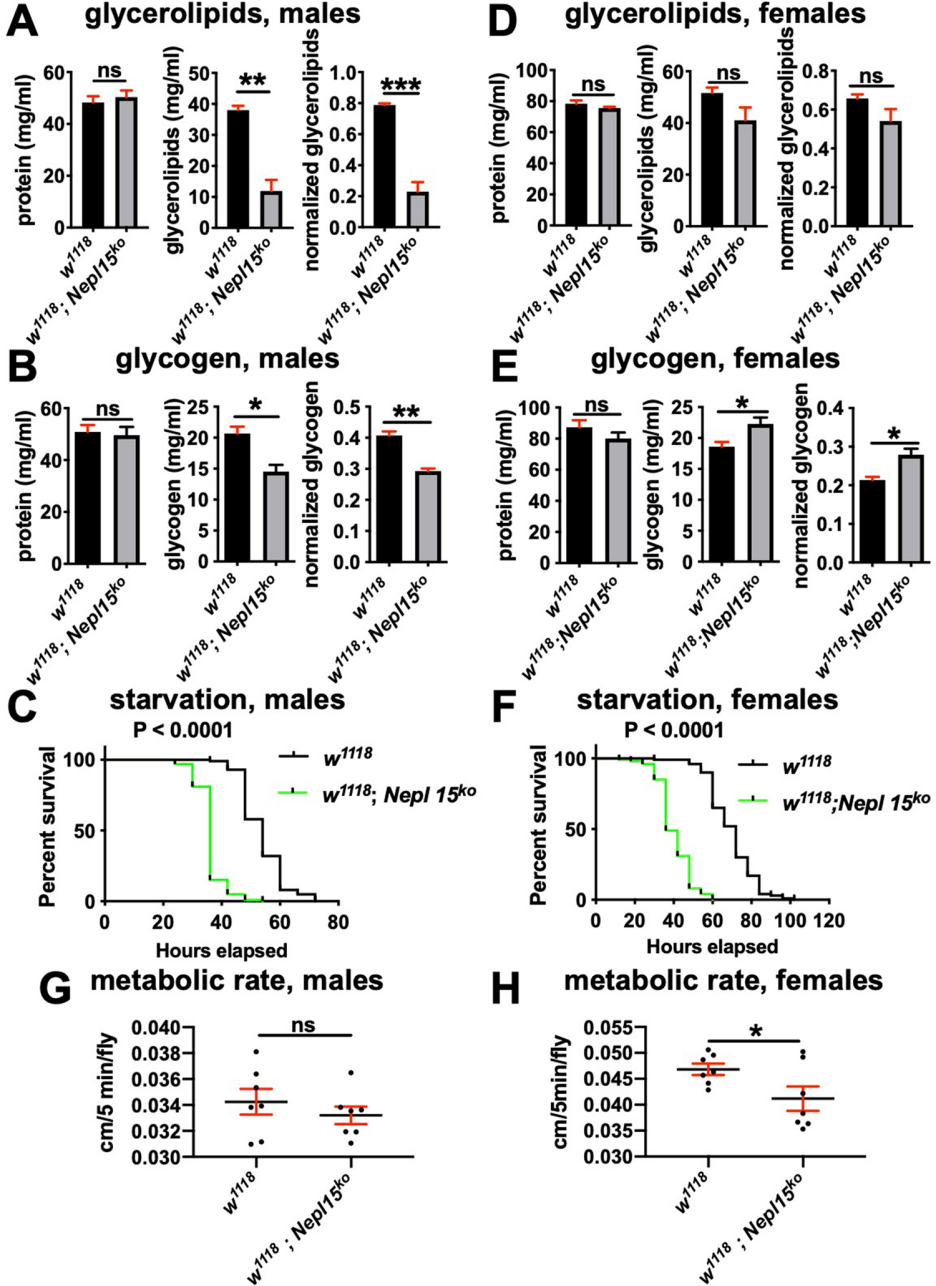
*Nepl15*^*ko*^ males have reduced glycerolipids and glycogen levels, but females have similar glycerolipid levels and slightly increased glycogen levels. **(A,D)** Protein concentration (left), glycerolipid concentration (center) and glycerolipid concentration normalized to protein concentration (right) for adult males **(A)** and females **(D)** of the indicated genotypes. **(B,E)** Protein concentration (left), glycogen concentration (center) and glycogen concentration normalized to protein concentration (right) for adult males **(B)** and females **(E)** of the indicated genotypes. Values were analyzed using an unpaired t test. *, ** and *** indicate P < 0.05, P < 0.01 and P < 0.001, respectively. ns = non-significant. Error bars represent the standard error of mean (SEM). (**A,B,D,E**: n = 3 biological replicates, with 3 technical replicates each) **(C,F)** survival curves for starvation assays for males (**C**; n = 100) and females (**F**; n = 100) of the indicated genotypes. Values were analyzed using a Log-rank test, and the *P* values are indicated on the graphs. **(G,H)** Metabolic rates for males **(G)** and females **(H)** of the indicated genotypes (n = 7 for both males and females). Values were analyzed using an unpaired t test. * indicates P < 0.05. Error bars represent the standard error of mean (SEM).

To confirm the differences between *w*^*1118*^ and *w*^*1118*^*; Nepl15*^*ko*^ flies in lipid and carbohydrate storage described above, we used more quantitative enzymatic assays coupled with colorimetric analysis for measuring lipid, carbohydrate and protein levels in age-matched, whole adult fly lysates^61^ (see also Materials and Methods). Total glycerolipid as well as glycogen concentrations were significantly reduced in whole adult *w*^*1118*^*; Nepl15*^*ko*^ male flies compared to controls (Fig. 6A,B). Accordingly, adult *w*^*1118*^*; Nepl15*^*ko*^ male flies succumbed significantly earlier to starvation than did *w*^*1118*^ control males (Fig. 6C). Only minor reductions in total glycerolipids were observed for whole adult *w*^*1118*^*; Nepl15*^*ko*^ female flies compared to controls, and they had significantly elevated glycogen levels (Fig. 6D,E). In spite of these differences, *w*^*1118*^*; Nepl15*^*ko*^ females succumb significantly earlier to starvation compared to controls (Fig. 6F), similar to males. The Ja lab independently performed similar starvation assays, with similar results (Figure S8F). Together with the staining and TLC data (Fig. 5), these results suggest that *Nepl15* is required for normal TAG and glycogen storage in both males and females.

We were intrigued by the differences between male and female *w*^*1118*^*; Nepl15*^*ko*^ flies in terms of nutrient storage, particularly of glycogen, with males having significantly less glycogen and females having significantly more. To determine whether the metabolic rates for either male and female *w*^*1118*^*; Nepl15*^*ko*^ flies differed from controls by measuring the amount of CO_2_ produced per fly over the course of a few hours. Accordingly, metabolic rate was slightly but significantly reduced in mutant females compared to control females, but did not differ in mutant males compared to control males (2 trials, similar results, representative data from 1 trial are shown in Fig. 6G,H).

We hypothesize that the differences in lipid and carbohydrate storage (elevated in *Nepl15* mutant females compared to males) as well as metabolic rate (reduced in *Nepl15* mutant females compared to males) reflects the fact that females but not males are obliged to supply resources to developing eggs. The increased levels of carbohydrates in females may be present in the eggs and not metabolically available for the females to use under starvation conditions, and the females may have to reduce their metabolic rates due to this lack of resources.

### Circulating carbohydrates are unaltered in adult *w*^*1118*^; *Nepl15*^*ko*^ flies

The nonreducing glucose dimer trehalose comprises the vast majority of circulating carbohydrates in insects including *D. melanogaster*. Trehalose is synthesized exclusively in the fat body from where it is released into the hemolymph^10,58,62–65^. Although trehalose constitutes the majority of circulating carbohydrates, hemolymph glucose levels appear to be more responsive to environmental and genetic alterations to metabolism^65^. Hemolymph glucose concentrations were slightly elevated in 2–4 day old and 8–10 day old *w*^*1118*^; *Nepl15*^*ko*^ adult male flies relative to the *w*^*1118*^ control, but the difference was not statistically significant (Figure S9A,B). Additionally, we found no significant differences in whole-body trehalose concentrations in *w*^*1118*^; *Nepl15*^*ko*^ adult male flies relative to *w*^*1118*^ flies, though whole-body glucose levels were significantly elevated (Figure S9C). Taken together, these results suggest that loss of *Nepl15* has little to no effect on circulating carbohydrates.

### Overexpressing Nepl15 results in elevated lipid and carbohydrate storage

To test whether *Nepl15* is capable of driving increased lipid and carbohydrate storage when overexpressed, we used the Gal4-UAS system to drive expression of either *UAS-GFP* or UAS-*Nepl15* (1) in all tissues using the *armadillo-Gal4* (*arm-Gal4*) driver^66^, (2) in the fat body using the *Cg-Gal4* driver^67^, and (3) in the midgut using the *mex1-Gal4* driver^68^. We used two independent *UAS-GFP* lines (one on the second chromosome and one on the third chromosome), as well as two independent *UAS-Nepl15* lines (*UAS-Nepl15*^*37*^ on the second chromosome, and *UAS-Nepl15*^*1*^ on the third). As for the loss-of-function experiments we back-crossed all strains 6 generations into the lab *w*^*1118*^ strain, and carefully controlled population densities, as described in the “Methods”. We analyzed the concentrations of different nutrients in whole-body lysates as described above.

**Figure 7:**
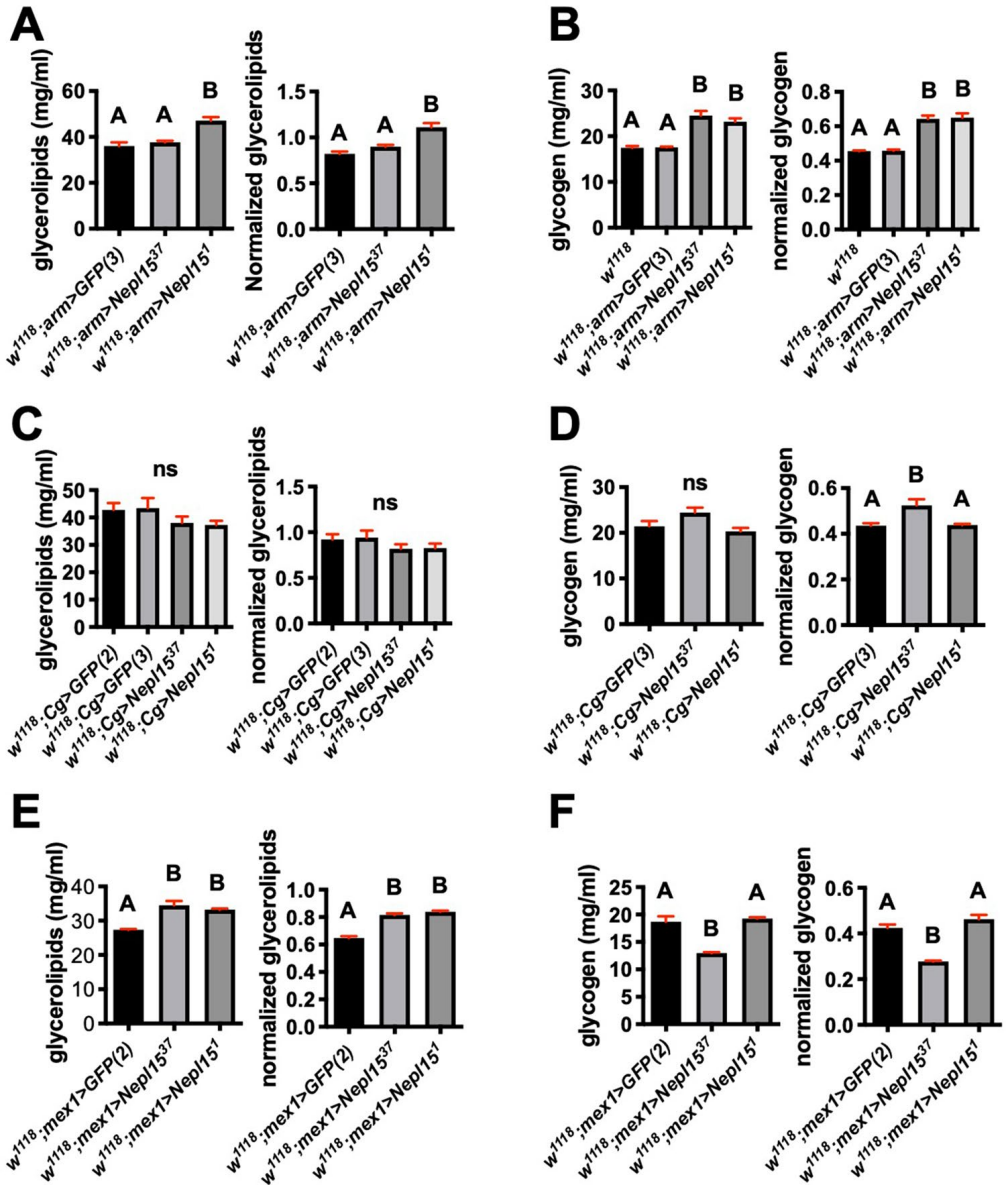
Overexpression of Nepl15 in the whole body or in the midgut results in increased nutrient storage. **(A,C,E)** Glycerolipid concentration (left) and glycerolipid concentration normalized to protein concentration (right) for adult males of the indicated genotypes. **(B,D,F)** Glycogen concentration (left) and glycogen concentration normalized to protein concentration (right) for adult males of the indicated genotypes. Values were analyzed using a one-way ANOVA and were assigned to statistical groups using Tukey’s multiple comparison test. Groups sharing at least one letter are not significantly different; groups not sharing any letter are significantly different (*P* < 0.05). Error bars represent the standard error of mean (SEM). n = 3 biological replicates with 3 technical replicates for all panels.

Both total glycerolipids and glycogen were higher in flies expressing Nepl15 in all tissues using *arm-Gal4* (Fig. 7A,B). In contrast, we did not observe consistent results with the tissue-specific drivers *Cg-Gal4* (Fig. 7C,D) or *mex1-Gal4* (Fig. 7E,F), with glycogen but not glycerolipids elevated only in *Cg* > *Nepl15*^*37*^ flies, and glycerolipids but not glycogen elevated in *mex1* > *Nepl15* flies. Nevertheless, these results suggest that overexpression of Nepl15 is sufficient to promote increased nutrient storage.

Given that *Nepl15* is apparently expressed at highest levels in the fat body, we were concerned by the fact that over-expressing *Nepl15* in the fat body using the *Cg-Gal4* driver did not consistently result in elevated glycerolipids or glycogen. We therefore used *r4-Gal4* to drive expression of either *GFP* or *Nepl15* in the fat body from the late embryonic to adult stage^69^. Interestingly, whereas most of the *r4* > *GFP* embryos laid developed to pupariation (63 $\pm$ 5.2%), most of the *r4* > *Nepl15* embryos laid did not develop to pupariation (7.5 $\pm$ 1.5%). These results suggest that maintaining appropriate levels of Nepl15 in the fat body is critical for proper development.

### Nepl15 is important for nutrient storage during the larval period

A high fat diet (HFD) has been shown to increase the amount of whole-body total glycerolipids in wild type adult flies^70–72^. To further investigate the role of *Nepl15* function in lipid storage, we cultured 5 days old *w*^*1118*^ and *w*^*1118*^; *Nepl15*^*ko*^ adult male flies for 5 days on either normal food (NF) or on a diet containing 10% w/v coconut oil added to NF (10% HFD) (see Materials and Methods). We then used the colorimetric assays described above to determine the concentrations of free glycerol and total glycerolipids.

**Figure 8:**
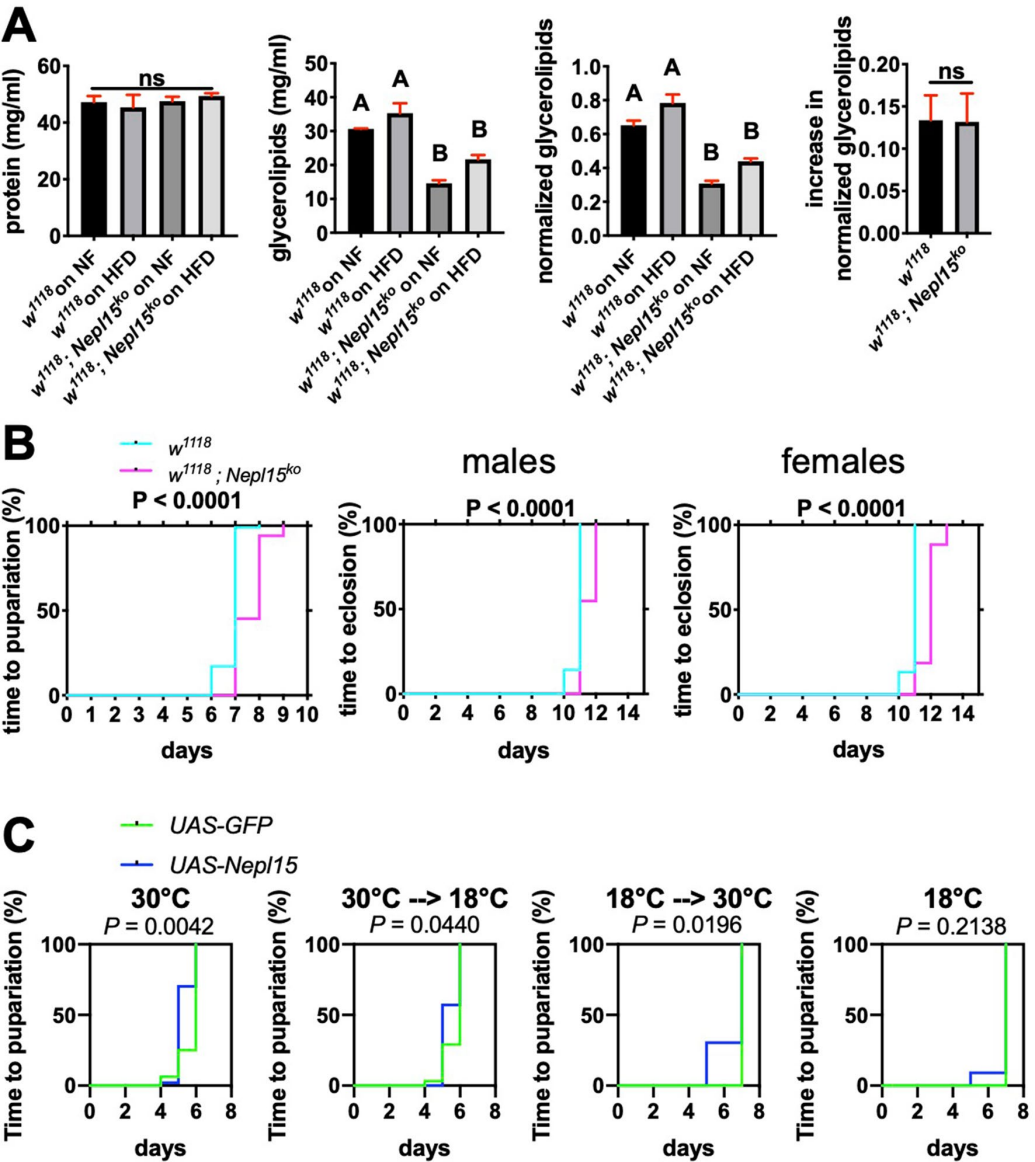
Reductions in nutrient storage in *Nepl15*^*ko*^ adult male flies may reflect defects during the larval stage. **(A)** Protein concentration (left), glycerolipid concentration (center left), and glycerolipid concentration normalized to protein concentration (center right) for adult males of the indicated genotypes subjected to either NF or a HFD. Increase in normalized glycerolipid levels between adult flies of the indicated genotypes subjected to a HFD compared to flies subjected to NF (right). Values were analyzed using a one-way ANOVA and were assigned to statistical groups using Tukey’s multiple comparison test. Groups sharing at least one letter are not significantly different; groups sharing no letters are significantly different (*P* < 0.05). Error bars represent the standard error of mean (SEM). n = 3 biological replicates with 3 technical replicates for all panels. **(B)** Survival curves of time to pupariation (left) and time to eclosion for males (center) and females (right). (Pupariation: n = 93 for w^*1118*^, n = 84 for *w*^*1118*^; *Nepl15*^*ko*^) (Eclosion, males: n = 42 for w^*1118*^, n = 31 for *w*^*1118*^; *Nepl15*^*ko*^) (Eclosion, females: n = 45 for w^*1118*^, n = 43 for *w*^*1118*^; *Nepl15*^*ko*^) (C) Survival curves of time to pupariation for the different temperature regimens for the TARGET experiment. (30 $^\circ$C: n = 16 for *UAS-GFP*, n = 47 for *UAS-Nepl15*). (30 $^\circ$C 18 $^\circ$C: n = 31 for *UAS-GFP*, n = 28 for *UAS-Nepl15*) (18 $^\circ$C 30 $^\circ$C: n = 15 for *UAS-GFP*, n = 23 for *UAS-Nepl15*) (18 $^\circ$C: n = 17 for *UAS-GFP*, n = 11 for *UAS-Nepl15*) Data in B and C were analyzed using a log-rank test, and the *P* values are shown on the graphs.

Exposure to a HFD elevated the whole-body glycerolipid concentrations in both *w*^*1118*^ and *w*^*1118*^; *Nepl15*^*ko*^ adult males compared to flies cultured on NF, though the differences were not statistically significant (Fig. 8A). In contrast, the whole-body glycerolipid concentrations were significantly less in the HFD-fed *w*^*1118*^; *Nepl15*^*ko*^ males as compared to either the *w*^*1118*^ adult male flies fed on a HFD or on NF. Nevertheless, the change in glycerolipid concentrations resulting from a HFD vs. NF were not significantly different between *w*^*1118*^ and *w*^*1118*^; *Nepl15*^*ko*^ adult males. These results suggest that *Nepl15* is important during the larval stage for nutrient uptake, that adult *w*^*1118*^; *Nepl15*^*ko*^ flies start out with a deficit of stored nutrients, and that *Nepl15* has a less important role in nutrient storage during the adult stage.

Glycogen, TAG, trehalose and total protein levels increase^62,73^ as *Drosophila* larvae take up nutrients to reach a critical weight during its third instar stage, at which they have stored enough nutrients and grown to a sufficient size to complete metamorphosis. Larvae can reach the critical weight faster if nutrients are readily available, whereas when nutrients are in short supply the larval period is prolonged^74–76^.

If *Nepl15* is important during larval stages for nutrient storage, then *w*^*1118*^; *Nepl15*^*ko*^ larvae should take longer to reach metamorphosis. In multiple separate trials, the median time to pupariation was significantly longer for the *w*^*1118*^; *Nepl15*^*ko*^ flies compared to *w*^*1118*^ flies (one representative example is shown in Fig. 8B) with a ~ 24-h delay in the time it took for *w*^*1118*^; *Nepl15*^*ko*^ larvae to initiate pupariation (median time: 8 days) compared to *w*^*1118*^ larvae (median time: 7 days). Once the pupal stage was reached, there was no further delay in time to eclosion *w*^*1118*^ (median time: 11 days) and *w*^*1118*^; *Nepl15*^*ko*^ flies (median time: 11–12 days). These results support the idea that *w*^*1118*^; *Nepl15*^*ko*^ larvae require more time to reach critical weight, likely because of their reduced ability to store nutrients.

To further test this idea, we used the TARGET system^77^ in combination with *tubulin-Gal4*^78^ to overexpress *Nepl15* in all cells (1) throughout development (embryonic to adult stages cultured at 30 $^\circ$C), (2) during just the embryonic and larval stages (embryonic and larval stages cultured at 30 $^\circ$C, then shifted to 18 $^\circ$C for pupal through adult stages), (3) during just the pupal and adult stages (embryonic and larval stages cultured at 18 $^\circ$C, then shifted to 30 $^\circ$C for pupal through adult stages), or (4) no overexpression during any stage (embryonic to adult stages cultured at 18 $^\circ$C).

When we assayed time to pupariation (Fig. 8C), we found that the time to pupariation for flies overexpressing Nepl15 during the embryonic and larval stages (30 $^\circ$C and 30 $^\circ$C  -->  18 $^\circ$C) was approximately a day shorter (median time to pupariation = 5 days) compared to controls expressing GFP (median time to pupariation = 6 days). In addition, the time to pupariation for flies overexpressing Nepl15 only during pupal and adult stages (18 $^\circ$C --> 30 $^\circ$C) was statistically significantly shorter, though the median time to pupariation was the same (7 days). Time to pupariation for flies never overexpressing Nepl15 (18 $^\circ$C) did not differ from control flies expressing GFP. However, when we assayed for sensitivity to starvation, we found no significant differences for any of our treatments (Figure S10). These data do not support the hypothesis that Nepl15 is more important for nutrient storage during the larval period than the adult period, but they are consistent with the idea that Nepl15 has a role in regulating nutrient storage during the larval period.

### *Nepl15* does not affect *thor*, *Akh* or *AkhR* expression

In theory, *Nepl15*^*ko*^ flies could show reductions in stored lipids and carbohydrates due to reduced activity in processes that lead to nutrient storage or to hyperactivity in processes that lead to nutrient mobilization. In flies and other insects, the insulin receptor (InR) signaling pathway promotes nutrient storage, and the adipokinetic hormone (Akh) signaling pathway promotes nutrient mobilization^79^.

**Figure 9:**
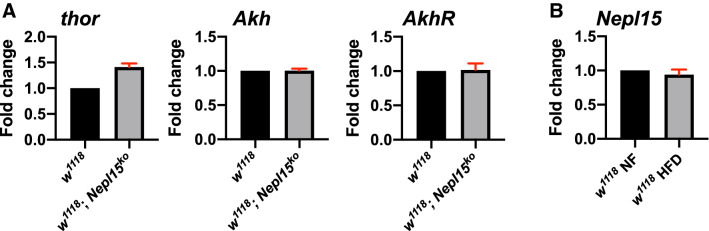
InR and AkhR signaling are not affected in *Nepl1*^*ko*^ males. RT-qPCR data to show relative abundance of transcripts of the indicated genes in *w*^*1118*^ and *Nepl15*^*ko*^ adult male flies. Data are displayed as fold differences compared to *w*^*1118*^. ΔCt values were analyzed using an unpaired t test. None of the differences was significant. Error bars represent the standard error of mean (SEM). n = 3 biological replicates with 3 replicates each for all.

Output of the InR can be monitored by transcription of *thor*, which encodes 4E Binding Protein (4EBP), a direct transcriptional target of dFOXO, a transcription factor that is negatively regulated by InR signaling^80,81^. Furthermore, transcription of *Akh* and the *Akh Receptor* (*Akhr*) are affected by nutrient availability as well as by activity of Insulin Producing Cells (IPCs), Akh Producing Cells (APCs) and activity of cells that express the neuropeptide Allatostatin A^82–86^. To determine whether *Nepl15*^*ko*^ mutations affect transcription of *thor*, *Akh* or *AkhR*, we performed quantitative qPCR on *w*^*1118*^ and *w*^*1118*^*; Nepl15*^*ko*^ whole adult males. We observed a modest but non-significant increase in *thor* transcript levels, suggesting a decrease in InR signaling in the absence of *Nepl15*, but no changes in *Akh* or *AkhR* transcript levels (Fig. 9A).

A HFD is known to alter expression of transcripts from hundreds of *Drosophila* genes^87^. To determine whether a HFD affects *Nepl15* transcription, we performed qPCR on *w*^*1118*^ adult males subjected to a HFD versus NF. We detected no difference in *Nepl15* transcription in flies raised on a HFD versus NF (Fig. 9B).

## Discussion

We have identified a role for the *D. melanogaster* family member Nepl15 in lipid and carbohydrate storage. *Nepl15* transcripts are enriched in the fat body, particularly in larvae, and *Nepl15*^*ko*^ adults show reductions in glycerolipids and glycogen, as well as increased susceptibility to starvation, while overexpressing Nepl15 results in increases in glycerolipids and glycogen. The fact that *Nepl15*^*ko*^ adults can accumulate glycerolipids on a high fat diet at the same apparent rate as *w*^*1118*^ flies, coupled with the increase in developmental time for *Nepl15*^*ko*^ larvae compared to *w*^*1118*^ larvae, suggests that Nepl15 functions during larval development to promote nutrient storage.

There appear to be differences between adult males and adult females, both in *Nepl15* expression levels, and in the effects of the *Nepl15*^*ko*^ mutation on lipid and carbohydrate storage. Recent studies have found intriguing differences in triglyceride storage and metabolism between males and females, as well as in expression of genes involved in these processes^88–91^. Consistent with these studies, our results suggest that Nepl15 has a sex-specific role in regulating nutrient storage.

Analysis of the Nepl15 protein sequence, together with molecular modeling, suggest that Nepl15 is secreted. One other *D. melanogaster* M13 family member, Nep2, has been experimentally shown to be secreted^92^, and there is a secreted version of a vertebrate Neprilysin called Membrane Metallopeptidase Like 1 (MMEL1) that is generated by alternative splicing^93–95^. In addition, *Hs*Neprilysin is itself found in soluble form in blood, urine and cerebrospinal fluid (reviewed in^96^). This analysis has also shown that critical catalytic residues differ in Nepl15 in ways that suggest that it lacks peptidase catalytic activity. One mammalian M13 family member, PHEX, has been experimentally shown to bind substrates and prevent their cleavage by other peptidases, a role that does not require catalytic residues^97,98^. It is possible that Nepl15 similarly binds to substrates to prevent their cleavage. The fact that these differences in the catalytic site are conserved across the evolution of the *Drosophila* genus, suggests that these differences are essential for this protein’s function.

Existing evidence indicates that invertebrate M13 metalloendopeptidases, like most of their vertebrate counterparts, prefer to cleave N-terminal to bulky hydrophobic residues (reviewed in^1,3^, as well as cleavage patterns of predicted *D. melanogaster* M13 target peptides^99^ and references therein). The fact that *Drosophila* genus Nepl15 homologs appear to share a hydrophobic S1′ subsite bolsters the idea that Nepl15 could bind and sequester substrates of catalytic M13 family members, preventing hydrolysis by catalytically active peptidases, and leaving them available to continue performing their functions.

What substrates might be targets of *Drosophila* Nepl15? The list includes Neuropeptide F (NPF) and short NeuroPeptide F (sNPF), counterparts to the mammalian orexigenic NeuroPeptide Y (NPY), the *D. melanogaster* tachykinins DTK and Leucokinin^100–106^, Allatostatin (*D. melanogaster* galanin)^4,86^, and Drosulfakinins (*D. melanogaster* cholecystokinins)^8,107–109^. All of these *D. melanogaster* peptides have known or predicted Neprilysin cleavage sites^99^. Many of them affect activity of InR and AkhR pathways, and vice versa^10,13–15,110^. Identification of which of these or other neuropeptides might have their activitie(s) regulated by Nepl15, as well as the mechanisms by which Nepl15 affects nutrient storage, awaits further experimentation.

The work described here provides evidence for a novel mechanism by which neuropeptide activity is regulated by M13 family peptidases in the context of lipid and carbohydrate metabolism. Further study is likely to inform thinking about how M13 family members such as Neprilysin function in diabetes, and how their activities may be controlled to treat diabetes.

## Methods

### D. melanogaster strains

*Nepl15* knock-out flies (*w*^*1118*^; *Nepl15*^*ko*^) were generated by ends-out homologous recombination^57^ and verified by genomic PCR and sequencing. *UAS-Nepl15* flies were generated by cloning the Nepl15 cDNA into the pUAST vector. *UAS-Nepl15*^*37*^ and *UAS-Nepl15*^*1*^ are insertions on the 2nd and the 3rd chromosomes, respectively. The *armadillo-Gal4* (*arm-Gal4*, BL #1560) and *Cg-Gal4* (BL # 7011) drivers as well as the *UAS-GFP.nls* (BL #4775 and BL #4776) strains were obtained from the Bloomington *Drosophila* Stock Center. The *mex1-Gal4* strain was a kind gift from Graham H. Thomas.

### *D. melanogaster* husbandry

All *D. melanogaster* stocks were maintained at 25 °C on normal yeast-cornmeal food (ingredients: 337.5 g Yeast, 195 g soy flour, 1425 g cornmeal, 95 g *Drosophila* food grade Agar type II, 900 g malt extract, 1.5 g molasses, 100 ml propionic acid, 250 ml 10% Tegosept and 25 L tap water) unless mentioned otherwise. High fat diet (HFD) was prepared by adding 10% (w/v) liquid coconut oil (Spectrum, organic virgin coconut oil) to freshly prepared normal food (NF). To minimize background genetic effects *Nepl15*^*ko*^, *UAS-Nepl15*, *UAS-GFP*, *arm-Gal4*, *Cg-Gal4* and *mex1-Gal4* strains were backcrossed to *w*^*1118*^ for six generations.

Population densities were controlled by collecting embryos 0–12 h after egg laying (AEL) laid by 2- to 5-day-old control and experimental flies. 100 embryos of each strain were seeded into vials containing 10 mL of food and allowed to develop at 25 °C. Time to pupariation was determined by counting the number of pre-pupae/pupae formed within a given 12-h time period. Time to eclosion was similarly determined by counting the number of adults that emerged within a given 12-h time period. Newly emerged adult male and female flies were collected every 12 h and kept separately in groups of 20 in vials containing regular food at 25 °C for 3–5 days. Age-matched 3 to 5 day-old flies were subsequently used for RT-qPCR, Bradford, triglyceride and glycogen assays, and starvation assays.

### Molecular modeling

Alignments of the predicted Nepl15 sequence to other M13 family members were generated using Clustal Omega. Molecular modeling of Nepl15 was performed using SwissModel and the models were analyzed using MolProbity. The Swiss-PdbViewer was used to further analyze the models and to generate the images of the Nepl15 structure.

### Quantitative RT-PCR

RNA was extracted from tissues using TRI reagent (Sigma-T9424) and cDNA synthesis was performed using iScript Reverse Transcriptase Supermix for RT-qPCR (Bio-Rad-1708841). Primers (see list below) either had sequences based on previous publications or were designed using the Integrated DNA Technology qPCR primer designing tool and were checked for their efficiency and any off-target amplification.

**Table:** 

*Nepl15*	FP-CAGCTGTACTGCACCGATTA RP-CGAACTGTTGCGAATTGGATAG
*Akh*^111^	FP-ATGAATCCCAAGAGCGAAGTCCTC RP-CTACTCGCGGTGCTTGCACTCCAG
*AkhR*^86^	FP-CACACCTCGCTGTCCAATC RP-CATCACCTGGCCTCTTCCA
*Thor (4E-BP)*	FP-CACTCCTGGAGGCACCA RP-GAGTTCCTCAGCAAGCAA
*RpL32 (rp49)*	FP-CCAAGCACTTCATCCGCCACC RP-GCGGGTGCGCTTGTTCGATCC

Quantitative PCR was performed using SsoFast EvaGreen Supermix (Bio-Rad-172-5201) on a Bio-Rad MiniOpticon Real-Time PCR System. RT-qPCR values for a transcript of interest were normalized to values for *RpL32* transcripts in the same samples. The final fold-changes were calculated by the 2^(− ΔΔCT)^ method^112^. Experiments complied with the MIQE guidelines^113^.

### Fat body staining for glycogen (PAS) and triglycerides (Nile Red)

Third instar larval fat bodies were dissected in ice cold 1 × PBS (10 × PBS:1.37 M NaCl, 27 mM KCl, 100 mM Na_2_HPO_4,_ 18 mM KH_2_PO_4_, pH 7.4), and fixed in 4% paraformaldehyde in PBS for 20 min at room temperature (RT). For Periodic acid Schiff (PAS) staining tissue was washed in 1% BSA in PBS, incubated in periodic acid solution (Sigma-3951, 1 g/dl) for 5 min at room temperature, washed with 1% BSA in PBS incubated in Schiff’s reagent (Sigma-3952) for 15 min at room temperature and again washed with 1% BSA in PBS. For Nile Red staining tissue was washed in PBS, incubated in PBS containing 0.2 µg/ml Nile Red (Sigma-072485) and 0.5 µg/ml Hoescht 33342 (Sigma-94403) in PBS. Stained tissue was mounted in 80% glycerol (PAS staining) or in 80% plus 5% propyl gallate (Sigma-P3130). Images of PAS-stained fat bodies were taken using bright-field microscopy. Images of Nile-Red-stained fat bodies were obtained on a Leica TCS SC5 Laser Scanning Confocal Microscope. Confocal images are projections of multiple sections obtained using FIJI is Just Image J. The FIJI Analyze Particles function was used to determine the total area of lipid droplets stained by Nile Red, and % area was determined by dividing the total area of lipid droplets by the total area of the fat body.

### Thin layer chromatography

10 adult males were homogenized in 250 µl chloroform:methanol solvent (2:1) on ice and centrifuged to remove debris. 2 µl of the resultant lipid supernatant was pipetted on a silica gel plate (Sigma-60805) and separated using a diethyl ether:hexane solvent system. Lipids were visualized using a heat activated phosphomolybdic acid solution (Sigma-02553).

### Feeding assays

Capillary Feeding (CAFE) and food labeling with radioactive tracer assays were performed as described^60^.

### Colorimetric assays for glycerolipids, glycogen, trehalose and glucose

Colorimetric Assays for these metabolites were performed essentially as described^61^. In all cases three biological and three technical replicates were included.

The Sigma GAGO-20 (G0885) kit was used to determine free glucose concentrations, to determine glycogen concentrations from homogenates treated with amyloglucosidase (Sigma A160220) and to determine trehalose concentrations from homogenates treated with porcine trehalase (Sigma-T8778-1UN). A D-( +)-Glucose solution (Sigma-G3285), a glycogen solution (Ambion-9510) and a trehalose solution (Sigma-90208) were used to generate standard curves. Absorbance was measured at 540 nm using an Eon BioTek plate reader.

Triglyceride Reagent (Sigma-T2449) was used to determine triglyceride concentrations; a glycerol solution (Sigma-G7793) was used to generate standard curves. Absorbance was measured at 540 nm using an Eon BioTek plate reader.

Bradford Reagent (Sigma-B6916) was used to determine protein concentrations; Bovine Serum Albumin (Sigma-A7906) was used to generate standard curves. Absorbance was measured at 595 nm using the Eon BioTek plate reader.

Briefly, for assays on whole adult flies, 10 age matched adult flies were rinsed with ice cold 1× PBS and homogenized in 200 µl of ice cold 1× PBS, 1× PBST or Trehalase Buffer (5 mM Tris pH 6.6, 137 mM NaCl, 2.7 mM KCl). 20 μl of the homogenized sample was cleared by centrifugation, diluted 2× with ice-cold buffer, and used to measure protein content using a Bradford assay. Remaining homogenates were heat-inactivated (10 min at 70 °C) and cleared by centrifugation. Resulting supernatants were either not diluted (free glucose, trehalose assays, glycerolipids), or were diluted 15× (males, glycogen), 20× (females, glycogen) with ice cold buffer prior to performing the assay.

For assays on hemolymph, 50 adult male flies were punctured in the thorax with a needle and placed in a 0.65 ml microcentrifuge tube with a small hole in the bottom. The 0.65 ml tube was placed in a 1.7 ml microcentrofuge tube and spun for 2 min at 10,000 × *g* generating approximately 1 µl of transparent hemolymph, which was mixed with 19 µl ice-cold 1XPBS. 3 µl was used to measure free glucose concentrations, and 3 µl was used to determine protein concentration.

### Metabolic rate assays

A few flies were placed in a sealed chamber containing soda lime to adsorb CO_2_ and a capillary to measure changes in pressure. The rate of change in the height of liquid in the capillary provides a measure of CO_2_ production and therefore metabolic rate. The amount of CO_2_ produced was normalized by dividing by the total number of flies in the sealed chamber.

### TARGET assays

Flies were allowed to lay for 10 h at 25 $^\circ$C. 100 embryos were transferred to a new vial of complete media and allowed to develop at the permissive temperature for Gal80ts (18 $^\circ$C) or at the non-permissive temperature (30 $^\circ$C) until the first pupae were noted. At this point vials were either retained at the original temperature, were transferred from the permissive to the non-permissive temperature (18 $^\circ$C  --> 30 $^\circ$ C), or were transferred from the non-permissive temperature to the permissive temperature (30 $^\circ$C --> 18 $^\circ$C).

### Starvation assays

Newly emerged adult flies were incubated separately in vials containing normal food at 25 °C for three days, then transferred to starvation media (1% food grade agar) and kept at 25 °C. The number of fly deaths due to starvation was recorded by monitoring the vials every 6 h.

## Supplementary Information

Supplementary Figures.

Supplementary Legends.
