# Author Correction: The *Drosophila melanogaster* Neprilysin Nepl15 is involved in lipid and carbohydrate storage

**DOI:** 10.1038/s41598-021-84100-4

**Published:** 2021-02-25

**Authors:** Surya Banerjee, Christine Woods, Micheal Burnett, Scarlet J. Park, William W. Ja, Jennifer Curtiss

**Affiliations:** 1 New Mexico State University, Las Cruces, NM USA; 2 The Scripps Research Institute Florida, Jupiter, FL USA

Correction to:* Scientific Reports*, 10.1038/s41598-021-81165-z, published online 22 January 2021.

In the original version of this Article, the author William W. Ja was incorrectly indexed. This error has now been corrected.

