# Supplementary material for: The Drosophila melanogaster Neprilysin Nepl15 is involved in lipid and carbohydrate storage
Source: Sci Rep. 2021 Jan 22;11:2099. doi: 10.1038/s41598-021-81165-z (PMC7822871; doi:10.1038/s41598-021-81165-z)
Supplement: Supplementary file 1 — Supplementary Figures. [file 41598_2021_81165_MOESM1_ESM.pdf]

Figure S1

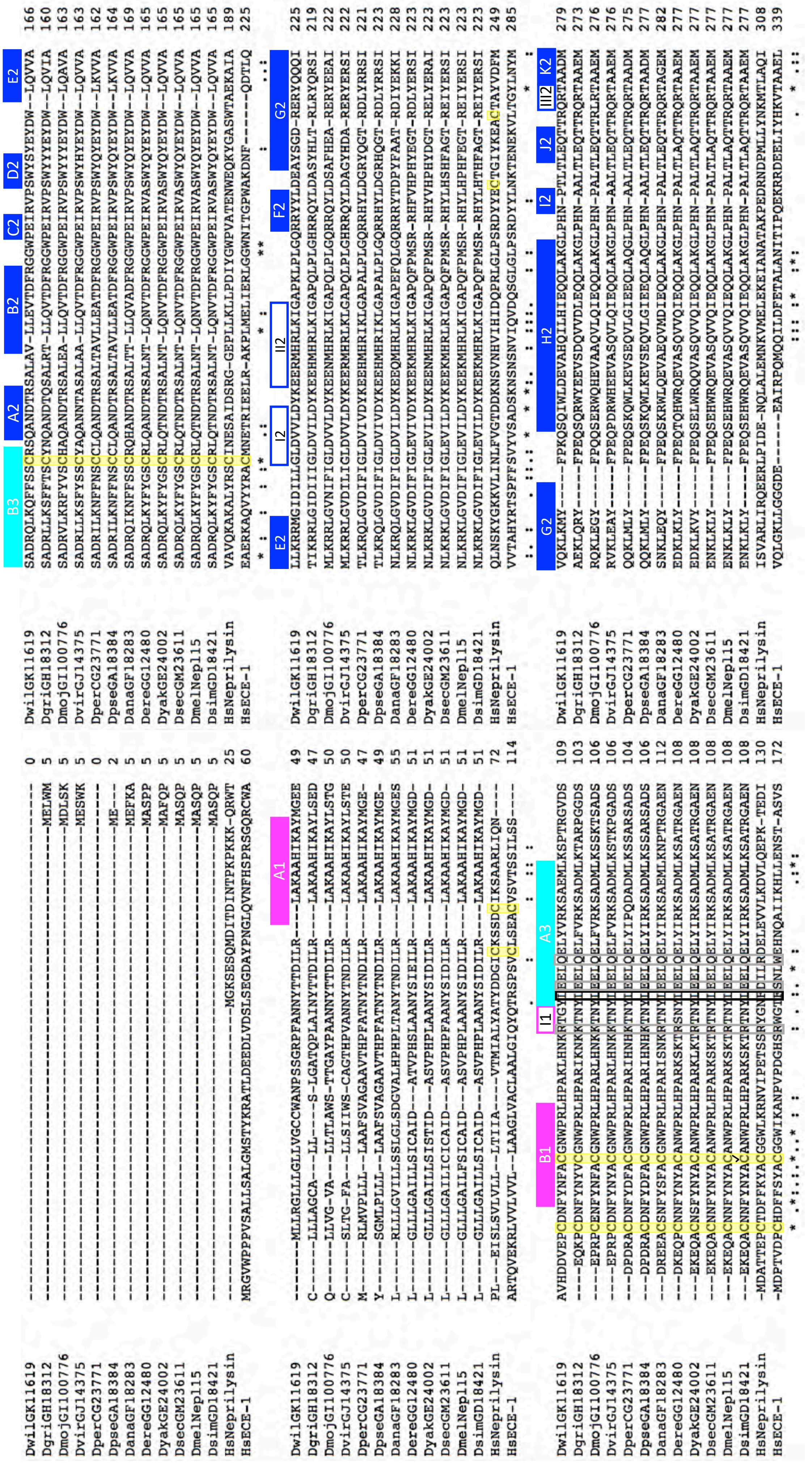

DwilGK11619
DgriGH18312
DmojGI100776
DvirGJ14375
DperCG23771
DpseGA18384
DanaGF18283
DereGG12480
DyakGE24002
DsecGM23611
DmelNep115
DsimGD18421
HsNeprilysin
HsECE-1
A1
B1
A3
I1
G2
E2
I2
II2
H2
B3
A2
B2
C2
D2
E2
F2
G2
I2
J2
III2
K2

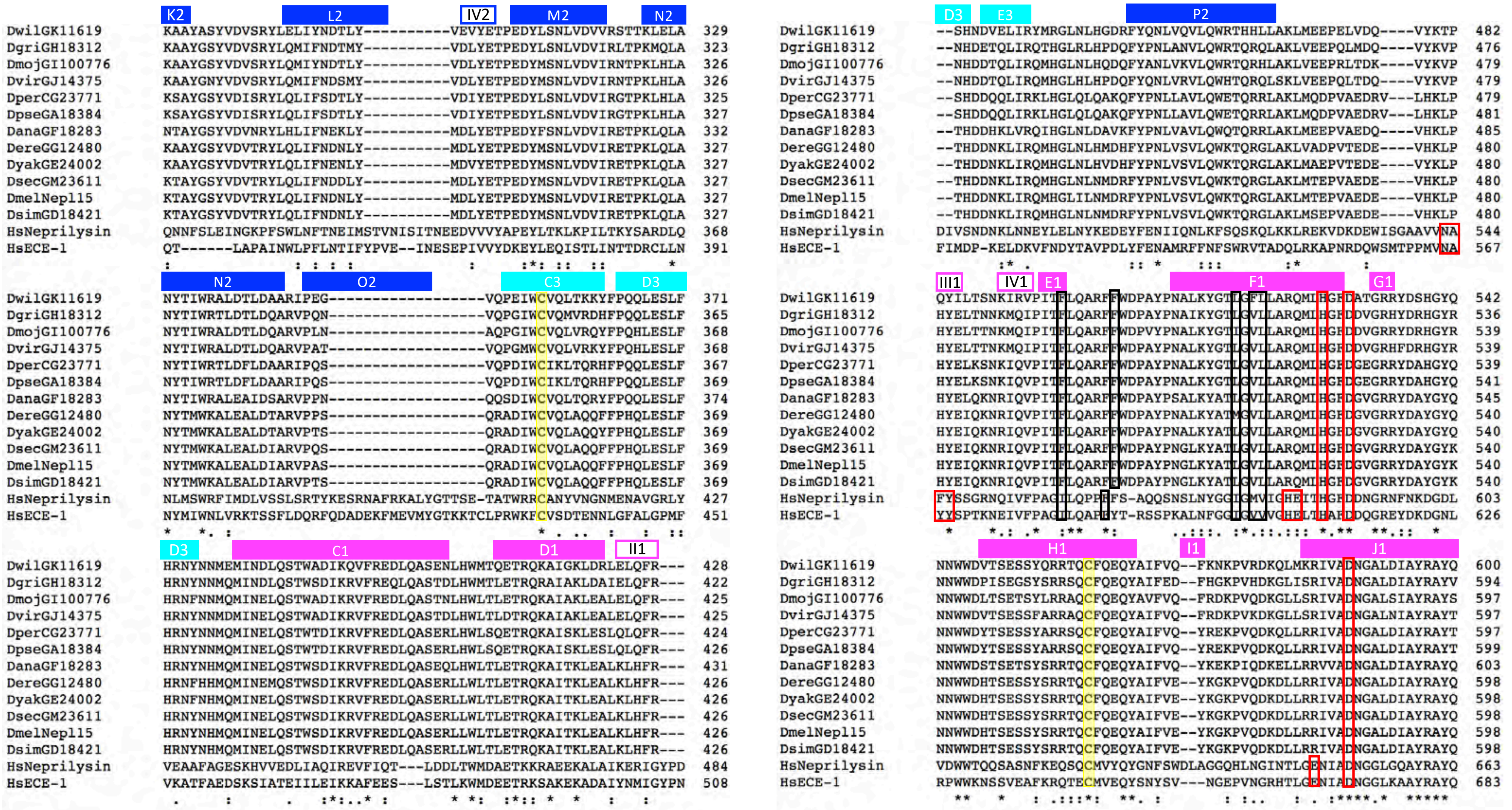
K2 L2 IV2 M2 N2
DwilGK11619 KAAYASYVDVSRYLELIYNDTLY-----------VEVYETPEDYLSNLVDVVRSTTKLELA 329
DgriGH18312 KAAYGSYVDVSRYLQMIFNDTMY-----------VDLYETPEDYLSNLVDVIRLTPKMQLA 323
DmojGI100776 KAAYGSYVDVSRYLQMIYNDTLY-----------VDLYETPEDYMSNLVDVIRNTPKLHLA 326
DvirGJ14375 KAAYGNYVDVSRYLQMIFNDSMY-----------VDLYETPEDYMSNLVDVIRNTPKLHLA 326
DperCG23771 KSAYGSYVDISRYLQLIFSDTLY-----------VDIYETPEDYLSNLVDVIRGTPKLHLA 325
DpseGA18384 KSAYGSYVDISRYLQLIFSDTLY-----------VDIYETPEDYLSNLVDVIRGTPKLHLA 327
DanaGF18283 NTAYGSYVDVNRYLHLIFNEKLY-----------MDLYETPEDYFSNLVDVIRETPKLQLA 332
DereGG12480 KAAYGSYVDVTRYLQLIFNDNLY-----------MDLYETPEDYMSNLVDVIRETPKLQLA 327
DyakGE24002 KAAYGSYVDVTRYLQLIFNENLY-----------MDVYETPEDYMSNLVDVIRETPKLQLA 327
DsecGM23611 KTAYGSYVDVTRYLQLIFNDDLY-----------MDLYETPEDYMSNLVDVIRETPKLQLA 327
DmelNepl15 KTAYGSYVDVTRYLQLIFNDNLY-----------MDLYETPEDYMSNLVDVIRETPKLQLA 327
DsimGD18421 KTAYGSYVDVTRYLQLIFNDNLY-----------MDLYETPEDYMSNLVDVIRETPKLQLA 327
HsNeprilysin QNNFSLEINGKPFSWLNFTNEIMSTVNISITNEEDVVVYAPEYLTKLKPILTKYSARDLQ 368
HsECE-1 QT------LAPAINWLPFLNTIFYPVE--INESEPIVVYDKEYLEQISTLINTTDRCLLN 391
N2 O2 C3 D3
DwilGK11619 NYTIWRALDTLDAARIPEG-------------------VQPEIWCVQLTKKYFPQQLESLF 371
DgriGH18312 NYTIWRTLDTLDQARVPQN-------------------VQPGIWCVQMVRDHFPQQLESLF 365
DmojGI100776 NYTIWRALDTLDQARVPLN-------------------AQPGIWCVQLVRQYFPQHLESLF 368
DvirGJ14375 NYTIWRALDTLDQARVPAT-------------------VQPGMWCVQLVRKYFPQHLESLF 368
DperCG23771 NYTIWRTLDFLDAARIPQS-------------------VQPDIWCIKLTQRHFPQQLESLF 367
DpseGA18384 NYTIWRTLDFLDAARIPQS-------------------VQPDIWCIKLTQRHFPQQLESLF 369
DanaGF18283 NYTIWRALEAIDSARVPPN-------------------QQSDIWCVQLTQRYFPQQLESLF 374
DereGG12480 NYTMWKALEALDTARVPPS-------------------QRADIWCVQLAQQFFPHQLESLF 369
DyakGE24002 NYTMWKALEALDTARVPTS-------------------QRADIWCVQLAQQYFPHQLESLF 369
DsecGM23611 NYTMWKALEALDIARVPQS-------------------QRADIWCVQLAQQFFPHQLESLF 369
DmelNepl15 NYTMWKALEALDIARVPAS-------------------QRADIWCVQLAQQFFPHQLESLF 369
DsimGD18421 NYTMWKALEALDIARVPTS-------------------QRADIWCVQLAQQFFPHQLESLF 369
HsNeprilysin NLMSWRFIMDLVSSLSRTYKESRNAFRKALYGTTSE-TATWRRCANYVNGNMENAVGRLY 427
HsECE-1 NYMIWNLVRKTSSFLDQRFQDADEKFMEVMYGTKKTCLPRWKFCVSDTENNLGFALGPMF 451
D3 C1 D1 II1
DwilGK11619 HRNYNNMEMINDLQSTWADIKQVFREDLQASENLHWMTQETRQKAIGKLDRLELQFR--- 428
DgriGH18312 HRNYNNMQMINDLQSTWSDIKRVFREQLQASTDLHWMTLETRQRAIAKLDAIELQFR--- 422
DmojGI100776 HRNFNNMQMINELQSTWADIKRVFREDLQASTNLHWLTLETRQKAIAKLEALELQFR--- 425
DvirGJ14375 HRNYNNMDMINELQSTWADIKRVFREDLQASTDLHWLTLDTRQKAIAKLEALELQFR--- 425
DperCG23771 HRNYNNMQMINELQSTWTDIKRVFREDLQASERLHWLSQETRQKAISKLESLQLQFR--- 424
DpseGA18384 HRNYNNMQMINELQSTWTDIKRVFREDLQASERLHWLSQETRQKAISKLESLQLQFR--- 426
DanaGF18283 HRNYNHMQMINELQSTWSDIKRVFREDLQASEQLHWLTLETRQKAITKLEALKLHFR--- 431
DereGG12480 HRNFHHMQMINEMQSTWSDIKRVFREDLQASERLLWLTLETRQKAITKLEALKLHFR--- 426
DyakGE24002 HRNFNHMQMINELQSTWSDIKRVFREDLQASERLLWLTLETRQKAITKLEALKLHFR--- 426
DsecGM23611 HRNYNHMQMINELQSTWSDIKRVFREDLQASERLLWLTLETRQKAITKLEALKLHFR--- 426
DmelNepl15 HRNYNHMQMINELQSTWSDIKRVFREDLQASERLLWLTLETRQKAITKLEALKLHFR--- 426
DsimGD18421 HRNYNHMQMINELQSTWSDIKRVFREDLQASERLLWLTLETRQKAITKLEALKLHFR--- 426
HsNeprilysin VEAAFAGESKHVVEDLIAQIREVFIQT---LDDLTWMDAETKKRAEEKALAIKERIGYPD 484
HsECE-1 VKATFAEDSKSIATEIILEIKKAFEES---LSTLKWMDEETRKSAKEKADAIYNMIGYPN 508
D3 E3 P2
DwilGK11619 --SHNDVELIRYMRGLNLHGDRFYQNLVQVLQWRTHHLLAKLMEEPELVDQ----VYKTP 482
DgriGH18312 --NHDETQLIRQTHGLRLHPDQFYPNLANVLQWRTQRQLAKLVEEPQLMDQ----VYKVP 476
DmojGI100776 --NHDDTQLIRQMHGLNLHQDQFYANLVKVLQWRTQRHLAKLVEEPRLTDK----VYKVP 479
DvirGJ14375 --NHDDTQLIRQMHGLHLHPDQFYQNLVRVLQWHTQRQLSKLVEEPQLTDQ----VYKVP 479
DperCG23771 --SHDDQQLIRKLHGLQLQAKQFYPNLLAVLQWETQRRLAKLMQDPVAEDRV---LHKLP 479
DpseGA18384 --SHDDQQLIRKLHGLQLQAKQFYPNLLAVLQWETQRRLAKLMQDPVAEDRV---LHKLP 481
DanaGF18283 --THDDHKLVRQIHGLNLDAVKFYPNLVAVLQWQTQRRLAKLMEEPVAEDQ----VHKLP 485
DereGG12480 --THDDNKLIRQMHGLNLHMDHFYPNLVSVLQWKTQRGLAKLVADPVTEDE----VHKLP 480
DyakGE24002 --THDDNKLIRQMHGLNLHVDHFYPNLVSVLQWKTQRGLAKLMAEPVTEDE----VYKLP 480
DsecGM23611 --THDDNKLIRQMHGLNLNMDRFYPNLVSVLQWKTQRGLAKLMTEPVAEDE----VHKLP 480
DmelNepl15 --THDDNKLIRQMHGLILNMDRFYPNLVSVLQWKTQRGLAKLMTEPVAEDE----VHKLP 480
DsimGD18421 --THDDNKLIRQMHGLNLNMDRFYPNLVSVLQWKTQRGLAKLMSEPVAEDE----VHKLP 480
HsNeprilysin DIVSNDNKLNNEYLELNYKEDEYFENIIQNLKFSQSKQLKKLREKVDKDEWISGAAVVNA 544
HsECE-1 FIMDP-KELDKVFNDYTAVPDLYFENAMRFFNFSWRVTADQLRKAPNRDQWSMTPPMVNA 567
III1 IV1 E1 F1 G1
DwilGK11619 QYILTSNKIRVPITFLQARFFWDPAYPNALKYGTLGFLLARQMIHGFDATGRRYDSHGYQ 542
DgriGH18312 HYELTNNKMQIPITFLQARFFWDPAYPNAIKYGTLGVLLARQMIHGFDDVGRHYDRHGYR 536
DmojGI100776 HYELTTNKMQIPITFLQARFFWDPVYPNALKYGTIGVLLARQMIHGFDDVGRRYDRHGYR 539
DvirGJ14375 HYELTTNKMQIPITFLQARFFWDPAYPNALKYGTLGVLLARQMIHGFDDVGRHFDRHGYR 539
DperCG23771 HYELKSNKIQVPITFLQARFFWDPAYPNALKYGTLGVLLARQMIHGFDGEGRRYDAHGYQ 539
DpseGA18384 HYELKSNKIQVPITFLQARFFWDPAYPNALKYGTLGVLLARQMIHGFDGEGRRYDAHGYQ 541
DanaGF18283 HYELQKNRIQVPITFLQARFFWDPAYPSALKYATLGVLLARQMIHGFDGVGRRYDAYGYQ 545
DereGG12480 HYEIQKNRIQVPITFLQARFFWDPAYPNALKYATMGVLLARQMIHGFDGVGRRYDAYGYQ 540
DyakGE24002 HYEIQKNRIQVPITFLQARFFWDPAYPNALKYATLGVLLARQMIHGFDGVGRRYDAYGYQ 540
DsecGM23611 HYEIQKNRIQVPITFLQARFFWDPAYPNGLKYATLGVLLARQMIHGFDGVGRRYDAYGYK 540
DmelNepl15 HYEIQKNRIQVPITFLQARFFWDPAYPNGLKYATLGVLLARQMIHGFDGVGRRYDAYGYK 540
DsimGD18421 HYEIQKNRIQVPITFLQARFFWDPAYPNGLKYATLGVLLARQMIHGFDGVGRRYDAYGYK 540
HsNeprilysin FYSSGRNQIVFPAGILQPPFFS-AQQSNSLNYGGIGMVIGHEITHGFDDNGRNFNKDGDL 603
HsECE-1 YYSPTKNEIVFPAGILQAPFYT-RSSPKALNFGGIGVVVGHELTHAFDDQGREYDKDGNL 626
H1 I1 J1
DwilGK11619 NNWWDVTSESSYQRRTQCFQEQYAIFVQ--FKNKPVRDKQLMKRIVADNGALDIAYRAYQ 600
DgriGH18312 NNWWDPISEGSYSRRSQCFQEQYAIFED--FHGKPVHDKGLISRIVADNGALDIAYRAYV 594
DmojGI100776 NNWWDLTSETSYLRRAQCFQEQYAVFVQ--FRDKPVQDKGLLSRIVADNGALSIAYRAYS 597
DvirGJ14375 NNWWDVTSESSFARRAQCFQEQYAIFVQ--FRDKPVKDKGLLSRIVADNGALNIAYRAYT 597
DperCG23771 NNWWDYTSESSYARRSQCFQEQYAIFVQ--YREKPVQDKQLLRRIVADNGALDIAYRAYT 597
DpseGA18384 NNWWDYTSESSYARRSQCFQEQYAIFVQ--YREKPVQDKQLLRRIVADNGALDIAYRAYT 599
DanaGF18283 NNWWDSTSETSYSRRTQCFQEQYAIFVQ--YKEKPIQDKELLRRVVADNGALDIAYRAYQ 603
DereGG12480 NNWWDHTSESSYSRRTQCFQEQYAIFVE--YKGKPVQDKDLLRRIVADNGALDIAYRAYQ 598
DyakGE24002 NNWWDHTSESSYSRRTQCFQEQYAIFVE--YKGKPVQDKDLLRRIVADNGALDIAYRAYQ 598
DsecGM23611 NNWWDHTSESSYSRRTQCFQEQYAIFVE--YKGKPVQDKDLLRRIVADNGALDIAYRAYQ 598
DmelNepl15 NNWWDHTSESSYSRRTQCFQEQYAIFVE--YKGKPVQDKDLLRRIVADNGALDIAYRAYQ 598
DsimGD18421 NNWWDHTSESSYSRRTQCFQEQYAIFVE--YKGKPVQDKDLLRRIVADNGALDIAYRAYQ 598
HsNeprilysin VDWWTQQSASNFKEQSQCMVYQYGNFSWDLAGGQHLNGINTLGENIADNGGLGQAYRAYQ 663
HsECE-1 RPWWKNSSVEAFKRQTECMVEQYSNYSV---NGEPVNGRHTLGENIADNGGLKAAYRAYQ 683

```
                 J1                         K1          V1    L1          M1
DwilGK11619      KWLKNTAETAAIYDRERLPLLDYSHNQLFYLATAQMYCTDYPD-----SVDIFDELPEQL   655
DgriGH18312      EWLKNTDETSTLYQREQLPLLDNTHNQLFYMAYAQLYCSDYPE-----SVEMFDELPEQI   649
DmojGI100776     KWLRNSAETSATYQRERLPLLEHTENELFFLAYAQLYCSDYPE-----SVELYEELPEPF   652
DvirGJ14375      KWLKNSAETPIIYQRERLPLLDHSENELFYLAYAQLYCSDYPD-----SLEIFDELPEQL   652
DperCG23771      KWLKNSAETMASYQRERLPLLDYSNLQLFYLAYAQLYCSDYPD-----TLERYEELPEQL   652
DpseGA18384      KWLKNSAETMASYQRERLPLLDYSNLQLFYLAYAQLYCSDYPE-----TLERYEELPEQL   654
DanaGF18283      QWFKNAADAPIIYQRERLPLLDYSHNQLFYLGYAQLYCSDYPQ-----SVERFDELPEAL   658
DereGG12480      QWLKNAAETPVIYQRERLPILNHDHNQIFYLGYAQLYCTDYPE-----SLERFDELPEQL   653
DyakGE24002      QWLKNAAETPVIYQRERLPLLDHDHNQIFYLGYAQLYCTDYPE-----SVERFDELPEQL   653
DsecGM23611      QWLKNAAETPVIYQRERLPLLDHDHNQIFYLGYAQLYCTDYPE-----SVERFDELPEQL   653
DmelNep115       QWLKNAAETPVIYQRERLPLLDHDHNQIFYLGYAQLYCTDYPE-----TVERFDELPEQL   653
DsimGD18421      QWLKNAAETPVIYQRERLPLLDHDHNQIFYLGYAQLYCTDYPE-----SVERFDELPEQL   653
HsNeprilysin     NYIKKNGEE------KLLPGLDLNHKQLFFLNFAQVWCGTYRPEYAVNSIKTDVHSPGNF   717
HsECE-1          NWVKKNGAE------HSLPTLGLTNNQLFFLGFAQVWCSVRTPESSHEGLITDPHSPSRF   737
                 ::.::          . ** *   . ::*::  **::*           :    . *  :

                 M1     N1
DwilGK11619      RINTALSNAQPFVNTYSCSYGDKLNARFKCNLY      688
DgriGH18312      RVNTALSNSEHFVNAFSCSRSDKLNARFKCALY      682
DmojGI100776     RVNTALSNSEQFAQAFSCSKSDKLNARFKCALY      685
DvirGJ14375      RVNTALSNSEQFANAFRCSRDDKLNARFKCALY      685
DperCG23771      RINAALSNSQQFTNAYSCPSGSALNARFKCTLY      685
DpseGA18384      RINAALSNSQQFTNAYSCPSGSALNARFKCTLY      687
DanaGF18283      RVNTALSNQQPFANAYSCSREDKVYARFKCTLY      691
DereGG12480      RVNTALSNSQPFANAYSCSREEKLNARFKCTLY      686
DyakGE24002      RINTALSNSQQFANAFGCSREDKLNARFKCTLY      686
DsecGM23611      RVNTALSNSQQFTNAYGCSREEKLNARFKCTLY      686
DmelNep115       RVNTALSNSQQFANAYGCSREEKLNARFKCTLY      686
DsimGD18421      RVNTALSNSQQFANAYGCSREEKLNARFKCTLY      686
HsNeprilysin     RIIGTLQNSAEFSEAFHCRKNSYMNPEKKCRVW      750
HsECE-1          RVIGSLSNSKEFSEHFRCPPGSPMNPPHKCEVW      770
                 *:  :*.*   * : : *    . :    ** ::
```

Figure S2

A

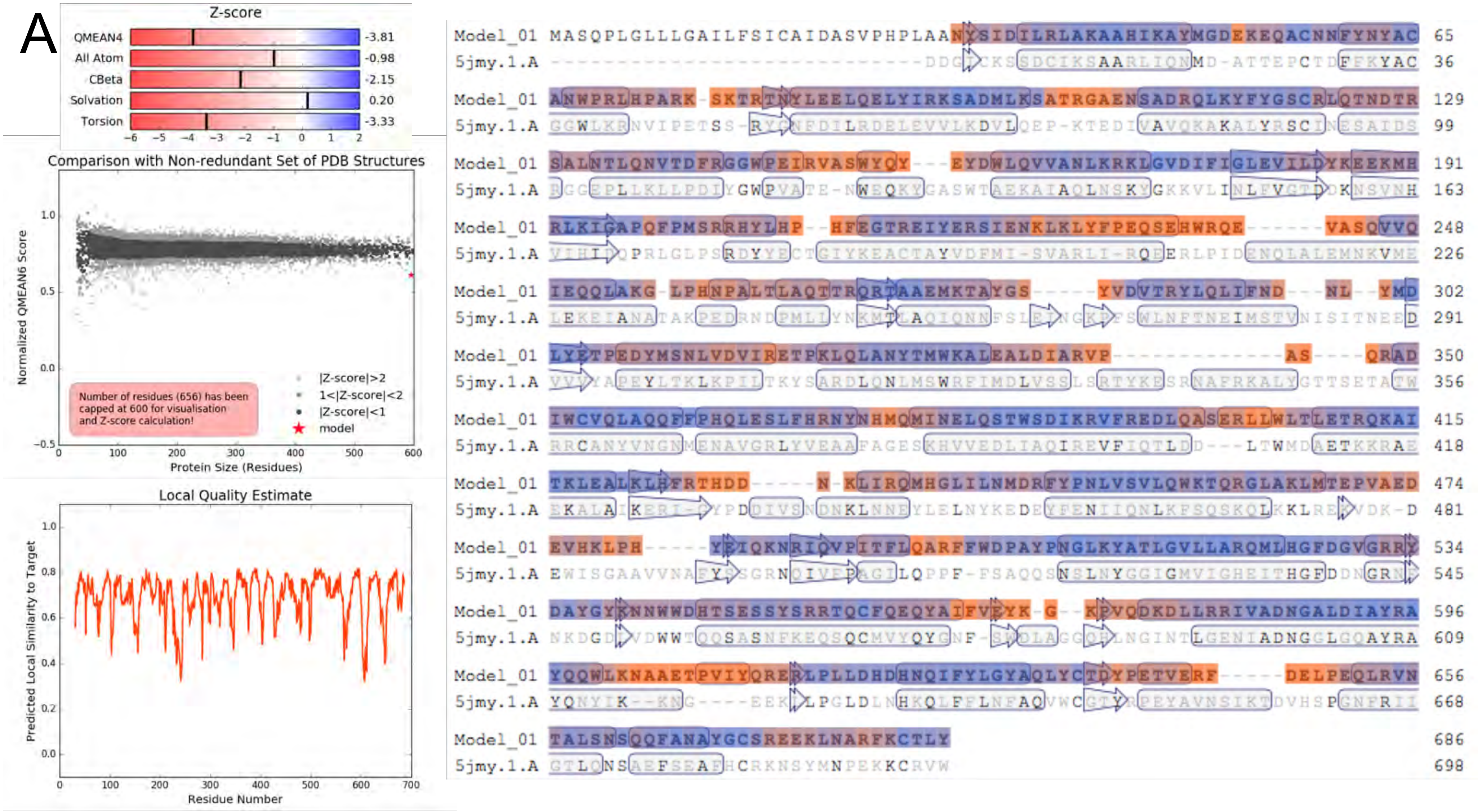
Z-score
QMEAN4
All Atom
CBeta
Solvation
Torsion
-3.81
-0.98
-2.15
0.20
-3.33
Comparison with Non-redundant Set of PDB Structures
Normalized QMEAN6 Score
Protein Size (Residues)
Number of residues (656) has been capped at 600 for visualisation and Z-score calculation!
|Z-score|>2
1<|Z-score|<2
|Z-score|<1
model
Local Quality Estimate
Predicted Local Similarity to Target
Residue Number
Model_01
5jmy.1.A

B

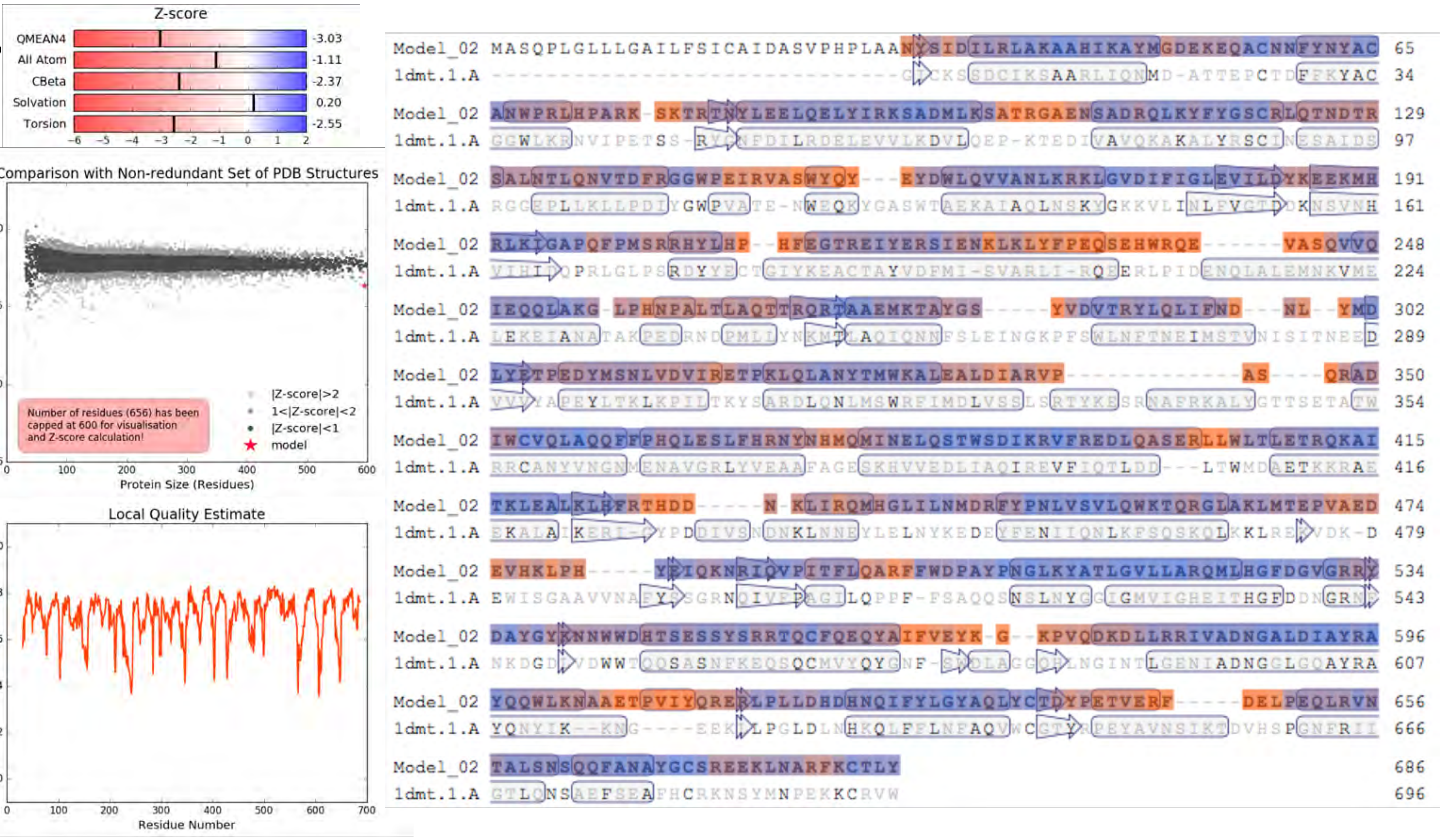

Z-score
QMEAN4 -3.03
All Atom -1.11
CBeta -2.37
Solvation 0.20
Torsion -2.55
Comparison with Non-redundant Set of PDB Structures
Normalized QMEAN6 Score
Protein Size (Residues)
Number of residues (656) has been capped at 600 for visualisation and Z-score calculation!
|Z-score|>2
1<|Z-score|<2
|Z-score|<1
model
Local Quality Estimate
Predicted Local Similarity to Target
Residue Number
Model_02
1dmt.1.A

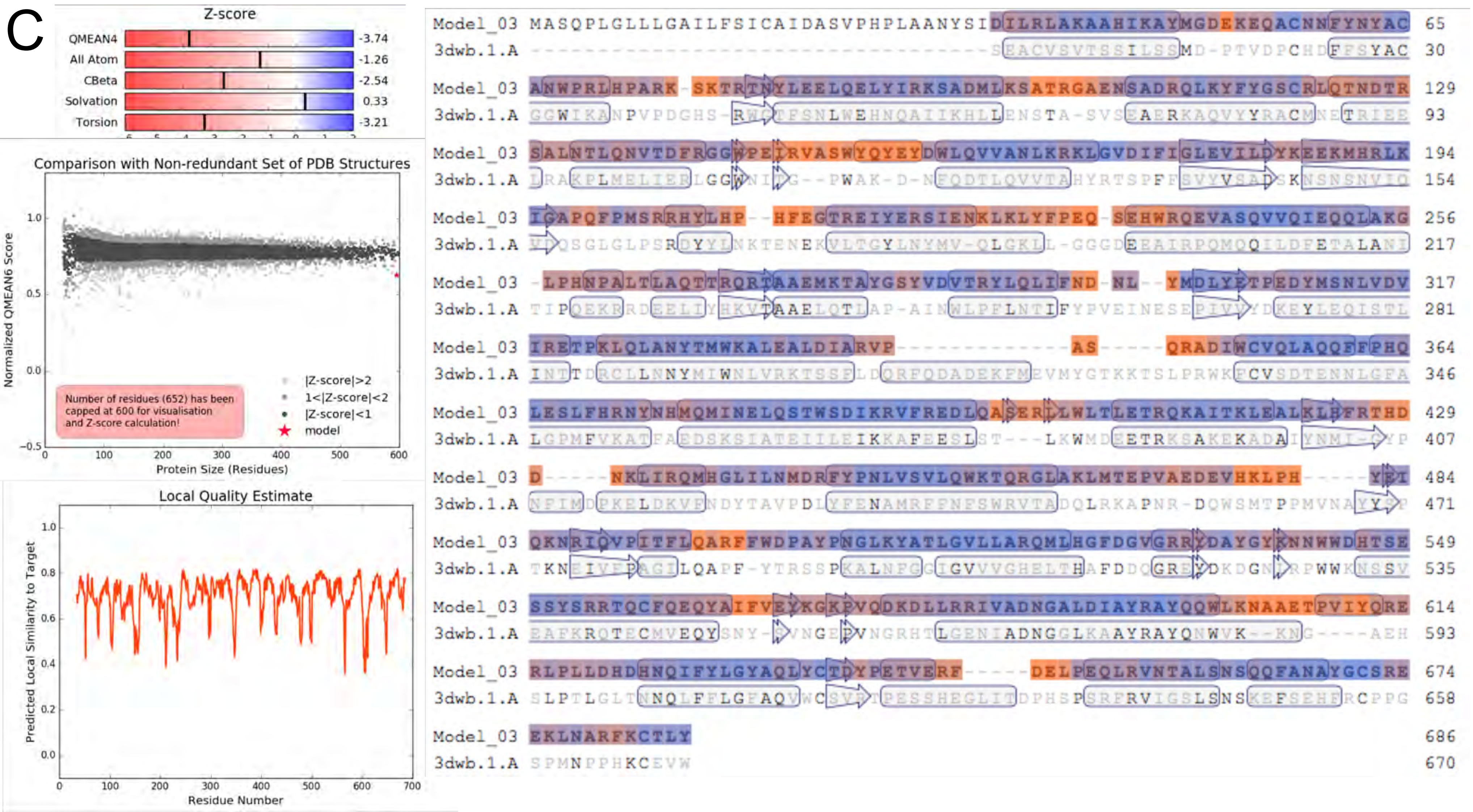
C
Z-score
QMEAN4 -3.74
All Atom -1.26
CBeta -2.54
Solvation 0.33
Torsion -3.21
Comparison with Non-redundant Set of PDB Structures
Normalized QMEAN6 Score
Protein Size (Residues)
|Z-score|>2
1<|Z-score|<2
|Z-score|<1
model
Number of residues (652) has been capped at 600 for visualisation and Z-score calculation!
Local Quality Estimate
Predicted Local Similarity to Target
Residue Number
Model_03 65
3dwb.1.A 30
Model_03 129
3dwb.1.A 93
Model_03 194
3dwb.1.A 154
Model_03 256
3dwb.1.A 217
Model_03 317
3dwb.1.A 281
Model_03 364
3dwb.1.A 346
Model_03 429
3dwb.1.A 407
Model_03 484
3dwb.1.A 471
Model_03 549
3dwb.1.A 535
Model_03 614
3dwb.1.A 593
Model_03 674
3dwb.1.A 658
Model_03 686
3dwb.1.A 670

A Figure S3

Viewing model_01FH-multi.table

Duke Biochemistry
Duke University School of Medicine

When finished, you should [close this window].

Hint: Use File | Save As... to save a copy of this page.

All-Atom Contacts	Clashscore, all atoms:	1.84		99th percentile* (N=1784, all resolutions)
	Clashscore is the number of serious steric overlaps (> 0.4 Å) per 1000 atoms.			
Protein Geometry	Poor rotamers	9	1.55%	Goal: <0.3%
	Favored rotamers	550	94.66%	Goal: >98%
	Ramachandran outliers	9	1.38%	Goal: <0.05%
	Ramachandran favored	609	93.12%	Goal: >98%
	MolProbity score^	1.53		94th percentile* (N=27675, 0Å - 99Å)
	Cβ deviations >0.25Å	13	2.05%	Goal: 0
	Bad bonds:	2 / 5624	0.04%	Goal: 0%
	Bad angles:	75 / 7620	0.98%	Goal: <0.1%
Peptide Omegas	Cis Prolines:	0 / 24	0.00%	Expected: ≤1 per chain, or ≤5%
	Cis nonProlines:	2 / 631	0.32%	Goal: <0.05%

In the two column results, the left column gives the raw count, right column gives the percentage.

* 100th percentile is the best among structures of comparable resolution; 0th percentile is the worst. For clashscore the comparative set of structures was selected in 2004, for MolProbity score in 2006.

^ MolProbity score combines the clashscore, rotamer, and Ramachandran evaluations into a single score, normalized to be on the same scale as X-ray resolution.

#	Alt	Res	High B	Clash > 0.4Å	Ramachandran	Rotamer	Cβ deviation	Bond lengths	Bond angles	Cis Peptides
			Avg: 0.69	Clashscore: 1.84	Outliers: 9 of 654	Poor rotamers: 9 of 581	Outliers: 13 of 633	Outliers: 1 of 656	Outliers: 66 of 656	Non-Trans: 2 of 655
A 31		ASN	0.56	0.61Å N with A 31 ASN HD22	-	OUTLIER (0%) chi angles: 58,153.1	0.07Å	-	-	-
A 32		TYR	0.65	-	Favored (52.15%) General / -125.6,130.7	Favored (78.7%) *m-80* chi angles: 288.5,93	0.15Å	-	-	-
A 33		SER	0.71	-	Favored (22.7%) General / -77.5,120.6	Favored (2%) *m* chi angles: 315.7	0.20Å	-	-	-
A 34		ILE	0.66	-	Favored (8.56%) Ile or Val / -111.3,10.3	Favored (22.1%) *pt* chi angles: 53.6,173.8	0.19Å	-	-	-

A 35		ASP	0.72	-	Favored (2.08%) General / -52.5,155.6	Favored (7.9%) *m-30* chi angles: 313.3,313.2	0.12Å	-	-	-
A 36		ILE	0.7	-	Favored (25.61%) Ile or Val / -50.4,-48.1	Favored (98.4%) *mt* chi angles: 293,168.1	0.08Å	-	-	-
A 37		LEU	0.76	0.67Å HD13 with A 505 ALA HB1	Favored (89.55%) General / -66.0,-38.9	Favored (7.6%) *mt* chi angles: 295.6,152.7	0.13Å	-	-	-
A 38		ARG	0.71	-	Favored (83.73%) General / -66.9,-43.3	Favored (25%) *mmt-90* chi angles: 298.6,274.5,199,286	0.07Å	-	-	-
A 39		LEU	0.76	-	Favored (96.4%) General / -61.8,-44.7	Favored (90.3%) *mt* chi angles: 291.6,169.6	0.05Å	-	-	-
A 40		ALA	0.79	-	Favored (96.04%) General / -60.7,-44.8	-	0.06Å	-	-	-
A 41		LYS	0.76	-	Favored (88.59%) General / -65.4,-44.0	Favored (18.1%) *tppt* chi angles: 184.4,58.4,71.5,172.9	0.03Å	-	-	-
A 42		ALA	0.8	-	Favored (96.6%) General / -62.2,-40.6	-	0.03Å	-	-	-
A 43		ALA	0.8	-	Favored (97.79%) General / -61.1,-44.1	-	0.01Å	-	-	-
A 44		HIS	0.76	-	Favored (71.54%) General / -60.3,-51.0	Favored (61.2%) *m170* chi angles: 291.7,168.8	0.09Å	-	OUTLIER(S) worst is ND1-CG-CD2: 4.5 σ	-
A 45		ILE	0.77	-	Favored (90.03%) Ile or Val / -59.9,-47.8	Favored (41.7%) *mt* chi angles: 285.3,172.1	0.09Å	-	-	-
A 46		LYS	0.76	-	Favored (89.76%) General / -65.4,-38.3	Favored (28.7%) *tptt* chi angles: 187,61.3,194.1,175.7	0.06Å	-	-	-
A 47		ALA	0.77	-	Favored (60.95%) General / -71.8,-11.7	-	0.04Å	-	-	-
A 48		TYR	0.71	-	Favored (10.82%) General /	Favored (14.9%) *m-10* chi angles: 298.7,348.2	0.08Å	-	-	-

#	Alt	Res	High B	Clash > 0.4Å	Ramachandran	Rotamer	Cβ deviation	Bond lengths	Bond angles	Cis Peptides
					-101.9,-31.2					
A 49		MET	0.75	-	Favored (19.79%) General / -89.2,154.9	Favored (6.7%) *ptm* chi angles: 66.9,204.2,296.9	0.11Å	-	-	-
A 50		GLY	0.76	-	Favored (2.49%) Glycine / -110.2,40.6	-	-	-	-	-
**#**	**Alt**	**Res**	**High B**	**Clash > 0.4Å**	**Ramachandran**	**Rotamer**	**Cβ deviation**	**Bond lengths**	**Bond angles**	**Cis Peptides**
			Avg: 0.69	Clashscore: 1.84	Outliers: 9 of 654	Poor rotamers: 9 of 581	Outliers: 13 of 633	Outliers: 1 of 656	Outliers: 66 of 656	Non-Trans: 2 of 655
A 51		ASP	0.73	-	Favored (82.28%) General / -58.9,-39.7	Favored (17.1%) *p0* chi angles: 53.6,342.6	0.11Å	-	OUTLIER(S) worst is CA-CB-CG: 5.0 σ	-
A 52		GLU	0.53	-	Favored (7.8%) General / -65.9,-4.9	Favored (50%) *mt-10* chi angles: 308.3,178.2,352.7	0.05Å	-	-	-
A 53		LYS	0.68	-	Allowed (1%) General / -135.9,-29.9	Favored (96.3%) *mttt* chi angles: 297.4,183.2,178.3,179.5	0.06Å	-	-	-
A 54		GLU	0.69	-	Favored (48.18%) General / -113.8,140.6	Favored (11.7%) *mm-30* chi angles: 294.6,277.7,305.3	0.10Å	-	-	-
A 55		GLN	0.7	-	Favored (34.24%) General / -83.0,128.2	Favored (53%) *tt0* chi angles: 177.1,181.3,341	0.03Å	-	-	-
A 56		ALA	0.78	-	Favored (63.88%) General / -53.1,-40.2	-	0.05Å	-	-	-
A 57		CYS	0.79	-	Favored (59.37%) General / -78.2,-14.7	Favored (51.3%) *m* chi angles: 303.3	0.03Å	-	-	-
A 58		ASN	0.73	-	Favored (24.92%) General / -82.9,-36.9	Favored (43.4%) *m-40* chi angles: 292.8,1.6	0.07Å	-	-	-
A 59		ASN	0.74	-	Allowed (0.42%) General / -168.5,100.7	Favored (32.8%) *t0* chi angles: 195.8,341.6	0.04Å	-	-	-
A 60		PHE	0.73	-	Favored (60.59%) General / -73.9,-26.6	Favored (17.1%) *t80* chi angles: 198.4,71.4	0.10Å	-	-	-

A 61		TYR	0.7	-	Favored (75.37%) General / -62.1,-49.6	Favored (54.8%) *t80* chi angles: 186.8,68.7	0.07Å	-	-	-
A 62		ASN	0.7	-	Favored (90.72%) General / -64.6,-44.4	Favored (11.2%) *m-40* chi angles: 296,10.3	0.11Å	-	-	-
A 63		TYR	0.7	-	Favored (74.71%) General / -58.0,-50.1	Favored (60.7%) *t80* chi angles: 173.2,87.7	0.07Å	-	-	-
A 64		ALA	0.77	-	Favored (78.8%) General / -69.2,-38.7	-	0.04Å	-	-	-
A 65		CYS	0.78	-	Favored (13.37%) General / -108.0,-19.9	Favored (72.5%) *m* chi angles: 297.7	0.12Å	-	-	-
A 66		ALA	0.73	-	Favored (12.64%) General / -62.4,-10.9	-	0.18Å	-	-	-
A 67		ASN	0.71	-	Favored (72.32%) General / -70.9,-40.2	Allowed (0.7%) *t0* chi angles: 230,4.6	0.08Å	-	-	-
A 68		TRP	0.66	-	Favored (85.47%) Pre-Pro / -62.9,-48.2	Favored (46.5%) *t60* chi angles: 192,99	0.17Å	-	OUTLIER(S) worst is CA-C-N: 6.4 σ	-
A 69		PRO	0.66	-	Favored (85.73%) Trans-Pro / -59.1,-27.7	Favored (88.6%) *Cg_exo* chi angles: 330.8,36.9,328.1	0.05Å	-	-	-
A 70		ARG	0.66	-	Favored (72.35%) General / -70.6,-41.4	Favored (67.9%) *mtm-85* chi angles: 293.5,190,302.6,253.6	0.07Å	-	-	-
**#**	**Alt**	**Res**	**High B**	**Clash > 0.4Å**	**Ramachandran**	**Rotamer**	**Cβ deviation**	**Bond lengths**	**Bond angles**	**Cis Peptides**
			Avg: 0.69	Clashscore: 1.84	Outliers: 9 of 654	Poor rotamers: 9 of 581	Outliers: 13 of 633	Outliers: 1 of 656	Outliers: 66 of 656	Non-Trans: 2 of 655
A 71		LEU	0.65	-	Favored (24.48%) General / -87.6,-22.9	Favored (95.6%) *mt* chi angles: 295.1,172.9	0.05Å	-	-	-
A 72		HIS	0.66	-	Favored (41.5%) Pre-Pro / -117.2,110.3	Favored (18.8%) *m90* chi angles: 274.8,82.4	0.12Å	-	OUTLIER(S) worst is ND1-CG-CD2: 4.4 σ	-

A 73		PRO	0.65	-	Favored (29.51%) Trans-Pro / -77.1,150.5	Favored (44.5%) *Cg_endo* chi angles: 33.6,325.8,21.6	0.08Å	-	-	-
A 74		ALA	0.7	-	Favored (57.32%) General / -65.8,137.5	-	0.11Å	-	-	-
A 75		ARG	0.66	0.52Å H with A 81 THR HG21	Favored (33.71%) General / -87.6,124.6	Favored (81.3%) *ttt180* chi angles: 186.3,180.8,178.4,184.2	0.03Å	-	-	-
A 76		LYS	0.57	-	Favored (3.93%) General / -63.6,-5.5	Favored (98.1%) *mttt* chi angles: 296.1,180.2,176.2,178.7	0.03Å	-	-	-
A 77		SER	0.52	-	Favored (57.3%) General / -86.8,-8.5	Favored (8.3%) *p* chi angles: 49.7	0.09Å	-	-	-
A 78		LYS	0.52	-	Favored (32.87%) General / -123.5,157.7	Favored (16.4%) *mmtp* chi angles: 290.9,300.1,192.9,62.8	0.06Å	-	-	-
A 79		THR	0.67	-	Favored (36.42%) General / -103.5,4.8	Favored (77.9%) *p* chi angles: 60.2	0.17Å	-	-	-
A 80		ARG	0.58	0.43Å HB3 with A 639 TYR CE1	Favored (19.91%) General / -160.8,152.8	Favored (5.2%) *ptp-170* chi angles: 53.8,214.4,70.2,180.1	0.08Å	-	-	-
A 81		THR	0.68	0.52Å HG21 with A 75 ARG H	Favored (20.79%) General / -146.1,133.5	Favored (26.7%) *p* chi angles: 52.2	0.09Å	-	-	-
A 82		ASN	0.67	0.54Å HB3 with A 637 THR HB	OUTLIER (0.01)%) General / 161.8,167.0	Favored (3.4%) *p0* chi angles: 55.7,92.2	0.24Å	-	OUTLIER(S) worst is C-CA-CB: 5.1 σ	-
A 83		TYR	0.66	-	Favored (10.83%) General / -45.0,-50.2	Favored (18.3%) *m-10* chi angles: 305,140.7	0.14Å	-	-	-
A 84		LEU	0.67	-	Favored (95.54%) General / -64.2,-43.3	Favored (25.6%) *tp* chi angles: 189.1,60.8	0.09Å	-	-	-
A 85		GLU	0.68	-	Favored (96.15%) General / -64.4,-42.4	Favored (4.9%) *tp30* chi angles: 184,84.7,38.1	0.04Å	-	-	-
A 86		GLU	0.69	-	Favored (92.18%) General /	Favored (52.8%) *mt-10* chi angles:	0.08Å	-	-	-

#	Alt	Res	High B	Clash > 0.4Å	Ramachandran	Rotamer	Cβ deviation	Bond lengths	Bond angles	Cis Peptides
					-59.7,-42.0	287.7,163.4,24.6				
A 87		LEU	0.73	-	Favored (88.48%) General / -58.5,-42.8	Favored (54%) *mt* chi angles: 288.4,176.9	0.10Å	-	-	-
A 88		GLN	0.72	-	Favored (99.2%) General / -61.5,-43.2	Favored (79.2%) *mt0* chi angles: 298.1,181.8,350.6	0.13Å	-	-	-
A 89		GLU	0.73	-	Favored (94.14%) General / -59.8,-43.9	Favored (19.4%) *mt-10* chi angles: 293.6,204.5,6.4	0.09Å	-	-	-
A 90		LEU	0.76	-	Favored (89.66%) General / -63.7,-38.0	Favored (94.4%) *mt* chi angles: 296.2,173.4	0.10Å	-	-	-
**#**	**Alt**	**Res**	**High B**	**Clash > 0.4Å**	**Ramachandran**	**Rotamer**	**Cβ deviation**	**Bond lengths**	**Bond angles**	**Cis Peptides**
			Avg: 0.69	Clashscore: 1.84	Outliers: 9 of 654	Poor rotamers: 9 of 581	Outliers: 13 of 633	Outliers: 1 of 656	Outliers: 66 of 656	Non-Trans: 2 of 655
A 91		TYR	0.75	-	Favored (84.12%) General / -57.7,-46.7	Favored (19.7%) *t80* chi angles: 175.5,100.2	0.04Å	-	-	-
A 92		ILE	0.78	-	Favored (78.45%) Ile or Val / -60.9,-38.2	Favored (29.6%) *mm* chi angles: 297.1,292.4	0.10Å	-	-	-
A 93		ARG	0.76	-	Favored (86.96%) General / -58.3,-46.0	Favored (97.8%) *mtt180* chi angles: 296.6,178.4,182.7,173.9	0.04Å	-	-	-
A 94		LYS	0.76	-	Favored (96.43%) General / -61.2,-41.2	Favored (65.3%) *mmtt* chi angles: 288.9,292.7,178.5,176	0.07Å	-	-	-
A 95		SER	0.81	-	Favored (88.64%) General / -66.6,-40.0	Favored (39.2%) *m* chi angles: 289.7	0.05Å	-	-	-
A 96		ALA	0.81	-	Favored (94.06%) General / -59.9,-44.6	-	0.02Å	-	-	-
A 97		ASP	0.78	-	Favored (79.43%) General / -56.3,-47.1	Favored (64.5%) *m-30* chi angles: 299.5,337.7	0.12Å	-	-	-
A 98		MET	0.77	-	Favored (61.26%) General / -74.9,-30.2	Favored (86.9%) *mmm* chi angles: 289,303.9,288.2	0.06Å	-	-	-

A 99		LEU	0.77	-	Favored (25.69%) General / -86.9,-22.6	Favored (85.8%) *mt* chi angles: 291.6,175.3	0.07Å	-	-	-
A 100		LYS	0.74	-	Favored (57.36%) General / -75.3,-23.1	Favored (83.5%) *mttt* chi angles: 285.4,168.8,182.2,184.2	0.06Å	-	-	-
A 101		SER	0.76	-	Allowed (1.06%) General / -96.6,46.2	Favored (52.6%) *p* chi angles: 56.8	0.08Å	-	-	-
A 102		ALA	0.57	-	Favored (64.27%) General / -57.1,-30.4	-	0.09Å	-	-	-
A 103		THR	0.44	-	Favored (28.98%) General / -97.2,13.5	Favored (16.4%) *p* chi angles: 48.9	0.10Å	-	-	-
A 104		ARG	0.52	-	Favored (51.49%) General / -59.6,131.6	Favored (70.7%) *ttt180* chi angles: 186.1,186.7,185.6,174.6	0.02Å	-	-	-
A 105		GLY	0.52	-	Favored (61.86%) Glycine / -76.5,1.3	-	-	-	-	-
A 106		ALA	0.53	-	Favored (23.02%) General / -107.5,0.8	-	0.08Å	-	-	-
A 107		GLU	0.59	-	Favored (50.01%) General / -65.9,133.0	Favored (42.8%) *mm-30* chi angles: 304.2,299.3,300.9	0.09Å	-	-	-
A 108		ASN	0.67	-	Favored (16.84%) General / -77.0,170.3	Favored (10.1%) *p0* chi angles: 69.7,63.9	0.19Å	-	-	-
A 109		SER	0.75	-	Favored (71.31%) General / -60.1,-32.8	Favored (61.1%) *m* chi angles: 298.7	0.11Å	-	-	-
A 110		ALA	0.76	-	Favored (77.34%) General / -67.3,-45.2	-	0.03Å	-	-	-
**#**	**Alt**	**Res**	**High B**	**Clash > 0.4Å**	**Ramachandran**	**Rotamer**	**Cβ deviation**	**Bond lengths**	**Bond angles**	**Cis Peptides**
			Avg: 0.69	Clashscore: 1.84	Outliers: 9 of 654	Poor rotamers: 9 of 581	Outliers: 13 of 633	Outliers: 1 of 656	Outliers: 66 of 656	Non-Trans: 2 of 655
					Favored	Favored (74.3%) *m-*				

A 111		ASP	0.75	-	(95.28%) General / -60.1,-44.0	*30* chi angles: 297.7,323.4	0.04Å	-	-	-
A 112		ARG	0.69	-	Favored (84.36%) General / -57.6,-46.1	Favored (84.4%) *mtp180* chi angles: 293.1,172.1,63.6,196.3	0.07Å	-	-	-
A 113		GLN	0.75	-	Favored (99.61%) General / -62.2,-43.1	Favored (85.6%) *mm-40* chi angles: 288.8,302.7,315.2	0.07Å	-	-	-
A 114		LEU	0.78	0.42Å HD13 with A 361 PHE HB2	Favored (86.3%) General / -58.0,-43.0	Favored (56.6%) *tp* chi angles: 173.8,61.1	0.15Å	-	-	-
A 115		LYS	0.76	-	Favored (29.58%) General / -84.7,-22.9	Favored (3.2%) *mtpt* chi angles: 307.4,159.5,108.3,180.3	0.15Å	-	-	-
A 116		TYR	0.74	-	Favored (85.48%) General / -66.1,-37.3	Favored (14.8%) *m-10* chi angles: 293.2,354.2	0.09Å	-	-	-
A 117		PHE	0.76	-	Favored (97.22%) General / -60.9,-44.4	Favored (49.3%) *t80* chi angles: 189.7,72.9	0.11Å	-	-	-
A 118		TYR	0.76	-	Favored (98.51%) General / -61.6,-42.2	Favored (74.1%) *t80* chi angles: 181.5,70.2	0.09Å	-	-	-
A 119		GLY	0.78	-	Favored (12.02%) Glycine / -51.2,-57.1	-	-	-	-	-
A 120		SER	0.76	-	Favored (94.78%) General / -60.6,-45.2	Favored (81.5%) *p* chi angles: 61.9	0.16Å	-	-	-
A 121		CYS	0.79	-	Favored (75.38%) General / -58.1,-37.9	Favored (49.4%) *t* chi angles: 184.7	0.06Å	-	-	-
A 122		ARG	0.68	-	Favored (55.49%) General / -93.2,-2.1	Favored (10.1%) *ptm-80* chi angles: 75.9,185,305.5,284.3	0.03Å	-	-	-
A 123		LEU	0.7	-	Favored (7.21%) General / -81.9,65.9	Favored (83.4%) *mt* chi angles: 289.5,172.1	0.10Å	-	-	-
A 124		GLN	0.66	-	Favored (56.32%) General / -53.4,-34.1	Favored (85.3%) *mt0* chi angles: 294.6,179.3,296.5	0.05Å	-	-	-

#	Alt	Res	High B	Clash > 0.4Å	Ramachandran	Rotamer	Cβ deviation	Bond lengths	Bond angles	Cis Peptides
A 125		THR	0.67	-	Favored (74.95%) General / -58.2,-37.6	Favored (33.7%) *m* chi angles: 306.2	0.04Å	-	-	-
A 126		ASN	0.66	-	Favored (65.46%) General / -70.6,-45.3	Favored (95.8%) *m-40* chi angles: 290.9,344	0.03Å	-	-	-
A 127		ASP	0.66	-	Favored (96.02%) General / -61.7,-44.9	Favored (93.7%) *m-30* chi angles: 285.6,346.7	0.04Å	-	-	-
A 128		THR	0.67	-	Favored (79.36%) General / -60.6,-36.8	Favored (37.3%) *p* chi angles: 54.3	0.16Å	-	-	-
A 129		ARG	0.66	-	Favored (64.43%) General / -68.7,-21.9	Favored (96.3%) *mtt180* chi angles: 288.8,172.7,180.9,180.1	0.05Å	-	-	-
A 130		SER	0.69	-	Favored (51.54%) General / -86.7,-11.3	Favored (27.1%) *p* chi angles: 77.9	0.11Å	-	-	-
**#**	**Alt**	**Res**	**High B**	**Clash > 0.4Å**	**Ramachandran**	**Rotamer**	**Cβ deviation**	**Bond lengths**	**Bond angles**	**Cis Peptides**
			Avg: 0.69	Clashscore: 1.84	Outliers: 9 of 654	Poor rotamers: 9 of 581	Outliers: 13 of 633	Outliers: 1 of 656	Outliers: 66 of 656	Non-Trans: 2 of 655
A 131		ALA	0.67	-	Allowed (1.6%) General / 52.2,-120.1	-	0.17Å	-	-	-
A 132		LEU	0.67	0.43Å HD21 with A 342 ARG HH21	Favored (17.58%) General / -114.0,10.6	OUTLIER (0.3%) chi angles: 270.2,273.6	0.08Å	-	-	-
A 133		ASN	0.69	-	Favored (86.28%) General / -66.3,-37.8	Favored (41.9%) *m-40* chi angles: 300.1,348.1	0.03Å	-	-	-
A 134		THR	0.72	-	Favored (97.25%) General / -60.6,-43.5	Favored (21.3%) *p* chi angles: 51	0.04Å	-	-	-
A 135		LEU	0.7	-	Favored (76.5%) General / -65.5,-47.3	OUTLIER (0%) chi angles: 214.8,205	0.03Å	-	-	-
A 136		GLN	0.67	-	Favored (91.71%) General / -59.1,-43.0	Favored (63.6%) *tt0* chi angles: 184.9,180.3,42.8	0.03Å	-	-	-

A 137		ASN	0.7	-	Favored (74.84%) General / -60.5,-35.0	Favored (93.9%) *m-40* chi angles: 291.4,329.9	0.03Å	-	-	-
A 138		VAL	0.76	-	Favored (14.12%) Ile or Val / -77.5,-19.0	Favored (50.8%) *t* chi angles: 169.5	0.07Å	-	-	-
A 139		THR	0.74	-	Favored (80.58%) General / -60.5,-48.7	Favored (16.1%) *m* chi angles: 289.7	0.09Å	-	-	-
A 140		ASP	0.75	0.46Å OD1 with A 149 ARG NH1	Favored (71.88%) General / -62.2,-31.4	Favored (19.3%) *m-30* chi angles: 281,316.5	0.12Å	-	-	-
A 141		PHE	0.66	-	Favored (59.58%) General / -67.4,-12.2	Favored (75%) *m-80* chi angles: 288.6,99	0.07Å	-	OUTLIER(S) worst is CA-CB-CG: 4.2 σ	-
A 142		ARG	0.69	-	Favored (22.73%) General / -110.7,10.9	Favored (37.7%) *mtm180* chi angles: 291.1,184.1,271.4,180.9	0.09Å	-	-	-
A 143		GLY	0.74	-	Favored (85.65%) Glycine / 85.9,3.4	-	-	-	-	-
A 144		GLY	0.77	-	Favored (3.25%) Glycine / 61.8,179.0	-	-	-	-	-
A 145		TRP	0.68	0.42Å HB3 with A 148 ILE HG13	Favored (52.27%) Pre-Pro / -112.4,111.6	Favored (15.1%) *t60* chi angles: 189.1,27.9	0.07Å	-	OUTLIER(S) worst is CA-C-N: 4.2 σ	-
A 146		PRO	0.7	-	Favored (83.06%) Trans-Pro / -58.9,-27.1	Favored (42.9%) *Cg_exo* chi angles: 338.2,11.5,3.1	0.09Å	-	-	-
A 147		GLU	0.61	-	Favored (60.25%) General / -75.9,-13.1	Favored (52.8%) *mt-10* chi angles: 282.6,169.2,25.4	0.14Å	-	-	-
A 148		ILE	0.53	0.42Å HG13 with A 145 TRP HB3	Favored (7.27%) Ile or Val / -117.8,16.1	Allowed (0.5%) *pp* chi angles: 60.5,65	0.10Å	-	OUTLIER(S) worst is CA-CB-CG1: 4.4 σ	-
A 149		ARG	0.56	0.46Å NH1 with A 140 ASP OD1	Favored (56.07%) General / -111.7,128.4	Favored (63.3%) *mmm-85* chi angles: 292.2,290.1,295.4,276.6	0.10Å	-	-	-
A 150		VAL	0.52	-	Favored (22.6%) Ile or Val / -110.4,142.5	Favored (3.2%) *p* chi angles: 55.2	0.07Å	-	-	-

#	Alt	Res	High B	Clash > 0.4Å	Ramachandran	Rotamer	Cβ deviation	Bond lengths	Bond angles	Cis Peptides
			Avg: 0.69	Clashscore: 1.84	Outliers: 9 of 654	Poor rotamers: 9 of 581	Outliers: 13 of 633	Outliers: 1 of 656	Outliers: 66 of 656	Non-Trans: 2 of 655
A 151		ALA	0.58	-	Favored (24.57%) General / -98.7,-9.4	-	0.08Å	-	-	Cis nonPRO omega= -4.9
A 152		SER	0.54	-	Favored (2.91%) General / -146.7,97.4	Favored (63.9%) *m* chi angles: 294.1	0.12Å	-	-	-
A 153		TRP	0.56	-	Favored (91%) General / -61.6,-39.4	Favored (64.5%) *t60* chi angles: 178,75.9	0.10Å	-	OUTLIER(S) worst is CG-CD2-CE3: 4.9 σ	-
A 154		TYR	0.55	-	OUTLIER (0.04)%) General / -63.8,11.6	Favored (58.2%) *p90* chi angles: 63,90.3	0.09Å	-	-	-
A 155		GLN	0.51	-	Favored (60.77%) General / -74.7,-14.7	Favored (19%) *pt0* chi angles: 64.1,185.3,33.3	0.03Å	-	-	-
A 156		TYR	0.45	-	Favored (44.67%) General / -129.9,156.3	Favored (37.9%) *m-80* chi angles: 288.3,120.5	0.08Å	-	-	-
A 157		GLU	0.54	-	Favored (49.71%) General / -106.5,124.5	Favored (5%) *mt-10* chi angles: 291.3,214.2,13.8	0.04Å	-	-	-
A 158		TYR	0.65	-	Favored (16.9%) General / -119.7,110.5	Favored (49.9%) *m-80* chi angles: 302.8,118	0.05Å	-	-	-
A 159		ASP	0.67	-	Favored (58.64%) General / -62.9,141.5	Favored (90.4%) *m-30* chi angles: 291.1,349.7	0.09Å	-	-	-
A 160		TRP	0.65	-	Favored (73.46%) General / -59.8,-34.8	Favored (28.5%) *m-10* chi angles: 307.8,340.9	0.04Å	-	-	-
A 161		LEU	0.7	-	Favored (65.27%) General / -54.0,-51.0	Favored (58.7%) *mt* chi angles: 303.5,173.5	0.14Å	-	-	-
A 162		GLN	0.7	-	Favored (63.43%) General / -72.2,-44.0	Favored (94.2%) *mt0* chi angles: 295.1,177.8,336.1	0.03Å	-	-	-
					Favored					

A 163		VAL	0.74	-	(80.91%) Ile or Val / -59.9,-50.2	Favored (81.4%) *t* chi angles: 176.6	0.03Å	-	-	-
A 164		VAL	0.75	-	Favored (82.66%) Ile or Val / -68.6,-41.1	Favored (48.6%) *t* chi angles: 181.7	0.12Å	-	-	-
A 165		ALA	0.78	-	Favored (98.49%) General / -62.5,-43.6	-	0.02Å	-	-	-
A 166		ASN	0.74	-	Favored (87.58%) General / -59.9,-47.0	Favored (84.6%) *m-40* chi angles: 284.1,334.4	0.09Å	-	-	-
A 167		LEU	0.76	-	Favored (89.76%) General / -65.2,-43.9	Favored (3.4%) *mt* chi angles: 275,152.7	0.07Å	-	-	-
A 168		LYS	0.77	-	Favored (48.43%) General / -65.1,-53.2	Favored (7%) *ttmm* chi angles: 177.3,161.2,282,293	0.17Å	-	-	-
A 169		ARG	0.72	-	Favored (71.57%) General / -71.3,-36.4	Favored (2.5%) *tmm160* chi angles: 186.7,240.2,294.8,160.8	0.03Å	-	-	-
A 170		LYS	0.75	-	Favored (22.06%) General / -96.2,-15.4	Favored (4.5%) *tttt* chi angles: 212.4,191.6,202.5,202.7	0.02Å	-	-	-
**#**	**Alt**	**Res**	**High B**	**Clash > 0.4Å**	**Ramachandran**	**Rotamer**	**Cβ deviation**	**Bond lengths**	**Bond angles**	**Cis Peptides**
			Avg: 0.69	Clashscore: 1.84	Outliers: 9 of 654	Poor rotamers: 9 of 581	Outliers: 13 of 633	Outliers: 1 of 656	Outliers: 66 of 656	Non-Trans: 2 of 655
A 171		LEU	0.75	-	Allowed (0.9%) General / -143.6,-3.6	Favored (72%) *mt* chi angles: 295.7,167.8	0.06Å	-	-	-
A 172		GLY	0.81	-	Favored (78.96%) Glycine / 72.3,22.3	-	-	-	-	-
A 173		VAL	0.79	-	Favored (53.41%) Ile or Val / -102.7,121.1	Favored (8.7%) *t* chi angles: 190.3	0.07Å	-	-	-
A 174		ASP	0.77	-	Favored (26.65%) General / -106.6,113.2	Favored (11.7%) *m-30* chi angles: 282.9,18.5	0.11Å	-	-	-
A					Favored (61.1%)	Allowed (0.6%) *mp*				

175	ILE	0.76	-	Ile or Val / -130.7,135.1	chi angles: 318.2,90.1	0.06Å	-	-	-
A 176	PHE	0.76	-	Allowed (0.48%) General / 76.6,-60.0	Favored (56.6%) *m-80* chi angles: 285.3,103	0.25Å	-	-	-
A 177	ILE	0.75	-	Favored (19.23%) Ile or Val / -132.1,112.0	Favored (32.1%) *mt* chi angles: 303,158.5	0.04Å	-	-	-
A 178	GLY	0.8	-	Favored (2.55%) Glycine / -85.4,73.8	-	-	-	-	-
A 179	LEU	0.78	0.48Å HD21 with A 193 LEU HD21	Favored (19.44%) General / -75.9,119.1	Allowed (0.7%) *tm* chi angles: 182.5,292.5	0.21Å	-	OUTLIER(S) worst is N-CA-CB: 5.0 σ	-
A 180	GLU	0.76	-	Favored (37.64%) General / -139.2,140.3	Favored (8.3%) *pt0* chi angles: 64.3,165.5,16.7	0.10Å	-	OUTLIER(S) worst is CG-CD-OE1: 4.2 σ	-
A 181	VAL	0.76	-	Favored (42.3%) Ile or Val / -89.3,125.8	Favored (69.7%) *t* chi angles: 172.1	0.05Å	-	-	-
A 182	ILE	0.69	-	Allowed (0.64%) Ile or Val / -155.2,171.5	Favored (18.1%) *tt* chi angles: 194.1,164.6	0.10Å	-	-	-
A 183	LEU	0.7	-	Favored (58%) General / -61.0,139.7	Favored (28.6%) *mt* chi angles: 305.4,169.2	0.08Å	-	-	-
A 184	ASP	0.73	-	Favored (29.19%) General / -69.9,126.3	Favored (44.1%) *t0* chi angles: 185.4,329.5	0.10Å	-	OUTLIER(S) worst is CA-CB-CG: 6.3 σ	-
A 185	TYR	0.66	-	Favored (69.41%) General / -64.7,-28.1	Favored (7.5%) *m-10* chi angles: 284.3,145.1	0.03Å	-	-	-
A 186	LYS	0.67	-	Favored (21.58%) General / -111.4,13.1	Favored (88.6%) *mttt* chi angles: 288.2,183.9,170,178.9	0.04Å	-	-	-
A 187	GLU	0.68	-	Allowed (1.12%) General / -155.5,88.1	Favored (5.9%) *tp30* chi angles: 174.2,52.2,90.2	0.07Å	-	-	-
A 188	GLU	0.7	-	Favored (65.46%) General / -59.9,-25.8	Allowed (1.6%) *pp20* chi angles: 69,95.5,1.9	0.11Å	-	OUTLIER(S) worst is CB-CG-CD: 4.5 σ	-

A 189		LYS	0.69	-	Favored (5.12%) General / -91.3,20.5	Favored (91.6%) *mttt* chi angles: 300.9,184.1,178,180.6	0.11Å	-	-	-
A 190		MET	0.7	-	Favored (41.15%) General / -139.3,147.1	Favored (94.9%) *mmm* chi angles: 292.5,296.6,294.3	0.09Å	-	-	-
**#**	**Alt**	**Res**	**High B**	**Clash > 0.4Å**	**Ramachandran**	**Rotamer**	**Cβ deviation**	**Bond lengths**	**Bond angles**	**Cis Peptides**
			Avg: 0.69	Clashscore: 1.84	Outliers: 9 of 654	Poor rotamers: 9 of 581	Outliers: 13 of 633	Outliers: 1 of 656	Outliers: 66 of 656	Non-Trans: 2 of 655
A 191		HIS	0.71	0.46Å CE1 with A 470 PRO HG3	Favored (26.99%) General / -82.0,151.7	Favored (78.5%) *m90* chi angles: 295.4,81	0.09Å	-	-	-
A 192		ARG	0.74	-	Favored (14.81%) General / -140.6,171.0	Favored (71.7%) *mmt-90* chi angles: 282.9,294.1,180.6,277.9	0.10Å	-	-	-
A 193		LEU	0.79	0.54Å HD21 with A 291 LEU HD22	Favored (31.72%) General / -95.9,139.7	Favored (49.8%) *tp* chi angles: 181.4,56.3	0.10Å	-	-	-
A 194		LYS	0.78	-	Favored (46.11%) General / -118.9,144.9	Favored (11.9%) *ttmt* chi angles: 185,193.8,292.5,159.4	0.04Å	-	-	-
A 195		ILE	0.77	-	Favored (55.34%) Ile or Val / -103.7,129.7	Favored (2.9%) *mm* chi angles: 284.4,280.2	0.09Å	-	-	-
A 196		GLY	0.77	-	Favored (19.52%) Glycine / -128.3,170.9	-	-	-	-	-
A 197		ALA	0.77	-	Favored (25.61%) Pre-Pro / -91.5,141.2	-	0.04Å	-	-	-
A 198		PRO	0.76	-	Favored (34.57%) Trans-Pro / -76.6,153.5	Favored (23.5%) *Cg_endo* chi angles: 36.4,322.1,24.8	0.05Å	-	-	-
A 199		GLN	0.61	-	Favored (50.88%) General / -65.0,148.8	Favored (99.7%) *mm-40* chi angles: 298.8,299.7,309.3	0.03Å	-	-	-
A 200		PHE	0.71	-	Favored (53.32%) Pre-Pro / -86.8,158.6	Favored (25%) *m-80* chi angles: 313.2,112.7	0.11Å	-	-	-

A 201		PRO	0.76	-	Favored (3.59%) Trans-Pro / -63.9,-49.9	Favored (55.4%) *Cg_exo* chi angles: 328.1,43.1,321.6	0.06Å	-	-	-
A 202		MET	0.74	-	Favored (56.16%) General / -67.9,142.2	Favored (67.5%) *mmm* chi angles: 304.4,313.1,291.1	0.11Å	-	-	-
A 203		SER	0.68	-	Favored (41.69%) General / -52.3,-33.3	Favored (26.9%) *m* chi angles: 303.6	0.15Å	-	-	-
A 204		ARG	0.66	-	Favored (32.9%) General / -160.0,164.9	Favored (30.2%) *ptt180* chi angles: 53.4,180.8,181.2,191	0.05Å	-	-	-
A 205		ARG	0.65	-	Favored (57.85%) General / -63.2,-15.7	Favored (9%) *ptm-80* chi angles: 67.6,191.5,299.5,261.5	0.01Å	-	-	-
A 206		HIS	0.68	-	Favored (69.23%) General / -60.3,-30.1	Favored (57.2%) *m170* chi angles: 296.4,163.8	0.13Å	-	-	-
A 207		TYR	0.67	-	Favored (62.27%) General / -69.7,-13.4	Favored (21.8%) *m-80* chi angles: 276.4,72.5	0.05Å	-	-	-
A 208		LEU	0.72	-	Favored (48.58%) General / -95.6,6.6	Allowed (1.6%) *mm* chi angles: 285,293.7	0.06Å	-	-	-
A 209		HIS	0.64	-	Favored (10.6%) Pre-Pro / -104.5,167.0	Favored (94.4%) *m-70* chi angles: 293.1,284	0.12Å	-	OUTLIER(S) worst is ND1-CG-CD2: 4.2 σ	-
A 210		PRO	0.48	-	Favored (2.15%) Trans-Pro / -84.6,15.6	Favored (54%) *Cg_endo* chi angles: 25.7,322.1,35.6	0.03Å	-	-	-
**#**	**Alt**	**Res**	**High B**	**Clash > 0.4Å**	**Ramachandran**	**Rotamer**	**Cβ deviation**	**Bond lengths**	**Bond angles**	**Cis Peptides**
			Avg: 0.69	Clashscore: 1.84	Outliers: 9 of 654	Poor rotamers: 9 of 581	Outliers: 13 of 633	Outliers: 1 of 656	Outliers: 66 of 656	Non-Trans: 2 of 655
A 211		HIS	0.45	-	Favored (55.81%) General / -90.8,2.7	Favored (99.2%) *m-70* chi angles: 299.1,286.6	0.06Å	-	OUTLIER(S) worst is ND1-CG-CD2: 4.0 σ	-
A 212		PHE	0.58	-	Favored (2.76%) General / -135.6,41.2	Favored (84.9%) *m-80* chi angles: 289.8,89.4	0.03Å	-	-	-
					Favored	Favored (29.7%)				

A 213		GLU	0.65	-	(80.08%)<br>General /<br>-58.0,-48.4	*tp30*<br>chi angles:<br>182.6,57.8,2.1	0.10Å	-	-	-
A 214		GLY	0.76	-	Favored<br>(73.38%)<br>Glycine /<br>-68.9,-32.1	-	-	-	-	-
A 215		THR	0.74	-	Favored<br>(80.92%)<br>General /<br>-68.6,-38.8	Favored (15.3%) *p*<br>chi angles: 48.3	0.20Å	-	-	-
A 216		ARG	0.71	-	Favored<br>(92.38%)<br>General /<br>-63.2,-45.3	Favored (57.9%)<br>*ttp80*<br>chi angles:<br>184.8,203.2,67.6,76.7	0.05Å	-	-	-
A 217		GLU	0.75	-	Favored<br>(88.43%)<br>General /<br>-61.7,-46.7	Favored (75.6%) *tt0*<br>chi angles:<br>184.6,186,7.5	0.07Å	-	-	-
A 218		ILE	0.75	-	Favored<br>(83.39%)<br>Ile or Val /<br>-57.4,-43.9	Favored (69.3%) *mt*<br>chi angles: 289,164	0.07Å	-	-	-
A 219		TYR	0.77	-	Favored<br>(79.17%)<br>General /<br>-58.4,-48.9	Favored (23.4%) *t80*<br>chi angles: 182.2,50	0.10Å	-	-	-
A 220		GLU	0.75	-	Favored<br>(85.12%)<br>General /<br>-67.0,-38.0	Favored (15.4%) *tt0*<br>chi angles:<br>198.3,158.7,17.5	0.10Å	-	-	-
A 221		ARG	0.73	-	Favored<br>(99.07%)<br>General /<br>-61.7,-42.5	Favored (91.2%)<br>*mtt-85*<br>chi angles:<br>292.4,188.2,185.2,285.4	0.10Å	-	-	-
A 222		SER	0.77	-	Favored<br>(78.24%)<br>General /<br>-61.0,-36.0	Favored (61.9%) *m*<br>chi angles: 293.8	0.13Å	-	-	-
A 223		ILE	0.71	-	Favored<br>(74.7%)<br>Ile or Val /<br>-60.1,-51.3	Favored (92.9%) *mt*<br>chi angles: 296,171.2	0.03Å	-	-	-
A 224		GLU	0.67	-	Favored<br>(76.72%)<br>General /<br>-64.5,-48.0	Favored (48.3%) *tt0*<br>chi angles:<br>184.3,179.4,59.9	0.06Å	-	-	-
A 225		ASN	0.69	-	Favored<br>(24.22%)<br>General /<br>-145.8,136.4	Allowed (2%) *p0*<br>chi angles: 60.5,102.8	0.14Å	-	-	-
A 226		LYS	0.52	-	Favored<br>(50.78%)<br>General /<br>-83.9,-14.6	Favored (93.3%)<br>*mttt*<br>chi angles:<br>286,185.8,179,179.9	0.08Å	-	-	Cis<br>nonPRO<br>omega=<br>5.42

A 227		LEU	0.64	-	Favored (50.63%) General / -66.4,-52.0	Favored (8.9%) *mt* chi angles: 286.6,152.5	0.23Å	-	-	-
A 228		LYS	0.64	-	Favored (62.3%) General / -73.7,-29.5	Favored (16.3%) *ttpt* chi angles: 183.8,194.9,71.7,191.6	0.10Å	-	-	-
A 229		LEU	0.56	-	Favored (18.31%) General / -83.0,-41.8	Favored (81.2%) *mt* chi angles: 289,168.1	0.25Å	-	-	-
A 230		TYR	0.48	-	Favored (81.43%) General / -67.7,-42.9	Allowed (1.7%) *t80* chi angles: 201.2,119.2	0.14Å	-	-	-
**#**	**Alt**	**Res**	**High B**	**Clash > 0.4Å**	**Ramachandran**	**Rotamer**	**Cβ deviation**	**Bond lengths**	**Bond angles**	**Cis Peptides**
			Avg: 0.69	Clashscore: 1.84	Outliers: 9 of 654	Poor rotamers: 9 of 581	Outliers: 13 of 633	Outliers: 1 of 656	Outliers: 66 of 656	Non-Trans: 2 of 655
A 231		PHE	0.54	-	Favored (74.3%) Pre-Pro / -61.1,-52.1	Allowed (0.3%) *t80* chi angles: 188.7,141	0.21Å	-	OUTLIER(S) worst is CA-C-N: 5.6 σ	-
A 232		PRO	0.44	-	OUTLIER (0.02)%) Trans-Pro / -90.4,-48.8	Favored (31.1%) *Cg_endo* chi angles: 21.5,328.4,30.2	0.08Å	-	-	-
A 233		GLU	0.4	-	Favored (79.76%) General / -67.7,-43.7	Favored (59%) *tt0* chi angles: 192.1,187.4,357	0.09Å	-	-	-
A 234		GLN	0.45	-	Favored (15.32%) General / -105.1,-14.7	Favored (28.6%) *mm-40* chi angles: 314,285.5,309	0.07Å	-	-	-
A 235		SER	0.51	-	Favored (6.05%) General / -126.9,-10.6	Favored (94.4%) *p* chi angles: 66.2	0.20Å	-	-	-
A 236		GLU	0.49	-	Favored (13.67%) General / -111.0,-15.7	Favored (37.4%) *tt0* chi angles: 189.3,183.2,62.3	0.07Å	-	-	-
A 237		HIS	0.47	-	Favored (3.2%) General / 67.6,37.9	Favored (87.7%) *m-70* chi angles: 306.1,283.3	0.15Å	-	OUTLIER(S) worst is ND1-CG-CD2: 4.2 σ	-
A 238		TRP	0.45	-	Favored (12.11%) General / -88.4,169.1	Favored (21.1%) *t60* chi angles: 183.9,30.2	0.08Å	-	-	-
					Favored	Favored (28.5%)				

A 239		ARG	0.41	-	(30.27%) General / -75.0,160.7	*ptt90* chi angles: 62.7,158.5,180.3,94.4	0.08Å	-	-	-
A 240		GLN	0.39	-	Favored (53.91%) General / -58.3,140.2	Favored (84.6%) *mt0* chi angles: 299.2,177.3,343.7	0.05Å	-	-	-
A 241		GLU	0.32	-	Favored (31.85%) General / -145.2,146.7	Favored (7.2%) *tp30* chi angles: 186.2,52.2,92.6	0.07Å	-	-	-
A 242		VAL	0.35	-	Favored (2.8%) Ile or Val / -95.9,160.5	Favored (46.1%) *t* chi angles: 182.3	0.14Å	-	-	-
A 243		ALA	0.39	-	Favored (43.37%) General / -151.0,157.8	-	0.09Å	-	-	-
A 244		SER	0.43	-	Favored (11.79%) General / -162.7,175.0	Favored (97.7%) *p* chi angles: 65.2	0.06Å	-	-	-
A 245		GLN	0.63	-	Allowed (0.2%) General / 86.4,10.8	Favored (91.2%) *mm-40* chi angles: 297.2,291.7,305.8	0.30Å	-	-	-
A 246		VAL	0.74	-	Favored (14.75%) Ile or Val / -90.2,-47.3	Favored (38.8%) *t* chi angles: 183.7	0.09Å	-	-	-
A 247		VAL	0.76	-	Favored (97.99%) Ile or Val / -61.2,-44.2	Favored (82.2%) *t* chi angles: 176.4	0.07Å	-	-	-
A 248		GLN	0.7	-	Favored (95.3%) General / -62.1,-40.3	Favored (74.9%) *mt0* chi angles: 291.7,185.9,358.3	0.11Å	-	-	-
A 249		ILE	0.73	0.42Å HD11 with A 325 GLN HB3	Favored (84.76%) Ile or Val / -57.5,-47.6	Favored (92.5%) *mt* chi angles: 297,171.7	0.11Å	-	-	-
A 250		GLU	0.74	-	Favored (74.4%) General / -69.1,-34.0	Favored (7.3%) *tt0* chi angles: 204.4,167.1,29.5	0.19Å	-	-	-
**#**	**Alt**	**Res**	**High B**	**Clash > 0.4Å**	**Ramachandran**	**Rotamer**	**Cβ deviation**	**Bond lengths**	**Bond angles**	**Cis Peptides**
			Avg: 0.69	Clashscore: 1.84	Outliers: 9 of 654	Poor rotamers: 9 of 581	Outliers: 13 of 633	Outliers: 1 of 656	Outliers: 66 of 656	Non-Trans: 2 of 655
A		GLN	0.72	-	Favored (69.34%)	Favored (72.1%) *mt0*	0.07Å	-	-	-

251					General / -57.6,-34.5	chi angles: 291,179.5,285.7				
A 252		GLN	0.72	-	Favored (72.71%) General / -68.2,-45.7	Favored (78%) *mt0* chi angles: 300.1,172.1,301.9	0.06Å	-	-	-
A 253		LEU	0.75	-	Favored (84.31%) General / -60.8,-38.1	Favored (94.4%) *mt* chi angles: 294.9,174.6	0.07Å	-	-	-
A 254		ALA	0.77	-	Favored (63.24%) General / -66.2,-15.5	-	0.08Å	-	-	-
A 255		LYS	0.69	-	Allowed (1.06%) General / -89.3,29.8	Favored (97.9%) *mttt* chi angles: 292.9,184.6,182.5,179.5	0.21Å	-	-	-
A 256		GLY	0.63	-	Allowed (1.16%) Glycine / -150.8,120.9	-	-	-	-	-
A 257		LEU	0.64	-	OUTLIER (0)%) Pre-Pro / 107.6,78.2	OUTLIER (0%) chi angles: 175.5,192.5	2.33Å	-	OUTLIER(S) worst is CA-C-N: 6.5 σ	-
A 258		PRO	0.65	-	Favored (30.57%) Trans-Pro / -75.4,165.5	Favored (7.2%) *Cg_exo* chi angles: 349,27,327.4	0.04Å	-	-	-
A 259		HIS	0.57	-	Favored (99.4%) General / -62.9,-41.4	Favored (98.2%) *m-70* chi angles: 296.4,289.8	0.10Å	-	OUTLIER(S) worst is ND1-CG-CD2: 4.2 σ	-
A 260		ASN	0.61	-	Favored (95.88%) Pre-Pro / -52.6,-44.8	Favored (77.1%) *m-40* chi angles: 302.8,310.3	0.15Å	-	OUTLIER(S) worst is CA-C-N: 6.2 σ	-
A 261		PRO	0.6	-	Favored (20.14%) Trans-Pro / -70.7,-6.9	Favored (97.6%) *Cg_exo* chi angles: 332.7,41.1,320.2	0.06Å	-	-	-
A 262		ALA	0.66	-	Favored (39.97%) General / -97.2,-4.0	-	0.07Å	-	-	-
A 263		LEU	0.63	-	Favored (65.31%) General / -71.7,-30.1	Favored (50.8%) *mt* chi angles: 299.9,168.2	0.04Å	-	-	-
A 264		THR	0.67	-	Favored (7.73%) General / -83.5,92.9	Favored (70.4%) *m* chi angles: 296	0.13Å	-	-	-
					Favored					

A 265		LEU	0.64	-	(64.05%) General / -59.8,-24.4	Favored (4%) *mt* chi angles: 281.2,192.9	0.10Å	-	-	-
A 266		ALA	0.68	-	Favored (94.59%) General / -65.2,-40.7	-	0.03Å	-	-	-
A 267		GLN	0.66	-	Favored (74.37%) General / -70.2,-41.0	Favored (96.8%) *mt0* chi angles: 291.8,171.4,318.3	0.06Å	-	-	-
A 268		THR	0.64	-	Favored (81.99%) General / -62.7,-36.2	Favored (65.3%) *p* chi angles: 63.1	0.17Å	-	-	-
A 269		THR	0.71	-	Allowed (1.96%) General / -67.6,105.8	Allowed (0.9%) *m* chi angles: 280.8	0.12Å	-	-	-
A 270		ARG	0.69	-	Favored (12.85%) General / -125.5,109.5	Favored (26%) *mmm160* chi angles: 306.5,290.6,286.7,185.8	0.06Å	-	OUTLIER(S) worst is CG-CD-NE: 4.2 σ	-
**#**	**Alt**	**Res**	**High B**	**Clash > 0.4Å**	**Ramachandran**	**Rotamer**	**Cβ deviation**	**Bond lengths**	**Bond angles**	**Cis Peptides**
			Avg: 0.69	Clashscore: 1.84	Outliers: 9 of 654	Poor rotamers: 9 of 581	Outliers: 13 of 633	Outliers: 1 of 656	Outliers: 66 of 656	Non-Trans: 2 of 655
A 271		GLN	0.76	-	Favored (40.4%) General / -93.9,129.3	Favored (62.2%) *tt0* chi angles: 182.8,181.8,8	0.02Å	-	-	-
A 272		ARG	0.77	-	Favored (29.49%) General / -137.7,164.2	Favored (52.1%) *mmt180* chi angles: 295.2,297.8,184.4,177.2	0.15Å	-	-	-
A 273		THR	0.8	-	Favored (16.1%) General / -90.2,160.6	Favored (49.9%) *p* chi angles: 65.7	0.12Å	-	-	-
A 274		ALA	0.8	-	Favored (64.43%) General / -56.9,-31.1	-	0.04Å	-	-	-
A 275		ALA	0.78	-	Favored (92.02%) General / -63.4,-45.2	-	0.03Å	-	-	-
A 276		GLU	0.73	-	Favored (96.19%) General / -64.4,-42.2	Favored (35.6%) *mt-10* chi angles: 291.9,174.2,269.4	0.09Å	-	OUTLIER(S) worst is CB-CG-CD: 4.8 σ	-
A					Favored (84.98%)	Favored (31.7%) *ttt*				

277		MET	0.72	-	General / -59.0,-40.4	chi angles: 184.9,177.2,174.1	0.04Å	-	-	-
A 278		LYS	0.7	-	Favored (97.71%) General / -61.3,-44.1	Favored (50.3%) *mtmt* chi angles: 288.6,180.7,289.3,189.2	0.04Å	-	-	-
A 279		THR	0.69	-	Favored (80.41%) General / -64.7,-46.9	Favored (99.6%) *m* chi angles: 300.5	0.06Å	-	-	-
A 280		ALA	0.69	-	Favored (21.75%) General / -79.8,-43.8	-	0.06Å	-	-	-
A 281		TYR	0.65	-	Favored (11.41%) General / -116.0,24.5	Favored (67.6%) *m-80* chi angles: 300.2,112.2	0.06Å	-	-	-
A 282		GLY	0.67	-	Favored (10.33%) Glycine / -49.5,122.3	-	-	-	-	-
A 283		SER	0.58	-	Allowed (0.88%) General / -107.2,-68.6	Favored (14.5%) *t* chi angles: 168.4	0.08Å	-	OUTLIER(S) worst is C-N-CA: 4.5 σ	-
A 284		TYR	0.42	-	OUTLIER (0)%) General / 170.5,37.0	Favored (94.5%) *m-80* chi angles: 293.1,86.6	2.42Å	-	-	-
A 285		VAL	0.63	-	Favored (56.96%) Ile or Val / -126.3,137.7	Favored (26.7%) *m* chi angles: 299.7	0.04Å	-	-	-
A 286		ASP	0.75	-	Favored (19.84%) General / -80.4,115.3	Favored (19.5%) *t0* chi angles: 179.3,35.6	0.10Å	-	-	-
A 287		VAL	0.75	-	Favored (82.12%) Ile or Val / -60.8,-39.6	Favored (83.3%) *t* chi angles: 173.4	0.09Å	-	-	-
A 288		THR	0.76	-	Favored (87.45%) General / -61.1,-47.2	Favored (25.6%) *m* chi angles: 291.7	0.10Å	-	-	-
A 289		ARG	0.7	-	Favored (95.16%) General / -60.4,-45.0	Favored (40.6%) *tpt170* chi angles: 186.5,65.1,172.2,174.8	0.02Å	-	-	-
A 290		TYR	0.75	-	Favored (82.34%) General / -56.9,-45.9	Favored (26.4%) *t80* chi angles: 174.6,54.6	0.12Å	-	-	-

#	Alt	Res	High B	Clash > 0.4Å	Ramachandran	Rotamer	Cβ deviation	Bond lengths	Bond angles	Cis Peptides
			Avg: 0.69	Clashscore: 1.84	Outliers: 9 of 654	Poor rotamers: 9 of 581	Outliers: 13 of 633	Outliers: 1 of 656	Outliers: 66 of 656	Non-Trans: 2 of 655
A 291		LEU	0.76	0.54Å HD22 with A 193 LEU HD21	Favored (84.3%) General / -63.4,-46.9	Favored (94%) *mt* chi angles: 296.4,172.6	0.07Å	-	-	-
A 292		GLN	0.71	-	Favored (60.02%) General / -76.1,-12.0	Favored (17.2%) *mm-40* chi angles: 299.1,286.6,29.1	0.06Å	-	-	-
A 293		LEU	0.72	-	Favored (59.13%) General / -75.5,-28.7	Favored (38.6%) *mt* chi angles: 292.4,161.4	0.01Å	-	-	-
A 294		ILE	0.75	-	Favored (17.28%) Ile or Val / -85.3,-45.9	Favored (14.8%) *mt* chi angles: 295.7,151.5	0.06Å	-	-	-
A 295		PHE	0.73	-	Favored (57.85%) General / -66.7,140.0	Favored (77.2%) *m-80* chi angles: 293.2,107.3	0.14Å	-	OUTLIER(S) worst is CA-CB-CG: 7.4 σ	-
A 296		ASN	0.69	-	Favored (4.99%) General / -82.8,-54.6	Favored (84.4%) *m-40* chi angles: 297.1,330.4	0.09Å	-	-	-
A 297		ASP	0.58	-	Favored (9.27%) General / -88.6,-46.2	Favored (7.9%) *m-30* chi angles: 308.5,340.5	0.06Å	-	-	-
A 298		ASN	0.67	-	Allowed (0.21%) General / 177.3,152.0	Favored (25.1%) *t0* chi angles: 200.7,10.4	0.10Å	-	-	-
A 299		LEU	0.62	-	Favored (27.91%) General / -87.3,142.4	Favored (21.2%) *tp* chi angles: 167.9,66.1	0.08Å	-	-	-
A 300		TYR	0.57	-	Favored (44.7%) General / -97.8,130.9	Favored (51.9%) *t80* chi angles: 189.8,77.3	0.04Å	-	-	-
A 301		MET	0.78	-	Favored (68.47%) General / -66.6,-28.4	Favored (92.2%) *mtp* chi angles: 290.4,182.2,69	0.12Å	-	-	-
A 302		ASP	0.79	-	Favored (9.77%) General / -69.6,116.2	Favored (17.5%) *m-30* chi angles: 298.5,0.8	0.10Å	-	-	-

A 303		LEU	0.79	0.47Å HD22 with A 193 LEU HD23	Favored (53.26%) General / -120.6,139.2	Favored (32.7%) *mt* chi angles: 307.1,184.8	0.14Å	-	-	-
A 304		TYR	0.78	-	Favored (19.35%) General / -103.3,108.2	Favored (15.6%) *m-80* chi angles: 276.1,67	0.06Å	-	-	-
A 305		GLU	0.77	-	Favored (10.04%) General / -93.3,96.6	Favored (24.5%) *mt-10* chi angles: 298.2,195.2,248.9	0.04Å	-	-	-
A 306		THR	0.73	-	Favored (88.97%) Pre-Pro / -61.3,-43.2	Favored (48%) *m* chi angles: 294.4	0.17Å	-	OUTLIER(S) worst is CA-C-N: 4.9 σ	-
A 307		PRO	0.7	-	OUTLIER (0)%) Trans-Pro / -120.0,69.7	OUTLIER (0%) chi angles: 45.4,321,17.4	0.14Å	-	-	-
A 308		GLU	0.65	-	Favored (52.78%) General / -50.1,-44.9	Favored (28.9%) *mt-10* chi angles: 288,193.7,299.5	0.06Å	-	-	-
A 309		ASP	0.66	-	Favored (83.78%) General / -57.4,-43.3	Favored (62.9%) *m-30* chi angles: 298.1,347.3	0.08Å	-	-	-
A 310		TYR	0.68	-	Favored (93.67%) General / -64.5,-43.5	Favored (57.5%) *t80* chi angles: 168.3,80.9	0.05Å	-	-	-
**#**	**Alt**	**Res**	**High B**	**Clash > 0.4Å**	**Ramachandran**	**Rotamer**	**Cβ deviation**	**Bond lengths**	**Bond angles**	**Cis Peptides**
			Avg: 0.69	Clashscore: 1.84	Outliers: 9 of 654	Poor rotamers: 9 of 581	Outliers: 13 of 633	Outliers: 1 of 656	Outliers: 66 of 656	Non-Trans: 2 of 655
A 311		MET	0.69	-	Favored (96.2%) General / -60.3,-42.7	Favored (96.7%) *mmm* chi angles: 292.5,301.7,295.4	0.05Å	-	-	-
A 312		SER	0.71	-	Favored (91.67%) General / -59.7,-45.6	Favored (62.3%) *m* chi angles: 298.3	0.08Å	-	-	-
A 313		ASN	0.67	-	Favored (71.13%) General / -66.8,-30.8	Favored (97.3%) *m-40* chi angles: 291.6,335.1	0.03Å	-	-	-
A 314		LEU	0.67	-	Favored (71.8%) General / -61.0,-32.2	Favored (9.6%) *tp* chi angles: 199.4,64.5	0.02Å	-	-	-
					Favored					

A 315		VAL	0.63	-	(79.82%) Ile or Val / -56.5,-48.7	Favored (65.5%) *t* chi angles: 179.3	0.03Å	-	-	-
A 316		ASP	0.62	-	Favored (69.07%) General / -71.5,-41.5	Favored (77.6%) *m-30* chi angles: 290.9,327.8	0.15Å	-	-	-
A 317		VAL	0.67	-	Favored (87.72%) Ile or Val / -58.8,-43.3	Favored (83.7%) *t* chi angles: 177	0.03Å	-	-	-
A 318		ILE	0.64	-	Favored (13.56%) Ile or Val / -78.1,-12.1	Favored (13.1%) *tt* chi angles: 199,169.8	0.13Å	-	-	-
A 319		ARG	0.56	-	Favored (57.37%) General / -81.0,-14.9	Favored (96.1%) *mtt180* chi angles: 291.2,170.4,179.2,177.7	0.04Å	-	-	-
A 320		GLU	0.63	-	Favored (51.57%) General / -83.8,-14.4	Favored (93.1%) *mt-10* chi angles: 293.4,180.9,337.7	0.03Å	-	-	-
A 321		THR	0.69	-	Favored (27.58%) Pre-Pro / -115.7,126.6	Favored (16.2%) *p* chi angles: 48.7	0.07Å	-	-	-
A 322		PRO	0.72	-	Favored (10.88%) Trans-Pro / -61.3,166.6	Favored (82.1%) *Cg_exo* chi angles: 334.5,32.8,332.3	0.07Å	-	-	-
A 323		LYS	0.69	-	Favored (65.59%) General / -54.2,-38.3	Favored (97.1%) *mttt* chi angles: 297.2,176.3,176.5,175.9	0.06Å	-	-	-
A 324		LEU	0.7	-	Favored (49.16%) General / -56.8,-54.2	Favored (7.4%) *tt* chi angles: 184.6,146.7	0.10Å	-	-	-
A 325		GLN	0.69	0.42Å HB3 with A 249 ILE HD11	Favored (95.74%) General / -64.7,-41.9	Favored (27.1%) *mm-40* chi angles: 293.6,296.1,6.5	0.07Å	-	-	-
A 326		LEU	0.75	-	Favored (95.6%) General / -62.4,-44.8	Favored (59.5%) *mt* chi angles: 296.4,166.5	0.03Å	-	-	-
A 327		ALA	0.77	-	Favored (81.04%) General / -61.9,-36.3	-	0.06Å	-	-	-
A 328		ASN	0.75	-	Favored (80.64%) General / -58.3,-48.4	Favored (11%) *m110* chi angles: 282.9,117.8	0.11Å	-	OUTLIER(S) worst is CA-CB-CG: 4.6 σ	-

#	Alt	Res	High B	Clash > 0.4Å	Ramachandran	Rotamer	Cβ deviation	Bond lengths	Bond angles	Cis Peptides
A 329		TYR	0.76	-	Favored (76.25%) General / -66.2,-46.7	Favored (9.6%) *t80* chi angles: 169.2,48.4	0.09Å	-	-	-
A 330		THR	0.76	-	Favored (91.12%) General / -60.5,-46.2	Favored (5.4%) *m* chi angles: 286.9	0.03Å	-	-	-
**#**	**Alt**	**Res**	**High B**	**Clash > 0.4Å**	**Ramachandran**	**Rotamer**	**Cβ deviation**	**Bond lengths**	**Bond angles**	**Cis Peptides**
			Avg: 0.69	Clashscore: 1.84	Outliers: 9 of 654	Poor rotamers: 9 of 581	Outliers: 13 of 633	Outliers: 1 of 656	Outliers: 66 of 656	Non-Trans: 2 of 655
A 331		MET	0.76	-	Favored (74.96%) General / -70.1,-40.3	Favored (9%) *tpt* chi angles: 189.1,55.5,169.7	0.07Å	-	OUTLIER(S) worst is CG-SD-CE: 4.4 σ	-
A 332		TRP	0.76	-	Favored (66.69%) General / -52.7,-48.1	Favored (13.9%) *t-100* chi angles: 158.6,255.1	0.04Å	-	OUTLIER(S) worst is CG-CD2-CE3: 4.6 σ	-
A 333		LYS	0.75	-	Favored (72.08%) General / -60.1,-33.4	Favored (50%) *tttp* chi angles: 184.8,168.8,173.6,64.5	0.07Å	-	-	-
A 334		ALA	0.77	-	Favored (58.77%) General / -76.4,-33.8	-	0.06Å	-	-	-
A 335		LEU	0.74	-	Favored (92.02%) General / -65.3,-39.2	Favored (52.2%) *mt* chi angles: 294.7,183.4	0.06Å	-	-	-
A 336		GLU	0.67	-	Favored (31.3%) General / -50.9,-35.1	Favored (30%) *tt0* chi angles: 180.5,199.2,359	0.15Å	-	-	-
A 337		ALA	0.67	-	Favored (54.38%) General / -93.5,-2.4	-	0.11Å	-	-	-
A 338		LEU	0.66	-	Favored (16.08%) General / -111.0,-2.1	Favored (85.3%) *mt* chi angles: 294.2,169.2	0.08Å	-	-	-
A 339		ASP	0.64	-	Favored (58.41%) General / -80.3,-14.4	Allowed (1.9%) *p0* chi angles: 84,332.2	0.23Å	-	-	-
A 340		ILE	0.56	-	Favored (8.29%) Ile or Val / -91.9,2.2	Favored (34.4%) *pt* chi angles: 57.3,174.6	0.08Å	-	-	-

A 341		ALA	0.69	-	Favored (14.45%) General / -117.3,18.4	-	0.04Å	-	-	-
A 342		ARG	0.62	0.43Å HH21 with A 132 LEU HD21	Favored (5.94%) General / -127.1,-8.8	Favored (14.2%) *ptt90* chi angles: 70.4,197.6,168.6,105.9	0.10Å	-	-	-
A 343		VAL	0.54	-	Favored (31.68%) Pre-Pro / -156.3,158.9	Favored (2.1%) *m* chi angles: 285	0.09Å	-	-	-
A 344		PRO	0.55	-	Favored (23.78%) Trans-Pro / -48.1,133.8	Favored (95.2%) *Cg_exo* chi angles: 332.9,37.3,326.6	0.06Å	-	-	-
A 345		ALA	0.53	-	Favored (10.08%) General / -83.3,175.2	-	0.04Å	-	-	-
A 346		SER	0.46	-	Favored (60.83%) General / -74.6,-15.5	Favored (18.3%) *p* chi angles: 52.6	0.07Å	-	-	-
A 347		GLN	0.51	-	Favored (9.39%) General / -94.2,170.6	Favored (80%) *mt0* chi angles: 298.7,183.8,334.4	0.04Å	-	-	-
A 348		ARG	0.66	-	Favored (3.72%) General / -61.8,115.1	Favored (24.2%) *tpt170* chi angles: 183.9,70.1,175.5,191	0.07Å	-	-	-
A 349		ALA	0.72	-	Favored (90.68%) General / -58.8,-43.3	-	0.07Å	-	-	-
A 350		ASP	0.69	-	Favored (67.93%) General / -53.4,-43.3	Favored (92.2%) *m-30* chi angles: 285.4,349.7	0.12Å	-	OUTLIER(S) worst is CA-CB-CG: 4.7 σ	-
**#**	**Alt**	**Res**	**High B**	**Clash > 0.4Å**	**Ramachandran**	**Rotamer**	**Cβ deviation**	**Bond lengths**	**Bond angles**	**Cis Peptides**
			Avg: 0.69	Clashscore: 1.84	Outliers: 9 of 654	Poor rotamers: 9 of 581	Outliers: 13 of 633	Outliers: 1 of 656	Outliers: 66 of 656	Non-Trans: 2 of 655
A 351		ILE	0.73	-	Favored (92.7%) Ile or Val / -64.2,-46.7	Favored (23.7%) *mt* chi angles: 293.2,184.2	0.06Å	-	-	-
A 352		TRP	0.73	-	Favored (85.42%) General / -64.0,-46.2	Favored (38.4%) *t-100* chi angles: 185.5,238.6	0.07Å	-	-	-

A 353		CYS	0.81	-	Favored (90.81%) General / -61.5,-39.4	Favored (83.9%) *m* chi angles: 295.3	0.04Å	-	-	-
A 354		VAL	0.81	-	Favored (81.7%) Ile or Val / -58.8,-49.8	Favored (4%) *t* chi angles: 160.3	0.06Å	-	-	-
A 355		GLN	0.77	-	Favored (96.03%) General / -64.6,-41.9	Favored (80.9%) *tp40* chi angles: 185.8,62,63.9	0.05Å	-	-	-
A 356		LEU	0.8	-	Favored (95.69%) General / -60.8,-45.1	Favored (94.7%) *mt* chi angles: 291.9,173.1	0.07Å	-	-	-
A 357		ALA	0.81	-	Favored (92.93%) General / -60.1,-41.5	-	0.09Å	-	-	-
A 358		GLN	0.78	-	Favored (97.52%) General / -60.8,-43.9	Favored (48.2%) *tt0* chi angles: 195.1,177.8,352.8	0.10Å	-	-	-
A 359		GLN	0.77	-	Favored (80.41%) General / -65.5,-35.3	Favored (55.3%) *tp40* chi angles: 189.5,59.1,70.8	0.07Å	-	-	-
A 360		PHE	0.79	-	Favored (15.28%) General / -101.2,-20.4	Favored (21.3%) *m-10* chi angles: 303.7,325	0.05Å	-	-	-
A 361		PHE	0.8	0.42Å HB2 with A 114 LEU HD13	Favored (60.15%) Pre-Pro / -131.4,61.6	Favored (67.1%) *m-80* chi angles: 304.2,89.7	0.14Å	-	OUTLIER(S) worst is CA-C-N: 4.5 σ	-
A 362		PRO	0.78	-	Favored (23.27%) Trans-Pro / -60.8,-44.8	Favored (83.7%) *Cg_exo* chi angles: 334.3,36,326.9	0.09Å	-	-	-
A 363		HIS	0.74	-	Favored (93.34%) General / -62.3,-45.4	Favored (56.3%) *m170* chi angles: 295.1,161.4	0.06Å	-	-	-
A 364		GLN	0.73	-	Favored (91.36%) General / -59.8,-45.8	Favored (16.3%) *mp10* chi angles: 298.2,79.3,356	0.04Å	-	-	-
A 365		LEU	0.77	-	Favored (60.78%) General / -74.9,-28.8	Favored (3.9%) *tp* chi angles: 193.9,42.1	0.13Å	-	-	-
A		GLU	0.74	-	Favored (89.94%)	Favored (90.5%) *tt0* chi angles:	0.03Å	-	-	-

#	Alt	Res	High B	Clash > 0.4Å	Ramachandran	Rotamer	Cβ deviation	Bond lengths	Bond angles	Cis Peptides
366					General / -58.6,-44.6	185.3,175.8,359.1				
A 367		SER	0.76	0.49Å HA with A 370 HIS CD2	Favored (98.95%) General / -63.3,-43.1	Favored (10.5%) *m* chi angles: 308.2	0.18Å	-	-	-
A 368		LEU	0.77	-	Favored (86.07%) General / -65.0,-45.3	Favored (88.7%) *mt* chi angles: 297.3,171.9	0.03Å	-	-	-
A 369		PHE	0.75	-	Favored (96.25%) General / -63.4,-43.9	Favored (34.6%) *t80* chi angles: 178.4,55.9	0.06Å	-	-	-
A 370		HIS	0.74	0.49Å CD2 with A 367 SER HA	Favored (98.2%) General / -62.0,-41.5	Favored (12.9%) *p90* chi angles: 70.1,68.2	0.29Å	-	OUTLIER(S) worst is CA-CB-CG: 9.1 σ	-
**#**	**Alt**	**Res**	**High B**	**Clash > 0.4Å**	**Ramachandran**	**Rotamer**	**Cβ deviation**	**Bond lengths**	**Bond angles**	**Cis Peptides**
			Avg: 0.69	Clashscore: 1.84	Outliers: 9 of 654	Poor rotamers: 9 of 581	Outliers: 13 of 633	Outliers: 1 of 656	Outliers: 66 of 656	Non-Trans: 2 of 655
A 371		ARG	0.68	-	Favored (70.45%) General / -61.6,-30.0	Favored (93.6%) *mtt180* chi angles: 296.4,168.3,183.8,176.6	0.04Å	-	-	-
A 372		ASN	0.76	-	Favored (26.98%) General / -104.0,-1.8	Favored (60.5%) *t0* chi angles: 190.2,42.7	0.13Å	-	-	-
A 373		TYR	0.71	-	Allowed (0.14%) General / -153.5,-17.0	Favored (20.6%) *t80* chi angles: 189.4,53.7	0.04Å	-	-	-
A 374		ASN	0.7	-	Favored (45.15%) General / -98.6,129.8	Favored (15%) *t0* chi angles: 189.2,255.2	0.17Å	-	-	-
A 375		HIS	0.58	0.47Å CE1 with A 497 GLN HE22	Favored (70.94%) General / -63.4,-29.7	Favored (58.4%) *m90* chi angles: 291.1,70.8	0.17Å	-	OUTLIER(S) worst is ND1-CG-CD2: 4.2 σ	-
A 376		MET	0.54	-	Allowed (0.54%) General / 76.5,-47.0	Favored (31.9%) *mtp* chi angles: 306.3,173.8,53.8	0.25Å	-	-	-
A 377		GLN	0.62	-	Favored (68.81%) General / -62.1,-27.3	Favored (88.8%) *mt0* chi angles: 299.1,177.9,334.3	0.04Å	-	-	-
A 378		MET	0.65	-	Favored (58.48%) General /	Favored (10.1%) *ptm* chi angles:	0.04Å	-	-	-

#	Alt	Res	High B	Clash > 0.4Å	Ramachandran	Rotamer	Cβ deviation	Bond lengths	Bond angles	Cis Peptides
					-75.0,-9.4	70,198.6,298.9				
A 379		ILE	0.65	-	Favored (76.65%) Ile or Val / -59.2,-38.8	Favored (4%) *pt* chi angles: 69.5,188.2	0.10Å	-	-	-
A 380		ASN	0.73	-	Favored (99.86%) General / -62.5,-43.0	Favored (60.3%) *m-40* chi angles: 300.3,338.7	0.07Å	-	-	-
A 381		GLU	0.75	-	Favored (96.68%) General / -62.6,-44.3	Favored (5.1%) *mm-30* chi angles: 289.8,272.1,292.1	0.09Å	-	-	-
A 382		LEU	0.78	-	Favored (93.96%) General / -61.9,-40.0	Favored (35.5%) *tp* chi angles: 180.1,53.8	0.03Å	-	-	-
A 383		GLN	0.76	-	Favored (88.98%) General / -58.9,-42.1	Favored (51.8%) *tt0* chi angles: 185.3,186.6,7.2	0.08Å	-	-	-
A 384		SER	0.8	-	Favored (89.17%) General / -61.3,-46.7	Favored (70.8%) *m* chi angles: 296.2	0.08Å	-	-	-
A 385		THR	0.79	-	Favored (90.58%) General / -59.9,-41.0	Favored (50.6%) *m* chi angles: 304.2	0.03Å	-	-	-
A 386		TRP	0.8	0.43Å CD1 with A 489 ILE HD11	Favored (86.17%) General / -58.3,-46.4	Favored (35.2%) *t60* chi angles: 170.1,69.7	0.09Å	OUTLIER(S) worst is CD2--CE2: 5.9 σ	OUTLIER(S) worst is CG-CD2-CE3: 6.2 σ	-
A 387		SER	0.81	-	Favored (93.19%) General / -60.1,-41.6	Favored (18.9%) *m* chi angles: 286.9	0.09Å	-	-	-
A 388		ASP	0.8	-	Favored (75.17%) General / -64.2,-48.6	Favored (92.4%) *m-30* chi angles: 289.1,350.9	0.02Å	-	-	-
A 389		ILE	0.82	-	Favored (81.42%) Ile or Val / -63.8,-38.1	Favored (8.4%) *mt* chi angles: 285.9,151.7	0.22Å	-	-	-
A 390		LYS	0.79	-	Favored (99.78%) General / -62.8,-42.6	Favored (28.8%) *ttpt* chi angles: 189.7,177.1,69.9,164.1	0.05Å	-	-	-
**#**	**Alt**	**Res**	**High B**	**Clash > 0.4Å**	**Ramachandran**	**Rotamer**	**Cβ deviation**	**Bond lengths**	**Bond angles**	**Cis Peptides**
			Avg: 0.69	Clashscore: 1.84	Outliers: 9 of 654	Poor rotamers: 9 of 581	Outliers: 13 of 633	Outliers: 1 of 656	Outliers: 66 of 656	Non-Trans: 2 of 655

A 391		ARG	0.78	-	Favored (91.79%) General / -61.7,-45.9	Favored (29.1%) *ttm110* chi angles: 186.7,195.7,302.5,107.7	0.04Å	-	-	-
A 392		VAL	0.81	-	Favored (95.34%) Ile or Val / -60.1,-45.8	Favored (87.5%) *t* chi angles: 173.8	0.09Å	-	-	-
A 393		PHE	0.79	-	Favored (82.78%) General / -60.3,-38.1	Favored (77.1%) *t80* chi angles: 183.8,83.8	0.10Å	-	-	-
A 394		ARG	0.76	-	Favored (99.34%) General / -62.3,-42.4	Favored (50.6%) *ttt-90* chi angles: 197.6,182.9,176.8,275.6	0.09Å	-	-	-
A 395		GLU	0.76	-	Favored (73.4%) General / -70.5,-40.5	Favored (81.1%) *mt-10* chi angles: 298.4,172.6,14.5	0.20Å	-	OUTLIER(S) worst is C-CA-CB: 4.1 σ	-
A 396		ASP	0.76	-	Favored (72.01%) General / -61.6,-31.9	Favored (9.6%) *m-30* chi angles: 280.1,299.3	0.18Å	-	OUTLIER(S) worst is CA-CB-CG: 4.5 σ	-
A 397		LEU	0.75	-	Favored (73.61%) General / -56.2,-40.2	Favored (39.4%) *mt* chi angles: 295.8,163.5	0.17Å	-	OUTLIER(S) worst is CA-C-N: 4.1 σ	-
A 398		GLN	0.64	-	Favored (61.76%) General / -67.6,-13.1	Favored (90.7%) *mt0* chi angles: 286.9,180.4,324.8	0.27Å	-	-	-
A 399		ALA	0.62	-	Allowed (0.43%) General / -154.8,16.4	-	0.02Å	-	-	-
A 400		SER	0.67	-	Favored (16.88%) General / -76.1,170.1	Favored (78.4%) *p* chi angles: 60.7	0.14Å	-	-	-
A 401		GLU	0.59	-	Favored (68.93%) General / -57.0,-35.2	Favored (47.3%) *tp30* chi angles: 178.1,72,3	0.08Å	-	-	-
A 402		ARG	0.59	-	Favored (74.88%) General / -56.6,-49.5	Allowed (1.4%) *tpm170* chi angles: 177,70.8,282.1,133.4	0.17Å	-	-	-
A 403		LEU	0.48	-	Favored (82.33%) General / -68.1,-38.6	Favored (4.9%) *tt* chi angles: 186.3,160	0.27Å	-	OUTLIER(S) worst is N-CA-CB: 4.2 σ	-
A 404		LEU	0.53	-	Favored (32.43%) General /	Favored (59.5%) *mt* chi angles: 298.4,168.2	0.06Å	-	-	-

#	Alt	Res	High B	Clash > 0.4Å	Ramachandran	Rotamer	Cβ deviation	Bond lengths	Bond angles	Cis Peptides
					-59.1,-18.1					
A 405		TRP	0.56	-	Favored (5.53%) General / -121.6,33.4	Favored (84.4%) *m100* chi angles: 300.7,103	0.10Å	-	-	-
A 406		LEU	0.67	-	Favored (18.27%) General / -142.2,127.1	Favored (10.6%) *tp* chi angles: 195.1,73	0.15Å	-	-	-
A 407		THR	0.71	-	Favored (38.02%) General / -66.6,155.4	Favored (48.7%) *p* chi angles: 56	0.20Å	-	-	-
A 408		LEU	0.7	-	Favored (75.6%) General / -56.4,-41.0	Favored (80.9%) *mt* chi angles: 288.9,167.9	0.05Å	-	-	-
A 409		GLU	0.75	-	Favored (96.72%) General / -62.9,-44.2	Favored (4.3%) *pt0* chi angles: 56.4,199.1,116.9	0.09Å	-	-	-
A 410		THR	0.75	-	Favored (81.63%) General / -66.9,-36.2	Favored (24.3%) *m* chi angles: 307.8	0.12Å	-	-	-
**#**	**Alt**	**Res**	**High B**	**Clash > 0.4Å**	**Ramachandran**	**Rotamer**	**Cβ deviation**	**Bond lengths**	**Bond angles**	**Cis Peptides**
			Avg: 0.69	Clashscore: 1.84	Outliers: 9 of 654	Poor rotamers: 9 of 581	Outliers: 13 of 633	Outliers: 1 of 656	Outliers: 66 of 656	Non-Trans: 2 of 655
A 411		ARG	0.72	-	Favored (79.72%) General / -57.0,-47.9	Favored (8.8%) *ttp80* chi angles: 166.1,194.3,78.2,103.6	0.07Å	-	-	-
A 412		GLN	0.77	-	Favored (97.42%) General / -60.6,-42.8	Favored (88.5%) *mt0* chi angles: 289,166.1,334.8	0.02Å	-	-	-
A 413		LYS	0.78	-	Favored (93.75%) General / -62.3,-39.7	Favored (93.6%) *mttt* chi angles: 297.9,173.2,184.4,177.2	0.08Å	-	-	-
A 414		ALA	0.81	-	Favored (97.32%) General / -61.4,-41.5	-	0.01Å	-	-	-
A 415		ILE	0.79	-	Favored (82.68%) Ile or Val / -58.4,-49.3	Favored (94.2%) *mt* chi angles: 291.9,168.6	0.04Å	-	-	-
A 416		THR	0.79	-	Favored (82.15%) General / -56.9,-44.4	Favored (93.1%) *m* chi angles: 299.2	0.04Å	-	-	-

A 417		LYS	0.78	-	Favored (98.01%) General / -63.6,-43.0	Favored (79.9%) *tttt* chi angles: 180.3,170.3,185.8,174	0.02Å	-	-	-
A 418		LEU	0.78	-	Favored (92.91%) General / -63.9,-44.5	Favored (21.7%) *tp* chi angles: 173.7,71.5	0.15Å	-	-	-
A 419		GLU	0.78	-	Favored (84.74%) General / -59.8,-39.4	Favored (96.8%) *mt-10* chi angles: 289.9,179.8,0.7	0.12Å	-	-	-
A 420		ALA	0.78	-	Favored (62.27%) General / -71.5,-19.0	-	0.08Å	-	-	-
A 421		LEU	0.78	-	Favored (4.72%) General / -52.1,119.8	Favored (26.2%) *tp* chi angles: 183.3,70.6	0.04Å	-	-	-
A 422		LYS	0.76	-	Favored (21.65%) General / -87.1,153.3	Favored (22.9%) *mttp* chi angles: 302.1,190,184.8,87.2	0.07Å	-	-	-
A 423		LEU	0.77	-	Favored (42.89%) General / -116.0,145.5	Favored (50.3%) *tp* chi angles: 171.8,62.4	0.03Å	-	-	-
A 424		HIS	0.76	-	Favored (36.59%) General / -123.9,121.9	Favored (73.5%) *t70* chi angles: 187.9,66.7	0.12Å	-	OUTLIER(S) worst is ND1-CG-CD2: 4.7 σ	-
A 425		PHE	0.7	-	Favored (12.04%) General / -116.5,-2.2	Allowed (0.9%) *p90* chi angles: 42.6,61.2	0.05Å	-	OUTLIER(S) worst is C-N-CA: 4.5 σ	-
A 426		ARG	0.63	-	OUTLIER (0)%) General / 135.2,152.6	Favored (55.9%) *ttt-90* chi angles: 178.3,189.3,178,280.8	0.12Å	-	-	-
A 427		THR	0.52	-	Favored (10.32%) General / -137.3,174.7	Favored (11.9%) *t* chi angles: 190.2	0.14Å	-	-	-
A 428		HIS	0.51	-	Favored (55.2%) General / -121.1,133.4	Favored (68.7%) *t70* chi angles: 185.1,82.5	0.08Å	-	OUTLIER(S) worst is ND1-CG-CD2: 4.7 σ	-
A 429		ASP	0.53	-	Favored (12.48%) General / -103.0,163.0	Favored (16.8%) *p0* chi angles: 57.7,320.2	0.08Å	-	-	-
A		ASP	0.51	-	Favored (7.39%)	Favored (70.1%) *m-30*	0.17Å	-	-	-

#	Alt	Res	High B	Clash > 0.4Å	Ramachandran	Rotamer	Cβ deviation	Bond lengths	Bond angles	Cis Peptides
430					General / -87.1,176.9	chi angles: 297.7,310.6				
			Avg: 0.69	Clashscore: 1.84	Outliers: 9 of 654	Poor rotamers: 9 of 581	Outliers: 13 of 633	Outliers: 1 of 656	Outliers: 66 of 656	Non-Trans: 2 of 655
A 431		ASN	0.59	-	Favored (2.59%) General / -80.3,13.0	Favored (68.5%) *m-40* chi angles: 297.7,291.8	0.03Å	-	-	-
A 432		LYS	0.58	-	Favored (60.43%) General / -74.1,-11.8	Favored (23.8%) *mttt* chi angles: 294.1,171.4,194.6,148	0.03Å	-	OUTLIER(S) worst is C-N-CA: 5.2 σ	-
A 433		LEU	0.68	-	Allowed (0.79%) General / -44.3,-30.2	Favored (48.7%) *tp* chi angles: 183.2,64.1	0.16Å	-	-	-
A 434		ILE	0.65	-	Favored (78.62%) Ile or Val / -56.8,-42.8	Favored (93%) *mt* chi angles: 295.9,171.1	0.06Å	-	-	-
A 435		ARG	0.59	-	Favored (73.7%) General / -65.2,-32.1	Favored (77.4%) *mtm180* chi angles: 297.9,184.4,296.8,164.9	0.04Å	-	-	-
A 436		GLN	0.65	-	Favored (81.2%) General / -61.2,-36.9	Favored (85.2%) *mt0* chi angles: 296,178.9,352.8	0.08Å	-	-	-
A 437		MET	0.67	-	Favored (55.32%) General / -95.0,3.1	Allowed (0.7%) *pp-130* chi angles: 62.6,72.9,178.8	0.06Å	-	-	-
A 438		HIS	0.61	-	Favored (5.54%) General / -46.0,-36.4	Favored (80.9%) *t70* chi angles: 177.6,80.8	0.03Å	-	OUTLIER(S) worst is ND1-CG-CD2: 4.3 σ	-
A 439		GLY	0.69	-	Favored (90.28%) Glycine / -81.3,-2.0	-	-	-	-	-
A 440		LEU	0.72	-	Favored (23.36%) General / -99.0,110.8	Favored (85.1%) *mt* chi angles: 295.8,170.5	0.05Å	-	-	-
A 441		ILE	0.69	-	Favored (71.48%) Ile or Val / -119.9,123.8	Favored (89.1%) *mt* chi angles: 295.9,173.2	0.03Å	-	-	-
A 442		LEU	0.76	-	Favored (53.57%) General /	Favored (40.5%) *mt* chi angles: 306,173	0.06Å	-	-	-

					-118.6,136.9					
A 443		ASN	0.74	-	Favored (49.67%) General / -102.7,130.4	Favored (55.3%) *m-40* chi angles: 302.4,287	0.10Å	-	-	-
A 444		MET	0.7	-	Favored (61.29%) General / -73.5,-14.9	Favored (96.4%) *mtp* chi angles: 291,177.4,66.8	0.05Å	-	-	-
A 445		ASP	0.68	-	Favored (51.28%) General / -91.3,-7.4	Favored (14.9%) *p0* chi angles: 56.9,310.1	0.09Å	-	-	-
A 446		ARG	0.71	-	Allowed (1.39%) General / -128.8,70.0	Favored (80.6%) *mtt180* chi angles: 299.6,175.9,188.3,194.1	0.07Å	-	-	-
A 447		PHE	0.71	-	Favored (86.04%) General / -60.3,-47.4	Favored (20.8%) *t80* chi angles: 177.9,49.1	0.04Å	-	-	-
A 448		TYR	0.72	-	Favored (95.48%) Pre-Pro / -59.8,-48.3	Favored (7.5%) *t80* chi angles: 183,110	0.21Å	-	OUTLIER(S) worst is CA-C-N: 5.4 σ	-
A 449		PRO	0.79	-	Favored (96.27%) Trans-Pro / -57.2,-33.6	Favored (67.6%) *Cg_exo* chi angles: 328.7,37.6,329.9	0.07Å	-	-	-
A 450		ASN	0.78	-	Favored (90.24%) General / -61.9,-46.2	Favored (3.3%) *m110* chi angles: 297,177.5	0.10Å	-	-	-
**#**	**Alt**	**Res**	**High B**	**Clash > 0.4Å**	**Ramachandran**	**Rotamer**	**Cβ deviation**	**Bond lengths**	**Bond angles**	**Cis Peptides**
			Avg: 0.69	Clashscore: 1.84	Outliers: 9 of 654	Poor rotamers: 9 of 581	Outliers: 13 of 633	Outliers: 1 of 656	Outliers: 66 of 656	Non-Trans: 2 of 655
A 451		LEU	0.79	-	Favored (99.63%) General / -63.0,-41.9	Favored (13.5%) *tp* chi angles: 191.6,53.5	0.10Å	-	-	-
A 452		VAL	0.78	-	Favored (89.14%) Ile or Val / -59.0,-43.5	Favored (99.7%) *t* chi angles: 175.5	0.07Å	-	-	-
A 453		SER	0.8	-	Favored (94.34%) General / -60.5,-41.4	Favored (73%) *m* chi angles: 295.4	0.10Å	-	-	-
A 454		VAL	0.79	-	Favored (97.83%) Ile or Val / -61.5,-44.0	Favored (57.2%) *t* chi angles: 170.4	0.04Å	-	-	-

A 455		LEU	0.78	-	Favored (94.61%) General / -60.0,-44.5	Favored (95.6%) *mt* chi angles: 293.5,173.9	0.06Å	-	-	-
A 456		GLN	0.76	-	Favored (77.61%) General / -60.8,-49.4	Favored (84.2%) *mt0* chi angles: 291.6,180.4,1.7	0.03Å	-	-	-
A 457		TRP	0.72	-	Favored (97.61%) General / -61.3,-44.2	Favored (88.5%) *t60* chi angles: 179.8,84.2	0.05Å	-	-	-
A 458		LYS	0.75	-	Favored (88.81%) General / -61.8,-46.6	Favored (76.1%) *tttt* chi angles: 179.4,182.7,172,187	0.05Å	-	-	-
A 459		THR	0.76	-	Favored (78.16%) General / -67.3,-44.9	Favored (88.9%) *m* chi angles: 298.4	0.06Å	-	-	-
A 460		GLN	0.71	-	Favored (95.14%) General / -61.3,-40.7	Favored (77.4%) *mm-40* chi angles: 294.4,296.3,291.4	0.05Å	-	-	-
A 461		ARG	0.69	-	Favored (83.03%) General / -61.0,-37.6	Favored (70.1%) *ttt-90* chi angles: 185.9,177.5,181.4,270.1	0.03Å	-	-	-
A 462		GLY	0.77	-	Favored (37.97%) Glycine / -73.5,-43.3	-	-	-	-	-
A 463		LEU	0.72	-	Favored (88.87%) General / -64.7,-37.8	Favored (95%) *mt* chi angles: 295.7,172.7	0.06Å	-	-	-
A 464		ALA	0.75	-	Favored (60.81%) General / -71.0,-11.2	-	0.03Å	-	-	-
A 465		LYS	0.7	-	Favored (61.67%) General / -70.2,-12.6	Favored (71.4%) *mttt* chi angles: 283.1,185.1,166.7,189	0.12Å	-	-	-
A 466		LEU	0.72	-	Favored (72.79%) General / -55.7,-40.6	Favored (50.8%) *tp* chi angles: 180.7,65.5	0.06Å	-	-	-
A 467		MET	0.67	-	Favored (32.55%) General / -102.3,14.2	Favored (97.9%) *mmm* chi angles: 295.1,300.5,294.2	0.03Å	-	-	-
A		THR	0.7	-	Favored (10.49%)	Favored (32.4%) *p*	0.20Å	-	-	-

468					General / -103.7,166.3	chi angles: 69				
A 469		GLU	0.68	-	Favored (44.17%) Pre-Pro / -84.8,133.2	Favored (71.3%) *tt0* chi angles: 189.8,169.8,7.8	0.04Å	-	-	-
A 470		PRO	0.7	0.46Å HG3 with A 191 HIS CE1	Favored (11.38%) Trans-Pro / -47.5,125.8	Favored (93.8%) *Cg_exo* chi angles: 331.3,38.2,326.3	0.05Å	-	-	-
**#**	**Alt**	**Res**	**High B**	**Clash > 0.4Å**	**Ramachandran**	**Rotamer**	**Cβ deviation**	**Bond lengths**	**Bond angles**	**Cis Peptides**
			Avg: 0.69	Clashscore: 1.84	Outliers: 9 of 654	Poor rotamers: 9 of 581	Outliers: 13 of 633	Outliers: 1 of 656	Outliers: 66 of 656	Non-Trans: 2 of 655
A 471		VAL	0.64	-	Allowed (0.12%) Ile or Val / -85.5,27.0	Favored (4.1%) *m* chi angles: 287.5	0.12Å	-	OUTLIER(S) worst is C-N-CA: 5.3 σ	-
A 472		ALA	0.58	-	Favored (61.96%) General / -51.3,-44.6	-	0.14Å	-	-	-
A 473		GLU	0.66	-	Favored (83.57%) General / -62.8,-47.4	Favored (3.2%) *tt0* chi angles: 185.4,146,329.6	0.12Å	-	-	-
A 474		ASP	0.6	-	Favored (22.77%) General / -76.6,-47.3	Favored (69.4%) *m-30* chi angles: 298.9,312.6	0.03Å	-	-	-
A 475		GLU	0.63	-	Favored (2.62%) General / -64.7,111.1	Favored (44.9%) *tt0* chi angles: 186.4,179.8,48.5	0.13Å	-	-	-
A 476		VAL	0.68	-	Favored (12.75%) Ile or Val / -60.9,145.2	Favored (3%) *t* chi angles: 192.6	0.10Å	-	-	-
A 477		HIS	0.62	-	Favored (28.37%) General / -81.0,151.6	Favored (54%) *t-90* chi angles: 198.7,289.3	0.05Å	-	OUTLIER(S) worst is CA-CB-CG: 5.1 σ	-
A 478		LYS	0.66	-	Favored (59.84%) General / -77.4,-11.4	Favored (98.8%) *mttt* chi angles: 294.3,179.9,175.3,183.8	0.02Å	-	-	-
A 479		LEU	0.53	-	Favored (58.14%) Pre-Pro / -127.5,160.9	Favored (70%) *mt* chi angles: 295.5,181.3	0.10Å	-	-	-
A 480		PRO	0.62	-	Favored (2.7%) Trans-Pro /	Favored (55%) *Cg_endo* chi angles:	0.08Å	-	-	-

					-72.5,74.3	25.8,327.7,26.3				
A 481		HIS	0.56	-	Favored (50.51%) General / -130.4,142.0	Favored (43.7%) *t70* chi angles: 192.6,57.1	0.08Å	-	OUTLIER(S) worst is ND1-CG-CD2: 4.5 σ	-
A 482		TYR	0.77	-	Favored (40.5%) General / -71.5,130.1	Favored (59.1%) *t80* chi angles: 168.7,72.3	0.06Å	-	-	-
A 483		GLU	0.77	-	Favored (29.84%) General / -105.7,115.2	Favored (11.8%) *tp30* chi angles: 187.5,50.3,63.7	0.03Å	-	-	-
A 484		ILE	0.76	-	Favored (81.01%) Ile or Val / -60.6,-39.3	Favored (4.7%) *pt* chi angles: 49.6,181.7	0.10Å	-	-	-
A 485		GLN	0.71	-	Favored (80.08%) General / -64.1,-35.2	Favored (33.5%) *mm-40* chi angles: 286.8,284.9,283	0.04Å	-	-	-
A 486		LYS	0.74	-	Favored (23.69%) General / -106.4,-1.0	Favored (40.5%) *mtmt* chi angles: 302.9,197.5,289.7,183.3	0.11Å	-	-	-
A 487		ASN	0.77	-	Allowed (0.93%) General / 38.5,63.0	Favored (65.2%) *t0* chi angles: 193,39.2	0.27Å	-	-	-
A 488		ARG	0.76	-	Favored (17.34%) General / -145.2,129.0	Favored (26.3%) *ttt180* chi angles: 189.8,165.6,199.4,195.3	0.03Å	-	-	-
A 489		ILE	0.77	0.43Å HD11 with A 386 TRP CD1	Favored (5.55%) Ile or Val / -96.8,148.3	Favored (17.9%) *pt* chi angles: 52.5,168.6	0.08Å	-	-	-
A 490		GLN	0.75	-	Favored (20.58%) General / -138.5,124.9	Favored (42.1%) *mt0* chi angles: 305.2,193.8,47	0.04Å	-	-	-
**#**	**Alt**	**Res**	**High B**	**Clash > 0.4Å**	**Ramachandran**	**Rotamer**	**Cβ deviation**	**Bond lengths**	**Bond angles**	**Cis Peptides**
			Avg: 0.69	Clashscore: 1.84	Outliers: 9 of 654	Poor rotamers: 9 of 581	Outliers: 13 of 633	Outliers: 1 of 656	Outliers: 66 of 656	Non-Trans: 2 of 655
A 491		VAL	0.76	-	Favored (35.45%) Pre-Pro / -118.8,88.4	Allowed (1.5%) *t* chi angles: 195.5	0.02Å	-	-	-
A 492		PRO	0.75	-	Favored (9.34%) Trans-Pro /	Favored (22.5%) *Cg_exo* chi angles:	0.09Å	-	-	-

					-55.5,160.3	341.6,33.8,322.6				
A 493		ILE	0.67	-	Favored (28.71%) Ile or Val / -53.0,-38.4	Favored (2.4%) *pt* chi angles: 44,178.6	0.13Å	-	-	-
A 494		THR	0.72	-	Favored (59.28%) General / -50.8,-43.9	Favored (40.9%) *m* chi angles: 305	0.05Å	-	-	-
A 495		PHE	0.73	-	Favored (17.48%) General / -83.5,-41.9	Favored (20%) *m-80* chi angles: 277.9,69.2	0.03Å	-	OUTLIER(S) worst is CA-CB-CG: 4.5 σ	-
A 496		LEU	0.71	-	Favored (56.43%) General / -79.0,-16.8	Favored (2%) *pp* chi angles: 68.4,94.3	0.07Å	-	-	-
A 497		GLN	0.5	0.47Å HE22 with A 375 HIS CE1	Favored (35.23%) General / -100.7,-1.4	Favored (88.9%) *tp40* chi angles: 185.4,60.2,61.2	0.15Å	-	-	-
A 498		ALA	0.6	-	Favored (25.51%) General / -75.0,123.6	-	0.06Å	-	-	-
A 499		ARG	0.52	-	Favored (53.16%) General / -64.3,-14.0	Favored (72.3%) *mtp180* chi angles: 293.1,179.8,66.5,205.9	0.04Å	-	-	-
A 500		PHE	0.57	-	Favored (46.33%) General / -85.9,-13.9	Favored (17.8%) *m-10* chi angles: 297.3,347.9	0.07Å	-	-	-
A 501		PHE	0.75	-	Allowed (0.29%) General / -106.1,-109.2	Favored (23.6%) *m-80* chi angles: 281.5,69.6	0.09Å	-	-	-
A 502		TRP	0.72	-	Favored (24.69%) General / -92.2,145.2	Favored (6.3%) *t-100* chi angles: 171.4,289.1	0.06Å	-	-	-
A 503		ASP	0.75	-	Favored (12.02%) Pre-Pro / -147.8,137.3	Favored (38%) *t0* chi angles: 179.1,338	0.17Å	-	OUTLIER(S) worst is CA-C-N: 6.0 σ	-
A 504		PRO	0.76	-	Favored (55.42%) Trans-Pro / -56.2,-25.4	Favored (57.5%) *Cg_endo* chi angles: 26.1,323.4,33.1	0.04Å	-	-	-
A 505		ALA	0.76	0.67Å HB1 with A 37 LEU HD13	Favored (44.32%) General / -96.0,-3.8	-	0.15Å	-	OUTLIER(S) worst is N-CA-CB: 4.2 σ	-
					Favored	Favored (13.7%) *m-*				

A 506		TYR	0.74	-	(27.64%) Pre-Pro / -92.4,163.2	*10* chi angles: 289.3,327.3	0.08Å	-	-	-
A 507		PRO	0.8	-	Favored (12.98%) Trans-Pro / -47.6,142.9	Favored (89%) *Cg_exo* chi angles: 333.4,34.6,330.1	0.13Å	-	-	-
A 508		ASN	0.77	-	Favored (86.95%) General / -62.1,-37.9	Favored (64.7%) *m-40* chi angles: 289.2,295.8	0.16Å	-	-	-
A 509		GLY	0.82	-	Favored (56.4%) Glycine / -54.1,-36.4	-	-	-	-	-
A 510		LEU	0.79	-	Favored (49.54%) General / -73.5,-46.2	Favored (50.1%) *mt* chi angles: 303,171.1	0.07Å	-	-	-
**#**	**Alt**	**Res**	**High B**	**Clash > 0.4Å**	**Ramachandran**	**Rotamer**	**Cβ deviation**	**Bond lengths**	**Bond angles**	**Cis Peptides**
			Avg: 0.69	Clashscore: 1.84	Outliers: 9 of 654	Poor rotamers: 9 of 581	Outliers: 13 of 633	Outliers: 1 of 656	Outliers: 66 of 656	Non-Trans: 2 of 655
A 511		LYS	0.77	-	Favored (83.94%) General / -58.3,-41.2	Favored (7.4%) *mtmt* chi angles: 286,190.4,253.9,168	0.06Å	-	-	-
A 512		TYR	0.79	-	Favored (90.85%) General / -66.2,-41.0	Favored (46.8%) *m-80* chi angles: 282.8,79.7	0.11Å	-	-	-
A 513		ALA	0.8	-	Favored (79.68%) General / -65.5,-35.0	-	0.08Å	-	-	-
A 514		THR	0.77	0.40Å HG22 with A 634 LEU HD11	Allowed (0.15%) General / -88.3,-88.0	Favored (75.5%) *p* chi angles: 59.9	0.13Å	-	-	-
A 515		LEU	0.79	-	Favored (73.91%) General / -68.9,-33.5	Favored (8.5%) *tp* chi angles: 187.6,45.7	0.07Å	-	-	-
A 516		GLY	0.81	-	Favored (95.48%) Glycine / -61.4,-38.3	-	-	-	-	-
A 517		VAL	0.81	-	Favored (97.23%) Ile or Val / -63.9,-45.3	Favored (87.1%) *t* chi angles: 173.8	0.05Å	-	-	-
A		LEU	0.79	-	Favored (98.53%)	Favored (45.6%) *tp*	0.09Å	-	-	-

#	Alt	Res	High B	Clash > 0.4Å	Ramachandran	Rotamer	Cβ deviation	Bond lengths	Bond angles	Cis Peptides
518					General / -61.5,-42.3	chi angles: 184.6,60.8				
A 519		LEU	0.81	-	Favored (89.83%) General / -60.1,-46.4	Favored (51.5%) *mt* chi angles: 284.9,166.4	0.19Å	-	-	-
A 520		ALA	0.82	-	Favored (99.24%) General / -61.8,-42.6	-	0.10Å	-	-	-
A 521		ARG	0.77	-	Favored (91.56%) General / -59.4,-42.3	Favored (35.8%) *ttt-90* chi angles: 184.9,189.6,189.2,257.3	0.12Å	-	-	-
A 522		GLN	0.78	-	Favored (96.57%) General / -63.5,-40.3	Favored (84.6%) *mt0* chi angles: 291.6,185,306.2	0.09Å	-	-	-
A 523		MET	0.79	-	Favored (97.77%) General / -62.6,-43.9	Favored (38.8%) *mtt* chi angles: 297.9,185.8,198.3	0.11Å	-	OUTLIER(S) worst is CG-SD-CE: 5.3 σ	-
A 524		LEU	0.79	-	Favored (69.7%) General / -65.0,-28.8	Allowed (0.6%) *mm* chi angles: 272.6,307.5	0.08Å	-	-	-
A 525		HIS	0.78	-	Favored (16.91%) General / -61.6,-12.8	Favored (40.4%) *m-70* chi angles: 284.7,306.5	0.09Å	-	OUTLIER(S) worst is ND1-CG-CD2: 4.1 σ	-
A 526		GLY	0.79	-	Favored (89.03%) Glycine / -79.1,-5.6	-	-	-	-	-
A 527		PHE	0.77	-	Allowed (1.95%) General / -140.9,5.3	Favored (97.9%) *m-80* chi angles: 298.2,277.3	0.07Å	-	-	-
A 528		ASP	0.77	-	OUTLIER (0.01)%) General / -59.4,-171.0	Allowed (0.7%) *p0* chi angles: 88.7,346.8	0.32Å	-	-	-
A 529		GLY	0.77	-	Favored (70.23%) Glycine / -66.9,-19.9	-	-	-	-	-
A 530		VAL	0.73	-	Favored (7.77%) Ile or Val / -98.7,-53.6	Allowed (0.9%) *t* chi angles: 155.1	0.14Å	-	-	-
**#**	**Alt**	**Res**	**High B**	**Clash > 0.4Å**	**Ramachandran**	**Rotamer**	**Cβ deviation**	**Bond lengths**	**Bond angles**	**Cis Peptides**
			Avg: 0.69	Clashscore: 1.84	Outliers: 9 of 654	Poor rotamers: 9 of 581	Outliers: 13 of	Outliers: 1 of 656	Outliers: 66 of 656	Non-Trans: 2

							633			of 655
A 531		GLY	0.76	-	Favored (76.55%) Glycine / -73.4,-23.7	-	-	-	-	-
A 532		ARG	0.69	-	Favored (61.26%) General / -72.2,-12.6	Favored (31%) *ptt90* chi angles: 76.8,180.8,180.1,70.8	0.06Å	-	-	-
A 533		ARG	0.69	-	Favored (12.69%) General / -89.4,13.8	Favored (90.3%) *mtt-85* chi angles: 298.2,182.1,174.9,283	0.10Å	-	-	-
A 534		TYR	0.71	-	Favored (53.52%) General / -126.4,138.2	Favored (29.1%) *m-80* chi angles: 280.3,72.7	0.15Å	-	-	-
A 535		ASP	0.75	-	Allowed (0.1%) General / -82.7,-151.6	Favored (53.5%) *p0* chi angles: 67.4,10.3	0.14Å	-	-	-
A 536		ALA	0.78	-	Favored (43.85%) General / -86.8,-13.9	-	0.10Å	-	-	-
A 537		TYR	0.71	-	Favored (12.97%) General / -104.0,-25.6	Favored (96.4%) *m-80* chi angles: 292.8,94.6	0.04Å	-	-	-
A 538		GLY	0.77	-	Favored (4.31%) Glycine / 117.2,22.0	-	-	-	-	-
A 539		TYR	0.68	-	Favored (36.98%) General / -96.8,136.7	Favored (7.7%) *m-80* chi angles: 293.2,65.4	0.12Å	-	-	-
A 540		LYS	0.7	-	Allowed (1.28%) General / -71.5,94.3	Favored (30.8%) *ttmt* chi angles: 182.6,176.8,296.9,183.3	0.03Å	-	-	-
A 541		ASN	0.69	-	Favored (29.84%) General / -146.6,144.9	Favored (20.5%) *t0* chi angles: 192.8,292.5	0.07Å	-	-	-
A 542		ASN	0.69	-	Favored (18.03%) General / -89.1,105.8	Favored (39%) *t0* chi angles: 192.3,12.6	0.03Å	-	-	-
A 543		TRP	0.7	-	Favored (56.38%) General / -93.7,0.3	Favored (37.3%) *p-90* chi angles: 46.1,267.3	0.11Å	-	-	-
					Favored	Favored (24.3%)				

A 544		TRP	0.7	-	(33.9%) General / -93.6,136.4	*m100* chi angles: 297.6,62.7	0.07Å	-	-	-
A 545		ASP	0.73	-	Favored (15.87%) General / -58.7,153.8	Favored (12.8%) *t70* chi angles: 176.8,45.3	0.11Å	-	-	-
A 546		HIS	0.69	-	Favored (80.7%) General / -56.6,-44.0	Favored (82.7%) *t70* chi angles: 184.5,69.5	0.05Å	-	OUTLIER(S) worst is ND1-CG-CD2: 4.2 σ	-
A 547		THR	0.7	-	Favored (86.52%) General / -66.8,-42.0	Favored (58.5%) *p* chi angles: 64.4	0.12Å	-	-	-
A 548		SER	0.75	-	Favored (93.03%) General / -60.4,-45.6	Favored (6.6%) *m* chi angles: 282	0.05Å	-	-	-
A 549		GLU	0.71	-	Favored (83.13%) General / -67.1,-43.2	Favored (18.7%) *tt0* chi angles: 180.5,161.9,62.3	0.09Å	-	-	-
A 550		SER	0.77	-	Favored (88.56%) General / -58.9,-42.1	Favored (45.2%) *t* chi angles: 179.2	0.05Å	-	-	-
**#**	**Alt**	**Res**	**High B**	**Clash > 0.4Å**	**Ramachandran**	**Rotamer**	**Cβ deviation**	**Bond lengths**	**Bond angles**	**Cis Peptides**
			Avg: 0.69	Clashscore: 1.84	Outliers: 9 of 654	Poor rotamers: 9 of 581	Outliers: 13 of 633	Outliers: 1 of 656	Outliers: 66 of 656	Non-Trans: 2 of 655
A 551		SER	0.76	-	Favored (60.47%) General / -75.1,-17.1	Favored (36.4%) *m* chi angles: 289.4	0.04Å	-	-	-
A 552		TYR	0.72	-	Favored (61.74%) General / -67.7,-49.6	Favored (56.4%) *t80* chi angles: 186.5,92.5	0.10Å	-	-	-
A 553		SER	0.77	-	Favored (97.75%) General / -61.6,-41.7	Favored (57.9%) *m* chi angles: 299.6	0.02Å	-	-	-
A 554		ARG	0.72	-	Favored (99.33%) General / -62.0,-42.6	Favored (21.6%) *ttm110* chi angles: 201.7,184.5,307.9,103.8	0.04Å	-	-	-
A 555		ARG	0.71	-	Favored (79.2%) General / -68.9,-40.5	Favored (9.9%) *mmp80* chi angles: 298.5,288.5,79.6,89.3	0.12Å	-	-	-
A					Favored				OUTLIER(S)	

556		THR	0.78	-	(70.43%) General / -65.4,-29.7	Favored (34.1%) *p* chi angles: 53.8	0.25Å	-	worst is N-CA-CB: 4.3 σ	-
A 557		GLN	0.74	-	Favored (71.48%) General / -57.8,-36.0	Allowed (1.6%) *tp-100* chi angles: 202.9,64.9,211	0.05Å	-	-	-
A 558		CYS	0.79	-	Favored (89.39%) General / -58.5,-44.5	Favored (21.3%) *t* chi angles: 190.8	0.06Å	-	-	-
A 559		PHE	0.74	-	Favored (81.7%) General / -67.0,-36.2	Favored (98.5%) *m-80* chi angles: 297.4,89.7	0.11Å	-	-	-
A 560		GLN	0.72	-	Favored (54.82%) General / -58.5,-53.8	Favored (75.3%) *mt0* chi angles: 296,175.9,55.7	0.12Å	-	-	-
A 561		GLU	0.7	-	Favored (98.71%) General / -60.9,-43.3	Favored (97.5%) *mt-10* chi angles: 295.1,180.8,355.7	0.07Å	-	-	-
A 562		GLN	0.7	-	Favored (81.56%) General / -58.4,-40.2	Favored (36.5%) *tt0* chi angles: 187.8,192.8,13.5	0.08Å	-	-	-
A 563		TYR	0.67	-	Allowed (0.98%) General / -79.2,-65.6	Allowed (0.6%) *t80* chi angles: 211.3,42.8	0.18Å	-	-	-
A 564		ALA	0.69	-	Favored (83.86%) General / -67.6,-41.5	-	0.15Å	-	-	-
A 565		ILE	0.53	-	Favored (45.5%) Ile or Val / -73.0,-34.4	Allowed (0.8%) *mp* chi angles: 270.3,63	0.24Å	-	-	-
A 566		PHE	0.41	-	Favored (19.64%) General / -109.0,-0.2	Favored (31.9%) *m-80* chi angles: 299.2,127.9	0.12Å	-	-	-
A 567		VAL	0.6	0.45Å HG11 with A 653 LEU HD13	Favored (57.97%) Ile or Val / -108.2,119.5	Favored (45.1%) *t* chi angles: 168.7	0.04Å	-	-	-
A 568		GLU	0.58	-	Favored (53.86%) General / -122.7,131.6	Favored (87.8%) *tt0* chi angles: 187.4,174.2,5.5	0.06Å	-	-	-
A 569		TYR	0.51	-	Allowed (1.11%) General /	Favored (16.4%) *t80* chi angles: 192.7,55.1	0.14Å	-	-	-

#	Alt	Res	High B	Clash > 0.4Å	Ramachandran	Rotamer	Cβ deviation	Bond lengths	Bond angles	Cis Peptides
					-116.9,-165.9					
A 570		LYS	0.52	-	Favored (65.24%) General / -66.1,-18.3	Favored (58.1%) *pttt* chi angles: 60.9,179.4,185.4,179.3	2.32Å	-	-	-
#	Alt	Res	High B	Clash > 0.4Å	Ramachandran	Rotamer	Cβ deviation	Bond lengths	Bond angles	Cis Peptides
			Avg: 0.69	Clashscore: 1.84	Outliers: 9 of 654	Poor rotamers: 9 of 581	Outliers: 13 of 633	Outliers: 1 of 656	Outliers: 66 of 656	Non-Trans: 2 of 655
A 571		GLY	0.47	-	Allowed (1.32%) Glycine / -94.5,-76.1	-	-	-	-	-
A 572		LYS	0.55	-	Allowed (0.4%) Pre-Pro / -164.6,-174.5	Favored (40.3%) *pttt* chi angles: 62.6,167.3,189,179.9	0.11Å	-	OUTLIER(S) worst is CA-C-N: 5.6 σ	-
A 573		PRO	0.64	-	Favored (40.53%) Trans-Pro / -54.5,130.5	Favored (28.9%) *Cg_exo* chi angles: 340.2,32.9,326.3	0.08Å	-	-	-
A 574		VAL	0.6	-	Favored (2.21%) Ile or Val / -55.5,151.1	Favored (15.1%) *m* chi angles: 292.2	0.11Å	-	-	-
A 575		GLN	0.59	-	Favored (17.59%) General / -86.2,106.8	Favored (38.8%) *tp40* chi angles: 190.2,68.1,75.7	0.10Å	-	-	-
A 576		ASP	0.66	-	Favored (4.26%) General / -53.8,-59.7	Favored (4.2%) *p0* chi angles: 44.6,1.5	0.09Å	-	OUTLIER(S) worst is CA-CB-CG: 4.9 σ	-
A 577		LYS	0.65	-	Favored (84.02%) General / -62.4,-47.5	Favored (92.8%) *mttt* chi angles: 290.9,173,182.7,185.2	0.13Å	-	-	-
A 578		ASP	0.64	-	Favored (94.24%) General / -59.7,-43.3	Favored (98.6%) *m-30* chi angles: 288.2,346	0.08Å	-	-	-
A 579		LEU	0.69	-	Favored (57.22%) General / -92.3,-1.2	Favored (14.4%) *mt* chi angles: 292.5,156	0.04Å	-	-	-
A 580		LEU	0.73	-	Favored (82.79%) General / -57.6,-42.2	Favored (74.3%) *tp* chi angles: 177.8,62	0.08Å	-	-	-
A 581		ARG	0.71	-	Favored (80.01%) General /	Favored (78.7%) *mtp180* chi angles:	0.20Å	-	-	-

					-56.4,-46.8	291.9,164.3,62.1,186.5				
A 582		ARG	0.71	-	Favored (85.63%) General / -65.9,-44.3	Favored (91.1%) *mtt180* chi angles: 297,183.9,170.4,175.5	0.04Å	-	-	-
A 583		ILE	0.75	-	Favored (98.59%) Ile or Val / -63.3,-43.4	Favored (43.5%) *mm* chi angles: 297.5,301.3	0.14Å	-	-	-
A 584		VAL	0.78	-	Favored (95.22%) Ile or Val / -60.1,-45.8	Favored (68.2%) *t* chi angles: 171.9	0.02Å	-	-	-
A 585		ALA	0.78	-	Favored (83.73%) General / -61.9,-47.7	-	0.05Å	-	-	-
A 586		ASP	0.77	-	Favored (98.31%) General / -63.6,-42.3	Favored (78.1%) *m-30* chi angles: 296.2,330.1	0.04Å	-	-	-
A 587		ASN	0.77	-	Favored (53.56%) General / -68.2,-50.5	Favored (6.4%) *m-40* chi angles: 280,257.5	0.07Å	-	-	-
A 588		GLY	0.8	-	Favored (72.6%) Glycine / -66.0,-27.2	-	-	-	-	-
A 589		ALA	0.79	-	Favored (93.31%) General / -59.8,-45.1	-	0.12Å	-	-	-
A 590		LEU	0.79	-	Favored (85.46%) General / -62.6,-47.0	Favored (50.2%) *tp* chi angles: 183,63.9	0.07Å	-	-	-
**#**	**Alt**	**Res**	**High B**	**Clash > 0.4Å**	**Ramachandran**	**Rotamer**	**Cβ deviation**	**Bond lengths**	**Bond angles**	**Cis Peptides**
			Avg: 0.69	Clashscore: 1.84	Outliers: 9 of 654	Poor rotamers: 9 of 581	Outliers: 13 of 633	Outliers: 1 of 656	Outliers: 66 of 656	Non-Trans: 2 of 655
A 591		ASP	0.78	-	Favored (80.09%) General / -66.4,-45.4	Favored (71.6%) *m-30* chi angles: 293.1,318.1	0.12Å	-	OUTLIER(S) worst is CA-CB-CG: 4.8 σ	-
A 592		ILE	0.8	-	Favored (96.66%) Ile or Val / -61.0,-46.4	Favored (46.1%) *mm* chi angles: 300.4,303	0.03Å	-	-	-
A 593		ALA	0.82	-	Favored (97.02%) General / -63.9,-43.1	-	0.04Å	-	-	-

A 594		TYR	0.8	-	Favored (89.07%) General / -64.9,-44.6	Favored (42.7%) *t80* chi angles: 190.4,70	0.04Å	-	-	-
A 595		ARG	0.77	-	Favored (96.79%) General / -61.2,-41.4	Favored (43.1%) *ttt180* chi angles: 177.5,169,163.1,162.2	0.06Å	-	-	-
A 596		ALA	0.82	-	Favored (88.01%) General / -60.0,-40.1	-	0.03Å	-	-	-
A 597		TYR	0.78	-	Favored (98.43%) General / -63.5,-41.7	Favored (7.6%) *t80* chi angles: 195.7,48.6	0.11Å	-	-	-
A 598		GLN	0.73	-	Favored (77.14%) General / -62.4,-34.7	Favored (96.3%) *mt0* chi angles: 291.1,179.6,309.4	0.07Å	-	-	-
A 599		GLN	0.72	-	Favored (95.98%) General / -64.6,-41.8	Favored (89.8%) *mt0* chi angles: 292,179.9,355.2	0.04Å	-	-	-
A 600		TRP	0.66	-	Favored (88.09%) General / -62.4,-46.5	Favored (47.4%) *t-100* chi angles: 180,272	0.05Å	-	OUTLIER(S) worst is CG-CD2-CE3: 5.0 σ	-
A 601		LEU	0.64	-	Favored (6.79%) General / -86.4,61.4	Favored (15.5%) *mt* chi angles: 302.3,192	0.10Å	-	-	-
A 602		LYS	0.55	-	Favored (30.6%) General / -89.6,139.4	Favored (85.2%) *tttt* chi angles: 187.1,176.6,178.4,184.2	0.08Å	-	-	-
A 603		ASN	0.43	-	Favored (88.34%) General / -59.3,-41.2	Favored (34.4%) *p0* chi angles: 65.3,331.8	0.17Å	-	-	-
A 604		ALA	0.42	-	Allowed (0.47%) General / -83.7,33.3	-	0.08Å	-	-	-
A 605		ALA	0.37	-	Favored (41.53%) General / -97.2,9.7	-	0.04Å	-	OUTLIER(S) worst is C-N-CA: 4.8 σ	-
A 606		GLU	0.33	-	OUTLIER (0.01)%) General / 129.0,-31.0	Favored (77.9%) *tt0* chi angles: 190.7,173.8,359	0.29Å	-	-	-
A 607		THR	0.32	-	Favored (18.75%) Pre-Pro /	Favored (3.9%) *t* chi angles: 177.9	0.10Å	-	OUTLIER(S) worst is CA-C-	-

					-162.9,158.4				N: 4.3 σ	
A 608		PRO	0.47	-	Favored (79.83%) Trans-Pro / -57.2,-28.5	Favored (82.1%) *Cg_exo* chi angles: 329.2,36.3,332.1	0.04Å	-	-	-
A 609		VAL	0.47	-	Favored (29.22%) Ile or Val / -72.4,-28.7	Favored (38.1%) *t* chi angles: 183.8	0.07Å	-	-	-
A 610		ILE	0.41	-	Favored (41.36%) Ile or Val / -75.1,-38.1	Favored (5.4%) *mm* chi angles: 306.5,284.1	0.20Å	-	-	-
**#**	**Alt**	**Res**	**High B**	**Clash > 0.4Å**	**Ramachandran**	**Rotamer**	**Cβ deviation**	**Bond lengths**	**Bond angles**	**Cis Peptides**
			Avg: 0.69	Clashscore: 1.84	Outliers: 9 of 654	Poor rotamers: 9 of 581	Outliers: 13 of 633	Outliers: 1 of 656	Outliers: 66 of 656	Non-Trans: 2 of 655
A 611		TYR	0.36	-	Favored (72.37%) General / -60.5,-50.7	Favored (4.8%) *t80* chi angles: 176.6,27	0.06Å	-	-	-
A 612		GLN	0.56	-	Favored (21.51%) General / -124.2,115.7	Favored (77.3%) *mt0* chi angles: 293.6,186.5,343.9	0.02Å	-	-	-
A 613		ARG	0.6	-	Favored (44.38%) General / -57.7,142.7	Favored (38.6%) *mmm160* chi angles: 304.9,297.8,294.4,176.4	0.05Å	-	-	-
A 614		GLU	0.61	-	Favored (53.6%) General / -56.3,135.5	Favored (89.7%) *mt-10* chi angles: 294.3,184.1,340	0.02Å	-	-	-
A 615		ARG	0.7	-	Favored (50.42%) General / -62.2,146.6	Favored (69.1%) *mtp180* chi angles: 286.3,182.6,60.9,202.3	0.05Å	-	-	-
A 616		LEU	0.76	-	Favored (84.46%) Pre-Pro / -71.5,140.4	Favored (70.7%) *mt* chi angles: 293,179.6	0.08Å	-	-	-
A 617		PRO	0.78	-	Favored (36.94%) Trans-Pro / -75.3,150.3	Favored (58.2%) *Cg_endo* chi angles: 32.1,324.5,25.4	0.03Å	-	-	-
A 618		LEU	0.72	-	Favored (4.35%) General / 74.9,6.4	Favored (17.1%) *mt* chi angles: 309.7,169.9	0.18Å	-	-	-
A 619		LEU	0.76	-	Favored (15.01%) General / -130.9,114.2	Favored (33.9%) *mt* chi angles: 284.7,161.8	0.05Å	-	-	-

A 620		ASP	0.77	-	Favored (2.33%) General / -94.9,59.2	Favored (9%) *m-30* chi angles: 281.4,291.1	0.23Å	-	OUTLIER(S) worst is CA-CB-CG: 4.6 σ	-
A 621		HIS	0.77	-	Favored (52.56%) General / -131.3,147.3	Favored (48.1%) *m90* chi angles: 305.7,86.2	0.09Å	-	-	-
A 622		ASP	0.77	-	Favored (10.57%) General / -83.3,174.6	Favored (2.5%) *p0* chi angles: 71.9,40.9	0.22Å	-	-	-
A 623		HIS	0.76	-	Favored (60.68%) General / -74.9,-16.3	Favored (39.3%) *m-70* chi angles: 278.5,275.1	0.13Å	-	OUTLIER(S) worst is ND1-CG-CD2: 4.1 σ	-
A 624		ASN	0.77	-	Favored (50.1%) General / -74.3,-45.0	Favored (75.7%) *m-40* chi angles: 299.9,328.3	0.09Å	-	-	-
A 625		GLN	0.77	-	Favored (93.8%) General / -61.5,-45.5	Favored (92.5%) *mt0* chi angles: 294,177.2,346.3	0.04Å	-	-	-
A 626		ILE	0.79	-	Favored (54.69%) Ile or Val / -56.8,-37.6	Favored (73.3%) *mt* chi angles: 288.4,167.5	0.12Å	-	-	-
A 627		PHE	0.78	-	Favored (39.23%) General / -54.6,-54.6	Favored (13.6%) *t80* chi angles: 175,281.9	0.07Å	-	-	-
A 628		TYR	0.78	-	Favored (93.66%) General / -64.8,-39.5	Favored (49.5%) *m-80* chi angles: 286.6,112.4	0.03Å	-	-	-
A 629		LEU	0.78	-	Favored (96.7%) General / -61.4,-44.7	OUTLIER (0%) chi angles: 217.1,226.3	0.10Å	-	-	-
A 630		GLY	0.8	-	Favored (75.42%) Glycine / -68.9,-33.7	-	-	-	-	-
**#**	**Alt**	**Res**	**High B**	**Clash > 0.4Å**	**Ramachandran**	**Rotamer**	**Cβ deviation**	**Bond lengths**	**Bond angles**	**Cis Peptides**
			Avg: 0.69	Clashscore: 1.84	Outliers: 9 of 654	Poor rotamers: 9 of 581	Outliers: 13 of 633	Outliers: 1 of 656	Outliers: 66 of 656	Non-Trans: 2 of 655
A 631		TYR	0.78	-	Favored (90%) General / -60.4,-46.4	Favored (30.9%) *t80* chi angles: 162.7,77	0.08Å	-	-	-
A		ALA	0.78	-	Favored (79.15%)	-	0.06Å	-	-	-

632					General / -67.1,-35.1					
A 633		GLN	0.74	-	Favored (6.69%) General / -86.1,14.2	Favored (92.4%) *mt0* chi angles: 293.1,178.8,348.6	0.08Å	-	-	-
A 634		LEU	0.75	0.40Å HD11 with A 514 THR HG22	Favored (80.6%) General / -68.7,-38.8	OUTLIER (0.1%) chi angles: 268,36.1	0.09Å	-	-	-
A 635		TYR	0.7	-	Favored (6.24%) General / -115.6,32.2	Favored (17.9%) *m-10* chi angles: 289.2,349.3	0.12Å	-	-	-
A 636		CYS	0.77	-	Favored (28.36%) General / -91.0,115.9	Favored (53.7%) *m* chi angles: 302.7	0.12Å	-	-	-
A 637		THR	0.7	0.54Å HB with A 82 ASN HB3	Favored (20.98%) General / -163.9,167.2	Favored (2.4%) *t* chi angles: 175.1	0.11Å	-	OUTLIER(S) worst is CA-CB-OG1: 4.9 σ	-
A 638		ASP	0.65	-	Favored (32.14%) General / -147.8,164.9	Favored (3.5%) *t0* chi angles: 218.6,331.5	0.13Å	-	-	-
A 639		TYR	0.57	0.43Å CE1 with A 80 ARG HB3	Favored (49.61%) Pre-Pro / -124.4,145.7	Allowed (0.3%) *m-10* chi angles: 260.8,14.4	0.12Å	-	OUTLIER(S) worst is CA-CB-CG: 4.4 σ	-
A 640		PRO	0.64	-	Favored (40.25%) Trans-Pro / -62.7,133.1	Favored (12.8%) *Cg_exo* chi angles: 345.8,29.1,326.5	0.08Å	-	-	-
A 641		GLU	0.61	-	Favored (16.82%) General / -50.7,-31.5	Favored (95%) *mt-10* chi angles: 296.9,173.7,352.2	0.08Å	-	-	-
A 642		THR	0.64	-	Favored (93.2%) General / -60.7,-40.7	Favored (17.7%) *m* chi angles: 290	0.13Å	-	-	-
A 643		VAL	0.62	-	Favored (13.06%) Ile or Val / -79.3,-27.1	OUTLIER (0%) chi angles: 93.4	0.19Å	-	-	-
A 644		GLU	0.57	-	Favored (76.66%) General / -59.8,-36.4	Favored (46.4%) *mt-10* chi angles: 303,191.3,322.8	0.11Å	-	-	-
A 645		ARG	0.49	-	Favored (63.14%) General / -70.0,-22.1	Favored (90.1%) *mtm-85* chi angles: 298.1,190.4,307,271.4	0.05Å	-	-	-

A 646		PHE	0.51	-	Favored (2.29%) General / -74.6,-60.4	OUTLIER (0.2%) chi angles: 166.9,12.3	0.10Å	-	OUTLIER(S) worst is CA-CB-CG: 7.8 σ	-
A 647		ASP	0.4	-	Favored (37.08%) General / -147.4,151.8	Favored (42.3%) *t0* chi angles: 186.2,13	0.06Å	-	-	-
A 648		GLU	0.41	-	Favored (7.6%) General / -83.4,179.7	Favored (63.5%) *mt-10* chi angles: 294.7,188.8,21.7	0.23Å	-	-	-
A 649		LEU	0.41	0.42Å HD21 with A 637 THR HG23	Favored (17.55%) Pre-Pro / -88.4,107.6	Favored (22.9%) *tp* chi angles: 173.8,53.4	0.12Å	-	-	-
A 650		PRO	0.56	-	Allowed (1.9%) Trans-Pro / -89.0,73.0	Favored (29.1%) *Cg_endo* chi angles: 35.3,327.6,17.3	0.11Å	-	-	-
**#**	**Alt**	**Res**	**High B**	**Clash > 0.4Å**	**Ramachandran**	**Rotamer**	**Cβ deviation**	**Bond lengths**	**Bond angles**	**Cis Peptides**
			Avg: 0.69	Clashscore: 1.84	Outliers: 9 of 654	Poor rotamers: 9 of 581	Outliers: 13 of 633	Outliers: 1 of 656	Outliers: 66 of 656	Non-Trans: 2 of 655
A 651		GLU	0.65	-	Allowed (0.26%) General / -147.7,-65.7	Favored (7.5%) *mm-30* chi angles: 294.8,285.5,86.6	0.08Å	-	-	-
A 652		GLN	0.66	-	Favored (75.2%) General / -55.7,-42.4	Favored (22.7%) *tt0* chi angles: 166.5,167,341.6	0.09Å	-	-	-
A 653		LEU	0.69	0.45Å HD13 with A 567 VAL HG11	Favored (49.61%) General / -77.7,-25.6	Favored (5.9%) *mt* chi angles: 307.3,158.5	0.08Å	-	-	-
A 654		ARG	0.68	-	Favored (83.82%) General / -67.7,-41.2	Favored (48.6%) *mtp180* chi angles: 295.9,163.7,83.2,186.8	0.06Å	-	-	-
A 655		VAL	0.76	-	Favored (41.57%) Ile or Val / -58.0,-53.7	Favored (70.1%) *t* chi angles: 178.7	0.04Å	-	-	-
A 656		ASN	0.71	-	Favored (81.19%) General / -66.2,-45.4	Favored (92.8%) *m-40* chi angles: 293.3,329.2	0.06Å	-	-	-
A 657		THR	0.71	-	Favored (83.71%) General / -61.3,-47.9	Favored (17.5%) *m* chi angles: 289.9	0.02Å	-	-	-
					Favored					

A 658		ALA	0.77	-	(76.11%) General / -69.1,-42.5	-	0.03Å	-	-	-
A 659		LEU	0.76	-	Favored (79.76%) General / -68.0,-36.3	Favored (81.4%) *mt* chi angles: 301.1,178.8	0.17Å	-	-	-
A 660		SER	0.76	-	Favored (76.68%) General / -58.0,-38.7	Favored (12.4%) *m* chi angles: 284.7	0.08Å	-	-	-
A 661		ASN	0.74	-	Favored (57.86%) General / -77.3,-18.1	Favored (90.5%) *m-40* chi angles: 294.5,328.7	0.06Å	-	OUTLIER(S) worst is CA-CB-CG: 4.1 σ	-
A 662		SER	0.78	-	Favored (17.41%) General / -85.7,107.0	Favored (9.7%) *t* chi angles: 191.2	0.07Å	-	-	-
A 663		GLN	0.72	-	Favored (81.01%) General / -61.4,-36.7	Favored (91.9%) *mt0* chi angles: 295,179.3,341.9	0.05Å	-	-	-
A 664		GLN	0.77	-	Favored (94.98%) General / -62.8,-39.7	Favored (93.8%) *mm-40* chi angles: 296.4,300.6,323.1	0.06Å	-	-	-
A 665		PHE	0.78	-	Favored (84.5%) General / -58.4,-47.2	Favored (73.1%) *t80* chi angles: 171.6,72.4	0.06Å	-	-	-
A 666		ALA	0.8	-	Favored (97.81%) General / -61.6,-41.7	-	0.02Å	-	-	-
A 667		ASN	0.78	-	Favored (96.69%) General / -62.7,-44.2	Favored (91.3%) *m-40* chi angles: 294.4,335.9	0.04Å	-	-	-
A 668		ALA	0.8	-	Favored (88.46%) General / -62.2,-38.3	-	0.02Å	-	-	-
A 669		TYR	0.78	-	Favored (36.7%) General / -103.2,11.7	Favored (35.1%) *m-80* chi angles: 278.9,96.2	0.03Å	-	-	-
A 670		GLY	0.81	-	Favored (54.44%) Glycine / 51.8,46.3	-	-	-	-	-
**#**	**Alt**	**Res**	**High B**	**Clash > 0.4Å**	**Ramachandran**	**Rotamer**	**Cβ deviation**	**Bond lengths**	**Bond angles**	**Cis Peptides**
							Outliers:			Non-

			Avg: 0.69	Clashscore: 1.84	Outliers: 9 of 654	Poor rotamers: 9 of 581	13 of 633	Outliers: 1 of 656	Outliers: 66 of 656	Trans: 2 of 655
A 671		CYS	0.8	-	Favored (29.43%) General / -80.8,124.9	Favored (69.1%) *m* chi angles: 298.8	0.06Å	-	-	-
A 672		SER	0.76	-	Favored (4.52%) General / -55.9,157.1	Favored (16.6%) *t* chi angles: 187.2	0.11Å	-	-	-
A 673		ARG	0.66	-	Favored (30.35%) General / -74.4,126.3	Favored (89.6%) *mtm180* chi angles: 295.3,177.9,293.6,171.2	0.06Å	-	-	-
A 674		GLU	0.63	-	Allowed (0.72%) General / 81.6,11.8	Favored (89.3%) *mt-10* chi angles: 300.1,175.2,345	0.36Å	-	-	-
A 675		GLU	0.66	-	Favored (8.97%) General / -58.1,156.8	Favored (59%) *mm-30* chi angles: 303.9,290.3,355.6	0.10Å	-	-	-
A 676		LYS	0.67	-	Favored (65.34%) General / -54.7,-37.1	Favored (82.4%) *tttt* chi angles: 182.4,173.4,172.5,183.8	0.08Å	-	-	-
A 677		LEU	0.71	-	Favored (38.55%) General / -96.8,10.6	Favored (55.5%) *mt* chi angles: 305.6,179.6	0.18Å	-	-	-
A 678		ASN	0.7	-	Allowed (1.89%) General / -137.2,53.3	Favored (47%) *t0* chi angles: 196.2,19.9	0.12Å	-	-	-
A 679		ALA	0.68	-	Favored (30.11%) General / -55.4,143.0	-	0.02Å	-	-	-
A 680		ARG	0.63	-	Favored (94.37%) General / -62.2,-45.2	Favored (69.3%) *ttm-80* chi angles: 179.2,185.8,300,271.8	0.07Å	-	-	-
A 681		PHE	0.66	-	Favored (29.77%) General / -84.5,123.0	Favored (86.8%) *m-80* chi angles: 290.5,94.4	0.06Å	-	OUTLIER(S) worst is CA-CB-CG: 4.4 σ	-
A 682		LYS	0.69	-	Favored (54.46%) General / -123.2,135.8	Favored (42%) *mttm* chi angles: 297.6,192.5,184.1,309.3	0.03Å	-	-	-
A 683		CYS	0.79	-	Favored (33.62%) General /	Favored (62.2%) *m* chi angles: 301	0.03Å	-	-	-

					-94.2,119.8					
A 684		THR	0.77	-	Favored (53.59%) General / -125.4,139.5	Favored (25.9%) *m* chi angles: 307.5	0.07Å	-	-	-
A 685		LEU	0.77	-	Allowed (0.58%) General / -127.6,-79.5	Favored (2.8%) *tt* chi angles: 192.2,140.5	0.14Å	-	-	-
A 686		TYR	0.75	-	-	Favored (22.7%) *m-80* chi angles: 277,110.1	0.04Å	-	-	-

B

Viewing model02FH-multi.table

Duke Biochemistry
Duke University School of Medicine

When finished, you should close this window .

Hint: Use File | Save As... to save a copy of this page.

All-Atom Contacts	Clashscore, all atoms:	9.96		71st percentile* (N=1784, all resolutions)
	Clashscore is the number of serious steric overlaps (> 0.4 Å) per 1000 atoms.			
Protein Geometry	Poor rotamers	8	1.38%	Goal: <0.3%
	Favored rotamers	560	96.39%	Goal: >98%
	Ramachandran outliers	7	1.07%	Goal: <0.05%
	Ramachandran favored	608	92.97%	Goal: >98%
	MolProbity score^	2.07		72nd percentile* (N=27675, 0Å - 99Å)
	Cβ deviations >0.25Å	7	1.11%	Goal: 0
	Bad bonds:	3 / 5624	0.05%	Goal: 0%
	Bad angles:	49 / 7620	0.64%	Goal: <0.1%
Peptide Omegas	Cis Prolines:	1 / 24	4.17%	Expected: ≤1 per chain, or ≤5%
	Cis nonProlines:	2 / 631	0.32%	Goal: <0.05%

In the two column results, the left column gives the raw count, right column gives the percentage.

* 100th percentile is the best among structures of comparable resolution; 0th percentile is the worst. For clashscore the comparative set of structures was selected in 2004, for MolProbity score in 2006.

^ MolProbity score combines the clashscore, rotamer, and Ramachandran evaluations into a single score, normalized to be on the same scale as X-ray resolution.

#	Alt	Res	High B	Clash > 0.4Å	Ramachandran	Rotamer	Cβ deviation	Bond lengths	Bond angles	Cis Peptides
			Avg: 0.70	Clashscore: 9.96	Outliers: 7 of 654	Poor rotamers: 8 of 581	Outliers: 7 of 633	Outliers: 2 of 656	Outliers: 48 of 656	Non-Trans: 3 of 655
A 31		ASN	0.57	-	-	Favored (41.1%) *p0*<br>chi angles: 62.2,355.3	0.07Å	-	-	-
A 32		TYR	0.65	-	Favored (41.2%)<br>General / -123.1,123.6	Favored (84.3%) *m-80*<br>chi angles: 302.7,96.3	0.11Å	-	-	-
A 33		SER	0.7	0.44Å<br>HB3 with A 38 ARG HG3	Favored (21.29%)<br>General / -67.5,123.8	Favored (6.8%) *m*<br>chi angles: 309.7	0.17Å	-	-	-
A 34		ILE	0.6	-	Favored (8.91%)<br>Ile or Val / -109.8,8.1	Favored (25.7%) *pt*<br>chi angles: 54.5,169.7	0.12Å	-	-	-

A 35		ASP	0.7	-	Favored (18.56%) General / -58.5,152.2	Favored (61%) *m-30* chi angles: 300.2,336.1	0.09Å	-	-	-
A 36		ILE	0.71	-	Favored (10.32%) Ile or Val / -47.6,-49.1	Favored (74.8%) *mt* chi angles: 289.5,170.9	0.09Å	-	-	-
A 37		LEU	0.74	0.74Å HD13 with A 505 ALA HB1	Favored (96.6%) General / -64.3,-41.8	Favored (37.2%) *mt* chi angles: 294.1,162.3	0.12Å	-	-	-
A 38		ARG	0.71	0.44Å HG3 with A 33 SER HB3	Favored (83.06%) General / -65.9,-45.1	Favored (63.7%) *mmt-90* chi angles: 296.3,279.2,191.4,281.1	0.04Å	-	-	-
A 39		LEU	0.77	-	Favored (89.34%) General / -66.5,-41.2	Favored (17.2%) *tp* chi angles: 190.4,55	0.09Å	-	-	-
A 40		ALA	0.78	-	Favored (76.59%) General / -56.8,-49.0	-	0.08Å	-	-	-
A 41		LYS	0.76	0.64Å HG2 with A 510 LEU HD13	Favored (80.38%) General / -68.7,-40.1	Favored (37.2%) *tptt* chi angles: 189.6,54.2,179.7,184.3	0.08Å	-	OUTLIER(S) worst is CB-CG-CD: 4.0 σ	-
A 42		ALA	0.81	-	Favored (98.93%) General / -62.1,-42.2	-	0.01Å	-	-	-
A 43		ALA	0.8	-	Favored (92.76%) General / -61.5,-45.8	-	0.05Å	-	-	-
A 44		HIS	0.76	-	Favored (3.98%) General / -53.8,-59.9	Favored (40.8%) *m-70* chi angles: 277.7,290.9	0.04Å	-	-	-
A 45		ILE	0.77	-	Favored (57.88%) Ile or Val / -53.3,-47.2	Favored (48.6%) *mt* chi angles: 285.5,166.8	0.02Å	-	-	-
A 46		LYS	0.76	-	Favored (97.64%) General / -60.7,-42.7	Favored (86.5%) *tttt* chi angles: 183.3,178.6,178.7,179	0.05Å	-	-	-
A 47		ALA	0.78	-	Favored (28.9%) General / -83.9,4.6	-	0.08Å	-	-	-
A		TYR	0.71	-	Favored (15.37%) General /	Favored (80.3%) *m-80*	0.03Å	-	-	-

48				-107.7,-11.7	chi angles: 293.4,105.9					
A 49		MET	0.75	-	Favored (11.22%) General / -92.3,-40.0	Favored (62.8%) *mtm* chi angles: 287,196.7,286.3	0.07Å	-	-	-
A 50		GLY	0.63	-	Favored (10.21%) Glycine / 118.2,-133.2	-	-	-	-	-
**#**	**Alt**	**Res**	**High B**	**Clash > 0.4Å**	**Ramachandran**	**Rotamer**	**Cβ deviation**	**Bond lengths**	**Bond angles**	**Cis Peptides**
			Avg: 0.70	Clashscore: 9.96	Outliers: 7 of 654	Poor rotamers: 8 of 581	Outliers: 7 of 633	Outliers: 2 of 656	Outliers: 48 of 656	Non-Trans: 3 of 655
A 51		ASP	0.71	-	Favored (47.24%) General / -97.2,7.1	Favored (68.9%) *m-30* chi angles: 299.8,317	0.22Å	-	OUTLIER(S) worst is CA-CB-CG: 5.3 σ	-
A 52		GLU	0.7	-	Favored (22.57%) General / 56.4,33.5	Favored (45.1%) *mt-10* chi angles: 295.5,173.6,48.9	0.12Å	-	-	-
A 53		LYS	0.69	-	Favored (59.29%) General / -84.0,-7.8	Favored (82.1%) *mttt* chi angles: 286.3,172.7,181.6,166.4	0.04Å	-	-	-
A 54		GLU	0.71	-	Favored (35.95%) General / -89.5,129.4	Favored (44.2%) *mm-30* chi angles: 300.4,286.8,319.1	0.12Å	-	-	-
A 55		GLN	0.7	-	Favored (29.93%) General / -66.0,126.9	Favored (75.6%) *tp40* chi angles: 181.2,70.1,45.9	0.03Å	-	-	-
A 56		ALA	0.78	-	Favored (65.84%) General / -54.8,-37.2	-	0.07Å	-	-	-
A 57		CYS	0.8	-	Favored (59.16%) General / -79.4,-14.1	Favored (32.1%) *m* chi angles: 306.5	0.03Å	-	-	-
A 58		ASN	0.74	-	Favored (15.92%) General / -91.5,-31.6	Favored (93.2%) *m-40* chi angles: 289.1,347.2	0.09Å	-	-	-
A 59		ASN	0.76	-	Allowed (0.32%) General / -169.5,97.6	Favored (13.2%) *t0* chi angles: 200,312.5	0.05Å	-	-	-
A 60		PHE	0.74	-	Favored (59.29%) General /	Favored (7.3%) *t80* chi angles: 205,74.2	0.09Å	-	-	-

#	Alt	Res	High B	Clash > 0.4Å	Ramachandran	Rotamer	Cβ deviation	Bond lengths	Bond angles	Cis Peptides
					-74.8,-25.9					
A 61		TYR	0.73	-	Favored (77.46%) General / -60.4,-49.5	Favored (64.2%) *t80* chi angles: 169.2,74.2	0.07Å	-	-	-
A 62		ASN	0.73	0.45Å O with A 66 ALA HB2	Favored (92%) General / -64.3,-44.3	Favored (88.2%) *m-40* chi angles: 293,346.5	0.08Å	-	-	-
A 63		TYR	0.72	-	Favored (81.32%) General / -62.0,-48.2	Favored (71.7%) *t80* chi angles: 173.1,84.6	0.07Å	-	-	-
A 64		ALA	0.78	-	Favored (80.48%) General / -66.7,-35.6	-	0.01Å	-	-	-
A 65		CYS	0.78	-	Favored (10.67%) General / -110.5,-24.3	Favored (42.2%) *m* chi angles: 304.3	0.07Å	-	-	-
A 66		ALA	0.76	0.45Å HB2 with A 62 ASN O	Favored (2.84%) General / -45.8,-33.1	-	0.07Å	-	-	-
A 67		ASN	0.73	-	Favored (94.08%) General / -59.8,-42.7	OUTLIER (0.3%) chi angles: 207.6,104.7	0.14Å	-	-	-
A 68		TRP	0.69	-	Favored (38.67%) Pre-Pro / -68.0,-44.3	Favored (53.6%) *t60* chi angles: 191.4,86.2	0.15Å	-	OUTLIER(S) worst is CA-C-N: 4.9 σ	-
A 69		PRO	0.71	-	Favored (88.05%) Trans-Pro / -59.1,-38.3	Favored (97.8%) *Cg_exo* chi angles: 332,33.9,332.1	0.04Å	-	-	-
A 70		ARG	0.68	-	Favored (77.86%) General / -69.4,-39.5	Favored (97.8%) *mtt180* chi angles: 292.3,181.5,180.6,173.2	0.10Å	-	-	-
**#**	**Alt**	**Res**	**High B**	**Clash > 0.4Å**	**Ramachandran**	**Rotamer**	**Cβ deviation**	**Bond lengths**	**Bond angles**	**Cis Peptides**
			Avg: 0.70	Clashscore: 9.96	Outliers: 7 of 654	Poor rotamers: 8 of 581	Outliers: 7 of 633	Outliers: 2 of 656	Outliers: 48 of 656	Non-Trans: 3 of 655
A 71		LEU	0.65	-	Favored (38.84%) General / -80.2,-28.5	Favored (40.4%) *mt* chi angles: 293.1,184.1	0.12Å	-	-	-
A 72		HIS	0.66	-	Favored (40.43%) Pre-Pro / -113.7,119.9	Favored (45.2%) *m-70* chi angles: 287.6,255.4	0.15Å	-	OUTLIER(S) worst is ND1-CG-CD2: 4.2 σ	-
					Favored	Favored (55.9%)				

A 73		PRO	0.67	-	(8.24%) Trans-Pro / -87.1,152.8	*Cg_endo* chi angles: 32.4,327.5,20	0.05Å	-	-	-
A 74		ALA	0.7	0.40Å HA with A 81 THR HG21	Favored (58.6%) General / -62.9,138.7	-	0.06Å	-	-	-
A 75		ARG	0.66	-	Favored (26.91%) General / -86.7,144.8	Favored (62.7%) *mtt90* chi angles: 300.9,181.9,190.9,95.5	0.06Å	-	-	-
A 76		LYS	0.61	-	Favored (65.01%) General / -59.3,-26.4	Favored (99.4%) *mttt* chi angles: 294.6,179.3,177.9,178.7	0.08Å	-	-	-
A 77		SER	0.53	-	Favored (62.61%) General / -71.0,-17.7	Favored (39%) *p* chi angles: 55.7	0.11Å	-	-	-
A 78		LYS	0.53	-	Favored (49.78%) General / -117.3,140.3	Favored (86.3%) *mttt* chi angles: 289.8,186.1,173.4,188.4	0.06Å	-	-	-
A 79		THR	0.68	0.53Å O with A 643 VAL HG11	Favored (58.06%) General / -88.9,-1.4	Favored (30.5%) *p* chi angles: 53.2	0.07Å	-	-	-
A 80		ARG	0.66	-	Favored (14.08%) General / -165.6,156.8	Favored (19.1%) *ptt180* chi angles: 58.1,204,179.4,180.5	0.03Å	-	-	-
A 81		THR	0.71	0.40Å HG21 with A 74 ALA HA	Favored (27.86%) General / -140.5,132.5	Favored (23.2%) *p* chi angles: 51.4	0.08Å	-	-	-
A 82		ASN	0.7	0.48Å HB3 with A 637 THR CB	OUTLIER (0.04)%) General / 172.6,-171.8	Favored (34.5%) *p0* chi angles: 68.4,331.9	0.20Å	-	OUTLIER(S) worst is CA-CB-CG: 7.9 σ	-
A 83		TYR	0.69	0.46Å CE1 with A 636 CYS HB2	Favored (8.39%) General / -45.0,-52.8	Favored (10%) *m-80* chi angles: 284.5,63.3	0.09Å	-	-	-
A 84		LEU	0.73	-	Favored (70.26%) General / -61.6,-51.0	Favored (57.6%) *tp* chi angles: 181,57.5	0.04Å	-	-	-
A 85		GLU	0.7	-	Favored (87.12%) General / -66.2,-38.1	Favored (40.8%) *mm-30* chi angles: 297.6,284.4,322.1	0.03Å	-	-	-
A 86		GLU	0.73	-	Favored (87.08%) General / -66.5,-42.7	Favored (4.3%) *mm-30* chi angles: 296.6,310.4,271.1	0.08Å	-	-	-

A 87		LEU	0.76	-	Favored (99.05%) General / -61.0,-43.2	Favored (69.7%) *mt* chi angles: 287.1,171.4	0.05Å	-	-	-
A 88		GLN	0.75	-	Favored (92.75%) General / -59.4,-44.5	Favored (93.4%) *mt0* chi angles: 290.8,178.6,346.4	0.07Å	-	-	-
A 89		GLU	0.75	-	Favored (99.37%) General / -61.2,-42.9	Favored (14.1%) *tm-30* chi angles: 197,281.5,321.4	0.07Å	-	-	-
A 90		LEU	0.77	-	Favored (85.97%) General / -61.9,-37.8	Favored (94.8%) *mt* chi angles: 294.9,172.1	0.03Å	-	-	-
**#**	**Alt**	**Res**	**High B**	**Clash > 0.4Å**	**Ramachandran**	**Rotamer**	**Cβ deviation**	**Bond lengths**	**Bond angles**	**Cis Peptides**
			Avg: 0.70	Clashscore: 9.96	Outliers: 7 of 654	Poor rotamers: 8 of 581	Outliers: 7 of 633	Outliers: 2 of 656	Outliers: 48 of 656	Non-Trans: 3 of 655
A 91		TYR	0.74	-	Favored (61.69%) General / -72.2,-19.7	Favored (24.7%) *m-10* chi angles: 298.4,340.1	0.12Å	-	-	-
A 92		ILE	0.76	-	Favored (24.37%) Ile or Val / -76.6,-34.6	Favored (37.2%) *mm* chi angles: 296.4,295.4	0.12Å	-	-	-
A 93		ARG	0.74	-	Favored (91.08%) General / -59.1,-45.4	Favored (91.2%) *mtt180* chi angles: 297.8,179.1,189,171.4	0.09Å	-	-	-
A 94		LYS	0.77	-	Favored (73.54%) General / -59.2,-35.4	Favored (51.1%) *pttt* chi angles: 63.2,176.7,186.8,189.6	0.06Å	-	-	-
A 95		SER	0.8	-	Favored (73.19%) General / -69.9,-42.5	Allowed (1.4%) *m* chi angles: 317.9	0.13Å	-	-	-
A 96		ALA	0.8	-	Favored (94.17%) General / -59.8,-44.3	-	0.11Å	-	-	-
A 97		ASP	0.77	-	Favored (97.05%) General / -61.2,-44.6	Favored (74.9%) *m-30* chi angles: 288.3,358.4	0.04Å	-	-	-
A 98		MET	0.76	-	Favored (74.43%) General / -69.9,-35.3	Favored (93.6%) *mmm* chi angles: 292.3,303.8,288.2	0.21Å	-	-	-
				0.48Å	Favored					

A 99		LEU	0.77	HD21 with A 365 LEU HD13	(21.38%) General / -88.6,-27.3	Favored (76.9%) *mt* chi angles: 290.9,176.3	0.04Å	-	-	-
A 100		LYS	0.73	-	Favored (87.54%) General / -63.4,-37.5	Favored (89.7%) *mttt* chi angles: 288.2,170.5,184.5,178.1	0.02Å	-	-	-
A 101		SER	0.73	-	Allowed (1.85%) General / -82.1,51.8	Favored (83.8%) *p* chi angles: 62.6	0.10Å	-	-	-
A 102		ALA	0.57	0.63Å HA with A 115 LYS HD3	Favored (67.87%) General / -63.8,-24.7	-	0.07Å	-	OUTLIER(S) worst is C-N-CA: 4.6 σ	-
A 103		THR	0.43	-	Favored (55.5%) General / -88.9,1.4	Favored (39.6%) *p* chi angles: 54.6	0.12Å	-	-	-
A 104		ARG	0.54	-	Favored (6.61%) General / -54.8,120.3	Favored (57.1%) *ttt-90* chi angles: 183.4,179,167.9,278.3	0.10Å	-	-	-
A 105		GLY	0.53	-	Favored (70.51%) Glycine / -66.2,-21.9	-	-	-	-	-
A 106		ALA	0.52	-	Favored (8.56%) General / -108.8,28.3	-	0.00Å	-	-	-
A 107		GLU	0.59	-	Favored (57.87%) General / -65.9,138.4	Favored (6%) *mm-30* chi angles: 296.7,296.4,264.5	0.04Å	-	-	-
A 108		ASN	0.67	-	Favored (22.19%) General / -75.0,167.3	Favored (19.6%) *p0* chi angles: 66.3,48.8	0.16Å	-	-	-
A 109		SER	0.76	-	Favored (69.86%) General / -59.0,-32.7	Favored (68.3%) *m* chi angles: 296.7	0.08Å	-	-	-
A 110		ALA	0.76	-	Favored (82.27%) General / -65.1,-46.1	-	0.05Å	-	-	-
**#**	**Alt**	**Res**	**High B**	**Clash > 0.4Å**	**Ramachandran**	**Rotamer**	**Cβ deviation**	**Bond lengths**	**Bond angles**	**Cis Peptides**
			Avg: 0.70	Clashscore: 9.96	Outliers: 7 of 654	Poor rotamers: 8 of 581	Outliers: 7 of 633	Outliers: 2 of 656	Outliers: 48 of 656	Non-Trans: 3 of 655
A					Favored (98.06%)	Favored (56.6%) *m-*				

111		ASP	0.74	-	General / -63.7,-41.8	*30* chi angles: 301.6,332.7	0.10Å	-	-	-
A 112		ARG	0.7	-	Favored (89.66%) General / -58.5,-45.1	Favored (87.1%) *mtp180* chi angles: 293,173.4,66.9,191.3	0.10Å	-	-	-
A 113		GLN	0.75	-	Favored (99.27%) General / -62.1,-43.2	Favored (89.6%) *mm-40* chi angles: 289.5,303.3,308	0.08Å	-	-	-
A 114		LEU	0.78	0.57Å HD13 with A 361 PHE HB2	Favored (83.36%) General / -58.2,-41.2	Favored (55.7%) *tp* chi angles: 182.3,59	0.17Å	-	-	-
A 115		LYS	0.75	0.63Å HD3 with A 102 ALA HA	Favored (30.47%) General / -83.4,-26.4	Favored (75.5%) *mttt* chi angles: 299.3,179.9,176.1,194.6	0.12Å	-	-	-
A 116		TYR	0.73	-	Favored (86.58%) General / -65.7,-37.5	Favored (11.4%) *m-10* chi angles: 292.3,357.5	0.10Å	-	-	-
A 117		PHE	0.77	-	Favored (91.91%) General / -60.2,-45.8	Favored (34.3%) *t80* chi angles: 190.9,97	0.11Å	-	-	-
A 118		TYR	0.76	-	Favored (96.68%) General / -60.9,-44.8	Favored (76.5%) *t80* chi angles: 179.3,70.3	0.06Å	-	-	-
A 119		GLY	0.79	-	Favored (23.33%) Glycine / -51.8,-54.9	-	-	-	-	-
A 120		SER	0.76	-	Favored (98.68%) General / -61.3,-42.5	Favored (85.1%) *p* chi angles: 69.8	0.13Å	-	-	-
A 121		CYS	0.78	-	Favored (87.8%) General / -60.3,-39.7	Favored (38.2%) *t* chi angles: 187.4	0.05Å	-	-	-
A 122		ARG	0.68	-	Favored (57.26%) General / -89.2,-6.0	Favored (9.5%) *ptm-80* chi angles: 69,200.5,298.9,275.5	0.03Å	-	-	-
A 123		LEU	0.7	-	Favored (7.38%) General / -81.2,68.3	Favored (95.5%) *mt* chi angles: 294.8,173.4	0.08Å	-	-	-
A 124		GLN	0.66	-	Favored (58.4%) General / -54.4,-33.1	Favored (80.4%) *mt0* chi angles: 295.2,185.3,336.4	0.03Å	-	-	-

#	Alt	Res	High B	Clash > 0.4Å	Ramachandran	Rotamer	Cβ deviation	Bond lengths	Bond angles	Cis Peptides
A 125		THR	0.68	-	Favored (80.22%) General / -57.3,-41.6	Favored (83.9%) *m* chi angles: 301.9	0.04Å	-	-	-
A 126		ASN	0.67	-	Favored (71.01%) General / -69.7,-44.2	Favored (98.6%) *m-40* chi angles: 290.5,339	0.07Å	-	-	-
A 127		ASP	0.66	-	Favored (93.34%) General / -62.3,-45.4	Favored (95.7%) *m-30* chi angles: 290.5,341.9	0.07Å	-	-	-
A 128		THR	0.67	0.43Å HG22 with A 344 PRO HD3	Favored (89.01%) General / -61.2,-39.1	Favored (22.4%) *p* chi angles: 51.2	0.14Å	-	-	-
A 129		ARG	0.64	-	Favored (65.79%) General / -65.8,-19.2	Favored (96.5%) *mtt180* chi angles: 289.7,171.8,183,177	0.05Å	-	-	-
A 130		SER	0.69	-	Favored (54.54%) General / -90.9,-6.1	Favored (8%) *p* chi angles: 83.3	0.18Å	-	-	-
**#**	**Alt**	**Res**	**High B**	**Clash > 0.4Å**	**Ramachandran**	**Rotamer**	**Cβ deviation**	**Bond lengths**	**Bond angles**	**Cis Peptides**
			Avg: 0.70	Clashscore: 9.96	Outliers: 7 of 654	Poor rotamers: 8 of 581	Outliers: 7 of 633	Outliers: 2 of 656	Outliers: 48 of 656	Non-Trans: 3 of 655
A 131		ALA	0.68	-	Allowed (1.92%) General / 50.0,-123.0	-	0.10Å	-	-	-
A 132		LEU	0.68	0.53Å HD13 with A 230 TYR CE2	Favored (18.06%) General / -112.3,19.0	Favored (8.7%) *mp* chi angles: 277.7,62.1	0.09Å	-	-	-
A 133		ASN	0.68	-	Favored (73.13%) General / -65.2,-31.8	Favored (84.9%) *m-40* chi angles: 296.6,332.2	0.07Å	-	-	-
A 134		THR	0.71	0.51Å HB with A 448 TYR CD2	Favored (83.6%) General / -67.9,-39.5	Favored (49.4%) *p* chi angles: 56.1	0.05Å	-	-	-
A 135		LEU	0.7	0.59Å HD21 with A 335 LEU CD2	Favored (94.04%) General / -64.8,-43.2	OUTLIER (0%) chi angles: 215.6,212.4	0.04Å	-	-	-
A 136		GLN	0.65	0.45Å CB with A 231 PHE CE1	Favored (88.76%) General / -58.4,-43.6	Favored (2.6%) *pt0* chi angles: 87.1,200.5,359.1	0.09Å	-	-	-

A 137		ASN	0.68	-	Favored (83.11%) General / -60.9,-37.7	Favored (88.6%) *m-40* chi angles: 294.9,339.2	0.08Å	-	-	-
A 138		VAL	0.74	-	Favored (62.75%) Ile or Val / -70.0,-35.2	Favored (33.8%) *t* chi angles: 165.8	0.07Å	-	-	-
A 139		THR	0.72	-	Favored (80.3%) General / -57.5,-41.2	Favored (10.3%) *m* chi angles: 288.1	0.14Å	-	-	-
A 140		ASP	0.74	-	Favored (75.03%) General / -60.3,-35.2	Favored (85%) *m-30* chi angles: 291.5,333.5	0.04Å	-	-	-
A 141		PHE	0.65	-	Favored (62.22%) General / -69.4,-13.2	Favored (64.8%) *m-80* chi angles: 285.9,96.6	0.08Å	-	OUTLIER(S) worst is CA-CB-CG: 4.1 σ	-
A 142		ARG	0.68	0.50Å HH21 with A 171 LEU HD13	Favored (25.87%) General / -109.1,8.2	Favored (19.9%) *mtm-85* chi angles: 292.8,214.7,282.3,269.5	0.06Å	-	-	-
A 143		GLY	0.75	0.49Å HA2 with A 158 TYR CZ	Favored (76.72%) Glycine / 84.4,12.0	-	-	-	-	-
A 144		GLY	0.77	-	Favored (23.16%) Glycine / 70.7,177.2	-	-	-	-	-
A 145		TRP	0.67	0.48Å CH2 with A 228 LYS HG3	Favored (32.77%) Pre-Pro / -115.6,122.5	Favored (14.4%) *t60* chi angles: 189.7,26.8	0.04Å	-	-	-
A 146		PRO	0.69	0.46Å HG3 with A 153 TRP CE2	Favored (18.43%) Trans-Pro / -71.5,-27.8	Favored (89.3%) *Cg_exo* chi angles: 329.8,24.8,350.3	0.12Å	-	-	-
A 147		GLU	0.57	0.52Å H with A 328 ASN HD21	Favored (90.06%) General / -58.8,-42.9	Favored (2.3%) *tp30* chi angles: 181.1,82.2,81.6	0.24Å	-	-	-
A 148		ILE	0.58	0.73Å HB with A 232 PRO HD2	Allowed (0.96%) Ile or Val / -98.8,24.5	Favored (9.5%) *pt* chi angles: 67.1,156.9	0.18Å	-	-	-
A 149		ARG	0.57	-	Favored (8.6%) General / -131.7,107.1	Favored (72.6%) *ttm-80* chi angles: 186.1,182.7,295.9,268.1	0.08Å	-	-	-
A 150		VAL	0.56	0.41Å HB with A 151 ALA HA	Favored (14.86%) Ile or Val /	Favored (12.4%) *m* chi angles: 305.5	0.04Å	-	-	-

#	Alt	Res	High B	Clash > 0.4Å	Ramachandran	Rotamer	Cβ deviation	Bond lengths	Bond angles	Cis Peptides
					-115.2,159.9					
			Avg: 0.70	Clashscore: 9.96	Outliers: 7 of 654	Poor rotamers: 8 of 581	Outliers: 7 of 633	Outliers: 2 of 656	Outliers: 48 of 656	Non-Trans: 3 of 655
A 151		ALA	0.56	0.41Å HA with A 150 VAL HB	Favored (58.98%) General / -86.5,-6.1	-	0.09Å	-	-	Cis nonPRO omega= 5.88
A 152		SER	0.51	-	Allowed (1.55%) General / -159.3,101.6	Favored (22.8%) *t* chi angles: 185.2	0.08Å	-	-	-
A 153		TRP	0.57	0.46Å CE2 with A 146 PRO HG3	Favored (89.03%) General / -63.7,-37.9	Favored (90.7%) *t60* chi angles: 177.4,87.4	0.11Å	-	-	-
A 154		TYR	0.56	-	Allowed (0.15%) General / -58.0,-2.2	Favored (35.1%) *m-80* chi angles: 286.1,119.8	0.03Å	-	-	-
A 155		GLN	0.5	-	Favored (86.47%) General / -61.8,-38.0	Favored (92.7%) *tp40* chi angles: 186.1,66.9,54.4	0.10Å	-	-	-
A 156		TYR	0.47	-	Favored (39.51%) General / -122.1,122.6	Favored (97.3%) *m-80* chi angles: 294.5,95.9	0.06Å	-	-	-
A 157		GLU	0.53	-	Favored (33.55%) General / -81.1,142.2	Favored (85.7%) *mt-10* chi angles: 294.4,189.2,0.3	0.05Å	-	-	-
A 158		TYR	0.69	0.49Å CZ with A 143 GLY HA2	Favored (13.8%) General / -131.9,113.8	Favored (98.3%) *m-80* chi angles: 298,96.7	0.04Å	-	-	-
A 159		ASP	0.71	-	Favored (45.66%) General / -71.3,150.0	Favored (87.7%) *m-30* chi angles: 289.2,336.8	0.04Å	-	-	-
A 160		TRP	0.71	0.41Å O with A 164 VAL HG23	Favored (86.16%) General / -58.3,-42.3	Favored (53.2%) *m-10* chi angles: 301.3,343.6	0.04Å	-	-	-
A 161		LEU	0.73	0.91Å HD22 with A 290 TYR HB2	Favored (95.17%) General / -60.6,-41.6	Favored (31.9%) *mt* chi angles: 306.2,171	0.03Å	-	-	-
A 162		GLN	0.72	-	Favored (63.01%) General / -70.7,-46.4	Favored (96.1%) *mt0* chi angles: 294.1,176.2,318.9	0.04Å	-	-	-

A 163		VAL	0.76	-	Favored (95.46%) Ile or Val / -65.2,-43.1	Favored (7.4%) *p* chi angles: 68.4	0.15Å	-	-	-
A 164		VAL	0.78	0.41Å HG23 with A 160 TRP O	Favored (90.7%) Ile or Val / -66.4,-42.5	Favored (25.1%) *t* chi angles: 186.2	0.04Å	-	-	-
A 165		ALA	0.77	-	Favored (99.94%) General / -63.0,-43.0	-	0.02Å	-	-	-
A 166		ASN	0.75	-	Favored (85.59%) General / -57.7,-45.6	Favored (89.7%) *m-40* chi angles: 286.1,332.9	0.11Å	-	-	-
A 167		LEU	0.76	0.47Å HD21 with A 142 ARG HD2	Favored (81.11%) General / -63.9,-47.3	Favored (2.2%) *mt* chi angles: 266,163.2	0.09Å	-	-	-
A 168		LYS	0.77	-	Favored (46.86%) General / -65.1,-53.4	Favored (7.3%) *ttmm* chi angles: 183.6,159.4,286.3,290	0.17Å	-	-	-
A 169		ARG	0.71	0.42Å HD3 with A 463 LEU CD1	Favored (69.48%) General / -71.9,-35.0	Favored (43.9%) *tpt90* chi angles: 171.4,55.2,181.3,94.4	0.03Å	-	-	-
A 170		LYS	0.73	-	Favored (15.19%) General / -96.6,-26.6	Favored (41.3%) *mmtt* chi angles: 315.7,297,167.4,176.7	0.03Å	-	-	-
**#**	**Alt**	**Res**	**High B**	**Clash > 0.4Å**	**Ramachandran**	**Rotamer**	**Cβ deviation**	**Bond lengths**	**Bond angles**	**Cis Peptides**
			Avg: 0.70	Clashscore: 9.96	Outliers: 7 of 654	Poor rotamers: 8 of 581	Outliers: 7 of 633	Outliers: 2 of 656	Outliers: 48 of 656	Non-Trans: 3 of 655
A 171		LEU	0.75	0.50Å HD13 with A 142 ARG HH21	Favored (3.59%) General / -132.6,0.0	Favored (12.9%) *mt* chi angles: 293.3,155.4	0.03Å	-	-	-
A 172		GLY	0.8	-	Favored (80%) Glycine / 74.7,18.1	-	-	-	-	-
A 173		VAL	0.78	-	Favored (18.29%) Ile or Val / -97.3,106.3	Favored (82.6%) *t* chi angles: 176.8	0.03Å	-	-	-
A 174		ASP	0.77	-	Favored (19.23%) General / -90.6,107.5	Favored (45.3%) *m-30* chi angles: 296.4,356	0.04Å	-	-	-
A		ILE	0.76	-	Favored (50.91%)	Allowed (1.3%) *pp*	0.04Å	-	-	-

175					Ile or Val / -124.4,139.4	chi angles: 57.6,94.9				
A 176		PHE	0.76	-	Allowed (0.46%) General / 73.5,-62.6	Favored (23.3%) *m-10* chi angles: 295.4,328.2	0.20Å	-	-	-
A 177		ILE	0.75	-	Favored (18.52%) Ile or Val / -126.8,108.2	Favored (61.5%) *mt* chi angles: 304.1,170	0.06Å	-	-	-
A 178		GLY	0.79	-	Favored (3.59%) Glycine / -84.7,68.0	-	-	-	OUTLIER(S) worst is C-N-CA: 4.3 σ	-
A 179		LEU	0.77	0.64Å HD21 with A 291 LEU CD2	Favored (32.83%) General / -75.7,127.3	OUTLIER (0%) chi angles: 178,321.1	0.20Å	-	OUTLIER(S) worst is N-CA-CB: 4.6 σ	-
A 180		GLU	0.76	-	Favored (31.38%) General / -155.8,154.4	Favored (4.6%) *tp30* chi angles: 185.4,54,107.5	0.09Å	-	-	-
A 181		VAL	0.77	-	Favored (36.02%) Ile or Val / -89.2,119.3	Favored (77.7%) *t* chi angles: 172.9	0.03Å	-	-	-
A 182		ILE	0.73	-	Favored (7.95%) Ile or Val / -138.6,175.6	Favored (14.6%) *pt* chi angles: 57.5,159	0.06Å	-	-	-
A 183		LEU	0.71	-	Favored (57.51%) General / -58.4,136.6	Favored (74.8%) *mt* chi angles: 301.7,175	0.05Å	-	-	-
A 184		ASP	0.72	-	Favored (18.83%) General / -68.3,122.7	Favored (49.3%) *t0* chi angles: 189.6,152.2	0.05Å	-	-	-
A 185		TYR	0.66	-	Favored (71.72%) General / -60.8,-32.4	Favored (24.6%) *m-80* chi angles: 296.6,137.7	0.05Å	-	-	-
A 186		LYS	0.65	-	Favored (33.48%) General / -104.8,6.0	Favored (85.3%) *mttt* chi angles: 289.6,191.5,174.8,183.7	0.08Å	-	-	-
A 187		GLU	0.69	-	Allowed (1.85%) General / -147.5,90.3	Favored (59%) *tt0* chi angles: 190.4,178,20.8	0.08Å	-	-	-
A 188		GLU	0.7	-	Favored (58.35%) General / -51.0,-40.7	Favored (5.1%) *tm-30* chi angles: 188,277,304.8	0.14Å	-	OUTLIER(S) worst is CB-CG-CD: 5.5 σ	-

#	Alt	Res	High B	Clash > 0.4Å	Ramachandran	Rotamer	Cβ deviation	Bond lengths	Bond angles	Cis Peptides
A 189		LYS	0.7	-	Allowed (1.68%) General / -84.8,20.9	Favored (59.5%) *mmtt* chi angles: 298,292.8,180,196.4	0.09Å	-	-	-
A 190		MET	0.7	-	Favored (48.67%) General / -135.8,156.5	Favored (94.2%) *mmm* chi angles: 290.6,298,287.6	0.09Å	-	-	-
#	Alt	Res	High B	Clash > 0.4Å	Ramachandran	Rotamer	Cβ deviation	Bond lengths	Bond angles	Cis Peptides
			Avg: 0.70	Clashscore: 9.96	Outliers: 7 of 654	Poor rotamers: 8 of 581	Outliers: 7 of 633	Outliers: 2 of 656	Outliers: 48 of 656	Non-Trans: 3 of 655
A 191		HIS	0.73	-	Favored (18.4%) General / -97.7,153.2	Favored (78.6%) *m90* chi angles: 296.8,86.1	0.06Å	-	-	-
A 192		ARG	0.75	-	Favored (21.64%) General / -143.8,167.7	Favored (87.6%) *mmt-90* chi angles: 290.4,285.1,186.3,271.4	0.12Å	-	-	-
A 193		LEU	0.78	0.56Å HD21 with A 291 LEU HD22	Favored (33.63%) General / -85.4,136.0	Favored (27.7%) *tp* chi angles: 185,53.9	0.06Å	-	-	-
A 194		LYS	0.77	-	Favored (43.58%) General / -115.0,144.4	Favored (31.1%) *ttpt* chi angles: 170.1,176.7,64.9,180.8	0.07Å	-	-	-
A 195		ILE	0.77	-	Favored (64.82%) Ile or Val / -114.9,132.2	Favored (53.6%) *mt* chi angles: 294.7,179.3	0.07Å	-	-	-
A 196		GLY	0.77	-	Favored (22.18%) Glycine / -136.1,172.8	-	-	-	-	-
A 197		ALA	0.77	-	Favored (35.15%) Pre-Pro / -88.2,145.1	-	0.03Å	-	-	-
A 198		PRO	0.76	-	Favored (26.98%) Trans-Pro / -79.0,157.1	Favored (24.1%) *Cg_endo* chi angles: 36.2,321.8,25.4	0.06Å	-	-	-
A 199		GLN	0.64	-	Favored (51.33%) General / -66.8,148.9	Favored (96.6%) *mm-40* chi angles: 299.8,296.3,309.8	0.10Å	-	-	-
A 200		PHE	0.69	-	Favored (33.26%) Pre-Pro / -93.3,160.4	Favored (22.8%) *m-80* chi angles: 313.7,113.8	0.10Å	-	-	-
					Favored	Favored (69.2%)				

A 201		PRO	0.75	-	(3.35%) Trans-Pro / -69.7,-42.5	*Cg_exo* chi angles: 335.4,31,332.8	0.13Å	-	-	-
A 202		MET	0.71	-	Favored (37.65%) General / -75.7,151.8	Favored (63.9%) *mtt* chi angles: 300,181.9,181.4	0.05Å	-	-	-
A 203		SER	0.66	-	Favored (18.02%) General / -46.6,-44.4	Favored (8.3%) *t* chi angles: 165	0.15Å	-	-	-
A 204		ARG	0.66	-	Favored (35.81%) General / -159.2,160.7	Favored (37.2%) *ptt180* chi angles: 60.1,179.4,189,185.4	0.04Å	-	-	-
A 205		ARG	0.68	-	Favored (36.93%) General / -54.5,-27.3	Favored (10.6%) *ptm-80* chi angles: 66.6,189.5,303.2,286.7	0.06Å	-	-	-
A 206		HIS	0.68	-	Favored (64.39%) General / -58.0,-28.3	Favored (54%) *m-70* chi angles: 281.1,278.3	0.09Å	-	-	-
A 207		TYR	0.68	-	Favored (63.09%) General / -68.9,-14.5	Favored (9.8%) *m-80* chi angles: 269.8,82.3	0.03Å	-	-	-
A 208		LEU	0.71	0.52Å CD1 with A 254 ALA HB3	Favored (46.27%) General / -94.4,-5.5	Favored (22.9%) *mt* chi angles: 295.9,160.2	0.21Å	-	-	-
A 209		HIS	0.65	-	Favored (77.62%) Pre-Pro / -78.9,153.2	Favored (96.9%) *m-70* chi angles: 295.3,289.9	0.04Å	-	OUTLIER(S) worst is ND1-CG-CD2: 4.3 σ	-
A 210		PRO	0.49	-	Favored (4.94%) Trans-Pro / -62.5,-4.0	Favored (91.6%) *Cg_exo* chi angles: 331.1,35.5,331.3	0.04Å	-	-	-
**#**	**Alt**	**Res**	**High B**	**Clash > 0.4Å**	**Ramachandran**	**Rotamer**	**Cβ deviation**	**Bond lengths**	**Bond angles**	**Cis Peptides**
			Avg: 0.70	Clashscore: 9.96	Outliers: 7 of 654	Poor rotamers: 8 of 581	Outliers: 7 of 633	Outliers: 2 of 656	Outliers: 48 of 656	Non-Trans: 3 of 655
A 211		HIS	0.46	-	Favored (88.73%) General / -59.3,-41.3	Favored (81%) *t70* chi angles: 184.7,75	0.07Å	-	OUTLIER(S) worst is ND1-CG-CD2: 4.2 σ	-
A 212		PHE	0.57	-	Allowed (0.81%) General / -103.5,64.1	Favored (55.8%) *m-80* chi angles: 287.5,110.6	0.04Å	-	-	-
A		GLU	0.65	-	Favored (83.68%)	Favored (9.2%) *tp30* chi angles:	0.08Å	-	-	-

213					General / -62.8,-47.4	181.7,52.7,357.3				
A 214		GLY	0.73	-	Favored (91.06%) Glycine / -62.4,-35.0	-	-	-	-	-
A 215		THR	0.7	-	Favored (92.64%) General / -64.6,-43.8	Favored (81.1%) *m* chi angles: 302.4	0.06Å	-	-	-
A 216		ARG	0.71	-	Favored (93.8%) General / -63.8,-44.3	Favored (57.5%) *ttp80* chi angles: 183.8,203.6,64.7,76.4	0.02Å	-	-	-
A 217		GLU	0.72	-	Favored (81.63%) General / -62.0,-48.1	Favored (66.3%) *tt0* chi angles: 185.9,185,16.1	0.04Å	-	-	-
A 218		ILE	0.75	-	Favored (84.6%) Ile or Val / -61.2,-40.1	Favored (68.6%) *mt* chi angles: 288.4,165.3	0.03Å	-	-	-
A 219		TYR	0.72	-	Favored (78.14%) General / -60.9,-49.3	Favored (38.9%) *t80* chi angles: 181,237.6	0.09Å	-	-	-
A 220		GLU	0.73	-	Favored (91.03%) General / -66.0,-40.3	Favored (60.3%) *tt0* chi angles: 191.1,167.9,9.3	0.07Å	-	-	-
A 221		ARG	0.7	-	Favored (93.92%) General / -61.7,-40.1	Favored (84.9%) *mtt-85* chi angles: 292.4,188,185.4,288.1	0.07Å	-	-	-
A 222		SER	0.77	-	Favored (96.72%) General / -60.3,-43.0	Favored (10.8%) *t* chi angles: 189.8	0.06Å	-	-	-
A 223		ILE	0.7	0.50Å HG12 with A 332 TRP CE2	Favored (55.77%) Ile or Val / -56.5,-52.2	Favored (79.3%) *mt* chi angles: 290.2,171.1	0.01Å	-	-	-
A 224		GLU	0.65	-	Favored (75.49%) General / -64.1,-48.6	Favored (55.5%) *tt0* chi angles: 181.4,192.6,3.9	0.06Å	-	-	-
A 225		ASN	0.7	-	Favored (21.76%) General / -147.2,135.7	Favored (12.4%) *p0* chi angles: 69.1,84.8	0.13Å	-	-	-
A 226		LYS	0.53	-	Favored (52.56%) General / -86.7,-10.9	Favored (95.3%) *mttt* chi angles: 287.4,183.4,182.8,178.3	0.09Å	-	-	Cis nonPRO omega= 9.41
					Favored					

A 227		LEU	0.64	0.63Å HD13 with A 332 TRP CZ3	(83.31%) General / -63.9,-46.8	Favored (2.5%) *mt* chi angles: 284.6,145.7	0.10Å	-	-	-
A 228		LYS	0.66	0.48Å HG3 with A 145 TRP CH2	Favored (9.72%) General / -75.7,-52.8	Favored (54.9%) *mttt* chi angles: 292.4,161.8,165.7,185.9	0.03Å	-	-	-
A 229		LEU	0.58	-	Favored (68.01%) General / -64.8,-25.7	Favored (31.7%) *mt* chi angles: 291.3,184.5	0.06Å	-	-	-
A 230		TYR	0.52	0.53Å CE2 with A 132 LEU HD13	Allowed (1.11%) General / -84.4,-64.8	Allowed (0.4%) *t80* chi angles: 192.5,141.7	0.08Å	-	-	-
**#**	**Alt**	**Res**	**High B**	**Clash > 0.4Å**	**Ramachandran**	**Rotamer**	**Cβ deviation**	**Bond lengths**	**Bond angles**	**Cis Peptides**
			Avg: 0.70	Clashscore: 9.96	Outliers: 7 of 654	Poor rotamers: 8 of 581	Outliers: 7 of 633	Outliers: 2 of 656	Outliers: 48 of 656	Non-Trans: 3 of 655
A 231		PHE	0.57	0.45Å CE1 with A 136 GLN CB	Favored (28.91%) Pre-Pro / -114.6,125.6	Favored (90.9%) *t80* chi angles: 175.4,78	0.17Å	-	OUTLIER(S) worst is N-CA-CB: 4.1 σ	-
A 232		PRO	0.52	0.73Å HD2 with A 148 ILE HB	Favored (29.55%) Cis-Pro / -98.8,3.9	Favored (55.5%) *Cg_endo* chi angles: 25.9,322.6,38.2	0.23Å	OUTLIER(S) worst is N--CD: 5.2 σ	OUTLIER(S) worst is N-CD-CG: 4.0 σ	Cis PRO omega= -1.04
A 233		GLU	0.5	-	Favored (27.33%) General / -53.3,-55.1	Favored (21%) *mm-30* chi angles: 288.8,287.2,284.7	0.05Å	-	-	-
A 234		GLN	0.49	-	Favored (14.24%) General / -87.2,-40.8	Favored (62.8%) *tp40* chi angles: 190.6,64.3,67.9	0.09Å	-	-	-
A 235		SER	0.53	-	Favored (63.68%) General / -74.2,-34.5	Favored (19.2%) *p* chi angles: 52.7	0.20Å	-	-	-
A 236		GLU	0.47	0.48Å HG3 with A 232 PRO HB3	Favored (34.57%) General / -93.3,9.8	Favored (7.5%) *mm-30* chi angles: 298.7,268.1,205.8	0.17Å	-	-	-
A 237		HIS	0.47	-	Allowed (0.94%) General / 69.3,44.5	Favored (68.2%) *m-70* chi angles: 308,276.7	0.08Å	-	OUTLIER(S) worst is ND1-CG-CD2: 4.2 σ	-
A 238		TRP	0.46	-	Favored (11.85%) General / -96.8,166.0	Favored (16.9%) *m100* chi angles: 296.3,130.7	0.11Å	-	-	-
					Favored	Favored (78.9%)				

A 239		ARG	0.45	-	(18.08%) General / -79.0,114.7	*ttt180* chi angles: 180.9,181.8,180.7,176.6	0.06Å	-	-	-
A 240		GLN	0.43	-	OUTLIER (0.04)%) General / -56.2,100.5	Favored (18.3%) *mm110* chi angles: 310.8,295.8,129	0.09Å	-	-	-
A 241		GLU	0.38	-	Favored (14.76%) General / -153.5,135.6	Favored (83.1%) *tt0* chi angles: 186.1,171,354.1	0.06Å	-	-	-
A 242		VAL	0.37	-	Favored (42.21%) Ile or Val / -134.3,123.0	Favored (18.2%) *t* chi angles: 164.3	0.08Å	-	-	-
A 243		ALA	0.4	-	Allowed (1.54%) General / -159.1,100.4	-	0.01Å	-	-	-
A 244		SER	0.44	-	Favored (12.19%) General / -143.9,122.0	Favored (46.5%) *m* chi angles: 290.7	0.13Å	-	-	-
A 245		GLN	0.63	-	Favored (5.72%) General / -74.6,105.5	Favored (60.4%) *tt0* chi angles: 178.7,182,40.4	0.11Å	-	-	-
A 246		VAL	0.76	-	Allowed (1.11%) Ile or Val / -125.3,-40.8	Favored (75.3%) *t* chi angles: 172.7	0.06Å	-	-	-
A 247		VAL	0.76	-	Favored (92.58%) Ile or Val / -61.2,-42.2	Favored (69.2%) *t* chi angles: 178.9	0.08Å	-	-	-
A 248		GLN	0.72	-	Favored (99.58%) General / -61.8,-43.1	Favored (86.3%) *tp40* chi angles: 186.2,69.6,53.2	0.06Å	-	-	-
A 249		ILE	0.75	0.61Å HD11 with A 325 GLN HB3	Favored (87.37%) Ile or Val / -60.3,-48.7	Favored (66.4%) *mt* chi angles: 290.2,174.5	0.16Å	-	-	-
A 250		GLU	0.75	-	Favored (83.98%) General / -67.6,-38.4	Favored (14.5%) *tt0* chi angles: 192.1,162.1,60.4	0.16Å	-	OUTLIER(S) worst is CA-CB-CG: 4.7 σ	-
**#**	**Alt**	**Res**	**High B**	**Clash > 0.4Å**	**Ramachandran**	**Rotamer**	**Cβ deviation**	**Bond lengths**	**Bond angles**	**Cis Peptides**
			Avg: 0.70	Clashscore: 9.96	Outliers: 7 of 654	Poor rotamers: 8 of 581	Outliers: 7 of 633	Outliers: 2 of 656	Outliers: 48 of 656	Non-Trans: 3 of 655
A		GLN	0.75	-	Favored (69.83%)	Favored (41%) *tt0* chi angles:	0.05Å	-	-	-

251					General / -55.2,-39.5	188.4,190.1,17.8				
A 252		GLN	0.76	-	Favored (87.65%) General / -65.5,-44.3	Favored (78.3%) *mt0* chi angles: 297.7,174.5,297.2	0.06Å	-	-	-
A 253		LEU	0.77	-	Favored (93.57%) General / -61.5,-40.1	Favored (95.7%) *mt* chi angles: 295.3,172.6	0.05Å	-	-	-
A 254		ALA	0.78	0.52Å HB3 with A 208 LEU CD1	Favored (53.6%) General / -78.7,-5.6	-	0.11Å	-	-	-
A 255		LYS	0.75	-	Allowed (0.44%) General / -73.1,56.5	Favored (93.2%) *mttt* chi angles: 294.9,173.2,186.5,183.4	0.06Å	-	-	-
A 256		GLY	0.7	-	Favored (11.09%) Glycine / -117.0,-158.2	-	-	-	-	-
A 257		LEU	0.65	-	Favored (85.55%) Pre-Pro / -74.3,147.8	Favored (68.1%) *mt* chi angles: 297.5,168.7	0.05Å	-	-	-
A 258		PRO	0.68	-	Favored (2.28%) Trans-Pro / -75.1,-175.2	Favored (36%) *Cg_exo* chi angles: 338.9,33.7,326.6	0.08Å	-	-	-
A 259		HIS	0.58	-	Favored (76.77%) General / -69.1,-35.8	Favored (99.5%) *m-70* chi angles: 298.1,289.8	0.11Å	-	OUTLIER(S) worst is ND1-CG-CD2: 4.2 σ	-
A 260		ASN	0.63	-	Favored (12.12%) Pre-Pro / -47.1,-38.1	Favored (89.2%) *m-40* chi angles: 295.2,321.8	0.12Å	-	OUTLIER(S) worst is CA-C-N: 5.4 σ	-
A 261		PRO	0.64	-	Favored (4.82%) Trans-Pro / -88.2,6.7	Favored (99.2%) *Cg_exo* chi angles: 332.3,40.9,321.1	0.05Å	-	-	-
A 262		ALA	0.63	-	Favored (17.12%) General / -113.6,6.7	-	0.04Å	-	-	-
A 263		LEU	0.63	-	Favored (58.18%) General / -89.5,-3.9	Favored (95.7%) *mt* chi angles: 294.9,173.1	0.06Å	-	-	-
A 264		THR	0.68	-	Favored (12.09%) General / -102.3,101.8	Favored (50.9%) *m* chi angles: 294.6	0.08Å	-	-	-

#	Alt	Res	High B	Clash > 0.4Å	Ramachandran	Rotamer	Cβ deviation	Bond lengths	Bond angles	Cis Peptides
A 265		LEU	0.65	0.75Å HD11 with A 530 VAL HG13	Favored (73.83%) General / -58.8,-36.1	Favored (33.4%) *mt* chi angles: 290.8,160.3	0.20Å	-	-	-
A 266		ALA	0.68	-	Favored (88.98%) General / -62.5,-38.2	-	0.03Å	-	-	-
A 267		GLN	0.64	-	Favored (62.47%) General / -74.0,-41.0	Favored (95.7%) *mt0* chi angles: 295.1,173.8,325	0.09Å	-	-	-
A 268		THR	0.64	-	Favored (72.83%) General / -62.7,-31.9	Favored (80.3%) *p* chi angles: 60.5	0.13Å	-	-	-
A 269		THR	0.69	-	Favored (2.58%) General / -73.5,97.6	Favored (34.1%) *m* chi angles: 293.1	0.07Å	-	OUTLIER(S) worst is OG1-CB-CG2: 4.0 σ	-
A 270		ARG	0.69	-	Favored (8.84%) General / -118.9,102.5	Favored (24.4%) *mmm160* chi angles: 308.3,300.6,299.4,155.4	0.03Å	-	-	-
**#**	**Alt**	**Res**	**High B**	**Clash > 0.4Å**	**Ramachandran**	**Rotamer**	**Cβ deviation**	**Bond lengths**	**Bond angles**	**Cis Peptides**
			Avg: 0.70	Clashscore: 9.96	Outliers: 7 of 654	Poor rotamers: 8 of 581	Outliers: 7 of 633	Outliers: 2 of 656	Outliers: 48 of 656	Non-Trans: 3 of 655
A 271		GLN	0.73	-	Favored (36.16%) General / -80.0,133.1	Favored (57.4%) *tt0* chi angles: 190.3,174.6,9.1	0.07Å	-	-	-
A 272		ARG	0.76	0.40Å NH1 with A 308 GLU OE2	Favored (44.25%) General / -144.6,157.4	Favored (87.9%) *mmt-90* chi angles: 290,295.3,182.4,276.5	0.15Å	-	-	-
A 273		THR	0.8	-	Favored (24.41%) General / -81.9,157.2	Favored (22.8%) *p* chi angles: 71.5	0.13Å	-	-	-
A 274		ALA	0.79	-	Favored (63.99%) General / -57.6,-28.9	-	0.05Å	-	-	-
A 275		ALA	0.78	-	Favored (85.99%) General / -63.8,-46.2	-	0.03Å	-	-	-
A 276		GLU	0.72	-	Favored (88.7%) General / -62.4,-38.2	Favored (66.5%) *mt-10* chi angles: 293.1,174.4,313.8	0.03Å	-	-	-
					Favored	Favored (90.8%)				

A 277		MET	0.73	-	(95.38%) General / -60.7,-45.1	*mmm* chi angles: 291.7,308.1,298.9	0.03Å	-	-	-
A 278		LYS	0.72	-	Favored (99.22%) General / -63.2,-41.8	Favored (57.1%) *mtmt* chi angles: 290.2,184.9,293.7,183.4	0.04Å	-	-	-
A 279		THR	0.71	-	Favored (75.3%) General / -68.5,-44.1	Favored (52.6%) *p* chi angles: 56.6	0.10Å	-	-	-
A 280		ALA	0.7	-	Favored (26.34%) General / -85.5,-26.2	-	0.03Å	-	-	-
A 281		TYR	0.67	-	Allowed (1.42%) General / -126.7,58.0	Favored (51.1%) *m-80* chi angles: 307.4,97.6	0.08Å	-	-	-
A 282		GLY	0.7	-	Favored (47.87%) Glycine / -61.4,132.4	-	-	-	-	-
A 283		SER	0.7	-	Favored (3.86%) General / -132.0,0.6	Favored (70.6%) *m* chi angles: 296.3	0.11Å	-	-	-
A 284		TYR	0.45	-	Allowed (0.26%) General / 83.3,17.7	Favored (60.5%) *m-80* chi angles: 305.9,94.7	2.45Å	-	-	-
A 285		VAL	0.66	0.58Å HB with A 161 LEU HD21	Favored (16.92%) Ile or Val / -115.6,155.1	Favored (8.3%) *m* chi angles: 307.2	0.11Å	-	-	-
A 286		ASP	0.75	-	Favored (22.65%) General / -92.2,111.2	Favored (43.5%) *t0* chi angles: 184.7,12.8	0.03Å	-	-	-
A 287		VAL	0.77	-	Favored (82.55%) Ile or Val / -60.5,-39.9	Favored (82.9%) *t* chi angles: 176.4	0.08Å	-	-	-
A 288		THR	0.78	-	Favored (78.59%) General / -56.6,-48.0	Favored (89.9%) *m* chi angles: 298.7	0.10Å	-	-	-
A 289		ARG	0.7	-	Favored (95.91%) General / -62.9,-44.5	Favored (60.5%) *ttt180* chi angles: 183.5,191.4,178.8,193.9	0.07Å	-	-	-
A 290		TYR	0.74	0.91Å HB2 with A 161 LEU	Favored (82.39%) General /	Favored (22.9%) *t80* chi angles: 178.1,50.3	0.16Å	-	-	-

				HD22	-57.8,-47.5					
#	Alt	Res	High B	Clash > 0.4Å	Ramachandran	Rotamer	Cβ deviation	Bond lengths	Bond angles	Cis Peptides
			Avg: 0.70	Clashscore: 9.96	Outliers: 7 of 654	Poor rotamers: 8 of 581	Outliers: 7 of 633	Outliers: 2 of 656	Outliers: 48 of 656	Non-Trans: 3 of 655
A 291		LEU	0.76	0.68Å HB3 with A 299 LEU HD13	Favored (89.55%) General / -59.9,-46.4	Favored (3.4%) *mt* chi angles: 280.6,194.2	0.22Å	-	OUTLIER(S) worst is N-CA-CB: 4.8 σ	-
A 292		GLN	0.7	-	Favored (81.41%) General / -68.4,-39.7	Favored (97.3%) *mt0* chi angles: 293.5,178.5,332.1	0.10Å	-	-	-
A 293		LEU	0.74	-	Favored (73.15%) General / -59.6,-34.7	Favored (57.7%) *mt* chi angles: 288.2,164	0.06Å	-	-	-
A 294		ILE	0.74	-	Favored (17%) Ile or Val / -71.9,-54.4	Favored (83.1%) *mt* chi angles: 292.8,162.9	0.02Å	-	-	-
A 295		PHE	0.7	0.62Å CE2 with A 466 LEU HD22	Favored (73.09%) General / -68.5,-45.1	Favored (3.8%) *m-10* chi angles: 280,37.3	0.07Å	-	-	-
A 296		ASN	0.7	-	Allowed (0.2%) General / 72.5,-23.3	Favored (47.7%) *m-40* chi angles: 308.2,296.3	0.13Å	-	-	-
A 297		ASP	0.53	-	Allowed (0.72%) General / -109.5,-160.5	Favored (49.7%) *p0* chi angles: 59.8,0.5	0.09Å	-	-	-
A 298		ASN	0.64	-	Favored (38.62%) General / -73.0,129.7	Favored (90.8%) *m-40* chi angles: 290.4,327.4	0.10Å	-	-	-
A 299		LEU	0.57	0.68Å HD13 with A 291 LEU HB3	Favored (42.5%) General / -97.6,133.2	Allowed (1%) *tt* chi angles: 188.6,129.6	0.06Å	-	-	-
A 300		TYR	0.53	-	Favored (24.12%) General / -67.7,124.8	Favored (6.4%) *m-10* chi angles: 283.7,333.5	0.06Å	-	-	-
A 301		MET	0.77	-	Favored (76.17%) General / -59.9,-36.1	Favored (86.7%) *mtp* chi angles: 290.5,178.3,62.2	0.05Å	-	-	-
A 302		ASP	0.78	-	Favored (3.17%) General / -65.1,112.0	Favored (91.7%) *m-30* chi angles: 292.1,348.3	0.05Å	-	-	-

A 303		LEU	0.78	-	Favored (52.91%) General / -120.8,139.6	Favored (2.6%) *mp* chi angles: 280,81.6	0.06Å	-	-	-
A 304		TYR	0.76	-	Favored (22.73%) General / -101.7,110.7	Favored (54.3%) *m-80* chi angles: 283.4,89.2	0.03Å	-	-	-
A 305		GLU	0.74	-	Favored (21.26%) General / -94.0,109.5	Favored (11.8%) *tp30* chi angles: 179.4,64.6,70.8	0.03Å	-	-	-
A 306		THR	0.71	-	Favored (2.33%) Pre-Pro / -76.4,-38.6	Favored (51.3%) *p* chi angles: 56.4	0.31Å	-	OUTLIER(S) worst is CA-C-N: 5.1 σ	-
A 307		PRO	0.7	-	OUTLIER (0)%) Trans-Pro / -119.8,73.4	OUTLIER (0.1%) chi angles: 42.7,312.7,34	0.13Å	-	-	-
A 308		GLU	0.67	0.40Å OE2 with A 272 ARG NH1	Favored (22.05%) General / -47.1,-45.2	Favored (64.4%) *mt-10* chi angles: 294.9,181.1,307.2	0.05Å	-	-	-
A 309		ASP	0.68	-	Favored (81.44%) General / -60.7,-37.4	Favored (95%) *m-30* chi angles: 292,344.5	0.03Å	-	-	-
A 310		TYR	0.71	-	Favored (87.97%) General / -61.2,-47.0	Favored (79.6%) *t80* chi angles: 171.6,256	0.04Å	-	-	-
**#**	**Alt**	**Res**	**High B**	**Clash > 0.4Å**	**Ramachandran**	**Rotamer**	**Cβ deviation**	**Bond lengths**	**Bond angles**	**Cis Peptides**
			Avg: 0.70	Clashscore: 9.96	Outliers: 7 of 654	Poor rotamers: 8 of 581	Outliers: 7 of 633	Outliers: 2 of 656	Outliers: 48 of 656	Non-Trans: 3 of 655
A 311		MET	0.7	-	Favored (95.76%) General / -60.3,-42.5	Favored (97.3%) *mmm* chi angles: 291.1,300.3,293.3	0.03Å	-	-	-
A 312		SER	0.71	-	Favored (97.12%) General / -61.2,-44.5	Favored (62.5%) *m* chi angles: 298.1	0.05Å	-	-	-
A 313		ASN	0.71	-	Favored (67.72%) General / -68.9,-29.4	Favored (94.9%) *m-40* chi angles: 290.9,331.7	0.04Å	-	-	-
A 314		LEU	0.7	-	Favored (74%) General / -62.7,-32.8	Favored (9.4%) *tp* chi angles: 198.5,58.2	0.04Å	-	-	-
A					Favored					

315		VAL	0.65	-	(84.24%) Ile or Val / -57.1,-45.1	Favored (8.5%) *p* chi angles: 67.1	0.11Å	-	-	-
A 316		ASP	0.65	-	Favored (72.44%) General / -70.6,-41.3	Favored (89.4%) *m-30* chi angles: 290.1,337.4	0.12Å	-	-	-
A 317		VAL	0.69	-	Favored (93.81%) Ile or Val / -59.6,-44.6	Favored (71.5%) *t* chi angles: 178.6	0.03Å	-	-	-
A 318		ILE	0.65	-	Favored (15.28%) Ile or Val / -76.1,-16.7	Favored (20.3%) *tt* chi angles: 194.2,168	0.12Å	-	-	-
A 319		ARG	0.59	-	Favored (59.69%) General / -77.2,-14.8	Favored (98.8%) *mtt180* chi angles: 291,176.5,180.6,176.7	0.07Å	-	-	-
A 320		GLU	0.65	-	Favored (57.95%) General / -90.1,-3.4	Favored (96.9%) *mt-10* chi angles: 293.4,179.7,342.7	0.05Å	-	-	-
A 321		THR	0.71	-	Favored (24.61%) Pre-Pro / -120.2,130.9	Favored (44.5%) *p* chi angles: 55.3	0.11Å	-	-	-
A 322		PRO	0.74	0.41Å HD2 with A 325 GLN HG3	Favored (27.43%) Trans-Pro / -65.3,164.0	Favored (20.7%) *Cg_exo* chi angles: 342.2,26.3,334.8	0.08Å	-	-	-
A 323		LYS	0.7	-	Favored (65.59%) General / -54.8,-37.1	Favored (99%) *mttt* chi angles: 295.1,179.4,175.6,184	0.05Å	-	-	-
A 324		LEU	0.7	-	Favored (67.05%) General / -58.9,-52.1	Favored (7.1%) *tt* chi angles: 183.8,145.4	0.10Å	-	-	-
A 325		GLN	0.71	0.61Å HB3 with A 249 ILE HD11	Favored (97.39%) General / -60.8,-42.3	Favored (9.2%) *mm-40* chi angles: 292.5,297.1,28.6	0.10Å	-	-	-
A 326		LEU	0.75	-	Favored (74.85%) General / -61.6,-49.9	Favored (93%) *mt* chi angles: 294.5,171.5	0.04Å	-	-	-
A 327		ALA	0.78	-	Favored (74.16%) General / -60.4,-34.6	-	0.10Å	-	-	-
A 328		ASN	0.74	0.52Å HD21 with A 147 GLU H	Favored (94.11%) General /	Favored (9.7%) *m110* chi angles: 282,118.1	0.10Å	-	-	-

#	Alt	Res	High B	Clash > 0.4Å	Ramachandran	Rotamer	Cβ deviation	Bond lengths	Bond angles	Cis Peptides
					-59.7,-43.7					
A 329		TYR	0.75	-	Favored (71.6%) General / -66.5,-47.9	Favored (40.8%) *t80* chi angles: 168.7,66	0.08Å	-	-	-
A 330		THR	0.76	-	Favored (91.13%) General / -62.0,-46.0	Favored (4.1%) *m* chi angles: 286	0.14Å	-	OUTLIER(S) worst is CA-CB-CG2: 4.0 σ	-
#	Alt	Res	High B	Clash > 0.4Å	Ramachandran	Rotamer	Cβ deviation	Bond lengths	Bond angles	Cis Peptides
			Avg: 0.70	Clashscore: 9.96	Outliers: 7 of 654	Poor rotamers: 8 of 581	Outliers: 7 of 633	Outliers: 2 of 656	Outliers: 48 of 656	Non-Trans: 3 of 655
A 331		MET	0.76	0.44Å HG2 with A 145 TRP HB2	Favored (69.06%) General / -72.2,-38.4	Favored (9.5%) *tpt* chi angles: 194.5,62.1,171	0.05Å	-	-	-
A 332		TRP	0.75	0.63Å CZ3 with A 227 LEU HD13	Favored (77.97%) General / -55.8,-46.1	Favored (38%) *t-100* chi angles: 165.9,250.5	0.06Å	OUTLIER(S) worst is CD2--CE2: 5.8 σ	OUTLIER(S) worst is NE1-CE2-CZ2: 5.5 σ	-
A 333		LYS	0.72	-	Favored (68.5%) General / -58.6,-31.8	Favored (20.6%) *tptp* chi angles: 183.4,63.5,170.7,63.9	0.05Å	-	-	-
A 334		ALA	0.76	-	Favored (43.32%) General / -76.1,-43.5	-	0.04Å	-	-	-
A 335		LEU	0.72	0.59Å CD2 with A 135 LEU HD21	Favored (92.29%) General / -62.9,-38.8	Favored (92.2%) *mt* chi angles: 293.2,175.5	0.08Å	-	-	-
A 336		GLU	0.65	-	Favored (47.59%) General / -66.2,-11.1	Favored (10.4%) *pt0* chi angles: 69.9,180.1,46.9	0.04Å	-	-	-
A 337		ALA	0.65	-	Favored (24.95%) General / -99.7,-7.9	-	0.10Å	-	-	-
A 338		LEU	0.66	-	Favored (15.47%) General / -108.7,-8.1	Favored (67.5%) *mt* chi angles: 296.1,167.4	0.10Å	-	-	-
A 339		ASP	0.6	0.41Å HB3 with A 227 LEU HD21	Favored (59.04%) General / -79.1,-14.6	Favored (42.6%) *p0* chi angles: 64.8,345.6	0.11Å	-	-	-
A 340		ILE	0.56	-	Favored (10.29%) Ile or Val / -93.4,-0.5	Favored (41.3%) *pt* chi angles: 59.5,170.6	0.08Å	-	-	-

A 341		ALA	0.67	-	Favored (31.44%) General / -106.5,9.7	-	0.03Å	-	-	-
A 342		ARG	0.64	0.52Å HH21 with A 132 LEU HD11	Favored (13.03%) General / -116.7,3.3	Favored (53.7%) *ptt90* chi angles: 62.4,182.3,181.4,86.1	0.15Å	-	-	-
A 343		VAL	0.57	-	Favored (25.83%) Pre-Pro / -147.4,163.9	Favored (2.7%) *m* chi angles: 285.9	0.08Å	-	-	-
A 344		PRO	0.55	0.43Å HD3 with A 128 THR HG22	Favored (21.78%) Trans-Pro / -75.6,138.5	Favored (48.2%) *Cg_endo* chi angles: 25,326.9,28.7	0.06Å	-	-	-
A 345		ALA	0.54	-	Favored (57.58%) General / -62.1,142.5	-	0.01Å	-	-	-
A 346		SER	0.41	-	Favored (59.85%) General / -77.0,-11.8	Favored (81%) *p* chi angles: 61.7	0.07Å	-	-	-
A 347		GLN	0.46	-	Allowed (1.4%) General / -105.0,-167.8	Favored (85.6%) *mm-40* chi angles: 299.5,304.6,294.9	0.07Å	-	-	-
A 348		ARG	0.64	-	OUTLIER (0.04)%) General / -62.1,91.6	Favored (64.9%) *mtp180* chi angles: 300.8,192.5,62.9,193	0.03Å	-	-	-
A 349		ALA	0.71	-	Favored (84.11%) General / -60.8,-38.0	-	0.04Å	-	-	-
A 350		ASP	0.66	-	Favored (62.89%) General / -53.0,-39.0	Favored (46.1%) *m-30* chi angles: 277.4,1.3	0.04Å	-	-	-
**#**	**Alt**	**Res**	**High B**	**Clash > 0.4Å**	**Ramachandran**	**Rotamer**	**Cβ deviation**	**Bond lengths**	**Bond angles**	**Cis Peptides**
			Avg: 0.70	Clashscore: 9.96	Outliers: 7 of 654	Poor rotamers: 8 of 581	Outliers: 7 of 633	Outliers: 2 of 656	Outliers: 48 of 656	Non-Trans: 3 of 655
A 351		ILE	0.7	-	Favored (90.94%) Ile or Val / -63.4,-47.7	Favored (11.1%) *mt* chi angles: 296,188.3	0.07Å	-	-	-
A 352		TRP	0.69	-	Favored (97.07%) General / -63.4,-43.6	Favored (97.3%) *m100* chi angles: 291.5,103.7	0.03Å	-	-	-
					Favored					

A 353		CYS	0.81	-	(96.09%) General / -64.1,-40.4	Favored (83.7%) *m* chi angles: 295.2	0.02Å	-	-	-
A 354		VAL	0.81	-	Favored (69.14%) Ile or Val / -56.0,-50.5	Allowed (1%) *t* chi angles: 156.1	0.07Å	-	-	-
A 355		GLN	0.76	-	Favored (92.56%) General / -65.4,-39.7	Favored (91%) *tp40* chi angles: 185.2,65.4,58.5	0.04Å	-	-	-
A 356		LEU	0.79	-	Favored (88.72%) General / -61.6,-46.7	Favored (81.9%) *mt* chi angles: 289.3,170.6	0.06Å	-	-	-
A 357		ALA	0.83	-	Favored (70.66%) General / -67.5,-30.9	-	0.15Å	-	-	-
A 358		GLN	0.77	-	Favored (98.9%) General / -61.1,-42.6	Favored (44.1%) *tt0* chi angles: 188.8,167.1,328.8	0.04Å	-	-	-
A 359		GLN	0.76	-	Favored (56.33%) General / -75.7,-24.4	Favored (98.2%) *mt0* chi angles: 290.3,172.5,340.2	0.07Å	-	-	-
A 360		PHE	0.79	-	Favored (13.66%) General / -103.0,-24.0	Favored (24%) *m-10* chi angles: 302,333.5	0.03Å	-	-	-
A 361		PHE	0.77	0.57Å HB2 with A 114 LEU HD13	Favored (41.18%) Pre-Pro / -131.7,58.9	Favored (97.8%) *m-80* chi angles: 298.2,94.6	0.12Å	-	-	-
A 362		PRO	0.77	-	Favored (42.7%) Trans-Pro / -61.4,-41.2	Favored (17.1%) *Cg_endo* chi angles: 18.1,328.8,32.6	0.10Å	-	-	-
A 363		HIS	0.71	-	Favored (94.07%) General / -65.2,-40.4	Favored (42.7%) *m170* chi angles: 286,163.8	0.05Å	-	OUTLIER(S) worst is ND1-CG-CD2: 4.5 σ	-
A 364		GLN	0.73	-	Favored (92.34%) General / -62.9,-45.5	Favored (18.7%) *mp10* chi angles: 294.8,80.2,350.7	0.06Å	-	-	-
A 365		LEU	0.76	0.50Å HG with A 369 PHE CZ	Favored (73.15%) General / -70.6,-40.5	Favored (9.5%) *tp* chi angles: 198.2,57.7	0.15Å	-	-	-
A 366		GLU	0.71	-	Favored (83.36%) General / -58.7,-40.4	Favored (89.6%) *tt0* chi angles: 186,174,0.2	0.03Å	-	-	-

A 367		SER	0.75	-	Favored (75.73%) General / -55.3,-47.3	Favored (3.4%) *m* chi angles: 279.2	0.14Å	-	-	-
A 368		LEU	0.75	-	Favored (86.76%) General / -67.0,-41.3	Favored (68.7%) *mt* chi angles: 298.3,169.6	0.03Å	-	-	-
A 369		PHE	0.73	0.50Å CZ with A 365 LEU HG	Favored (88.15%) General / -65.4,-44.3	Favored (12.1%) *m-10* chi angles: 290.2,357.7	0.08Å	-	-	-
A 370		HIS	0.71	-	Favored (77.66%) General / -69.5,-39.3	Favored (4.5%) *p-80* chi angles: 91.3,275.1	0.17Å	-	OUTLIER(S) worst is ND1-CG-CD2: 4.3 σ	-
**#**	**Alt**	**Res**	**High B**	**Clash > 0.4Å**	**Ramachandran**	**Rotamer**	**Cβ deviation**	**Bond lengths**	**Bond angles**	**Cis Peptides**
			Avg: 0.70	Clashscore: 9.96	Outliers: 7 of 654	Poor rotamers: 8 of 581	Outliers: 7 of 633	Outliers: 2 of 656	Outliers: 48 of 656	Non-Trans: 3 of 655
A 371		ARG	0.67	-	Favored (96.3%) General / -60.5,-42.3	Favored (98.2%) *mtt180* chi angles: 290.7,173.9,184.1,175.9	0.04Å	-	-	-
A 372		ASN	0.72	-	Favored (33.81%) General / -76.5,-44.7	Allowed (0.5%) *t0* chi angles: 198.5,183.9	0.12Å	-	-	-
A 373		TYR	0.65	-	Favored (6.09%) General / -129.2,12.8	Favored (34.1%) *m-80* chi angles: 278.8,92.7	0.07Å	-	-	-
A 374		ASN	0.67	-	Favored (22.17%) General / -125.1,116.5	Favored (54.1%) *t0* chi angles: 187.7,58.2	0.10Å	-	-	-
A 375		HIS	0.59	-	Favored (13.78%) General / -74.5,115.2	Favored (61.5%) *t70* chi angles: 189.5,61	0.04Å	-	OUTLIER(S) worst is ND1-CG-CD2: 4.4 σ	-
A 376		MET	0.58	-	Favored (66.26%) General / -62.0,-23.1	Favored (80.1%) *mtm* chi angles: 287.8,188.3,288.9	0.04Å	-	-	-
A 377		GLN	0.65	-	Favored (60.61%) General / -75.1,-15.1	Favored (92.1%) *mt0* chi angles: 297.9,175.2,330.6	0.01Å	-	-	-
A 378		MET	0.68	-	Favored (69.63%) General / -58.0,-34.0	Favored (52%) *ttm* chi angles: 185.5,175.2,279.9	0.09Å	-	-	-
					Favored					

A 379		ILE	0.69	0.67Å HG23 with A 425 PHE CD1	(79.58%) Ile or Val / -56.5,-44.0	OUTLIER (0.3%) chi angles: 290.2,332.2	0.16Å	-	-	-
A 380		ASN	0.75	-	Favored (90.63%) General / -66.2,-41.2	Favored (98.4%) *m-40* chi angles: 290.9,339.9	0.02Å	-	-	-
A 381		GLU	0.76	-	Favored (96.83%) General / -61.9,-44.4	Favored (22%) *mm-30* chi angles: 298.2,284.9,300.4	0.06Å	-	-	-
A 382		LEU	0.79	0.66Å HD13 with A 515 LEU HD21	Favored (98.82%) General / -61.6,-43.5	Favored (33%) *tp* chi angles: 175,54.5	0.03Å	-	-	-
A 383		GLN	0.77	-	Favored (82.39%) General / -57.5,-42.2	Favored (83.9%) *tp40* chi angles: 183,69.2,49.4	0.07Å	-	-	-
A 384		SER	0.81	-	Favored (92.35%) General / -63.0,-45.4	Favored (72.1%) *m* chi angles: 295.3	0.04Å	-	-	-
A 385		THR	0.8	-	Favored (87.42%) General / -59.9,-40.0	Favored (23.3%) *m* chi angles: 308	0.06Å	-	-	-
A 386		TRP	0.81	0.55Å CD1 with A 489 ILE HD11	Favored (88.13%) General / -59.5,-46.7	Favored (50.1%) *t60* chi angles: 170.7,74	0.04Å	-	-	-
A 387		SER	0.81	-	Favored (91.56%) General / -59.8,-41.6	Favored (37.5%) *m* chi angles: 289.5	0.04Å	-	-	-
A 388		ASP	0.81	-	Favored (81.28%) General / -63.0,-47.9	Favored (67.1%) *m-30* chi angles: 286.3,329.5	0.05Å	-	-	-
A 389		ILE	0.82	-	Favored (77.41%) Ile or Val / -68.5,-37.2	Favored (56.3%) *mt* chi angles: 288.7,160.9	0.15Å	-	-	-
A 390		LYS	0.8	0.47Å O with A 418 LEU HD21	Favored (99.35%) General / -61.7,-43.2	Favored (37.3%) *ttpt* chi angles: 186.4,176.7,72.1,175	0.03Å	-	-	-
**#**	**Alt**	**Res**	**High B**	**Clash > 0.4Å**	**Ramachandran**	**Rotamer**	**Cβ deviation**	**Bond lengths**	**Bond angles**	**Cis Peptides**
			Avg: 0.70	Clashscore: 9.96	Outliers: 7 of 654	Poor rotamers: 8 of 581	Outliers: 7 of 633	Outliers: 2 of 656	Outliers: 48 of 656	Non-Trans: 3 of 655
					Favored	Favored (36.2%) *ttp-*				

A 391		ARG	0.77	-	(92.83%) General / -59.9,-45.3	*110* chi angles: 183.4,186,60.6,248.7	0.03Å	-	-	-
A 392		VAL	0.83	-	Favored (83.77%) Ile or Val / -58.9,-41.7	Favored (37.9%) *t* chi angles: 167	0.06Å	-	-	-
A 393		PHE	0.79	0.44Å HD1 with A 418 LEU HD22	Favored (88.8%) General / -59.2,-46.3	Favored (19%) *t80* chi angles: 180.9,284	0.04Å	-	-	-
A 394		ARG	0.75	-	Favored (98.95%) General / -62.0,-42.2	Favored (74.2%) *ttp80* chi angles: 189.3,184.1,57.7,74.7	0.03Å	-	-	-
A 395		GLU	0.76	-	Favored (96.99%) General / -63.5,-43.5	Favored (72.6%) *mt-10* chi angles: 291.1,163.9,332.1	0.04Å	-	-	-
A 396		ASP	0.76	-	Favored (68.45%) General / -65.7,-27.6	Favored (8.1%) *t70* chi angles: 198.7,67.7	0.04Å	-	-	-
A 397		LEU	0.74	-	Favored (57.57%) General / -52.9,-34.9	Favored (52.1%) *mt* chi angles: 287.6,176	0.04Å	-	-	-
A 398		GLN	0.65	-	Favored (94.21%) General / -62.4,-39.7	Favored (11.4%) *mt0* chi angles: 291.6,171.5,129.5	0.03Å	-	-	-
A 399		ALA	0.67	-	Favored (80.09%) General / -68.2,-42.4	-	0.05Å	-	-	-
A 400		SER	0.7	-	Favored (75.26%) General / -70.2,-37.9	Favored (89.6%) *p* chi angles: 68.9	0.20Å	-	-	-
A 401		GLU	0.65	-	Favored (61.61%) General / -72.0,-24.6	Favored (13.3%) *pt0* chi angles: 78,178.4,349.6	0.11Å	-	-	-
A 402		ARG	0.55	-	Favored (68.32%) General / -57.6,-33.5	Favored (82.1%) *ttp80* chi angles: 181.7,186.5,62.6,81.2	0.05Å	-	-	-
A 403		LEU	0.42	-	Favored (58.56%) General / -82.5,-11.4	Favored (94.7%) *mt* chi angles: 296.4,174.9	0.12Å	-	-	-
A 404		LEU	0.48	-	Favored (8.17%) General / 67.7,25.1	Favored (30%) *mt* chi angles: 306.5,171	0.17Å	-	-	-

A 405		TRP	0.64	-	Favored (72.66%) General / -66.5,-31.7	Favored (51.6%) *m-10* chi angles: 291.5,339.1	0.07Å	-	-	-
A 406		LEU	0.72	-	Favored (55.23%) General / -119.9,134.5	Favored (91.7%) *mt* chi angles: 297.9,173.7	0.08Å	-	-	-
A 407		THR	0.75	-	Favored (30.12%) General / -75.2,160.7	Favored (7.1%) *p* chi angles: 45.7	0.04Å	-	-	-
A 408		LEU	0.73	-	Favored (76.8%) General / -56.8,-40.9	Favored (30.1%) *mt* chi angles: 288.5,159.4	0.06Å	-	-	-
A 409		GLU	0.72	-	Favored (77.23%) General / -61.5,-49.3	Favored (68.2%) *tt0* chi angles: 182.4,169.6,344	0.03Å	-	-	-
A 410		THR	0.76	-	Favored (90.75%) General / -63.6,-38.3	Favored (98.4%) *m* chi angles: 300.2	0.07Å	-	-	-
**#**	**Alt**	**Res**	**High B**	**Clash > 0.4Å**	**Ramachandran**	**Rotamer**	**Cβ deviation**	**Bond lengths**	**Bond angles**	**Cis Peptides**
			Avg: 0.70	Clashscore: 9.96	Outliers: 7 of 654	Poor rotamers: 8 of 581	Outliers: 7 of 633	Outliers: 2 of 656	Outliers: 48 of 656	Non-Trans: 3 of 655
A 411		ARG	0.73	-	Favored (97.92%) General / -60.8,-42.6	Allowed (1.9%) *tmm160* chi angles: 187.6,270.8,298.1,197.2	0.05Å	-	-	-
A 412		GLN	0.77	-	Favored (93.09%) General / -59.7,-45.1	Favored (94.2%) *mt0* chi angles: 289.5,170.4,343.3	0.06Å	-	-	-
A 413		LYS	0.78	-	Favored (79.45%) General / -65.7,-35.0	Favored (91.6%) *mttt* chi angles: 296.1,179,185.3,170.3	0.14Å	-	-	-
A 414		ALA	0.81	-	Favored (94.58%) General / -61.8,-40.2	-	0.07Å	-	-	-
A 415		ILE	0.8	-	Favored (87.41%) Ile or Val / -59.7,-48.5	Favored (78.6%) *mt* chi angles: 294.7,175	0.02Å	-	-	-
A 416		THR	0.8	-	Favored (83.45%) General / -57.1,-44.7	Favored (93.3%) *m* chi angles: 299.2	0.10Å	-	-	-

A 417		LYS	0.78	-	Favored (96.71%) General / -63.5,-43.6	Favored (76.2%) *tttt* chi angles: 180.6,167.9,187.3,174.7	0.05Å	-	-	-
A 418		LEU	0.79	0.47Å HD21 with A 390 LYS O	Favored (98.99%) General / -63.3,-42.9	Favored (36.4%) *tp* chi angles: 174.8,68.6	0.08Å	-	-	-
A 419		GLU	0.77	-	Favored (86.2%) General / -61.5,-38.1	Favored (97%) *mt-10* chi angles: 289.2,179.6,357.5	0.05Å	-	-	-
A 420		ALA	0.79	-	Favored (61.68%) General / -72.6,-17.3	-	0.08Å	-	-	-
A 421		LEU	0.78	-	Favored (2.59%) General / -54.1,116.5	Favored (46.2%) *tp* chi angles: 184.6,62.8	0.04Å	-	-	-
A 422		LYS	0.76	-	Favored (35.76%) General / -89.4,130.4	Favored (64%) *tttt* chi angles: 197.8,175.7,190.3,178.5	0.07Å	-	-	-
A 423		LEU	0.76	-	Favored (34.27%) General / -96.9,138.6	Favored (51.3%) *tp* chi angles: 173.2,64.3	0.08Å	-	-	-
A 424		HIS	0.77	0.42Å CD2 with A 488 ARG HD2	Favored (49.71%) General / -120.5,126.2	Favored (25.2%) *m-70* chi angles: 288.9,242	0.07Å	-	-	-
A 425		PHE	0.75	0.67Å CD1 with A 379 ILE HG23	Favored (2.55%) General / -128.0,-29.5	Favored (13.6%) *p90* chi angles: 47.7,78.6	0.07Å	-	-	-
A 426		ARG	0.65	0.44Å HG2 with A 427 THR H	Favored (12.18%) General / -166.7,170.8	Favored (45.6%) *ttp-170* chi angles: 186.7,199.2,68.2,194.3	0.09Å	-	-	-
A 427		THR	0.53	0.44Å H with A 426 ARG HG2	Favored (2.01%) General / -130.0,-168.7	Favored (8.7%) *t* chi angles: 184.6	0.09Å	-	-	-
A 428		HIS	0.43	-	Favored (7.52%) General / -137.7,110.3	Favored (90.1%) *t70* chi angles: 178.2,75.3	0.09Å	-	OUTLIER(S) worst is ND1-CG-CD2: 4.5 σ	-
A 429		ASP	0.53	-	Favored (55.59%) General / -117.4,129.1	Favored (96.3%) *m-30* chi angles: 290.6,343.7	0.06Å	-	-	-
A		ASP	0.51	-	Favored (2.17%)	Favored (90.9%) *m-30*	0.14Å	-	-	-

430					General / -100.3,-172.2	chi angles: 289.9,338.5				
**#**	**Alt**	**Res**	**High B**	**Clash > 0.4Å**	**Ramachandran**	**Rotamer**	**Cβ deviation**	**Bond lengths**	**Bond angles**	**Cis Peptides**
			Avg: 0.70	Clashscore: 9.96	Outliers: 7 of 654	Poor rotamers: 8 of 581	Outliers: 7 of 633	Outliers: 2 of 656	Outliers: 48 of 656	Non-Trans: 3 of 655
A 431		ASN	0.62	-	Allowed (1.91%) General / -78.4,12.6	Favored (72.8%) *m-40* chi angles: 294,301.9	0.06Å	-	OUTLIER(S) worst is CA-CB-CG: 4.5 σ	-
A 432		LYS	0.63	-	Favored (60.38%) General / -75.5,-13.8	Favored (45.9%) *mttp* chi angles: 298.9,170.2,176.1,65.7	0.05Å	-	OUTLIER(S) worst is C-N-CA: 5.5 σ	-
A 433		LEU	0.68	-	Favored (2.12%) General / -46.2,-30.8	Favored (46.8%) *tp* chi angles: 182.7,65.3	0.21Å	-	OUTLIER(S) worst is N-CA-CB: 4.9 σ	-
A 434		ILE	0.67	-	Favored (86.93%) Ile or Val / -58.2,-44.1	Favored (94.8%) *mt* chi angles: 294,170.2	0.05Å	-	-	-
A 435		ARG	0.59	-	Favored (67.08%) General / -68.3,-28.4	Favored (97.6%) *mtt180* chi angles: 288.7,180.5,178.1,173.7	0.06Å	-	-	-
A 436		GLN	0.66	-	Favored (76.32%) General / -60.7,-35.5	Favored (17.7%) *mt0* chi angles: 296.4,189.4,118.9	0.03Å	-	-	-
A 437		MET	0.69	-	Favored (41.59%) General / -101.4,4.7	Allowed (0.8%) *pp-130* chi angles: 65.5,75.2,188.7	0.05Å	-	-	-
A 438		HIS	0.66	-	Favored (37.56%) General / -51.4,-35.1	Favored (86.1%) *t70* chi angles: 176.9,78.1	0.03Å	-	OUTLIER(S) worst is ND1-CG-CD2: 4.2 σ	-
A 439		GLY	0.73	-	Favored (87.12%) Glycine / -79.8,-1.4	-	-	-	-	-
A 440		LEU	0.73	-	Favored (46.64%) General / -104.2,123.0	Favored (62.4%) *mt* chi angles: 295.6,166.4	0.05Å	-	-	-
A 441		ILE	0.72	-	Favored (59.07%) Ile or Val / -126.8,121.8	Favored (85.3%) *mt* chi angles: 299.1,171.2	0.06Å	-	-	-
A 442		LEU	0.76	-	Favored (56.33%) General /	Favored (28.7%) *mt* chi angles: 307.5,172.5	0.04Å	-	-	-

#	Alt	Res	High B	Clash > 0.4Å	Ramachandran	Rotamer	Cβ deviation	Bond lengths	Bond angles	Cis Peptides
					-113.6,130.7					
A 443		ASN	0.75	-	Favored (40.5%) General / -94.3,128.0	Favored (69.8%) *m-40* chi angles: 300,297.2	0.11Å	-	-	-
A 444		MET	0.71	-	Favored (62.38%) General / -71.4,-16.1	Favored (93.1%) *mtp* chi angles: 288.3,177.7,67.1	0.04Å	-	-	-
A 445		ASP	0.68	-	Favored (42.56%) General / -99.4,0.4	Favored (17.1%) *p0* chi angles: 61.4,136	0.08Å	-	-	-
A 446		ARG	0.7	-	Allowed (1.43%) General / -129.3,67.5	Favored (97.7%) *mtt180* chi angles: 293.4,182.1,182.3,184.5	0.05Å	-	-	-
A 447		PHE	0.71	-	Favored (85.4%) General / -57.9,-46.2	Favored (24.4%) *t80* chi angles: 177.9,51.2	0.03Å	-	-	-
A 448		TYR	0.71	0.51Å CD2 with A 134 THR HB	Favored (82.13%) Pre-Pro / -63.7,-45.9	Favored (49%) *t80* chi angles: 186.5,95.2	0.14Å	-	OUTLIER(S) worst is CA-C-N: 5.3 σ	-
A 449		PRO	0.8	-	Favored (96.68%) Trans-Pro / -58.4,-32.6	Favored (76.6%) *Cg_exo* chi angles: 329,37.2,330	0.09Å	-	-	-
A 450		ASN	0.78	-	Favored (86.14%) General / -62.2,-47.1	Favored (6%) *m110* chi angles: 294.2,172.5	0.07Å	-	-	-
**#**	**Alt**	**Res**	**High B**	**Clash > 0.4Å**	**Ramachandran**	**Rotamer**	**Cβ deviation**	**Bond lengths**	**Bond angles**	**Cis Peptides**
			Avg: 0.70	Clashscore: 9.96	Outliers: 7 of 654	Poor rotamers: 8 of 581	Outliers: 7 of 633	Outliers: 2 of 656	Outliers: 48 of 656	Non-Trans: 3 of 655
A 451		LEU	0.79	-	Favored (94.21%) General / -65.2,-40.6	Favored (39.8%) *tp* chi angles: 182.1,54.7	0.13Å	-	-	-
A 452		VAL	0.78	-	Favored (94.38%) Ile or Val / -60.5,-43.5	Favored (84%) *t* chi angles: 177.1	0.07Å	-	-	-
A 453		SER	0.81	-	Favored (91.41%) General / -59.7,-41.7	Favored (63.2%) *m* chi angles: 294	0.08Å	-	-	-
A 454		VAL	0.8	-	Favored (95.14%) Ile or Val / -62.9,-42.1	Favored (74.2%) *t* chi angles: 172.6	0.06Å	-	-	-

A 455		LEU	0.78	-	Favored (85.96%) General / -59.7,-47.4	Favored (84.5%) *mt* chi angles: 292,176.5	0.08Å	-	-	-
A 456		GLN	0.75	-	Favored (86.06%) General / -62.3,-47.0	Favored (93.5%) *mt0* chi angles: 289.5,179,341.1	0.05Å	-	-	-
A 457		TRP	0.73	-	Favored (93.97%) General / -62.0,-45.3	Favored (87.7%) *t60* chi angles: 179.9,83.6	0.05Å	-	-	-
A 458		LYS	0.76	-	Favored (96.51%) General / -63.9,-43.2	Favored (85.6%) *tttt* chi angles: 184,177.4,174,184.8	0.03Å	-	-	-
A 459		THR	0.76	-	Favored (90.41%) General / -66.2,-40.8	Favored (62.2%) *m* chi angles: 303.4	0.05Å	-	-	-
A 460		GLN	0.71	-	Favored (92.74%) General / -59.4,-45.0	Favored (86.1%) *mt0* chi angles: 285.8,178,345.7	0.08Å	-	-	-
A 461		ARG	0.68	-	Favored (85.35%) General / -60.8,-38.5	Favored (69.3%) *ttt-90* chi angles: 184.5,184.3,178.5,272.4	0.03Å	-	-	-
A 462		GLY	0.77	-	Favored (43.14%) Glycine / -71.7,-45.7	-	-	-	-	-
A 463		LEU	0.71	0.42Å CD1 with A 169 ARG HD3	Favored (81.45%) General / -67.8,-37.0	Favored (54.8%) *mt* chi angles: 301,169.9	0.03Å	-	-	-
A 464		ALA	0.76	-	Favored (33.53%) General / -62.3,-13.9	-	0.05Å	-	-	-
A 465		LYS	0.72	-	Favored (60.55%) General / -74.4,-12.5	Favored (80.2%) *mttt* chi angles: 291.3,186.6,169.2,188.4	0.08Å	-	-	-
A 466		LEU	0.73	0.62Å HD22 with A 295 PHE CE2	Favored (76.92%) General / -55.9,-43.1	Favored (50.3%) *tp* chi angles: 179.1,66.3	0.08Å	-	-	-
A 467		MET	0.71	-	Favored (53.88%) General / -95.0,4.3	Favored (86.4%) *mtp* chi angles: 296.7,182.4,66.7	0.09Å	-	-	-
A		THR	0.72	-	Favored (10.22%)	Favored (28.5%) *p*	0.15Å	-	-	-

#	Alt	Res	High B	Clash > 0.4Å	Ramachandran	Rotamer	Cβ deviation	Bond lengths	Bond angles	Cis Peptides
468					General / -94.6,169.4	chi angles: 69.8				
A 469		GLU	0.69	-	Favored (22.38%) Pre-Pro / -94.6,138.0	Favored (96%) *mt-10* chi angles: 295.7,179.4,4.2	0.06Å	-	-	-
A 470		PRO	0.7	-	Favored (18.79%) Trans-Pro / -48.0,140.3	Favored (34.2%) *Cg_exo* chi angles: 339.4,35.2,322.1	0.13Å	-	-	-
**#**	**Alt**	**Res**	**High B**	**Clash > 0.4Å**	**Ramachandran**	**Rotamer**	**Cβ deviation**	**Bond lengths**	**Bond angles**	**Cis Peptides**
			Avg: 0.70	Clashscore: 9.96	Outliers: 7 of 654	Poor rotamers: 8 of 581	Outliers: 7 of 633	Outliers: 2 of 656	Outliers: 48 of 656	Non-Trans: 3 of 655
A 471		VAL	0.6	0.45Å HG23 with A 475 GLU HB2	Favored (3.51%) Ile or Val / -105.2,21.4	Allowed (1.2%) *m* chi angles: 283	0.10Å	-	OUTLIER(S) worst is C-N-CA: 5.0 σ	-
A 472		ALA	0.59	0.62Å HB1 with A 486 LYS HG2	Allowed (0.58%) General / -35.3,-51.1	-	0.15Å	-	OUTLIER(S) worst is N-CA-CB: 4.3 σ	-
A 473		GLU	0.62	-	Favored (89.46%) General / -60.9,-39.4	Favored (5.7%) *mt-10* chi angles: 275.3,200.1,62.4	0.17Å	-	-	-
A 474		ASP	0.61	-	Favored (8.48%) General / -96.4,-41.7	Favored (67%) *m-30* chi angles: 297.8,346.3	0.11Å	-	-	-
A 475		GLU	0.57	0.45Å HB2 with A 471 VAL HG23	Allowed (1.33%) General / -66.4,104.9	Favored (9%) *tt0* chi angles: 184.2,201.1,53	0.09Å	-	-	-
A 476		VAL	0.66	-	Favored (7.48%) Ile or Val / -59.2,148.0	Allowed (1%) *t* chi angles: 197.2	0.09Å	-	-	-
A 477		HIS	0.6	0.45Å HB2 with A 488 ARG HH22	Favored (41.46%) General / -74.5,147.8	Favored (4.9%) *t-90* chi angles: 209.7,277.3	0.08Å	-	OUTLIER(S) worst is ND1-CG-CD2: 4.2 σ	-
A 478		LYS	0.64	-	Favored (61.56%) General / -72.8,-17.8	Favored (96.9%) *mttt* chi angles: 293.9,180.8,174,184.1	0.07Å	-	-	-
A 479		LEU	0.54	-	Favored (21.87%) Pre-Pro / -120.0,164.5	Favored (67.2%) *mt* chi angles: 296.6,182.7	0.09Å	-	-	-
A 480		PRO	0.63	0.43Å HG3 with A	Favored (7.13%) Trans-Pro /	Favored (38.5%) *Cg_endo* chi angles:	0.14Å	-	-	-

#	Alt	Res	High B	Clash > 0.4Å	Ramachandran	Rotamer	Cβ deviation	Bond lengths	Bond angles	Cis Peptides
				495 PHE CE1	-76.6,67.8	23,330.7,24.5				
A 481		HIS	0.58	-	Favored (52.95%) General / -124.5,130.7	Favored (64.4%) *t70* chi angles: 189.7,63.5	0.05Å	-	OUTLIER(S) worst is ND1-CG-CD2: 4.3 σ	-
A 482		TYR	0.77	0.44Å CZ with A 526 GLY HA2	Favored (31.44%) General / -61.7,127.4	Favored (11.7%) *t80* chi angles: 169.9,97.7	0.06Å	-	-	-
A 483		GLU	0.76	-	Favored (37.49%) General / -106.0,118.8	Favored (52%) *tp30* chi angles: 181.9,65.2,34.1	0.04Å	-	-	-
A 484		ILE	0.77	0.47Å HG21 with A 534 TYR CD1	Favored (85.29%) Ile or Val / -65.5,-47.8	Favored (69.7%) *mt* chi angles: 289.2,172.6	0.09Å	-	-	-
A 485		GLN	0.71	-	Favored (98.7%) General / -63.4,-41.2	Favored (48.4%) *mm-40* chi angles: 281.8,289.7,295.7	0.12Å	-	-	-
A 486		LYS	0.72	0.62Å HG2 with A 472 ALA HB1	Favored (50.69%) General / -96.1,5.6	Favored (72%) *mttt* chi angles: 305.6,181.1,176.7,191.8	0.11Å	-	-	-
A 487		ASN	0.77	-	Allowed (0.47%) General / 36.5,65.3	Favored (61.6%) *t0* chi angles: 191.7,27.3	0.31Å	-	-	-
A 488		ARG	0.75	0.45Å HH22 with A 477 HIS HB2	Favored (38.81%) General / -152.6,156.2	Favored (39.8%) *ptt90* chi angles: 66.9,167.7,176.2,72.4	0.10Å	-	-	-
A 489		ILE	0.77	0.55Å HD11 with A 386 TRP CD1	Favored (17.09%) Ile or Val / -115.3,150.9	Favored (5.1%) *pt* chi angles: 46.7,174.5	0.04Å	-	-	-
A 490		GLN	0.76	-	Favored (20.1%) General / -138.0,123.9	Favored (36.2%) *tp40* chi angles: 165.2,63.4,53.5	0.02Å	-	-	-
**#**	**Alt**	**Res**	**High B**	**Clash > 0.4Å**	**Ramachandran**	**Rotamer**	**Cβ deviation**	**Bond lengths**	**Bond angles**	**Cis Peptides**
			Avg: 0.70	Clashscore: 9.96	Outliers: 7 of 654	Poor rotamers: 8 of 581	Outliers: 7 of 633	Outliers: 2 of 656	Outliers: 48 of 656	Non-Trans: 3 of 655
A 491		VAL	0.79	-	Favored (58.46%) Pre-Pro / -125.0,84.8	Allowed (0.5%) *t* chi angles: 199.6	0.04Å	-	-	-
A 492		PRO	0.76	-	Favored (23.82%) Trans-Pro / -59.2,159.0	Favored (25%) *Cg_exo* chi angles: 340.9,33.8,323.2	0.06Å	-	-	-

A 493		ILE	0.7	-	Favored (16.92%) Ile or Val / -54.5,-26.9	Favored (35.1%) *pt* chi angles: 66.4,170.1	0.06Å	-	-	-
A 494		THR	0.74	-	Favored (65.99%) General / -66.9,-23.5	Favored (30.1%) *p* chi angles: 53.1	0.04Å	-	-	-
A 495		PHE	0.73	0.43Å CE1 with A 480 PRO HG3	Favored (27.44%) General / -104.6,-0.6	Favored (51.5%) *m-80* chi angles: 282.9,90.8	0.04Å	-	OUTLIER(S) worst is CA-CB-CG: 6.0 σ	-
A 496		LEU	0.73	-	Favored (16.22%) General / -100.0,-20.2	Favored (76.9%) *mt* chi angles: 302.6,178.7	0.13Å	-	-	-
A 497		GLN	0.6	-	Favored (52.06%) General / -96.6,4.2	Favored (63.9%) *mm-40* chi angles: 288,292.1,337.6	0.11Å	-	-	-
A 498		ALA	0.63	-	Favored (23.65%) General / -76.3,121.9	-	0.05Å	-	-	-
A 499		ARG	0.53	-	Favored (61.04%) General / -64.3,-15.8	Favored (82.7%) *mtt-85* chi angles: 289.1,180.2,192.2,286.5	0.12Å	-	-	-
A 500		PHE	0.58	0.42Å O with A 514 THR HG21	Favored (47.26%) General / -79.2,-20.9	Favored (42.4%) *m-80* chi angles: 305.2,119	0.06Å	-	OUTLIER(S) worst is CA-CB-CG: 6.3 σ	-
A 501		PHE	0.75	0.55Å HA with A 514 THR HB	Allowed (0.45%) General / -108.3,-90.2	Favored (3.5%) *m-80* chi angles: 297.6,64.6	0.10Å	-	-	-
A 502		TRP	0.72	-	Favored (47.68%) General / -119.2,142.8	Favored (49.7%) *t-100* chi angles: 181.5,271.4	0.06Å	-	-	-
A 503		ASP	0.73	-	Favored (6.43%) Pre-Pro / -153.8,131.8	Favored (35.7%) *t0* chi angles: 180,334.3	0.11Å	-	OUTLIER(S) worst is CA-C-N: 5.4 σ	-
A 504		PRO	0.76	-	Favored (47.06%) Trans-Pro / -53.4,-29.2	Favored (79.8%) *Cg_exo* chi angles: 329.1,37.4,330.1	0.02Å	-	-	-
A 505		ALA	0.76	0.74Å HB1 with A 37 LEU HD13	Favored (25.69%) General / -101.8,-4.8	-	0.13Å	-	-	-
A 506		TYR	0.75	-	Favored (66.83%) Pre-Pro /	Favored (9.7%) *m-10*	0.14Å	-	-	-

					-82.2,158.4	chi angles: 280.8,6.8				
A 507		PRO	0.81	0.55Å HD2 with A 510 LEU HD12	Favored (16.09%) Trans-Pro / -48.4,143.0	Favored (41.3%) *Cg_exo* chi angles: 338.3,27.8,336.3	0.12Å	-	-	-
A 508		ASN	0.78	-	Favored (88.04%) General / -61.9,-38.4	Favored (65.7%) *m-40* chi angles: 298.3,287.2	0.08Å	-	-	-
A 509		GLY	0.82	-	Favored (48.54%) Glycine / -53.4,-34.4	-	-	-	-	-
A 510		LEU	0.79	0.64Å HD13 with A 41 LYS HG2	Favored (22.19%) General / -73.5,-50.3	Favored (26.7%) *mt* chi angles: 309.3,174	0.07Å	-	-	-
**#**	**Alt**	**Res**	**High B**	**Clash > 0.4Å**	**Ramachandran**	**Rotamer**	**Cβ deviation**	**Bond lengths**	**Bond angles**	**Cis Peptides**
			Avg: 0.70	Clashscore: 9.96	Outliers: 7 of 654	Poor rotamers: 8 of 581	Outliers: 7 of 633	Outliers: 2 of 656	Outliers: 48 of 656	Non-Trans: 3 of 655
A 511		LYS	0.77	-	Favored (81.15%) General / -57.3,-42.2	Favored (38.6%) *mtmt* chi angles: 286,192.7,275,183.3	0.07Å	-	-	-
A 512		TYR	0.8	-	Favored (81.3%) General / -68.5,-39.5	Favored (69.8%) *m-80* chi angles: 286.6,272	0.07Å	-	-	-
A 513		ALA	0.81	-	Favored (66.06%) General / -64.7,-20.0	-	0.15Å	-	-	-
A 514		THR	0.78	0.61Å HA with A 634 LEU HD11	Allowed (0.59%) General / -113.1,-83.8	Favored (54.9%) *p* chi angles: 56.8	0.09Å	-	-	-
A 515		LEU	0.8	0.66Å HD21 with A 382 LEU HD13	Favored (81.3%) General / -68.0,-37.4	Favored (41.6%) *tp* chi angles: 184.5,57.1	0.08Å	-	-	-
A 516		GLY	0.81	-	Favored (30.74%) Glycine / -49.1,-49.3	-	-	-	-	-
A 517		VAL	0.81	-	Favored (99.28%) Ile or Val / -63.1,-44.0	Favored (66.1%) *t* chi angles: 179.2	0.03Å	-	-	-
A 518		LEU	0.81	0.44Å HD12 with A 521 ARG HE	Favored (95.25%) General / -60.0,-42.8	Favored (47.8%) *tp* chi angles: 183.5,57.4	0.04Å	-	-	-

#	Alt	Res	High B	Clash > 0.4Å	Ramachandran	Rotamer	Cβ deviation	Bond lengths	Bond angles	Cis Peptides
A 519		LEU	0.81	-	Favored (83.89%) General / -60.7,-48.0	Favored (33.5%) *mt* chi angles: 287.9,180.2	0.12Å	-	-	-
A 520		ALA	0.83	-	Favored (91.11%) General / -62.3,-38.9	-	0.09Å	-	-	-
A 521		ARG	0.78	0.44Å HE with A 518 LEU HD12	Favored (91.86%) General / -59.0,-44.9	Favored (79.6%) *ttp80* chi angles: 187,189.8,64.8,79.7	0.06Å	-	-	-
A 522		GLN	0.79	0.42Å HB3 with A 489 ILE HG22	Favored (86.43%) General / -66.5,-42.9	Favored (81.8%) *mt0* chi angles: 293.9,181.3,0.1	0.07Å	-	-	-
A 523		MET	0.81	0.43Å HE2 with A 386 TRP CD1	Favored (89.3%) General / -58.4,-44.9	Favored (22.9%) *mtt* chi angles: 289.7,188.9,206.2	0.09Å	-	OUTLIER(S) worst is CG-SD-CE: 5.1 σ	-
A 524		LEU	0.79	-	Favored (64.56%) General / -68.5,-23.3	Favored (21.4%) *mt* chi angles: 291,186.8	0.05Å	-	-	-
A 525		HIS	0.79	-	Favored (52.42%) General / -64.6,-13.2	Favored (11.6%) *m-70* chi angles: 276,309.7	0.07Å	-	-	-
A 526		GLY	0.81	0.44Å HA2 with A 482 TYR CZ	Favored (84.13%) Glycine / -78.3,-2.5	-	-	-	-	-
A 527		PHE	0.79	-	Allowed (1.15%) General / -146.0,9.7	Favored (95%) *m-80* chi angles: 293.2,277.8	0.08Å	-	-	-
A 528		ASP	0.78	-	OUTLIER (0.04)%) General / -75.2,-153.2	Favored (22.2%) *p0* chi angles: 75.8,5.2	0.20Å	-	-	-
A 529		GLY	0.79	-	Favored (82.03%) Glycine / -76.9,-15.0	-	-	-	-	-
A 530		VAL	0.77	0.75Å HG13 with A 265 LEU HD11	Favored (5.83%) Ile or Val / -109.4,-53.8	Favored (10.5%) *t* chi angles: 161.8	0.11Å	-	-	-
**#**	**Alt**	**Res**	**High B**	**Clash > 0.4Å**	**Ramachandran**	**Rotamer**	**Cβ deviation**	**Bond lengths**	**Bond angles**	**Cis Peptides**
			Avg: 0.70	Clashscore: 9.96	Outliers: 7 of 654	Poor rotamers: 8 of 581	Outliers: 7 of 633	Outliers: 2 of 656	Outliers: 48 of 656	Non-Trans: 3 of 655

Favored

A 531		GLY	0.79	-	(70.2%) Glycine / -60.5,-29.8	-	-	-	-	-
A 532		ARG	0.72	-	Favored (63.08%) General / -67.0,-14.6	Favored (30.3%) *ptt90* chi angles: 79,180.6,185.5,73.9	0.06Å	-	-	-
A 533		ARG	0.7	-	Favored (30.89%) General / -91.2,9.5	Favored (85.9%) *mtt-85* chi angles: 293.5,192.5,175.9,280.2	0.12Å	-	-	-
A 534		TYR	0.73	0.56Å CE1 with A 265 LEU HD13	Favored (50.43%) General / -124.7,144.0	Favored (31.5%) *m-80* chi angles: 278,95.5	0.11Å	-	-	-
A 535		ASP	0.76	-	Allowed (0.73%) General / -88.5,-161.3	Favored (52.3%) *p0* chi angles: 67.7,11.3	0.10Å	-	-	-
A 536		ALA	0.8	-	Favored (63.47%) General / -69.8,-20.2	-	0.12Å	-	-	-
A 537		TYR	0.71	-	Favored (19.02%) General / -112.6,8.1	Favored (96.5%) *m-80* chi angles: 295.2,98.2	0.06Å	-	-	-
A 538		GLY	0.77	-	Favored (62.11%) Glycine / 86.2,16.8	-	-	-	-	-
A 539		TYR	0.68	-	Favored (35.36%) General / -95.0,136.4	Favored (9.4%) *m-80* chi angles: 295.2,67.7	0.05Å	-	-	-
A 540		LYS	0.7	0.48Å HE2 with A 265 LEU HB3	Favored (2.71%) General / -75.2,92.6	Favored (32.7%) *ttmt* chi angles: 184.5,174.8,292.3,185.3	0.04Å	-	-	-
A 541		ASN	0.7	-	Favored (27.02%) General / -148.4,143.7	Favored (14.8%) *t0* chi angles: 196,293.4	0.06Å	-	-	-
A 542		ASN	0.71	-	Favored (18.79%) General / -81.8,112.3	Favored (35.7%) *t0* chi angles: 191.8,6.5	0.03Å	-	-	-
A 543		TRP	0.71	-	Favored (26.3%) General / -108.9,11.0	Favored (50%) *p-90* chi angles: 49.1,267.6	0.07Å	-	-	-
A 544		TRP	0.72	-	Favored (50.54%) General /	Favored (45%) *m100* chi angles: 293.8,68.6	0.13Å	-	-	-

#	Alt	Res	High B	Clash > 0.4Å	Ramachandran	Rotamer	Cβ deviation	Bond lengths	Bond angles	Cis Peptides
					-105.0,132.9					
A 545		ASP	0.73	-	Favored (23.98%) General / -57.6,148.4	Favored (2.7%) *m-30* chi angles: 309.4,348.8	0.12Å	-	-	-
A 546		HIS	0.7	-	Favored (69.71%) General / -54.2,-42.4	Favored (87.4%) *t70* chi angles: 182.2,69.6	0.05Å	-	OUTLIER(S) worst is ND1-CG-CD2: 4.3 σ	-
A 547		THR	0.71	-	Favored (93.41%) General / -62.8,-45.3	Favored (62.5%) *m* chi angles: 303.4	0.05Å	-	-	-
A 548		SER	0.77	-	Favored (87.58%) General / -66.6,-38.7	Favored (43.7%) *m* chi angles: 290.3	0.11Å	-	-	-
A 549		GLU	0.72	-	Favored (94.72%) General / -62.1,-45.1	Favored (29.8%) *tt0* chi angles: 185.8,160.3,26.8	0.09Å	-	-	-
A 550		SER	0.77	-	Favored (98.46%) General / -61.7,-42.1	Favored (40.1%) *t* chi angles: 178	0.06Å	-	-	-
**#**	**Alt**	**Res**	**High B**	**Clash > 0.4Å**	**Ramachandran**	**Rotamer**	**Cβ deviation**	**Bond lengths**	**Bond angles**	**Cis Peptides**
			Avg: 0.70	Clashscore: 9.96	Outliers: 7 of 654	Poor rotamers: 8 of 581	Outliers: 7 of 633	Outliers: 2 of 656	Outliers: 48 of 656	Non-Trans: 3 of 655
A 551		SER	0.76	-	Favored (66.96%) General / -67.5,-27.4	Favored (6.8%) *m* chi angles: 282.1	0.08Å	-	-	-
A 552		TYR	0.73	-	Favored (79.02%) General / -62.9,-48.4	Favored (57%) *t80* chi angles: 183.5,93.7	0.06Å	-	-	-
A 553		SER	0.77	-	Favored (86.96%) General / -66.4,-38.2	Favored (89%) *p* chi angles: 62.9	0.17Å	-	-	-
A 554		ARG	0.71	-	Favored (96.35%) General / -62.3,-44.5	Favored (30.9%) *ttp-110* chi angles: 186.9,191,65.8,252	0.03Å	-	-	-
A 555		ARG	0.71	-	Favored (90.15%) General / -66.2,-40.4	Favored (83.4%) *mtm180* chi angles: 290.6,181.1,294.4,166.9	0.06Å	-	-	-
A 556		THR	0.78	-	Favored (76.7%) General / -61.6,-35.0	Favored (60.1%) *p* chi angles: 57.4	0.15Å	-	-	-

A 557		GLN	0.73	-	Favored (68.33%) General / -57.5,-33.7	Favored (4%) *tp-100* chi angles: 197.6,59.6,218.8	0.05Å	-	-	-
A 558		CYS	0.79	-	Favored (91.89%) General / -59.1,-43.1	Favored (21.2%) *t* chi angles: 190.8	0.07Å	-	-	-
A 559		PHE	0.73	-	Favored (72.36%) General / -70.0,-33.6	Favored (82.5%) *m-80* chi angles: 300.8,87.2	0.12Å	-	-	-
A 560		GLN	0.71	-	Favored (65.61%) General / -61.4,-52.2	Favored (85.3%) *mt0* chi angles: 293.2,178.9,51.6	0.04Å	-	-	-
A 561		GLU	0.7	-	Favored (91.32%) General / -64.3,-44.5	Favored (96.6%) *mt-10* chi angles: 291.9,184.2,355.1	0.03Å	-	-	-
A 562		GLN	0.69	-	Favored (85.73%) General / -57.7,-45.4	Favored (43.5%) *tt0* chi angles: 178.5,189.5,21.9	0.08Å	-	-	-
A 563		TYR	0.66	0.62Å CD2 with A 574 VAL HG21	Favored (58.75%) General / -76.1,-31.5	Favored (6.5%) *t80* chi angles: 206.3,77.4	0.20Å	-	-	-
A 564		ALA	0.66	0.56Å HB2 with A 576 ASP HB3	Favored (2.9%) General / -92.7,-56.8	-	0.06Å	-	-	-
A 565		ILE	0.48	-	Favored (28.31%) Ile or Val / -69.5,125.8	Favored (76.7%) *mt* chi angles: 301.1,171.6	0.04Å	-	-	-
A 566		PHE	0.37	-	Favored (2.58%) General / 77.4,9.3	Favored (58.8%) *m-80* chi angles: 294,115.3	0.14Å	-	-	-
A 567		VAL	0.54	-	Favored (71.02%) Ile or Val / -119.3,123.3	Favored (85.8%) *t* chi angles: 177.4	0.03Å	-	-	-
A 568		GLU	0.58	-	Favored (28.18%) General / -146.3,142.4	Favored (89.2%) *tt0* chi angles: 186.1,172.9,1.2	0.03Å	-	-	-
A 569		TYR	0.53	-	Favored (2.26%) General / -114.2,-173.3	Favored (10%) *t80* chi angles: 194.4,50.4	0.14Å	-	-	-
A 570		LYS	0.51	-	Allowed (0.57%) General / -59.0,-5.0	Favored (59%) *pttt* chi angles: 61.1,180.7,183.3,180.9	2.33Å	-	-	-

#	Alt	Res	High B	Clash > 0.4Å	Ramachandran	Rotamer	Cβ deviation	Bond lengths	Bond angles	Cis Peptides
			Avg: 0.70	Clashscore: 9.96	Outliers: 7 of 654	Poor rotamers: 8 of 581	Outliers: 7 of 633	Outliers: 2 of 656	Outliers: 48 of 656	Non-Trans: 3 of 655
A 571		GLY	0.51	-	Allowed (0.52%) Glycine / -112.4,-67.6	-	-	-	-	-
A 572		LYS	0.56	-	Allowed (0.59%) Pre-Pro / -172.0,179.8	Favored (44.2%) *pttt* chi angles: 59.9,172.1,190.5,175.9	0.03Å	-	OUTLIER(S) worst is CA-C-N: 6.3 σ	-
A 573		PRO	0.59	-	Favored (26.28%) Trans-Pro / -53.4,127.7	Favored (13.2%) *Cg_exo* chi angles: 345,29.6,326.5	0.12Å	-	-	-
A 574		VAL	0.62	0.62Å HG21 with A 563 TYR CD2	OUTLIER (0.04)%) Ile or Val / -46.6,163.3	Favored (6.5%) *m* chi angles: 307.7	0.05Å	-	-	-
A 575		GLN	0.6	-	Favored (21.81%) General / -95.4,109.7	Favored (42.5%) *mm-40* chi angles: 304.6,295.1,283.3	0.03Å	-	-	-
A 576		ASP	0.66	0.56Å HB3 with A 564 ALA HB2	Favored (78.93%) General / -62.7,-48.5	Favored (8.4%) *p0* chi angles: 52.6,306	0.12Å	-	OUTLIER(S) worst is CA-CB-CG: 4.2 σ	-
A 577		LYS	0.66	-	Favored (78.71%) General / -63.5,-48.2	Favored (98.2%) *mttt* chi angles: 292.7,182.2,178.5,180.3	0.05Å	-	-	-
A 578		ASP	0.65	-	Favored (91.15%) General / -61.5,-39.5	Favored (58.5%) *m-30* chi angles: 288.3,319.8	0.03Å	-	-	-
A 579		LEU	0.7	-	Favored (55.9%) General / -93.4,-1.4	Favored (88.5%) *mt* chi angles: 295,170.7	0.06Å	-	-	-
A 580		LEU	0.75	-	Favored (64.87%) General / -52.0,-45.9	Favored (36%) *tp* chi angles: 173.1,56.6	0.07Å	-	-	-
A 581		ARG	0.74	-	Favored (63.91%) General / -53.1,-50.7	Favored (61.1%) *mtm180* chi angles: 288.8,190.7,288.9,180.6	0.20Å	-	-	-
A 582		ARG	0.73	-	Favored (90.7%) General / -65.8,-39.2	Favored (98.9%) *mtt180* chi angles: 293.3,178.8,176.5,174.1	0.03Å	-	-	-

A 583		ILE	0.78	-	Favored (93.64%) Ile or Val / -65.4,-42.5	Favored (46.3%) *mm* chi angles: 299.6,302.5	0.10Å	-	-	-
A 584		VAL	0.78	-	Favored (98.54%) Ile or Val / -61.0,-45.5	Favored (90%) *t* chi angles: 174.2	0.04Å	-	-	-
A 585		ALA	0.79	-	Favored (74.19%) General / -60.9,-50.2	-	0.04Å	-	-	-
A 586		ASP	0.78	-	Favored (92.85%) General / -61.0,-45.9	Favored (92%) *m-30* chi angles: 286.7,172.7	0.07Å	-	-	-
A 587		ASN	0.77	-	Favored (52.53%) General / -66.0,-52.1	Favored (11.1%) *m-40* chi angles: 278.7,261.7	0.02Å	-	-	-
A 588		GLY	0.81	-	Favored (72.55%) Glycine / -68.9,-29.2	-	-	-	-	-
A 589		ALA	0.81	-	Favored (92.2%) General / -64.5,-44.1	-	0.12Å	-	-	-
A 590		LEU	0.8	-	Favored (94.26%) General / -62.8,-45.1	Favored (53.8%) *tp* chi angles: 183,60.9	0.01Å	-	-	-
**#**	**Alt**	**Res**	**High B**	**Clash > 0.4Å**	**Ramachandran**	**Rotamer**	**Cβ deviation**	**Bond lengths**	**Bond angles**	**Cis Peptides**
			Avg: 0.70	Clashscore: 9.96	Outliers: 7 of 654	Poor rotamers: 8 of 581	Outliers: 7 of 633	Outliers: 2 of 656	Outliers: 48 of 656	Non-Trans: 3 of 655
A 591		ASP	0.79	-	Favored (96.24%) General / -63.5,-43.8	Favored (59.2%) *m-30* chi angles: 283.4,3.2	0.07Å	-	-	-
A 592		ILE	0.82	-	Favored (94.03%) Ile or Val / -60.0,-46.6	Favored (78%) *mt* chi angles: 289.2,167.8	0.01Å	-	-	-
A 593		ALA	0.82	-	Favored (95.48%) General / -63.4,-39.8	-	0.04Å	-	-	-
A 594		TYR	0.8	-	Favored (97.83%) General / -62.6,-43.8	Favored (63.2%) *t80* chi angles: 186.7,73.8	0.03Å	-	-	-
					Favored	Favored (2.2%)				

A 595		ARG	0.78	-	(94.56%) General / -59.9,-44.1	*ttm170* chi angles: 223.6,168.4,294.1,139.6	0.08Å	-	-	-
A 596		ALA	0.82	-	Favored (98.14%) General / -62.2,-41.3	-	0.03Å	-	-	-
A 597		TYR	0.78	-	Favored (96.62%) General / -62.6,-44.3	Favored (10.4%) *t80* chi angles: 190.6,46.2	0.06Å	-	-	-
A 598		GLN	0.75	-	Favored (79%) General / -60.2,-37.0	Favored (76.5%) *mt0* chi angles: 292.8,185.3,295.8	0.05Å	-	-	-
A 599		GLN	0.73	-	Favored (95.81%) General / -62.5,-44.7	Favored (78.7%) *mt0* chi angles: 288.8,183,351.4	0.11Å	-	-	-
A 600		TRP	0.69	-	Favored (93.02%) General / -59.6,-45.0	Favored (64.3%) *t-100* chi angles: 182,246.5	0.03Å	-	-	-
A 601		LEU	0.68	-	Favored (31.76%) General / -65.6,-54.2	Favored (70.9%) *mt* chi angles: 301.1,172.7	0.05Å	-	-	-
A 602		LYS	0.66	-	Favored (3.91%) General / -122.3,-30.9	Favored (96.8%) *mttt* chi angles: 288.5,180.7,178.6,178.2	0.06Å	-	-	-
A 603		ASN	0.66	-	Favored (76.22%) General / -69.6,-41.0	Favored (44.3%) *t0* chi angles: 186.7,66	0.10Å	-	-	-
A 604		ALA	0.67	-	Favored (35.87%) General / -98.8,-3.3	-	0.11Å	-	-	-
A 605		ALA	0.57	-	Favored (33.72%) General / -92.0,135.7	-	0.07Å	-	-	-
A 606		GLU	0.36	-	Favored (18.94%) General / -93.9,-21.9	Favored (95.1%) *mt-10* chi angles: 297.6,177.6,349.2	0.13Å	-	-	-
A 607		THR	0.36	-	Favored (29.44%) Pre-Pro / -133.3,137.5	Favored (74.1%) *m* chi angles: 296.1	0.10Å	-	OUTLIER(S) worst is CA-C-N: 4.7 σ	-
A 608		PRO	0.56	0.40Å O with A 611 TYR CD2	Favored (53.91%) Trans-Pro / -66.4,-17.6	Favored (73%) *Cg_exo* chi angles: 335.1,32.6,332	0.06Å	-	OUTLIER(S) worst is O-C-N: 5.6 σ	-

#	Alt	Res	High B	Clash > 0.4Å	Ramachandran	Rotamer	Cβ deviation	Bond lengths	Bond angles	Cis Peptides
A 609		VAL	0.49	-	Favored (32.22%) Ile or Val / -74.0,-32.2	Favored (65.2%) *t* chi angles: 179.3	0.13Å	-	-	-
A 610		ILE	0.45	-	Favored (16.66%) Ile or Val / -74.3,-16.6	Favored (93%) *mt* chi angles: 296.4,167.9	0.09Å	-	-	-
**#**	**Alt**	**Res**	**High B**	**Clash > 0.4Å**	**Ramachandran**	**Rotamer**	**Cβ deviation**	**Bond lengths**	**Bond angles**	**Cis Peptides**
			Avg: 0.70	Clashscore: 9.96	Outliers: 7 of 654	Poor rotamers: 8 of 581	Outliers: 7 of 633	Outliers: 2 of 656	Outliers: 48 of 656	Non-Trans: 3 of 655
A 611		TYR	0.47	0.40Å CD2 with A 608 PRO O	Favored (86.63%) General / -62.1,-37.8	OUTLIER (0.1%) chi angles: 101,75.2	0.18Å	-	-	-
A 612		GLN	0.61	-	Favored (14.97%) General / -103.0,160.0	Favored (15.5%) *pt0* chi angles: 58.9,183.4,353.4	0.06Å	-	-	-
A 613		ARG	0.61	-	Favored (25.39%) General / -100.3,147.8	Favored (39.3%) *mmm160* chi angles: 299.8,298.4,295.2,175.8	0.07Å	-	-	-
A 614		GLU	0.62	-	Favored (50.32%) General / -55.5,134.0	Favored (93.1%) *mt-10* chi angles: 294.9,185.1,349.3	0.03Å	-	-	-
A 615		ARG	0.69	-	Favored (40.86%) General / -72.9,153.8	Favored (87.6%) *mtm180* chi angles: 291.6,178.6,291.5,173.2	0.06Å	-	-	-
A 616		LEU	0.75	-	Favored (91.31%) Pre-Pro / -70.2,143.6	Favored (56.3%) *mt* chi angles: 293.5,181.9	0.12Å	-	-	-
A 617		PRO	0.76	-	Favored (29.63%) Trans-Pro / -75.9,146.5	Favored (62.3%) *Cg_endo* chi angles: 31.6,323.6,27.2	0.03Å	-	-	-
A 618		LEU	0.71	-	Allowed (1.56%) General / 79.7,9.0	Favored (34.9%) *mt* chi angles: 301.2,166.8	0.10Å	-	-	-
A 619		LEU	0.73	-	Favored (15.69%) General / -133.0,115.9	Favored (38.7%) *mt* chi angles: 284.5,163.3	0.11Å	-	-	-
A 620		ASP	0.71	-	Allowed (1.39%) General / -85.3,47.2	Favored (50.7%) *m-30* chi angles: 294.1,299.7	0.07Å	-	-	-
A					Favored (51.08%)	Favored (17.8%)			OUTLIER(S) worst is ND1-	

621		HIS	0.76	-	General / -125.9,143.5	*m90* chi angles: 312.2,88.5	0.07Å	-	CG-CD2: 4.1 σ	-
A 622		ASP	0.77	-	Favored (10.07%) General / -82.8,175.5	Favored (8.9%) *p0* chi angles: 80.1,7.8	0.19Å	-	-	-
A 623		HIS	0.76	-	Favored (62.09%) General / -71.8,-18.6	Favored (41.5%) *m-70* chi angles: 277.7,283.5	0.15Å	-	-	-
A 624		ASN	0.77	-	Favored (64.25%) General / -72.8,-42.1	Favored (97.3%) *m-40* chi angles: 291.8,335	0.06Å	-	-	-
A 625		GLN	0.77	-	Favored (87.59%) General / -65.2,-44.7	Favored (98.4%) *mt0* chi angles: 292.9,176.7,329.2	0.05Å	-	-	-
A 626		ILE	0.79	-	Favored (64.82%) Ile or Val / -57.5,-38.7	Favored (63.1%) *mt* chi angles: 287.4,169.5	0.06Å	-	-	-
A 627		PHE	0.78	-	Favored (41.72%) General / -54.6,-54.4	Favored (28.7%) *t80* chi angles: 175.5,277.1	0.06Å	-	-	-
A 628		TYR	0.78	-	Favored (80.44%) General / -66.5,-35.5	Favored (50.8%) *m-80* chi angles: 287.6,113.2	0.03Å	-	-	-
A 629		LEU	0.78	-	Favored (89.65%) General / -61.9,-46.4	OUTLIER (0%) chi angles: 213.7,213.9	0.10Å	-	-	-
A 630		GLY	0.81	-	Favored (89.79%) Glycine / -66.1,-35.0	-	-	-	-	-
**#**	**Alt**	**Res**	**High B**	**Clash > 0.4Å**	**Ramachandran**	**Rotamer**	**Cβ deviation**	**Bond lengths**	**Bond angles**	**Cis Peptides**
			Avg: 0.70	Clashscore: 9.96	Outliers: 7 of 654	Poor rotamers: 8 of 581	Outliers: 7 of 633	Outliers: 2 of 656	Outliers: 48 of 656	Non-Trans: 3 of 655
A 631		TYR	0.78	-	Favored (63.75%) General / -59.9,-52.8	Favored (43.2%) *t80* chi angles: 165.1,74.5	0.06Å	-	-	-
A 632		ALA	0.78	-	Favored (81.94%) General / -63.3,-35.9	-	0.04Å	-	-	-
A					Favored	Favored (38.4%)				

633		GLN	0.73	-	(75.29%) General / -59.6,-36.0	*mt0* chi angles: 281.5,191,2.9	0.26Å	-	-	-
A 634		LEU	0.77	0.61Å HD11 with A 514 THR HA	Favored (43.07%) General / -51.8,-35.2	Favored (14.9%) *mt* chi angles: 307.1,166.3	0.14Å	-	-	-
A 635		TYR	0.76	-	Favored (23.52%) General / -106.8,17.2	Favored (59.8%) *m-80* chi angles: 295.2,115.5	0.13Å	-	-	-
A 636		CYS	0.79	0.46Å HB2 with A 83 TYR CE1	Favored (55.3%) General / -67.7,144.7	Favored (70%) *m* chi angles: 298.5	0.06Å	-	-	-
A 637		THR	0.75	0.48Å CB with A 82 ASN HB3	OUTLIER (0.03)%) General / 166.9,158.9	Favored (11.4%) *t* chi angles: 189.3	0.13Å	-	OUTLIER(S) worst is CA-CB-OG1: 4.5 σ	-
A 638		ASP	0.68	-	Favored (27.64%) General / -142.8,165.3	Favored (14.2%) *t0* chi angles: 208.8,357.1	0.05Å	-	-	-
A 639		TYR	0.6	-	Favored (54.76%) Pre-Pro / -133.1,144.9	Favored (55.7%) *m-80* chi angles: 288.1,257.5	0.10Å	-	-	-
A 640		PRO	0.66	0.47Å HD2 with A 79 THR O	Favored (38.1%) Trans-Pro / -69.2,137.8	Favored (16.5%) *Cg_exo* chi angles: 343.5,26.2,333.3	0.08Å	-	-	-
A 641		GLU	0.59	-	Favored (7.02%) General / -43.5,-47.0	Favored (71.1%) *tt0* chi angles: 184.8,188,346.6	0.05Å	-	-	-
A 642		THR	0.6	-	Favored (78.96%) General / -57.5,-40.6	Favored (97.1%) *m* chi angles: 300.7	0.02Å	-	-	-
A 643		VAL	0.6	0.53Å HG11 with A 79 THR O	Favored (21.67%) Ile or Val / -74.9,-28.8	OUTLIER (0.1%) chi angles: 91.1	0.14Å	-	-	-
A 644		GLU	0.57	-	Favored (91.65%) General / -62.1,-39.2	Favored (39.9%) *mt-10* chi angles: 296.9,196.6,323.5	0.04Å	-	-	-
A 645		ARG	0.49	-	Favored (72.62%) General / -60.3,-33.6	Favored (94.6%) *mtt-85* chi angles: 295.7,179.9,187.5,281.5	0.09Å	-	-	-
A 646		PHE	0.52	-	Allowed (0.87%) General / -68.0,5.7	Favored (6.6%) *t80* chi angles: 167.3,97.9	0.19Å	-	-	-

A 647		ASP	0.42	-	Allowed (0.05%) General / 84.8,30.7	Allowed (0.5%) *t70* chi angles: 215.7,57.4	0.25Å	-	-	-
A 648		GLU	0.42	-	Favored (53.75%) General / -61.9,145.2	Favored (61.7%) *mm-30* chi angles: 288.2,286.5,351	0.12Å	-	-	-
A 649		LEU	0.53	-	Favored (7.87%) Pre-Pro / -95.4,101.5	Favored (10.6%) *tp* chi angles: 167.3,55.1	0.07Å	-	OUTLIER(S) worst is CA-C-N: 4.1 σ	-
A 650		PRO	0.7	-	Favored (10.48%) Trans-Pro / -67.6,123.2	Favored (45.9%) *Cg_endo* chi angles: 33.5,321.3,29.1	0.06Å	-	-	-
**#**	**Alt**	**Res**	**High B**	**Clash > 0.4Å**	**Ramachandran**	**Rotamer**	**Cβ deviation**	**Bond lengths**	**Bond angles**	**Cis Peptides**
			Avg: 0.70	Clashscore: 9.96	Outliers: 7 of 654	Poor rotamers: 8 of 581	Outliers: 7 of 633	Outliers: 2 of 656	Outliers: 48 of 656	Non-Trans: 3 of 655
A 651		GLU	0.72	-	Favored (69.4%) General / -59.0,-32.1	Favored (27.5%) *mp0* chi angles: 282.5,87,339.4	0.09Å	-	-	-
A 652		GLN	0.71	-	Favored (69.38%) General / -57.5,-34.8	Favored (7.7%) *pt0* chi angles: 68.1,177.9,258.3	0.10Å	-	-	-
A 653		LEU	0.71	-	Favored (35.24%) General / -81.8,-25.4	Favored (92.4%) *mt* chi angles: 294.5,176.2	0.04Å	-	-	-
A 654		ARG	0.71	-	Favored (98.95%) General / -63.1,-40.9	Favored (41%) *mtp180* chi angles: 303,176.8,79.5,166	0.07Å	-	-	-
A 655		VAL	0.77	-	Favored (10.93%) Ile or Val / -62.1,-57.7	Favored (74%) *t* chi angles: 178.4	0.01Å	-	-	-
A 656		ASN	0.72	-	Favored (90.45%) General / -65.4,-43.5	Favored (92.8%) *m-40* chi angles: 289.5,330.4	0.06Å	-	-	-
A 657		THR	0.72	-	Favored (86.28%) General / -64.4,-45.8	Favored (33.3%) *m* chi angles: 293	0.04Å	-	-	-
A 658		ALA	0.78	-	Favored (86.39%) General / -65.1,-45.1	-	0.02Å	-	-	-
					Favored					

A 659		LEU	0.77	-	(70.07%) General / -71.1,-33.3	Favored (74.9%) *mt* chi angles: 302.5,180.2	0.17Å	-	-	-
A 660		SER	0.77	-	Favored (71.9%) General / -58.1,-35.7	Favored (13.8%) *m* chi angles: 285.3	0.07Å	-	-	-
A 661		ASN	0.76	-	Favored (58.57%) General / -84.8,-9.0	Favored (91.3%) *m-40* chi angles: 291.1,326.7	0.08Å	-	-	-
A 662		SER	0.79	-	Favored (23.53%) General / -93.2,111.8	Favored (28.5%) *t* chi angles: 173.1	0.04Å	-	-	-
A 663		GLN	0.74	-	Favored (88.72%) General / -66.0,-38.5	Favored (87.1%) *tp40* chi angles: 185.9,58.2,48.8	0.03Å	-	-	-
A 664		GLN	0.77	-	Favored (87.11%) General / -58.7,-41.7	Favored (15.9%) *mp10* chi angles: 303.8,86.8,317.8	0.03Å	-	-	-
A 665		PHE	0.78	-	Favored (81.17%) General / -60.5,-48.6	Favored (61.7%) *t80* chi angles: 170.3,263.6	0.07Å	-	-	-
A 666		ALA	0.8	-	Favored (93.15%) General / -63.4,-39.0	-	0.07Å	-	-	-
A 667		ASN	0.78	-	Favored (96.26%) General / -62.0,-44.7	Favored (80.2%) *m-40* chi angles: 297,339.4	0.05Å	-	-	-
A 668		ALA	0.81	-	Favored (91.44%) General / -62.4,-38.9	-	0.02Å	-	-	-
A 669		TYR	0.78	-	Favored (30.45%) General / -105.7,13.0	Favored (28.5%) *m-80* chi angles: 277.4,100.1	0.02Å	-	-	-
A 670		GLY	0.82	-	Favored (50.37%) Glycine / 51.0,46.4	-	-	-	-	-
**#**	**Alt**	**Res**	**High B**	**Clash > 0.4Å**	**Ramachandran**	**Rotamer**	**Cβ deviation**	**Bond lengths**	**Bond angles**	**Cis Peptides**
			Avg: 0.70	Clashscore: 9.96	Outliers: 7 of 654	Poor rotamers: 8 of 581	Outliers: 7 of 633	Outliers: 2 of 656	Outliers: 48 of 656	Non-Trans: 3 of 655

Favored

A 671		CYS	0.81	-	(28.55%) General / -79.0,124.7	Favored (62.2%) *m* chi angles: 301	0.06Å	-	-	-
A 672		SER	0.78	-	Favored (18.69%) General / -58.7,152.4	Favored (22.5%) *t* chi angles: 185.3	0.11Å	-	-	-
A 673		ARG	0.69	-	Favored (40.79%) General / -63.9,129.6	Favored (85.7%) *mtm180* chi angles: 297,178.6,293.5,168.1	0.07Å	-	-	-
A 674		GLU	0.68	-	Allowed (1.49%) General / 79.4,10.4	Favored (44.5%) *mt-10* chi angles: 299.9,177.8,47.3	0.27Å	-	-	-
A 675		GLU	0.7	-	Favored (20.74%) General / -62.2,156.9	Favored (74.5%) *mm-30* chi angles: 301.8,293.9,328.8	0.09Å	-	-	-
A 676		LYS	0.7	-	Favored (70.09%) General / -56.9,-36.4	Favored (78.2%) *tttt* chi angles: 183.5,175.2,170.5,187	0.08Å	-	-	-
A 677		LEU	0.72	-	Favored (42.1%) General / -92.4,7.7	Favored (3.1%) *mm* chi angles: 274.9,289.6	0.09Å	-	-	-
A 678		ASN	0.73	-	Allowed (1.62%) General / -140.0,56.9	Favored (52.2%) *t0* chi angles: 194.1,21.4	0.10Å	-	-	-
A 679		ALA	0.72	-	Favored (55.44%) General / -59.2,140.9	-	0.03Å	-	-	-
A 680		ARG	0.65	-	Favored (97.25%) General / -63.8,-40.7	Favored (75.9%) *ttm-80* chi angles: 190,181.2,297,273.6	0.04Å	-	-	-
A 681		PHE	0.64	-	Favored (23.34%) General / -81.7,119.0	Favored (96.6%) *m-80* chi angles: 292.8,93.9	0.06Å	-	-	-
A 682		LYS	0.7	-	Favored (54.02%) General / -124.7,136.6	Favored (44%) *mttm* chi angles: 297.9,191.9,190.2,306.4	0.03Å	-	-	-
A 683		CYS	0.79	-	Favored (31.71%) General / -92.4,138.2	Favored (40.2%) *m* chi angles: 304.5	0.07Å	-	-	-
A 684		THR	0.77	-	Favored (29.82%) General /	Allowed (1.9%) *t* chi angles: 173.9	0.14Å	-	-	-

					-147.1,146.1					
A 685		LEU	0.77	-	Allowed (0.63%) General / -138.6,-58.3	Favored (6%) *tt* chi angles: 188.7,146	0.11Å	-	-	-
A 686		TYR	0.76	-	-	Favored (30%) *m-80* chi angles: 294.3,309.8	0.10Å	-	-	-

C

Viewing model03FH-multi.table

Duke Biochemistry
Duke University School of Medicine

When finished, you should close this window .

Hint: Use File | Save As... to save a copy of this page.

All-Atom Contacts	Clashscore, all atoms:	10.3		70th percentile* (N=1784, all resolutions)
	Clashscore is the number of serious steric overlaps (> 0.4 Å) per 1000 atoms.			
Protein Geometry	Poor rotamers	11	1.91%	Goal: <0.3%
	Favored rotamers	547	94.80%	Goal: >98%
	Ramachandran outliers	12	1.85%	Goal: <0.05%
	Ramachandran favored	597	91.85%	Goal: >98%
	MolProbity score^	2.24		62nd percentile* (N=27675, 0Å - 99Å)
	Cβ deviations >0.25Å	10	1.59%	Goal: 0
	Bad bonds:	1 / 5589	0.02%	Goal: 0%
	Bad angles:	88 / 7572	1.16%	Goal: <0.1%
Peptide Omegas	Cis Prolines:	0 / 24	0.00%	Expected: ≤1 per chain, or ≤5%
	Cis nonProlines:	1 / 627	0.16%	Goal: <0.05%

In the two column results, the left column gives the raw count, right column gives the percentage.

* 100th percentile is the best among structures of comparable resolution; 0th percentile is the worst. For clashscore the comparative set of structures was selected in 2004, for MolProbity score in 2006.

^ MolProbity score combines the clashscore, rotamer, and Ramachandran evaluations into a single score, normalized to be on the same scale as X-ray resolution.

#	Alt	Res	High B	Clash > 0.4Å	Ramachandran	Rotamer	Cβ deviation	Bond lengths	Bond angles	Cis Peptides
			Avg: 0.70	Clashscore: 10.3	Outliers: 12 of 650	Poor rotamers: 11 of 577	Outliers: 10 of 629	Outliers: 1 of 652	Outliers: 75 of 652	Non-Trans: 1 of 651
A 35		ASP	0.71	-	-	Favored (5.2%) *m-30* chi angles: 302.9,359.8	0.19Å	-	OUTLIER(S) worst is CA-CB-CG: 4.8 σ	-
A 36		ILE	0.67	-	Favored (34.48%) Ile or Val / -53.7,-38.2	Favored (18.7%) *tt* chi angles: 195.4,167	0.18Å	-	-	-
A 37		LEU	0.7	-	Favored (93.36%) General / -59.5,-44.0	Favored (73%) *mt* chi angles: 300.8,172.9	0.02Å	-	-	-
A 38		ARG	0.7	-	Favored (96.15%) General /	Favored (98.3%) *mtt180* chi angles:	0.08Å	-	-	-

					-64.4,-42.5	291.9,176.6,181,180.7				
A 39		LEU	0.71	-	Favored (87.7%) General / -63.5,-46.1	Favored (88.5%) *mt* chi angles: 295.4,171	0.03Å	-	-	-
A 40		ALA	0.77	-	Favored (90.32%) General / -62.5,-46.0	-	0.03Å	-	-	-
A 41		LYS	0.76	-	Favored (99.26%) General / -62.1,-42.4	Favored (48.1%) *tptt* chi angles: 187.7,60.2,175,182.6	0.08Å	-	-	-
A 42		ALA	0.79	-	Favored (91.74%) General / -60.2,-40.9	-	0.03Å	-	-	-
A 43		ALA	0.78	-	Favored (95.97%) General / -62.4,-44.6	-	0.01Å	-	-	-
A 44		HIS	0.73	-	Favored (92.73%) General / -65.5,-40.0	Favored (57.9%) *m170* chi angles: 296.6,166.3	0.11Å	-	OUTLIER(S) worst is ND1-CG-CD2: 4.3 σ	-
A 45		ILE	0.76	-	Favored (79.65%) Ile or Val / -60.0,-50.6	Favored (78.7%) *mt* chi angles: 291,163.7	0.03Å	-	-	-
A 46		LYS	0.74	-	Favored (93.17%) General / -63.3,-39.0	Favored (87.9%) *tttt* chi angles: 183.9,176.5,179,177.7	0.06Å	-	-	-
A 47		ALA	0.76	-	Favored (70.74%) General / -61.0,-31.1	-	0.15Å	-	OUTLIER(S) worst is C-CA-CB: 4.1 σ	-
A 48		TYR	0.71	-	Favored (40.41%) General / -82.2,-19.2	Favored (5.6%) *m-10* chi angles: 293.5,4.5	0.13Å	-	-	-
A 49		MET	0.71	-	Favored (20.92%) General / -93.6,149.8	Allowed (1.7%) *ptm* chi angles: 39.2,199.3,306	0.25Å	-	-	-
A 50		GLY	0.71	-	Allowed (1.02%) Glycine / -107.4,47.7	-	-	-	-	-
A 51		ASP	0.66	-	Favored (88.26%) General / -61.2,-38.9	Favored (17.3%) *p0* chi angles: 51.2,1.6	0.17Å	-	-	-
A		GLU	0.43	-	Favored (28.01%)	Favored (95.3%) *mt-10*	0.12Å	-	-	-

#	Alt	Res	High B	Clash > 0.4Å	Ramachandran	Rotamer	Cβ deviation	Bond lengths	Bond angles	Cis Peptides
52					General / -80.0,1.3	chi angles: 298.5,180.5,353				
A 53		LYS	0.67	-	Favored (4.14%) General / -117.0,-35.2	Favored (71.9%) *mmtt* chi angles: 299.3,298.7,182.4,182.5	0.11Å	-	-	-
A 54		GLU	0.67	-	Favored (53.84%) General / -125.0,139.4	Favored (7.4%) *tp30* chi angles: 180.9,72.1,72	0.07Å	-	-	-
**#**	**Alt**	**Res**	**High B**	**Clash > 0.4Å**	**Ramachandran**	**Rotamer**	**Cβ deviation**	**Bond lengths**	**Bond angles**	**Cis Peptides**
			Avg: 0.70	Clashscore: 10.3	Outliers: 12 of 650	Poor rotamers: 11 of 577	Outliers: 10 of 629	Outliers: 1 of 652	Outliers: 75 of 652	Non-Trans: 1 of 651
A 55		GLN	0.68	-	Favored (34.91%) General / -85.6,128.9	Favored (62.7%) *tp40* chi angles: 172.6,61,49.1	0.04Å	-	-	-
A 56		ALA	0.78	-	Allowed (1.54%) General / -56.2,-12.4	-	0.06Å	-	-	-
A 57		CYS	0.8	-	Favored (20.6%) General / -105.7,-4.8	Favored (90.5%) *m* chi angles: 291.3	0.04Å	-	-	-
A 58		ASN	0.73	-	Favored (4.66%) General / -95.5,-51.2	Favored (3.8%) *t0* chi angles: 176.4,89.2	0.08Å	-	-	-
A 59		ASN	0.72	-	Favored (3.51%) General / -150.1,106.6	Favored (55.3%) *t0* chi angles: 186.4,329.2	0.06Å	-	-	-
A 60		PHE	0.71	0.40Å N with A 677 LEU HD11	Favored (57.96%) General / -76.0,-29.1	Favored (35.6%) *t80* chi angles: 193.7,77	0.07Å	-	-	-
A 61		TYR	0.71	-	Favored (83.15%) General / -63.8,-46.9	Favored (73.1%) *t80* chi angles: 184.8,76.1	0.04Å	-	-	-
A 62		ASN	0.7	-	Favored (97.57%) General / -63.4,-40.5	Favored (74%) *m-40* chi angles: 288.5,355.6	0.07Å	-	-	-
A 63		TYR	0.69	-	Favored (84.75%) General / -61.3,-47.7	Favored (69.4%) *t80* chi angles: 170.2,80.1	0.12Å	-	-	-
A 64		ALA	0.78	-	Favored (40.34%) General /	-	0.11Å	-	-	-

					-79.9,-32.9					
A 65		CYS	0.78	0.47Å O with A 69 PRO HD2	Favored (7.52%) General / -110.6,-30.7	Favored (98.7%) *m* chi angles: 292.4	0.09Å	-	OUTLIER(S) worst is CA-C-N: 4.4 σ	-
A 66		ALA	0.73	-	Allowed (1.04%) General / -45.8,-28.3	-	0.14Å	-	OUTLIER(S) worst is C-CA-CB: 4.2 σ	-
A 67		ASN	0.7	-	Favored (81.52%) General / -67.4,-43.4	Favored (8.8%) *t0* chi angles: 206.2,328.4	0.12Å	-	-	-
A 68		TRP	0.68	-	Favored (98.75%) Pre-Pro / -57.8,-47.5	Favored (64.9%) *t60* chi angles: 190.5,95.6	0.17Å	-	OUTLIER(S) worst is CA-C-N: 5.8 σ	-
A 69		PRO	0.69	0.47Å HD2 with A 65 CYS O	Favored (77.25%) Trans-Pro / -58.1,-40.4	Favored (88.9%) *Cg_exo* chi angles: 330.8,34.8,331	0.07Å	-	-	-
A 70		ARG	0.67	-	Favored (77.64%) General / -69.5,-38.5	Favored (98%) *mtm-85* chi angles: 289.8,196,296.4,267.8	0.08Å	-	-	-
A 71		LEU	0.66	-	Favored (21.35%) General / -78.8,-45.3	Favored (60%) *tp* chi angles: 180.8,59	0.05Å	-	-	-
A 72		HIS	0.66	-	Favored (69.05%) Pre-Pro / -90.6,119.3	Favored (72.9%) *t-90* chi angles: 187.9,274	0.07Å	-	-	-
A 73		PRO	0.66	-	Allowed (1.39%) Trans-Pro / -96.0,152.4	Favored (46.3%) *Cg_endo* chi angles: 33.4,331.7,12.2	0.08Å	-	-	-
A 74		ALA	0.69	-	Allowed (0.23%) General / -57.6,105.3	-	0.05Å	-	-	-
**#**	**Alt**	**Res**	**High B**	**Clash > 0.4Å**	**Ramachandran**	**Rotamer**	**Cβ deviation**	**Bond lengths**	**Bond angles**	**Cis Peptides**
			Avg: 0.70	Clashscore: 10.3	Outliers: 12 of 650	Poor rotamers: 11 of 577	Outliers: 10 of 629	Outliers: 1 of 652	Outliers: 75 of 652	Non-Trans: 1 of 651
A 75		ARG	0.67	-	Favored (45.33%) General / -65.8,152.0	Favored (65%) *mtm180* chi angles: 298.1,191.8,295.3,169.7	0.07Å	-	-	-
A 76		LYS	0.57	-	Favored (22.67%) General / -64.1,-10.5	Favored (98.3%) *mttt* chi angles: 295.9,179.1,178.3,178.6	0.08Å	-	-	-

A 77		SER	0.52	-	Favored (61.91%) General / -71.4,-21.9	Favored (11%) *p* chi angles: 50.3	0.08Å	-	-	-
A 78		LYS	0.52	0.43Å HG3 with A 85 GLU HG3	Favored (28.87%) General / -148.1,146.7	Favored (55.3%) *mmtt* chi angles: 282.7,285.8,184.8,184.3	0.15Å	-	-	-
A 79		THR	0.65	0.42Å HB with A 640 PRO HG2	Favored (54.55%) General / -72.6,-46.4	Favored (44.6%) *p* chi angles: 55.3	0.18Å	-	-	-
A 80		ARG	0.68	-	Favored (55.35%) General / -120.0,132.1	Favored (17.3%) *ptm160* chi angles: 65.3,186.6,302.5,172.8	0.04Å	-	-	-
A 81		THR	0.7	-	Favored (38.31%) General / -139.0,140.8	Favored (21.9%) *p* chi angles: 51.1	0.06Å	-	-	-
A 82		ASN	0.71	-	OUTLIER (0.03)%) General / 177.2,-163.7	Favored (20.1%) *p0* chi angles: 52.4,37.2	0.07Å	-	-	-
A 83		TYR	0.67	0.52Å CE1 with A 500 PHE HB2	Favored (43.45%) General / -50.7,-52.0	Favored (7.9%) *t80* chi angles: 174.6,104.8	0.19Å	-	OUTLIER(S) worst is N-CA-CB: 4.1 σ	-
A 84		LEU	0.73	-	Favored (72.55%) General / -70.5,-41.4	Favored (90.1%) *mt* chi angles: 291.6,169.7	0.06Å	-	-	-
A 85		GLU	0.71	0.43Å HG3 with A 78 LYS HG3	Favored (38.19%) General / -56.9,-55.1	Favored (2.1%) *tm-30* chi angles: 177.8,255.9,322	0.12Å	-	OUTLIER(S) worst is CB-CG-CD: 4.0 σ	-
A 86		GLU	0.7	-	Favored (83.45%) General / -57.5,-46.6	Favored (54%) *tp30* chi angles: 176.2,62.8,32.2	0.14Å	-	-	-
A 87		LEU	0.74	0.67Å HD11 with A 498 ALA HB2	Favored (78.5%) General / -68.8,-41.6	OUTLIER (0%) chi angles: 219,211.7	0.02Å	-	-	-
A 88		GLN	0.74	-	Favored (81.98%) General / -57.5,-42.0	Favored (83.7%) *mt0* chi angles: 283.8,170.8,349.7	0.04Å	-	-	-
A 89		GLU	0.73	-	Favored (89.6%) General / -65.1,-38.1	Allowed (1.1%) *mm-30* chi angles: 304.5,263.1,314.1	0.06Å	-	-	-
A 90		LEU	0.75	-	Favored (89.14%) General /	Favored (60.5%) *tp* chi angles: 181.3,60.9	0.06Å	-	-	-

					-58.8,-42.6					
A 91		TYR	0.74	-	Favored (98.19%) General / -63.6,-42.9	Favored (22.7%) *m-10* chi angles: 295.7,322.4	0.09Å	-	-	-
A 92		ILE	0.77	-	Favored (69.11%) Ile or Val / -55.3,-42.9	Favored (40.3%) *mt* chi angles: 285.2,172.3	0.09Å	-	-	-
A 93		ARG	0.71	-	Favored (95.91%) General / -63.3,-39.9	Favored (98.9%) *mtt180* chi angles: 292.4,178.7,178.1,175.9	0.07Å	-	-	-
A 94		LYS	0.75	-	Favored (97.96%) General / -63.8,-41.0	Favored (72.3%) *mmtt* chi angles: 300.4,296.8,182.1,180.7	0.05Å	-	-	-
**#**	**Alt**	**Res**	**High B**	**Clash > 0.4Å**	**Ramachandran**	**Rotamer**	**Cβ deviation**	**Bond lengths**	**Bond angles**	**Cis Peptides**
			Avg: 0.70	Clashscore: 10.3	Outliers: 12 of 650	Poor rotamers: 11 of 577	Outliers: 10 of 629	Outliers: 1 of 652	Outliers: 75 of 652	Non-Trans: 1 of 651
A 95		SER	0.78	-	Favored (70.52%) General / -60.7,-31.2	Favored (71.8%) *m* chi angles: 295.9	0.10Å	-	-	-
A 96		ALA	0.76	-	Favored (81.44%) General / -60.5,-48.5	-	0.01Å	-	-	-
A 97		ASP	0.7	-	Favored (90.61%) General / -65.3,-38.6	Favored (89.5%) *m-30* chi angles: 293.3,346.6	0.05Å	-	-	-
A 98		MET	0.71	-	Favored (96.56%) General / -62.0,-44.5	Favored (49.6%) *mmp* chi angles: 293.5,297.7,102.5	0.04Å	-	-	-
A 99		LEU	0.71	-	Favored (77.62%) General / -68.5,-43.2	Favored (70.8%) *mt* chi angles: 287.1,170.4	0.08Å	-	-	-
A 100		LYS	0.65	-	Favored (2.08%) General / -79.3,56.4	Favored (98.4%) *mttt* chi angles: 295.9,183,179.2,177.6	0.05Å	-	-	-
A 101		SER	0.66	-	Favored (28.12%) General / -85.1,144.6	Favored (14.4%) *t* chi angles: 168.3	0.10Å	-	OUTLIER(S) worst is CA-CB-OG: 4.5 σ	-
A 102		ALA	0.51	-	Favored (56.21%) General / -94.0,0.7	-	0.07Å	-	-	-

#	Alt	Res	High B	Clash > 0.4Å	Ramachandran	Rotamer	Cβ deviation	Bond lengths	Bond angles	Cis Peptides
A 103		THR	0.48	-	Allowed (1.46%) General / -124.5,53.1	Favored (24.4%) *p* chi angles: 51.7	0.07Å	-	-	-
A 104		ARG	0.52	-	Favored (72.48%) General / -59.3,-34.5	Favored (96.3%) *mtt180* chi angles: 289,181.9,176.6,171	0.10Å	-	-	-
A 105		GLY	0.47	-	Favored (72.77%) Glycine / -65.5,-27.6	-	-	-	-	-
A 106		ALA	0.56	-	Favored (76.3%) General / -68.9,-43.0	-	0.04Å	-	-	-
A 107		GLU	0.65	-	Favored (45%) General / -72.1,149.2	Favored (47.1%) *mm-30* chi angles: 303.5,297.6,354.3	0.13Å	-	OUTLIER(S) worst is CB-CG-CD: 4.1 σ	-
A 108		ASN	0.69	-	Favored (2.96%) General / -60.4,166.6	Favored (83.9%) *m-40* chi angles: 292,316.8	0.09Å	-	-	-
A 109		SER	0.74	-	Favored (25.75%) General / -48.9,-52.2	Favored (14.6%) *t* chi angles: 188.6	0.11Å	-	-	-
A 110		ALA	0.76	-	Favored (83.75%) General / -59.8,-47.9	-	0.12Å	-	-	-
A 111		ASP	0.75	-	Favored (96.45%) General / -60.8,-41.8	Favored (7.3%) *m-30* chi angles: 296,266.2	0.12Å	-	-	-
A 112		ARG	0.67	-	Favored (98.73%) General / -62.4,-41.5	Favored (2.5%) *ttm-80* chi angles: 203.1,207.2,333,275.7	0.11Å	-	-	-
A 113		GLN	0.74	-	Favored (71.67%) General / -57.6,-50.9	Favored (55.8%) *mt0* chi angles: 280.2,180.2,38.4	0.09Å	-	-	-
A 114		LEU	0.77	0.65Å HD21 with A 365 LEU HG	Favored (81.28%) General / -58.5,-39.9	Favored (13.8%) *tp* chi angles: 187.9,50	0.10Å	-	-	-
**#**	**Alt**	**Res**	**High B**	**Clash > 0.4Å**	**Ramachandran**	**Rotamer**	**Cβ deviation**	**Bond lengths**	**Bond angles**	**Cis Peptides**
			Avg: 0.70	Clashscore: 10.3	Outliers: 12 of 650	Poor rotamers: 11 of 577	Outliers: 10 of 629	Outliers: 1 of 652	Outliers: 75 of 652	Non-Trans: 1 of 651

A 115		LYS	0.73	-	Favored (71.57%) General / -57.7,-36.2	Favored (99.2%) *mttt* chi angles: 293.6,180,180.9,175.9	0.04Å	-	-	-
A 116		TYR	0.7	-	Favored (90.44%) General / -66.2,-40.7	Favored (25.6%) *m-10* chi angles: 296.8,328	0.08Å	-	-	-
A 117		PHE	0.75	0.47Å HB2 with A 442 LEU HD13	Favored (89.61%) General / -61.7,-46.5	Favored (45.4%) *t80* chi angles: 187.8,65.6	0.08Å	-	-	-
A 118		TYR	0.73	-	Favored (95.25%) General / -63.9,-43.7	Favored (24%) *t80* chi angles: 185.2,52.2	0.04Å	-	-	-
A 119		GLY	0.75	-	Favored (99.1%) Glycine / -62.9,-43.3	-	-	-	-	-
A 120		SER	0.71	-	Favored (99.58%) General / -61.3,-43.0	Favored (87.8%) *p* chi angles: 68.1	0.22Å	-	-	-
A 121		CYS	0.77	-	Favored (52.23%) General / -77.0,-23.0	Favored (27.7%) *p* chi angles: 64.3	0.03Å	-	-	-
A 122		ARG	0.65	-	Favored (40.46%) General / -80.5,-23.1	Favored (21%) *tpp-160* chi angles: 177.6,58.5,72.4,198.7	0.04Å	-	-	-
A 123		LEU	0.65	-	Favored (2.02%) General / -76.0,65.4	Favored (94.1%) *mt* chi angles: 291.6,172.1	0.07Å	-	-	-
A 124		GLN	0.6	-	Favored (55.26%) General / -81.0,-4.5	Favored (97.4%) *mt0* chi angles: 292.9,179.4,334.3	0.03Å	-	-	-
A 125		THR	0.6	-	Favored (84.87%) General / -60.0,-39.2	Allowed (1.4%) *m* chi angles: 282.4	0.02Å	-	-	-
A 126		ASN	0.64	-	Favored (68.88%) General / -70.2,-44.4	Favored (88.4%) *m-40* chi angles: 295.5,328.9	0.04Å	-	OUTLIER(S) worst is CA-CB-CG: 4.8 σ	-
A 127		ASP	0.65	-	Favored (93.13%) General / -60.4,-45.5	Favored (79%) *m-30* chi angles: 295.9,331.5	0.07Å	-	-	-
A 128		THR	0.67	-	Favored (66.63%) General /	Favored (65.5%) *p* chi angles: 58.2	0.16Å	-	-	-

#	Alt	Res	High B	Clash > 0.4Å	Ramachandran	Rotamer	Cβ deviation	Bond lengths	Bond angles	Cis Peptides
					-57.6,-31.8					
A 129		ARG	0.59	-	Favored (59.54%) General / -75.7,-18.5	Favored (94.3%) *mtt180* chi angles: 292.7,169.4,182.1,180.6	0.04Å	-	-	-
A 130		SER	0.67	-	Favored (20.41%) General / -87.8,-30.1	Favored (48.6%) *p* chi angles: 74.9	0.14Å	-	-	-
A 131		ALA	0.65	0.66Å HA with A 343 VAL HG11	Allowed (1.42%) General / 54.9,-135.4	-	0.23Å	-	-	-
A 132		LEU	0.65	-	Favored (48.41%) General / -90.4,-9.2	Favored (23.1%) *mt* chi angles: 302.6,165.3	0.17Å	-	-	-
A 133		ASN	0.66	-	Favored (7.18%) General / -45.8,-38.4	Favored (92.9%) *m-40* chi angles: 288.4,331.2	0.10Å	-	-	-
A 134		THR	0.68	-	Favored (3.22%) General / -55.4,-60.6	Favored (48.5%) *p* chi angles: 55.9	0.04Å	-	-	-
#	Alt	Res	High B	Clash > 0.4Å	Ramachandran	Rotamer	Cβ deviation	Bond lengths	Bond angles	Cis Peptides
			Avg: 0.70	Clashscore: 10.3	Outliers: 12 of 650	Poor rotamers: 11 of 577	Outliers: 10 of 629	Outliers: 1 of 652	Outliers: 75 of 652	Non-Trans: 1 of 651
A 135		LEU	0.71	0.46Å HD11 with A 335 LEU HG	Favored (83.54%) General / -67.9,-40.6	OUTLIER (0%) chi angles: 211.1,200.3	0.04Å	-	-	-
A 136		GLN	0.66	0.51Å HA with A 231 PHE CE1	Favored (89.07%) General / -59.0,-42.0	Favored (56.5%) *tt0* chi angles: 189.7,180.7,48.8	0.22Å	-	-	-
A 137		ASN	0.69	-	Favored (95.82%) General / -64.6,-42.1	Favored (95.4%) *m-40* chi angles: 291.7,331.8	0.06Å	-	-	-
A 138		VAL	0.73	-	Favored (82.16%) Ile or Val / -59.9,-40.2	Favored (26%) *t* chi angles: 165	0.04Å	-	-	-
A 139		THR	0.75	-	Favored (90.17%) General / -58.7,-44.4	Favored (19.6%) *m* chi angles: 290.4	0.12Å	-	-	-
A 140		ASP	0.72	-	Favored (63.26%) General / -72.6,-29.1	Favored (69.3%) *m-30* chi angles: 293.2,355	0.10Å	-	-	-

A 141		PHE	0.66	-	Favored (70.49%) General / -64.7,-29.5	Favored (71.2%) *t80* chi angles: 185.7,79.5	0.06Å	-	-	-
A 142		ARG	0.68	-	Favored (58.67%) General / -82.6,-11.0	Favored (88.8%) *mmt-90* chi angles: 290.6,290,185.2,268.1	0.03Å	-	-	-
A 143		GLY	0.73	-	Favored (19.81%) Glycine / 112.5,9.3	-	-	-	-	-
A 144		GLY	0.75	-	Favored (53.55%) Glycine / 82.6,-176.9	-	-	-	-	-
A 145		TRP	0.65	-	Favored (15.96%) Pre-Pro / -158.3,148.9	Allowed (0.5%) *p-90* chi angles: 64,343.8	0.20Å	-	-	-
A 146		PRO	0.62	0.79Å CG with A 324 LEU HD11	Allowed (0.13%) Trans-Pro / -35.7,-34.9	Favored (54.9%) *Cg_exo* chi angles: 337.5,33.1,326.5	0.30Å	OUTLIER(S) worst is N--CD: 4.2 σ	OUTLIER(S) worst is C-CA-CB: 5.7 σ	-
A 147		GLU	0.59	0.48Å HB3 with A 232 PRO CG	Favored (66.86%) General / -56.6,-34.0	Favored (14.4%) *mp0* chi angles: 306.6,88.1,324.8	0.27Å	-	OUTLIER(S) worst is C-CA-CB: 5.6 σ	-
A 148		ILE	0.56	-	Favored (2.11%) Ile or Val / -128.4,93.3	Favored (22.6%) *tt* chi angles: 186.7,166.6	0.11Å	-	OUTLIER(S) worst is O-C-N: 5.6 σ	-
A 149		ARG	0.52	-	Favored (24.15%) General / -120.8,160.1	Favored (30.8%) *mtp180* chi angles: 287.5,205.2,68.8,195.6	0.17Å	-	-	Cis nonPRO omega= -14.4
A 150		VAL	0.44	0.43Å HA with A 153 TRP CD1	Favored (25.19%) Ile or Val / -63.2,-20.1	Favored (21.9%) *m* chi angles: 301.7	0.21Å	-	-	-
A 151		ALA	0.57	-	Favored (48.09%) General / -82.2,-16.9	-	0.06Å	-	-	-
A 152		SER	0.47	-	Favored (8.4%) General / -124.4,3.2	Favored (12.9%) *p* chi angles: 51	0.03Å	-	-	-
A 153		TRP	0.55	0.50Å CE2 with A 324 LEU HD22	Favored (58.79%) General / -83.0,-10.4	Favored (58.8%) *p-90* chi angles: 53.7,260.9	0.16Å	-	OUTLIER(S) worst is CB-CG-CD2: 4.7 σ	-
A 154		TYR	0.51	0.40Å HA with A 153 TRP CE3	Favored (72.35%) General / -57.9,-36.4	Favored (82.9%) *t80* chi angles: 180.7,84.2	0.18Å	-	-	-

#	Alt	Res	High B	Clash > 0.4Å	Ramachandran	Rotamer	Cβ deviation	Bond lengths	Bond angles	Cis Peptides
			Avg: 0.70	Clashscore: 10.3	Outliers: 12 of 650	Poor rotamers: 11 of 577	Outliers: 10 of 629	Outliers: 1 of 652	Outliers: 75 of 652	Non-Trans: 1 of 651
A 155		GLN	0.45	-	Favored (24.29%) General / -65.2,-9.6	Favored (25%) *pt0* chi angles: 70,182.3,300.6	0.10Å	-	-	-
A 156		TYR	0.45	-	Favored (9.12%) General / -86.8,13.3	Favored (6.5%) *m-10* chi angles: 310.4,332.8	0.08Å	-	-	-
A 157		GLU	0.51	-	Favored (64.4%) General / -67.9,-17.2	Favored (12.1%) *pt0* chi angles: 68.2,181.3,318.2	0.08Å	-	-	-
A 158		TYR	0.5	-	Favored (25.62%) General / -101.7,16.5	Favored (38.8%) *m-80* chi angles: 296.6,124.6	0.05Å	-	-	-
A 159		ASP	0.69	-	Favored (19.89%) General / -108.6,-1.0	Favored (40%) *p0* chi angles: 71.9,7.6	0.06Å	-	-	-
A 160		TRP	0.7	-	Favored (50.49%) General / -58.0,-54.1	Favored (20.2%) *t60* chi angles: 192.4,78	0.06Å	-	-	-
A 161		LEU	0.75	-	Favored (60.39%) General / -73.6,-21.1	Favored (11.3%) *tp* chi angles: 196.5,59.9	0.08Å	-	-	-
A 162		GLN	0.75	-	Favored (64.13%) General / -69.3,-47.6	Favored (90.9%) *mt0* chi angles: 299.5,172.1,330.8	0.07Å	-	-	-
A 163		VAL	0.78	-	Favored (78.51%) Ile or Val / -69.3,-39.1	Favored (17.1%) *m* chi angles: 303.6	0.25Å	-	-	-
A 164		VAL	0.8	-	Favored (88.65%) Ile or Val / -58.6,-47.4	Favored (95.7%) *t* chi angles: 174.9	0.01Å	-	-	-
A 165		ALA	0.8	-	Favored (87.69%) General / -62.6,-46.6	-	0.05Å	-	-	-
A 166		ASN	0.78	-	Favored (94.53%) General / -61.2,-45.4	Favored (65.3%) *m-40* chi angles: 281.9,321.5	0.11Å	-	-	-

A 167		LEU	0.78	0.49Å HD12 with A 330 THR HG23	Favored (75.92%) General / -68.2,-44.3	Favored (74.1%) *mt* chi angles: 291.3,177.6	0.06Å	-	-	-
A 168		LYS	0.77	0.44Å HD2 with A 290 TYR OH	Favored (92.41%) General / -62.3,-45.6	Favored (81.8%) *mttt* chi angles: 292.4,188.8,179.5,168.7	0.11Å	-	-	-
A 169		ARG	0.71	-	Favored (26.23%) General / -86.5,-22.8	Favored (20.4%) *ptt180* chi angles: 76,194.2,170.5,169.3	0.14Å	-	-	-
A 170		LYS	0.76	-	Favored (12.52%) General / -108.4,-22.0	Favored (99%) *mttt* chi angles: 295.6,176.2,175.4,177.7	0.09Å	-	-	-
A 171		LEU	0.77	-	Favored (11.54%) General / -118.8,6.7	Favored (77.1%) *mt* chi angles: 292.5,178.6	0.14Å	-	-	-
A 172		GLY	0.8	-	Favored (71.48%) Glycine / 59.0,32.7	-	-	-	-	-
A 173		VAL	0.79	0.58Å HG13 with A 333 LYS HG3	Favored (2.95%) Ile or Val / -92.4,157.6	Favored (3.4%) *t* chi angles: 159.9	0.16Å	-	-	-
A 174		ASP	0.73	-	Allowed (1.46%) General / -133.3,70.7	Favored (12.4%) *m-30* chi angles: 283.8,290.5	0.04Å	-	-	-
**#**	**Alt**	**Res**	**High B**	**Clash > 0.4Å**	**Ramachandran**	**Rotamer**	**Cβ deviation**	**Bond lengths**	**Bond angles**	**Cis Peptides**
			Avg: 0.70	Clashscore: 10.3	Outliers: 12 of 650	Poor rotamers: 11 of 577	Outliers: 10 of 629	Outliers: 1 of 652	Outliers: 75 of 652	Non-Trans: 1 of 651
A 175		ILE	0.73	-	Favored (22.77%) Ile or Val / -78.4,137.0	Favored (36.9%) *pt* chi angles: 57.4,170.1	0.05Å	-	-	-
A 176		PHE	0.73	0.42Å HB3 with A 314 LEU HD11	Allowed (0.5%) General / 74.0,-61.3	Favored (51.9%) *m-80* chi angles: 305.9,112.4	0.16Å	-	OUTLIER(S) worst is C-N-CA: 4.3 σ	-
A 177		ILE	0.7	0.61Å HG23 with A 314 LEU HD22	Favored (15.24%) Ile or Val / -144.3,160.2	Favored (7.3%) *pt* chi angles: 48,173.3	0.10Å	-	-	-
A 178		GLY	0.71	-	Favored (9.89%) Glycine / -112.6,131.1	-	-	-	-	-
					Favored					

A 179	LEU	0.75	-	(38.71%) General / -123.9,122.7	Favored (2.1%) *mp* chi angles: 289.3,95.4	0.13Å	-	-	-
A 180	GLU	0.75	-	Favored (11.76%) General / -154.5,176.9	Favored (19.6%) *pt0* chi angles: 65.6,173.1,354.9	0.07Å	-	-	-
A 181	VAL	0.74	-	Favored (23.02%) Ile or Val / -123.6,109.4	Favored (47.7%) *t* chi angles: 181.9	0.07Å	-	-	-
A 182	ILE	0.69	0.55Å HA with A 478 LYS HZ2	Favored (7.64%) Ile or Val / -141.1,174.3	Favored (45.7%) *pt* chi angles: 63.5,171.7	0.23Å	-	-	-
A 183	LEU	0.69	0.57Å HD22 with A 485 GLN HG3	Favored (11.23%) General / -47.5,135.3	Favored (3.5%) *mp* chi angles: 290.7,68.6	0.03Å	-	-	-
A 184	ASP	0.71	-	Favored (16.3%) General / -67.8,121.6	Favored (37.4%) *t0* chi angles: 194.1,155.4	0.04Å	-	-	-
A 185	TYR	0.65	0.55Å CD1 with A 265 LEU HD21	Favored (68.44%) General / -64.1,-26.2	Favored (40.4%) *m-80* chi angles: 301.8,122.9	0.04Å	-	-	-
A 186	LYS	0.67	-	Favored (47.42%) General / -98.4,2.1	Favored (78.2%) *mttt* chi angles: 287.6,186.3,180.1,165	0.03Å	-	-	-
A 187	GLU	0.68	-	Allowed (0.75%) General / -157.0,73.5	Favored (11.9%) *tp30* chi angles: 180.3,58.7,75.8	0.12Å	-	-	-
A 188	GLU	0.69	0.47Å HG3 with A 485 GLN O	Favored (69.08%) General / -56.0,-37.4	Favored (21.7%) *tm-30* chi angles: 183.3,276.7,332.9	0.01Å	-	-	-
A 189	LYS	0.69	-	Favored (2.22%) General / -69.0,3.1	Favored (98.5%) *mttt* chi angles: 294.2,176.6,184.1,182.2	0.05Å	-	-	-
A 190	MET	0.69	-	Favored (49.38%) General / -125.0,146.5	Favored (94%) *mmm* chi angles: 297.9,294.3,291.1	0.12Å	-	-	-
A 191	HIS	0.7	-	Favored (22.05%) General / -81.4,164.4	Favored (54.7%) *m90* chi angles: 295.1,98.9	0.07Å	-	OUTLIER(S) worst is ND1-CE1-NE2: 4.3 σ	-
A 192	ARG	0.72	-	Favored (44.26%) General / -142.8,157.8	Favored (88.3%) *mmt-90* chi angles: 289,286.3,184.7,271.8	0.16Å	-	-	-

A 193		LEU	0.77	0.48Å HD13 with A 295 PHE CZ	Favored (56.66%) General / -63.5,135.1	Favored (8.1%) *tp* chi angles: 172.7,48.6	0.05Å	-	-	-
A 194		LYS	0.77	-	Favored (51.05%) General / -126.3,134.8	Favored (85.6%) *tttt* chi angles: 176.8,174.8,176.4,182.5	0.11Å	-	-	-
**#**	**Alt**	**Res**	**High B**	**Clash > 0.4Å**	**Ramachandran**	**Rotamer**	**Cβ deviation**	**Bond lengths**	**Bond angles**	**Cis Peptides**
			Avg: 0.70	Clashscore: 10.3	Outliers: 12 of 650	Poor rotamers: 11 of 577	Outliers: 10 of 629	Outliers: 1 of 652	Outliers: 75 of 652	Non-Trans: 1 of 651
A 195		ILE	0.76	-	Favored (73.55%) Ile or Val / -118.4,124.7	Favored (81.1%) *mt* chi angles: 295.2,174.6	0.05Å	-	-	-
A 196		GLY	0.72	-	Favored (12.59%) Glycine / -136.3,-172.4	-	-	-	-	-
A 197		ALA	0.7	-	Favored (55.88%) Pre-Pro / -101.4,108.6	-	0.09Å	-	-	-
A 198		PRO	0.61	0.42Å HD2 with A 177 ILE HA	OUTLIER (0)%) Trans-Pro / -124.1,-153.9	OUTLIER (0%) chi angles: 45.5,318.5,22.5	0.24Å	-	OUTLIER(S) worst is C-N-CA: 5.6 σ	-
A 199		GLN	0.59	0.55Å HE22 with A 333 LYS HB2	OUTLIER (0)%) General / 113.4,4.3	Allowed (1.1%) *tp40* chi angles: 157.6,74.2,104.9	0.29Å	-	OUTLIER(S) worst is CB-CG-CD: 7.7 σ	-
A 200		PHE	0.67	-	Favored (30.83%) Pre-Pro / -91.7,133.5	OUTLIER (0.1%) chi angles: 299.6,25.9	0.05Å	-	OUTLIER(S) worst is CA-C-N: 4.4 σ	-
A 201		PRO	0.75	-	Favored (5.81%) Trans-Pro / -89.6,-2.4	Favored (51.8%) *Cg_exo* chi angles: 336.7,38.5,321.2	0.03Å	-	-	-
A 202		MET	0.7	-	Favored (17.1%) General / -96.6,154.8	Favored (95.6%) *mmm* chi angles: 295.7,301.1,286.7	0.07Å	-	-	-
A 203		SER	0.69	-	Favored (65.72%) General / -66.5,-19.2	Favored (26%) *p* chi angles: 53.9	0.08Å	-	-	-
A 204		ARG	0.65	-	Favored (23.25%) General / -155.8,147.7	Favored (62%) *ttp-170* chi angles: 185.2,173.1,65.7,180.9	0.11Å	-	-	-

#	Alt	Res	High B	Clash > 0.4Å	Ramachandran	Rotamer	Cβ deviation	Bond lengths	Bond angles	Cis Peptides
A 205		ARG	0.65	-	Favored (16.29%) General / -50.8,-31.2	OUTLIER (0.2%) chi angles: 146.6,185.1,227.8,267	0.11Å	-	-	-
A 206		HIS	0.66	-	Favored (32.56%) General / -85.9,5.5	Favored (96%) *m-70* chi angles: 294.3,289.2	0.08Å	-	OUTLIER(S) worst is ND1-CG-CD2: 4.5 σ	-
A 207		TYR	0.65	-	Favored (32.23%) General / -82.2,-28.3	Favored (17.8%) *m-80* chi angles: 273.8,89.6	0.05Å	-	-	-
A 208		LEU	0.68	-	Favored (29.76%) General / -88.3,-17.8	Favored (67.7%) *mt* chi angles: 295.9,167.3	0.13Å	-	-	-
A 209		HIS	0.59	-	OUTLIER (0.03)%) Pre-Pro / -106.4,-70.2	Favored (87.1%) *t70* chi angles: 180.9,76.3	0.26Å	-	OUTLIER(S) worst is CA-C-N: 5.8 σ	-
A 210		PRO	0.53	-	Favored (11.22%) Trans-Pro / -48.0,-50.7	Favored (37%) *Cg_exo* chi angles: 326.9,32.7,339.7	0.05Å	-	-	-
A 211		HIS	0.39	-	Favored (3.58%) General / -127.7,41.1	Favored (98.8%) *m-70* chi angles: 302.6,288.2	0.12Å	-	OUTLIER(S) worst is ND1-CG-CD2: 4.1 σ	-
A 212		PHE	0.44	-	Allowed (0.1%) General / -63.6,7.7	Favored (11.9%) *p90* chi angles: 82.7,97.6	0.19Å	-	-	-
A 213		GLU	0.53	-	Favored (15.21%) General / -110.6,-4.9	Favored (97.8%) *mt-10* chi angles: 294.7,177.8,350.6	0.05Å	-	-	-
A 214		GLY	0.65	-	Allowed (0.42%) Glycine / -114.1,-54.3	-	-	-	-	-
**#**	**Alt**	**Res**	**High B**	**Clash > 0.4Å**	**Ramachandran**	**Rotamer**	**Cβ deviation**	**Bond lengths**	**Bond angles**	**Cis Peptides**
			Avg: 0.70	Clashscore: 10.3	Outliers: 12 of 650	Poor rotamers: 11 of 577	Outliers: 10 of 629	Outliers: 1 of 652	Outliers: 75 of 652	Non-Trans: 1 of 651
A 215		THR	0.71	-	Favored (18.85%) General / -102.2,-11.3	Favored (28.1%) *p* chi angles: 52.5	0.07Å	-	-	-
A 216		ARG	0.69	-	Favored (95.17%) General / -64.8,-42.2	Favored (4.9%) *tmm160* chi angles: 185.2,262.6,298.4,170.6	0.04Å	-	-	-

A 217		GLU	0.72	-	Favored (86.85%) General / -58.8,-46.7	Favored (60%) *tt0* chi angles: 181.7,190.2,343.4	0.06Å	-	-	-
A 218		ILE	0.73	-	Favored (93.86%) Ile or Val / -59.7,-44.6	Favored (83.3%) *mt* chi angles: 290.8,171.1	0.09Å	-	-	-
A 219		TYR	0.75	0.53Å CE1 with A 223 ILE HD11	Favored (61.27%) General / -64.8,-51.6	Favored (16%) *t80* chi angles: 195.3,238.9	0.18Å	-	-	-
A 220		GLU	0.74	-	Favored (68.81%) General / -53.1,-46.6	Favored (72%) *mt-10* chi angles: 292.4,175.9,317.3	0.07Å	-	-	-
A 221		ARG	0.69	-	Favored (82.98%) General / -57.3,-43.4	Favored (87.5%) *mtt-85* chi angles: 283.5,173.3,182.1,282.7	0.18Å	-	-	-
A 222		SER	0.74	-	Favored (98.82%) General / -63.4,-41.3	Favored (16.3%) *m* chi angles: 286	0.15Å	-	-	-
A 223		ILE	0.7	0.53Å HD11 with A 219 TYR CE1	Allowed (1.68%) Ile or Val / -75.0,-61.5	Favored (74.3%) *mt* chi angles: 300.8,167.3	0.04Å	-	-	-
A 224		GLU	0.65	0.54Å HG2 with A 243 ALA HB3	Favored (90.26%) General / -66.2,-40.3	Favored (32.6%) *mt-10* chi angles: 287.6,158,27.3	0.12Å	-	-	-
A 225		ASN	0.62	-	Favored (2.19%) General / -111.5,-57.9	Favored (48.7%) *t0* chi angles: 185.5,46.4	0.14Å	-	OUTLIER(S) worst is CA-CB-CG: 5.3 σ	-
A 226		LYS	0.57	-	Favored (40.02%) General / -66.3,-10.1	Favored (21.3%) *mtpt* chi angles: 296.8,170.2,49.1,191.6	0.04Å	-	-	-
A 227		LEU	0.65	-	Allowed (0.89%) General / -134.5,-35.7	Favored (88.5%) *mt* chi angles: 294,169.9	0.12Å	-	-	-
A 228		LYS	0.64	-	Favored (60.69%) General / -74.8,-16.8	Favored (2.2%) *mptt* chi angles: 253.5,84.4,167.9,181	0.09Å	-	-	-
A 229		LEU	0.61	-	Favored (11.59%) General / -78.0,-50.7	Favored (25.7%) *tp* chi angles: 171.6,55.7	0.09Å	-	-	-
A		TYR	0.57	-	Allowed (1.18%)	Favored (17.2%) *m-80*	0.05Å	-	-	-

230					General / -54.2,-63.4	chi angles: 273.6,90.1				
A 231		PHE	0.58	0.51Å CE1 with A 136 GLN HA	Favored (39.69%) Pre-Pro / -68.2,-46.3	OUTLIER (0%) chi angles: 169.1,134.6	0.30Å	-	OUTLIER(S) worst is C-CA-CB: 5.1 σ	-
A 232		PRO	0.55	0.48Å CG with A 147 GLU HB3	Favored (2.94%) Trans-Pro / -83.6,-24.4	Favored (4.4%) *Cg_exo* chi angles: 323.1,38.4,334.7	0.17Å	-	-	-
A 233		GLU	0.46	-	Favored (18.45%) General / 55.8,48.3	Favored (85.4%) *mt-10* chi angles: 301.8,179.2,358.2	0.15Å	-	-	-
A 234		GLN	0.42	-	Favored (30.15%) General / -113.7,152.7	Favored (2.8%) *mm-40* chi angles: 292,293.1,251.1	0.08Å	-	-	-
**#**	**Alt**	**Res**	**High B**	**Clash > 0.4Å**	**Ramachandran**	**Rotamer**	**Cβ deviation**	**Bond lengths**	**Bond angles**	**Cis Peptides**
			Avg: 0.70	Clashscore: 10.3	Outliers: 12 of 650	Poor rotamers: 11 of 577	Outliers: 10 of 629	Outliers: 1 of 652	Outliers: 75 of 652	Non-Trans: 1 of 651
A 235		SER	0.57	-	Favored (34.24%) General / -61.8,151.4	Favored (9.4%) *m* chi angles: 308.7	0.10Å	-	-	-
A 236		GLU	0.6	-	Favored (75.41%) General / -57.2,-39.5	OUTLIER (0.3%) chi angles: 200.4,131.7,295.4	0.18Å	-	-	-
A 237		HIS	0.56	-	Favored (55.87%) General / -52.5,-36.9	Favored (68.4%) *t70* chi angles: 189.1,71.1	0.08Å	-	OUTLIER(S) worst is ND1-CG-CD2: 4.6 σ	-
A 238		TRP	0.54	-	Favored (49.86%) General / -77.3,-38.8	Favored (96.2%) *m100* chi angles: 294.6,101.4	0.04Å	-	-	-
A 239		ARG	0.62	-	Favored (58.28%) General / -75.1,-41.6	Favored (48.4%) *mtm110* chi angles: 295.4,173.8,287.5,105.3	0.07Å	-	-	-
A 240		GLN	0.65	-	Favored (72%) General / -58.0,-50.8	Favored (62.2%) *tt0* chi angles: 179.6,181.2,10.7	0.06Å	-	-	-
A 241		GLU	0.7	-	Favored (93.42%) General / -60.7,-45.6	Favored (84.7%) *tt0* chi angles: 181.1,184.9,355.8	0.03Å	-	-	-
A 242		VAL	0.74	-	Favored (91.86%) Ile or Val / -61.2,-47.8	Favored (46.1%) *t* chi angles: 168.9	0.11Å	-	-	-

#	Alt	Res	High B	Clash > 0.4Å	Ramachandran	Rotamer	Cβ deviation	Bond lengths	Bond angles	Cis Peptides
A 243		ALA	0.78	0.54Å HB3 with A 224 GLU HG2	Favored (53.58%) General / -77.6,-33.6	-	0.12Å	-	-	-
A 244		SER	0.81	-	Favored (70.52%) General / -55.6,-39.2	Favored (11.8%) *m* chi angles: 284.5	0.13Å	-	-	-
A 245		GLN	0.78	-	Favored (91.04%) General / -62.4,-38.8	Favored (74.7%) *mt0* chi angles: 294.1,176.4,59.6	0.08Å	-	-	-
A 246		VAL	0.81	-	Favored (87.5%) Ile or Val / -58.2,-47.4	Favored (85.1%) *t* chi angles: 177.3	0.07Å	-	-	-
A 247		VAL	0.81	-	Favored (87.21%) Ile or Val / -57.8,-45.6	Favored (99.6%) *t* chi angles: 175.4	0.08Å	-	-	-
A 248		GLN	0.78	-	Favored (92.87%) General / -59.5,-42.8	Favored (98.9%) *mt0* chi angles: 291.1,174.4,333.5	0.06Å	-	-	-
A 249		ILE	0.79	-	Favored (98.73%) Ile or Val / -61.8,-44.4	Favored (70.8%) *mt* chi angles: 288.5,169.1	0.05Å	-	-	-
A 250		GLU	0.75	-	Favored (96.79%) General / -60.4,-43.0	OUTLIER (0.3%) chi angles: 233.5,147.6,240.7	0.07Å	-	-	-
A 251		GLN	0.73	-	Favored (86.94%) General / -62.8,-37.5	Favored (59.8%) *mt0* chi angles: 296.7,180.6,281.4	0.04Å	-	-	-
A 252		GLN	0.75	-	Favored (91.46%) General / -62.1,-39.1	Favored (85.9%) *tp40* chi angles: 180,60.6,46.2	0.10Å	-	-	-
A 253		LEU	0.76	-	Favored (78.7%) General / -58.0,-39.7	OUTLIER (0.1%) chi angles: 230.2,76.1	0.19Å	-	-	-
A 254		ALA	0.77	-	Favored (4.51%) General / -82.4,12.4	-	0.03Å	-	-	-
**#**	**Alt**	**Res**	**High B**	**Clash > 0.4Å**	**Ramachandran**	**Rotamer**	**Cβ deviation**	**Bond lengths**	**Bond angles**	**Cis Peptides**
			Avg: 0.70	Clashscore: 10.3	Outliers: 12 of 650	Poor rotamers: 11 of 577	Outliers: 10 of 629	Outliers: 1 of 652	Outliers: 75 of 652	Non-Trans: 1 of 651

A 255		LYS	0.72	-	Favored (24.38%) General / -88.9,9.6	Favored (84.7%) *mttt* chi angles: 297.8,181,188.2,170.6	0.04Å	-	-	-
A 256		GLY	0.68	-	OUTLIER (0.01)%) Glycine / -63.0,-99.5	-	-	-	-	-
A 257		LEU	0.65	-	Favored (15.27%) Pre-Pro / -145.8,140.0	Allowed (1.4%) *pt* chi angles: 65.6,167.2	0.10Å	-	-	-
A 258		PRO	0.65	-	Favored (25.68%) Trans-Pro / -78.3,165.4	Favored (19.1%) *Cg_exo* chi angles: 342.7,33.6,323	0.07Å	-	-	-
A 259		HIS	0.64	-	Favored (45.29%) General / -80.8,-19.2	Favored (59.3%) *p-80* chi angles: 62.4,286.4	0.10Å	-	OUTLIER(S) worst is ND1-CG-CD2: 4.4 σ	-
A 260		ASN	0.66	-	Favored (86.87%) Pre-Pro / -62.8,-44.7	Favored (32.9%) *m-40* chi angles: 296.4,359.3	0.18Å	-	-	-
A 261		PRO	0.66	-	Allowed (0.23%) Trans-Pro / -71.6,26.7	Favored (55.9%) *Cg_exo* chi angles: 336.2,35.7,325.7	0.10Å	-	-	-
A 262		ALA	0.66	-	Favored (2.08%) General / -141.8,13.2	-	0.02Å	-	-	-
A 263		LEU	0.65	-	Favored (49.74%) General / -74.1,-45.4	Favored (93.1%) *mt* chi angles: 297.5,173.2	0.13Å	-	-	-
A 264		THR	0.68	-	Favored (9.48%) General / -93.5,95.8	Favored (25.2%) *m* chi angles: 291.7	0.06Å	-	-	-
A 265		LEU	0.68	0.55Å HD21 with A 185 TYR CD1	Favored (68.76%) General / -59.7,-30.3	Favored (13.3%) *tp* chi angles: 195.2,66.5	0.08Å	-	-	-
A 266		ALA	0.7	-	Favored (66.76%) General / -57.2,-32.7	-	0.14Å	-	-	-
A 267		GLN	0.66	-	Favored (19.78%) General / -95.4,-19.6	Favored (99.4%) *mm-40* chi angles: 298.2,300,315.9	0.07Å	-	-	-
A 268		THR	0.66	-	Favored (18.27%) General /	Favored (31.2%) *p* chi angles: 53.4	0.08Å	-	-	-

					-98.1,-18.9					
A 269		THR	0.7	0.46Å HA with A 304 TYR CE1	Favored (2.47%) General / -66.5,108.6	Favored (60.6%) *m* chi angles: 295.6	0.25Å	-	-	-
A 270		ARG	0.7	-	Favored (12.39%) General / -125.6,109.2	Favored (92.3%) *mmt-90* chi angles: 298,294.8,183.1,272.4	0.05Å	-	-	-
A 271		GLN	0.73	-	Favored (29.96%) General / -87.2,140.0	Favored (63.4%) *tt0* chi angles: 183.7,177.4,31.8	0.04Å	-	-	-
A 272		ARG	0.75	-	Favored (44.25%) General / -143.1,157.7	Favored (87.5%) *mmt-90* chi angles: 291.2,291.9,179.7,269.7	0.16Å	-	-	-
A 273		THR	0.78	-	Favored (31%) General / -77.2,157.2	Favored (32.3%) *p* chi angles: 69	0.17Å	-	-	-
A 274		ALA	0.78	-	Favored (55.81%) General / -54.3,-32.2	-	0.09Å	-	-	-
**#**	**Alt**	**Res**	**High B**	**Clash > 0.4Å**	**Ramachandran**	**Rotamer**	**Cβ deviation**	**Bond lengths**	**Bond angles**	**Cis Peptides**
			Avg: 0.70	Clashscore: 10.3	Outliers: 12 of 650	Poor rotamers: 11 of 577	Outliers: 10 of 629	Outliers: 1 of 652	Outliers: 75 of 652	Non-Trans: 1 of 651
A 275		ALA	0.77	-	Favored (85.01%) General / -65.1,-45.5	-	0.02Å	-	-	-
A 276		GLU	0.73	-	Favored (97.99%) General / -63.7,-42.7	Favored (77.4%) *mt-10* chi angles: 287.8,171.2,147.3	0.04Å	-	-	-
A 277		MET	0.75	0.60Å HE3 with A 281 TYR CE2	Favored (83.24%) General / -58.0,-41.6	Favored (94.3%) *mmm* chi angles: 300.5,305.1,291.1	0.05Å	-	-	-
A 278		LYS	0.75	-	Favored (66.35%) General / -65.8,-20.8	Favored (76.4%) *tttt* chi angles: 194.2,178.2,178.2,177.8	0.07Å	-	-	-
A 279		THR	0.73	-	Favored (14.32%) General / -84.9,-43.1	Favored (64.7%) *p* chi angles: 63.2	0.15Å	-	-	-
A 280		ALA	0.75	-	Favored (65.39%) General / -54.5,-37.5	-	0.08Å	-	-	-
				0.60Å	Favored (2.5%)					

A 281		TYR	0.73	CE2 with A 277 MET HE3	General / -139.8,17.1	OUTLIER (0.1%) chi angles: 70.1,42.6	0.21Å	-	-	-
A 282		GLY	0.73	-	Favored (55.38%) Glycine / -62.3,-17.1	-	-	-	-	-
A 283		SER	0.67	-	Favored (36.39%) General / -79.7,-1.0	Favored (29.7%) *m* chi angles: 303.1	0.02Å	-	-	-
A 284		TYR	0.68	0.63Å CD1 with A 285 VAL HG23	Favored (2.86%) General / -112.7,-51.0	Favored (6.3%) *p90* chi angles: 85.2,102.5	0.21Å	-	OUTLIER(S) worst is CA-CB-CG: 6.6 σ	-
A 285		VAL	0.77	0.63Å HG23 with A 284 TYR CD1	Favored (67.46%) Ile or Val / -129.2,129.3	Allowed (1.4%) *t* chi angles: 157.6	0.05Å	-	-	-
A 286		ASP	0.79	-	Favored (3.78%) General / -77.3,91.2	Favored (41.1%) *t0* chi angles: 183.2,18.7	0.11Å	-	-	-
A 287		VAL	0.79	-	Favored (97.06%) Ile or Val / -62.6,-42.7	Favored (8.2%) *p* chi angles: 61.2	0.15Å	-	-	-
A 288		THR	0.77	0.45Å HG23 with A 299 LEU HD22	Favored (92.62%) General / -61.7,-45.8	Favored (23.7%) *m* chi angles: 291.4	0.07Å	-	-	-
A 289		ARG	0.71	-	Favored (99.67%) General / -62.7,-42.5	Favored (82.5%) *ttt180* chi angles: 181.3,177.7,177.7,180.6	0.15Å	-	-	-
A 290		TYR	0.76	0.44Å OH with A 168 LYS HD2	Favored (73.33%) General / -61.8,-50.2	Favored (42%) *t80* chi angles: 190.4,69.7	0.15Å	-	-	-
A 291		LEU	0.75	-	Favored (81.67%) General / -67.6,-42.9	Favored (88.4%) *mt* chi angles: 297.9,179.3	0.07Å	-	-	-
A 292		GLN	0.71	-	Favored (68.82%) General / -59.5,-30.8	Favored (94.6%) *mt0* chi angles: 286.6,173.2,339.1	0.04Å	-	-	-
A 293		LEU	0.73	-	Favored (24.44%) General / -93.5,-16.5	Favored (13.4%) *mt* chi angles: 284.5,184	0.10Å	-	-	-
A 294		ILE	0.72	0.41Å O with A 463 LEU HD13	Favored (13.99%) Ile or Val / -91.2,-48.3	Favored (46.6%) *mt* chi angles: 291.4,179.1	0.08Å	-	-	-

#	Alt	Res	High B	Clash > 0.4Å	Ramachandran	Rotamer	Cβ deviation	Bond lengths	Bond angles	Cis Peptides
			Avg: 0.70	Clashscore: 10.3	Outliers: 12 of 650	Poor rotamers: 11 of 577	Outliers: 10 of 629	Outliers: 1 of 652	Outliers: 75 of 652	Non-Trans: 1 of 651
A 295		PHE	0.71	0.48Å CZ with A 193 LEU HD13	Favored (17.91%) General / -76.7,169.7	Favored (68.6%) *m-80* chi angles: 287,83.7	0.09Å	-	OUTLIER(S) worst is CA-CB-CG: 6.7 σ	-
A 296		ASN	0.55	-	Favored (16.3%) General / -91.9,158.4	Favored (42.3%) *m-40* chi angles: 286.3,6.1	0.09Å	-	-	-
A 297		ASP	0.52	-	Allowed (0.3%) General / 61.2,-107.8	Favored (32%) *m-30* chi angles: 303.9,340.5	0.15Å	-	-	-
A 298		ASN	0.69	-	Favored (2.14%) General / -110.5,-173.0	Favored (28.7%) *p0* chi angles: 57.5,1.4	0.07Å	-	-	-
A 299		LEU	0.7	0.45Å HD22 with A 288 THR HG23	Favored (58.34%) General / -63.1,137.8	Favored (55.7%) *tp* chi angles: 180.4,64.6	0.10Å	-	-	-
A 300		TYR	0.54	-	Favored (29.46%) General / -95.7,116.8	Favored (8.5%) *t80* chi angles: 203.6,72.9	0.11Å	-	-	-
A 301		MET	0.77	-	Favored (70.96%) General / -63.9,-49.9	Favored (57.4%) *ttp* chi angles: 175.1,182.7,74.9	0.05Å	-	-	-
A 302		ASP	0.78	-	Favored (27.06%) General / -85.1,120.8	Favored (80.9%) *m-30* chi angles: 283.1,342.5	0.15Å	-	-	-
A 303		LEU	0.78	-	Favored (55.5%) General / -118.7,129.8	Favored (2.9%) *mp* chi angles: 289.9,90.9	0.08Å	-	-	-
A 304		TYR	0.76	0.46Å CE1 with A 269 THR HA	Favored (31.64%) General / -87.1,123.0	Favored (2.9%) *t80* chi angles: 167.4,36.1	0.10Å	-	-	-
A 305		GLU	0.76	-	Favored (52.6%) General / -115.3,125.0	Favored (47.3%) *tt0* chi angles: 182.2,182.1,59.3	0.13Å	-	-	-
A 306		THR	0.71	0.53Å HB with A 307 PRO HD3	Allowed (1.41%) Pre-Pro / -79.4,-49.5	Favored (16.1%) *m* chi angles: 309.8	0.14Å	-	OUTLIER(S) worst is CA-CB-OG1: 4.2 σ	-
					OUTLIER	Favored (56.4%)				

A 307		PRO	0.72	0.53Å HD3 with A 306 THR HB	(0)%) Trans-Pro / -121.4,117.8	*Cg_endo* chi angles: 32.3,327.8,20	0.03Å	-	-	-
A 308		GLU	0.69	-	Favored (70.53%) General / -59.9,-51.2	Favored (52.5%) *tt0* chi angles: 183.5,161.5,2.6	0.05Å	-	-	-
A 309		ASP	0.73	-	Favored (72.11%) General / -61.8,-31.9	Favored (92.2%) *m-30* chi angles: 293,340.4	0.08Å	-	-	-
A 310		TYR	0.71	0.53Å HB3 with A 307 PRO HG2	Favored (99%) General / -63.3,-42.0	Favored (54.5%) *t80* chi angles: 172.3,88.3	0.07Å	-	-	-
A 311		MET	0.74	0.55Å SD with A 177 ILE HG21	Favored (95.32%) General / -64.7,-42.4	Favored (65.6%) *mmm* chi angles: 287.9,308,307.4	0.07Å	-	-	-
A 312		SER	0.77	0.41Å HA with A 315 VAL HG12	Favored (84.87%) General / -59.4,-40.0	Favored (25.3%) *m* chi angles: 303.8	0.10Å	-	-	-
A 313		ASN	0.76	-	Favored (73.21%) General / -70.5,-40.8	Favored (89.1%) *m-40* chi angles: 289.9,326.1	0.04Å	-	OUTLIER(S) worst is CA-CB-CG: 5.0 σ	-
A 314		LEU	0.75	0.61Å HD22 with A 177 ILE HG23	Favored (88.71%) General / -58.4,-43.2	Favored (62.9%) *tp* chi angles: 175.2,61	0.11Å	-	-	-
**#**	**Alt**	**Res**	**High B**	**Clash > 0.4Å**	**Ramachandran**	**Rotamer**	**Cβ deviation**	**Bond lengths**	**Bond angles**	**Cis Peptides**
			Avg: 0.70	Clashscore: 10.3	Outliers: 12 of 650	Poor rotamers: 11 of 577	Outliers: 10 of 629	Outliers: 1 of 652	Outliers: 75 of 652	Non-Trans: 1 of 651
A 315		VAL	0.76	0.41Å HG12 with A 312 SER HA	Favored (98.87%) Ile or Val / -61.2,-45.3	Favored (5.7%) *p* chi angles: 57.9	0.17Å	-	-	-
A 316		ASP	0.74	-	Favored (93.9%) General / -64.4,-43.6	Favored (91.2%) *m-30* chi angles: 292.1,337.6	0.14Å	-	-	-
A 317		VAL	0.77	-	Favored (77.09%) Ile or Val / -55.0,-46.8	Favored (35.4%) *t* chi angles: 166.3	0.15Å	-	-	-
A 318		ILE	0.75	-	Favored (82.29%) Ile or Val / -67.7,-39.6	Favored (82.6%) *mt* chi angles: 291.8,163.7	0.11Å	-	-	-
					Favored	Favored (36.7%)				

A 319		ARG	0.7	-	(59.64%) General / -75.2,-19.7	*ptt90* chi angles: 68.9,185.4,178.4,70.2	0.05Å	-	-	-
A 320		GLU	0.73	-	Favored (19.09%) General / -94.5,-21.1	Favored (96.6%) *mt-10* chi angles: 289.9,177.8,1.9	0.03Å	-	-	-
A 321		THR	0.77	-	Favored (44.1%) Pre-Pro / -91.3,152.7	Allowed (0.7%) *p* chi angles: 86.4	0.26Å	-	-	-
A 322		PRO	0.77	-	Favored (68.5%) Trans-Pro / -68.9,147.5	Favored (48.6%) *Cg_endo* chi angles: 25,326.1,30.1	0.05Å	-	-	-
A 323		LYS	0.7	-	Favored (67.78%) General / -58.8,-30.7	Favored (72.6%) *mmtt* chi angles: 295,296.7,182.2,185.5	0.08Å	-	-	-
A 324		LEU	0.69	0.79Å HD11 with A 146 PRO CG	Favored (71.27%) General / -63.8,-49.8	Favored (75%) *tp* chi angles: 177.8,62.5	0.09Å	-	-	-
A 325		GLN	0.75	-	Favored (79.28%) General / -60.0,-37.3	Favored (68.3%) *mt0* chi angles: 284.9,173,22.8	0.09Å	-	-	-
A 326		LEU	0.76	-	Favored (79.69%) General / -57.5,-48.3	Favored (86.3%) *mt* chi angles: 296.7,171.3	0.03Å	-	-	-
A 327		ALA	0.77	-	Favored (86.37%) General / -60.5,-39.0	-	0.02Å	-	-	-
A 328		ASN	0.75	0.72Å HD21 with A 146 PRO HG2	Favored (74.83%) General / -69.6,-42.1	Favored (50.9%) *m-40* chi angles: 287.3,4	0.08Å	-	-	-
A 329		TYR	0.78	-	Favored (87.1%) General / -62.4,-46.8	Favored (34.3%) *t80* chi angles: 175.4,237.4	0.31Å	-	OUTLIER(S) worst is N-CA-CB: 6.6 σ	-
A 330		THR	0.77	0.49Å HG23 with A 167 LEU HD12	Favored (79.72%) General / -57.3,-48.1	Allowed (1.8%) *m* chi angles: 283.4	0.13Å	-	-	-
A 331		MET	0.78	-	Favored (85.64%) General / -65.8,-37.2	Favored (70.4%) *mmm* chi angles: 292.6,314.5,301.2	0.09Å	-	-	-
A 332		TRP	0.79	0.43Å CZ2 with A 223 ILE HG12	Favored (89.14%) General / -58.4,-44.4	Favored (60.2%) *t-100* chi angles: 171.4,254.7	0.04Å	-	-	-

A 333		LYS	0.76	0.58Å HG3 with A 173 VAL HG13	Favored (84.36%) General / -62.5,-37.0	Favored (53.8%) *tptt* chi angles: 182.8,64.1,177.6,184.6	0.04Å	-	-	-
A 334		ALA	0.8	-	Favored (89.86%) General / -59.7,-46.2	-	0.02Å	-	-	-
**#**	**Alt**	**Res**	**High B**	**Clash > 0.4Å**	**Ramachandran**	**Rotamer**	**Cβ deviation**	**Bond lengths**	**Bond angles**	**Cis Peptides**
			Avg: 0.70	Clashscore: 10.3	Outliers: 12 of 650	Poor rotamers: 11 of 577	Outliers: 10 of 629	Outliers: 1 of 652	Outliers: 75 of 652	Non-Trans: 1 of 651
A 335		LEU	0.76	0.46Å HG with A 135 LEU HD11	Favored (89.56%) General / -60.0,-46.4	Favored (8.7%) *mp* chi angles: 273.9,60.8	0.11Å	-	-	-
A 336		GLU	0.72	-	Favored (69.85%) General / -56.1,-37.7	Favored (53.5%) *tp30* chi angles: 183.7,64.9,32.6	0.03Å	-	-	-
A 337		ALA	0.77	-	Favored (61.26%) General / -72.3,-22.1	-	0.06Å	-	-	-
A 338		LEU	0.74	-	Favored (7.4%) General / -120.4,-19.9	Favored (81.5%) *mt* chi angles: 293.6,167.4	0.12Å	-	-	-
A 339		ASP	0.7	-	Favored (67.86%) General / -59.6,-29.3	Favored (74.8%) *m-30* chi angles: 281.8,342.7	0.03Å	-	-	-
A 340		ILE	0.68	-	Favored (14.38%) Ile or Val / -54.6,-25.2	Favored (33.7%) *pt* chi angles: 62.6,165.1	0.07Å	-	-	-
A 341		ALA	0.73	-	Favored (25.63%) General / -100.7,16.2	-	0.05Å	-	-	-
A 342		ARG	0.65	-	Allowed (0.58%) General / -103.0,-156.8	Favored (69.7%) *mtm180* chi angles: 301.6,181.4,305.2,177.3	0.04Å	-	-	-
A 343		VAL	0.64	0.66Å HG11 with A 131 ALA HA	Favored (6.78%) Pre-Pro / -81.4,174.0	Favored (29.7%) *m* chi angles: 298.1	0.15Å	-	OUTLIER(S) worst is CA-C-N: 4.3 σ	-
A 344		PRO	0.62	0.50Å HD2 with A 343 VAL HB	Favored (7.92%) Trans-Pro / -77.5,63.7	Favored (50.8%) *Cg_endo* chi angles: 33,321.2,30.1	0.01Å	-	-	-
					Favored					

A 345		ALA	0.52	-	(55.94%) General / -78.7,-17.4	-	0.08Å	-	-	-
A 346		SER	0.52	-	Favored (41.84%) General / -142.9,152.2	Favored (85.4%) *p* chi angles: 67.7	0.23Å	-	-	-
A 347		GLN	0.43	-	Favored (31.94%) General / -79.5,148.1	Favored (89.4%) *mm-40* chi angles: 302.5,295.3,309	0.03Å	-	-	-
A 348		ARG	0.46	-	Favored (32.75%) General / -143.4,145.6	Favored (83.6%) *mmt-90* chi angles: 291.8,298.1,183.4,266.8	0.05Å	-	-	-
A 349		ALA	0.53	-	Favored (57.23%) General / -62.7,143.6	-	0.15Å	-	-	-
A 350		ASP	0.61	-	Favored (51.85%) General / -52.4,-35.5	Favored (90.5%) *m-30* chi angles: 287.7,338.9	0.03Å	-	-	-
A 351		ILE	0.7	-	Allowed (1.82%) Ile or Val / -61.1,-8.1	Favored (47.5%) *pt* chi angles: 62.1,171.5	0.05Å	-	OUTLIER(S) worst is C-N-CA: 4.8 σ	-
A 352		TRP	0.7	-	OUTLIER (0.03)%) General / -22.1,-59.1	Favored (33.5%) *t-100* chi angles: 168.6,240.8	0.12Å	-	-	-
A 353		CYS	0.79	-	Favored (41.49%) General / -101.9,7.4	Favored (9.8%) *p* chi angles: 76.6	0.24Å	-	-	-
A 354		VAL	0.79	-	Favored (87.51%) Ile or Val / -67.3,-42.4	Favored (3.8%) *t* chi angles: 160.2	0.10Å	-	-	-
**#**	**Alt**	**Res**	**High B**	**Clash > 0.4Å**	**Ramachandran**	**Rotamer**	**Cβ deviation**	**Bond lengths**	**Bond angles**	**Cis Peptides**
			Avg: 0.70	Clashscore: 10.3	Outliers: 12 of 650	Poor rotamers: 11 of 577	Outliers: 10 of 629	Outliers: 1 of 652	Outliers: 75 of 652	Non-Trans: 1 of 651
A 355		GLN	0.77	-	Favored (86.6%) General / -61.0,-47.4	Favored (27%) *tm-30* chi angles: 184.4,280.2,324.4	0.08Å	-	-	-
A 356		LEU	0.8	-	Favored (99.56%) General / -61.7,-42.9	Favored (95.4%) *mt* chi angles: 294.5,172.1	0.08Å	-	-	-
				0.50Å	Favored					

A 357	ALA	0.82	HB1 with A 114 LEU HD11	(95.37%) General / -60.1,-43.7	-	0.13Å	-	-	-
A 358	GLN	0.78	-	Favored (63.62%) General / -69.6,-24.3	Favored (14.2%) *mt0* chi angles: 293.9,175.2,162.3	0.08Å	-	-	-
A 359	GLN	0.78	-	Favored (51.5%) General / -74.7,-44.2	Favored (47.7%) *tt0* chi angles: 182.1,179.8,304.9	0.03Å	-	-	-
A 360	PHE	0.79	-	Favored (13.24%) General / -94.6,-32.8	Favored (49.6%) *m-80* chi angles: 283.4,101.6	0.04Å	-	OUTLIER(S) worst is CA-CB-CG: 4.9 σ	-
A 361	PHE	0.79	0.51Å CE2 with A 440 LEU HD21	Allowed (1.17%) Pre-Pro / -125.7,44.5	Favored (21.3%) *m-80* chi angles: 303,137.9	0.14Å	-	-	-
A 362	PRO	0.79	-	Favored (37.32%) Trans-Pro / -66.1,-34.4	Favored (18.6%) *Cg_endo* chi angles: 18.7,335,21.6	0.06Å	-	-	-
A 363	HIS	0.75	-	Favored (58.7%) General / -75.0,-22.2	Favored (10%) *m170* chi angles: 309.4,168.8	0.02Å	-	-	-
A 364	GLN	0.73	-	Favored (89.42%) General / -66.3,-39.6	Favored (77.1%) *mt0* chi angles: 295.1,176.8,16.7	0.10Å	-	-	-
A 365	LEU	0.78	0.65Å HG with A 114 LEU HD21	Favored (61.81%) General / -72.0,-20.0	Favored (17.7%) *mt* chi angles: 288.9,157.1	0.21Å	-	OUTLIER(S) worst is C-CA-CB: 4.5 σ	-
A 366	GLU	0.73	0.48Å CD with A 428 HIS HB2	Favored (89.76%) General / -58.6,-44.4	Favored (12.1%) *tm-30* chi angles: 189,271.8,348.5	0.10Å	-	-	-
A 367	SER	0.77	-	Favored (90.21%) General / -58.8,-43.2	Favored (57.8%) *m* chi angles: 293.2	0.04Å	-	-	-
A 368	LEU	0.77	-	Favored (87.88%) General / -63.6,-46.0	Favored (91.1%) *mt* chi angles: 291.9,169.6	0.13Å	-	-	-
A 369	PHE	0.76	-	Favored (76.43%) General / -63.2,-48.9	Favored (64.2%) *t80* chi angles: 187.4,260.5	0.04Å	-	-	-
A 370	HIS	0.75	0.43Å HB2 with A 493 ILE HD13	Favored (57.18%) General / -76.3,-29.3	Favored (3.4%) *p-80* chi angles: 93.4,282.1	0.18Å	-	OUTLIER(S) worst is ND1-CG-CD2: 4.4 σ	-

A 371		ARG	0.71	-	Favored (91.36%) General / -59.7,-41.7	Favored (58.6%) *ttp-170* chi angles: 180.5,186.6,67,197.1	0.05Å	-	-	-
A 372		ASN	0.75	-	Favored (57.81%) General / -72.0,-46.3	Favored (52.5%) *t0* chi angles: 186.8,60.8	0.11Å	-	-	-
A 373		TYR	0.69	-	Favored (15.01%) General / -109.2,-9.8	Favored (5.8%) *t80* chi angles: 205.8,70.2	0.07Å	-	-	-
A 374		ASN	0.71	-	Favored (9.75%) General / -123.5,105.4	Favored (50.8%) *t0* chi angles: 190.1,54.9	0.06Å	-	-	-
**#**	**Alt**	**Res**	**High B**	**Clash > 0.4Å**	**Ramachandran**	**Rotamer**	**Cβ deviation**	**Bond lengths**	**Bond angles**	**Cis Peptides**
			Avg: 0.70	Clashscore: 10.3	Outliers: 12 of 650	Poor rotamers: 11 of 577	Outliers: 10 of 629	Outliers: 1 of 652	Outliers: 75 of 652	Non-Trans: 1 of 651
A 375		HIS	0.69	-	Favored (57.96%) General / -61.8,136.4	Favored (96.5%) *m-70* chi angles: 295.6,283.7	0.14Å	-	OUTLIER(S) worst is ND1-CG-CD2: 4.4 σ	-
A 376		MET	0.66	-	Favored (64.13%) General / -68.2,-16.9	Favored (94.9%) *mtp* chi angles: 294.4,176.5,66.6	0.05Å	-	-	-
A 377		GLN	0.71	-	Favored (66.84%) General / -54.4,-39.0	Favored (45.2%) *tp40* chi angles: 180.6,62.6,18.9	0.12Å	-	-	-
A 378		MET	0.72	0.47Å O with A 382 LEU HD23	Favored (87.26%) General / -65.5,-44.4	Favored (24.5%) *mmp* chi angles: 301.7,288.2,110.6	0.02Å	-	OUTLIER(S) worst is CG-SD-CE: 4.7 σ	-
A 379		ILE	0.72	0.40Å HG23 with A 425 PHE CE1	Favored (30.24%) Ile or Val / -50.9,-46.7	Favored (67.2%) *mt* chi angles: 288.9,172.7	0.07Å	-	-	-
A 380		ASN	0.77	-	Favored (82.99%) General / -65.1,-45.9	Favored (87.4%) *m-40* chi angles: 295.6,333	0.04Å	-	-	-
A 381		GLU	0.77	-	Favored (88.27%) General / -65.2,-37.7	Favored (91.3%) *mt-10* chi angles: 293.5,182.7,9	0.01Å	-	-	-
A 382		LEU	0.78	0.64Å CD1 with A 515 LEU HD21	Favored (92.37%) General / -65.2,-39.2	Favored (2.5%) *mm* chi angles: 275.8,298.2	0.06Å	-	OUTLIER(S) worst is CB-CG-CD2: 4.1 σ	-
					Favored					

A 383		GLN	0.76	-	(96.61%) General / -64.4,-41.4	Favored (62.3%) *tt0* chi angles: 189.3,173.5,4.4	0.07Å	-	-	-
A 384		SER	0.81	-	Favored (98.15%) General / -63.3,-43.3	Favored (94.3%) *p* chi angles: 64.7	0.21Å	-	-	-
A 385		THR	0.79	0.46Å HG21 with A 502 TRP CZ3	Favored (95.95%) General / -60.8,-41.5	Favored (47.3%) *p* chi angles: 55.7	0.15Å	-	-	-
A 386		TRP	0.79	0.46Å CG with A 423 LEU HD13	Favored (85.07%) General / -57.4,-44.8	Favored (16.3%) *t60* chi angles: 170.5,56.7	0.04Å	-	-	-
A 387		SER	0.81	-	Favored (93.41%) General / -59.8,-45.1	Favored (46%) *m* chi angles: 290.7	0.10Å	-	-	-
A 388		ASP	0.8	-	Favored (96.3%) General / -62.0,-44.6	Favored (98.2%) *m-30* chi angles: 287.3,348.4	0.05Å	-	-	-
A 389		ILE	0.82	-	Favored (93.35%) Ile or Val / -62.3,-41.9	Favored (19.7%) *mm* chi angles: 298.7,289	0.10Å	-	-	-
A 390		LYS	0.79	-	Favored (87.48%) General / -66.5,-38.6	Favored (38.2%) *tttt* chi angles: 194.8,179.5,197.9,163.7	0.04Å	-	-	-
A 391		ARG	0.78	-	Favored (79.34%) General / -57.1,-48.1	Favored (69.9%) *ttt180* chi angles: 174.1,179.4,176.6,169.3	0.15Å	-	-	-
A 392		VAL	0.81	0.52Å HG13 with A 595 ARG HG3	Favored (90.26%) Ile or Val / -66.6,-43.6	Favored (63.8%) *t* chi angles: 171.3	0.07Å	-	-	-
A 393		PHE	0.8	-	Favored (76.73%) General / -60.3,-36.0	Favored (81.9%) *t80* chi angles: 183.2,259.1	0.03Å	-	-	-
A 394		ARG	0.77	-	Favored (98.83%) General / -63.4,-42.9	Favored (29.2%) *ttp80* chi angles: 200.8,168.2,54.1,74.3	0.08Å	-	-	-
**#**	**Alt**	**Res**	**High B**	**Clash > 0.4Å**	**Ramachandran**	**Rotamer**	**Cβ deviation**	**Bond lengths**	**Bond angles**	**Cis Peptides**
			Avg: 0.70	Clashscore: 10.3	Outliers: 12 of 650	Poor rotamers: 11 of 577	Outliers: 10 of 629	Outliers: 1 of 652	Outliers: 75 of 652	Non-Trans: 1 of 651
A					Favored	Favored (82.8%) *mt-*				

395		GLU	0.77	-	(95.69%) General / -60.1,-43.5	*10* chi angles: 294.3,168,11.7	0.05Å	-	-	-
A 396		ASP	0.77	-	Favored (74.58%) General / -65.6,-32.8	Favored (91.4%) *m-30* chi angles: 285.5,344.4	0.21Å	-	-	-
A 397		LEU	0.75	-	Favored (52.64%) General / -73.1,-7.7	Favored (19.7%) *mt* chi angles: 284.7,180.9	0.14Å	-	-	-
A 398		GLN	0.71	-	Favored (25.16%) General / -96.6,146.1	Favored (90%) *mt0* chi angles: 291.6,176,299.4	0.04Å	-	-	-
A 399		ALA	0.53	-	Allowed (0.65%) General / -40.8,116.0	-	0.01Å	-	-	-
A 400		SER	0.64	-	Favored (14.83%) General / -159.6,174.3	Favored (16.1%) *p* chi angles: 80.4	0.09Å	-	-	-
A 401		GLU	0.57	-	Favored (66.31%) General / -59.8,-27.2	Favored (99.5%) *mt-10* chi angles: 292.6,179,353.3	0.09Å	-	-	-
A 402		ARG	0.57	-	Favored (52.69%) General / -96.5,2.6	Favored (25.5%) *mmm160* chi angles: 300.8,314.9,300.4,168.2	0.09Å	-	-	-
A 403		LEU	0.61	0.40Å HD12 with A 405 TRP HE1	Allowed (1.58%) General / -132.9,65.6	Favored (3.2%) *tp* chi angles: 191.1,86	0.03Å	-	-	-
A 404		LEU	0.66	-	Favored (17.76%) General / -101.5,-13.7	Favored (84.4%) *mt* chi angles: 299.6,174.7	0.12Å	-	-	-
A 405		TRP	0.64	0.42Å CZ3 with A 406 LEU HD21	Favored (74.46%) General / -67.3,-33.1	Favored (28.5%) *p-90* chi angles: 60.1,284.1	0.10Å	-	-	-
A 406		LEU	0.74	0.42Å HD21 with A 405 TRP CZ3	Favored (29.54%) General / -93.9,140.6	Favored (84.4%) *mt* chi angles: 292.2,177.1	0.14Å	-	-	-
A 407		THR	0.77	-	Favored (19.51%) General / -64.1,159.8	Favored (38.4%) *p* chi angles: 54.4	0.07Å	-	-	-
A 408		LEU	0.72	-	Favored (78.54%) General / -59.0,-38.1	Favored (19.3%) *tp* chi angles: 189.2,54.9	0.09Å	-	-	-

A 409		GLU	0.77	-	Favored (87.56%) General / -58.0,-44.9	Favored (43%) *tt0* chi angles: 186.4,177.6,311.7	0.09Å	-	-	-
A 410		THR	0.78	-	Favored (84.49%) General / -67.0,-37.5	Favored (6.3%) *m* chi angles: 313.6	0.08Å	-	-	-
A 411		ARG	0.73	-	Favored (94.34%) General / -59.7,-43.0	Favored (16.2%) *mtm-85* chi angles: 297.2,187.6,290.7,239	0.06Å	-	-	-
A 412		GLN	0.77	-	Favored (89.75%) General / -58.9,-45.7	Favored (96.5%) *mt0* chi angles: 289.6,175.3,337.4	0.04Å	-	-	-
A 413		LYS	0.77	-	Favored (98.69%) General / -61.1,-43.6	Favored (98.3%) *mttt* chi angles: 295.1,179.7,181.2,174	0.05Å	-	-	-
A 414		ALA	0.8	-	Favored (95.53%) General / -61.7,-40.6	-	0.08Å	-	-	-
**#**	**Alt**	**Res**	**High B**	**Clash > 0.4Å**	**Ramachandran**	**Rotamer**	**Cβ deviation**	**Bond lengths**	**Bond angles**	**Cis Peptides**
			Avg: 0.70	Clashscore: 10.3	Outliers: 12 of 650	Poor rotamers: 11 of 577	Outliers: 10 of 629	Outliers: 1 of 652	Outliers: 75 of 652	Non-Trans: 1 of 651
A 415		ILE	0.78	-	Favored (93.79%) Ile or Val / -59.7,-46.0	Favored (93.9%) *mt* chi angles: 292.1,169.8	0.02Å	-	-	-
A 416		THR	0.78	-	Favored (94.85%) General / -59.9,-43.4	Favored (75.5%) *m* chi angles: 296.2	0.04Å	-	-	-
A 417		LYS	0.77	-	Favored (97%) General / -64.1,-42.1	Favored (64.9%) *tttt* chi angles: 171.8,163.8,184.9,177.6	0.09Å	-	-	-
A 418		LEU	0.78	-	Favored (92.61%) General / -61.2,-45.9	Favored (52.8%) *tp* chi angles: 174.1,64.6	0.07Å	-	-	-
A 419		GLU	0.76	-	Favored (67.21%) General / -52.8,-45.7	Favored (92.6%) *tt0* chi angles: 180.4,177.2,356	0.04Å	-	-	-
A 420		ALA	0.78	-	Favored (45.6%) General / -76.5,-5.0	-	0.05Å	-	-	-
A		LEU	0.76	-	Favored (2.64%)	Favored (63.2%) *tp*	0.05Å	-	-	-

421					General / -58.7,114.7	chi angles: 180.9,62				
A 422		LYS	0.71	-	Favored (27.96%) General / -84.0,146.4	Favored (96.2%) *mttt* chi angles: 297.4,182.7,177.3,181	0.04Å	-	-	-
A 423		LEU	0.74	0.46Å HD13 with A 386 TRP CG	Favored (51.28%) General / -126.2,143.1	Favored (2.3%) *tt* chi angles: 171,143.3	0.11Å	-	-	-
A 424		HIS	0.75	-	Favored (54.54%) General / -122.6,134.0	Favored (4.2%) *t70* chi angles: 199,36.8	0.05Å	-	OUTLIER(S) worst is ND1-CG-CD2: 4.3 σ	-
A 425		PHE	0.71	0.40Å CE1 with A 379 ILE HG23	Favored (13.93%) General / -109.0,-17.3	Favored (3.3%) *p90* chi angles: 47.9,67.7	0.03Å	-	-	-
A 426		ARG	0.69	-	Favored (18.36%) General / -112.2,108.9	Favored (71%) *ttt180* chi angles: 173.8,182,182.9,188.5	0.10Å	-	-	-
A 427		THR	0.58	-	Favored (5.94%) General / -62.1,-8.8	Favored (6.7%) *t* chi angles: 194.1	0.11Å	-	-	-
A 428		HIS	0.55	0.48Å HB2 with A 366 GLU CD	Favored (38.85%) General / -75.5,148.7	Favored (3%) *t-90* chi angles: 201.6,243.1	0.12Å	-	OUTLIER(S) worst is CA-CB-CG: 5.3 σ	-
A 429		ASP	0.54	-	Favored (43.7%) General / -97.7,127.5	Favored (21.8%) *t0* chi angles: 170.9,4	0.24Å	-	OUTLIER(S) worst is N-CA-CB: 4.4 σ	-
A 430		ASP	0.57	-	Favored (46.54%) General / -85.9,2.3	Favored (54.7%) *p0* chi angles: 62.1,5.7	0.15Å	-	-	-
A 431		ASN	0.53	-	OUTLIER (0.01)%) General / -60.0,71.8	Favored (28.7%) *t0* chi angles: 194.8,358.9	0.06Å	-	-	-
A 432		LYS	0.67	-	Allowed (1.18%) General / 58.4,65.9	Favored (88.1%) *mttt* chi angles: 303.8,175.8,177.5,179.2	0.09Å	-	-	-
A 433		LEU	0.69	-	Favored (26.33%) General / -47.5,-45.6	Favored (67.3%) *tp* chi angles: 179.5,61.3	0.04Å	-	-	-
A 434		ILE	0.67	-	Favored (87.84%) Ile or Val / -61.7,-40.7	Favored (89.4%) *mt* chi angles: 296.4,173.1	0.07Å	-	-	-

#	Alt	Res	High B	Clash > 0.4Å	Ramachandran	Rotamer	Cβ deviation	Bond lengths	Bond angles	Cis Peptides
			Avg: 0.70	Clashscore: 10.3	Outliers: 12 of 650	Poor rotamers: 11 of 577	Outliers: 10 of 629	Outliers: 1 of 652	Outliers: 75 of 652	Non-Trans: 1 of 651
A 435		ARG	0.66	-	Favored (99.55%) General / -62.5,-42.5	Favored (40.8%) *tpt170* chi angles: 180.5,68.8,175.1,173.6	0.07Å	-	-	-
A 436		GLN	0.68	-	Favored (74.53%) General / -61.6,-33.8	Favored (69.7%) *mt0* chi angles: 286,185.2,345.2	0.02Å	-	-	-
A 437		MET	0.7	-	Favored (27.02%) General / -108.4,7.3	Allowed (1.3%) *mpt* chi angles: 284.7,74.9,178	0.02Å	-	-	-
A 438		HIS	0.68	-	Favored (80.74%) General / -60.6,-37.2	Favored (88.9%) *t70* chi angles: 181.1,74	0.05Å	-	OUTLIER(S) worst is ND1-CG-CD2: 4.4 σ	-
A 439		GLY	0.75	-	Favored (85.39%) Glycine / -79.8,-0.6	-	-	-	-	-
A 440		LEU	0.74	0.51Å HD21 with A 361 PHE CE2	Favored (40.35%) General / -102.5,137.7	Favored (58.1%) *tp* chi angles: 181.2,63.6	0.06Å	-	-	-
A 441		ILE	0.72	-	Favored (63.91%) Ile or Val / -130.9,132.4	Favored (92.5%) *mt* chi angles: 297.3,172.2	0.02Å	-	-	-
A 442		LEU	0.77	0.47Å HD13 with A 117 PHE HB2	Favored (56.04%) General / -119.0,133.1	Favored (48%) *mt* chi angles: 299.4,167.3	0.07Å	-	-	-
A 443		ASN	0.74	-	Favored (40.32%) General / -103.9,138.5	Favored (70.6%) *m-40* chi angles: 297.9,296	0.11Å	-	-	-
A 444		MET	0.67	-	Favored (54.32%) General / -59.8,132.6	Favored (44.4%) *ttm* chi angles: 192.7,173.4,289	0.13Å	-	-	-
A 445		ASP	0.68	-	Favored (3.93%) General / 75.2,2.6	Favored (3.5%) *m-30* chi angles: 300.7,187.8	0.12Å	-	-	-
A 446		ARG	0.69	-	Allowed (0.8%) General / -119.2,68.0	Favored (97.7%) *mtt180* chi angles: 293,184.9,180.7,180.1	0.04Å	-	-	-
A		PHE	0.7	-	Favored (76.33%)	Favored (3.2%) *t80*	0.04Å	-	-	-

447					General / -59.0,-49.9	chi angles: 175.4,23				
A 448		TYR	0.71	-	Favored (95.73%) Pre-Pro / -60.8,-47.4	Favored (13.9%) *t80* chi angles: 185,106.9	0.18Å	-	OUTLIER(S) worst is CA-C-N: 5.0 σ	-
A 449		PRO	0.78	-	Favored (75.13%) Trans-Pro / -62.0,-35.8	Favored (99.3%) *Cg_exo* chi angles: 332.6,36.5,327.7	0.07Å	-	-	-
A 450		ASN	0.77	0.43Å O with A 454 VAL HG23	Favored (83.03%) General / -59.8,-48.1	Favored (99.6%) *m-40* chi angles: 288,342	0.07Å	-	-	-
A 451		LEU	0.78	-	Favored (90.9%) General / -58.9,-44.0	Favored (62%) *tp* chi angles: 180.3,63.4	0.02Å	-	-	-
A 452		VAL	0.79	-	Favored (98.47%) Ile or Val / -61.8,-44.2	Favored (66.2%) *t* chi angles: 171.6	0.05Å	-	-	-
A 453		SER	0.78	-	Favored (75.29%) General / -57.8,-38.3	Favored (55.8%) *m* chi angles: 292.8	0.03Å	-	-	-
A 454		VAL	0.79	0.43Å HG23 with A 450 ASN O	Favored (97.31%) Ile or Val / -64.3,-45.0	Favored (32.6%) *t* chi angles: 185.5	0.10Å	-	-	-
**#**	**Alt**	**Res**	**High B**	**Clash > 0.4Å**	**Ramachandran**	**Rotamer**	**Cβ deviation**	**Bond lengths**	**Bond angles**	**Cis Peptides**
			Avg: 0.70	Clashscore: 10.3	Outliers: 12 of 650	Poor rotamers: 11 of 577	Outliers: 10 of 629	Outliers: 1 of 652	Outliers: 75 of 652	Non-Trans: 1 of 651
A 455		LEU	0.79	-	Favored (95.29%) General / -60.2,-44.1	Favored (89.6%) *mt* chi angles: 292.5,168	0.02Å	-	-	-
A 456		GLN	0.75	-	Favored (93.61%) General / -63.2,-45.1	Favored (67.2%) *tt0* chi angles: 186.2,175.1,358.8	0.03Å	-	-	-
A 457		TRP	0.72	-	Favored (92.2%) General / -60.3,-45.8	Favored (71.1%) *t60* chi angles: 171,80.6	0.06Å	-	-	-
A 458		LYS	0.75	-	Favored (99.65%) General / -63.0,-43.1	Favored (87.6%) *tttt* chi angles: 185.6,174.4,180.6,179.4	0.04Å	-	-	-
A		THR	0.75	-	Favored (75.85%)	Favored (58.3%) *m*	0.05Å	-	-	-

459					General / -59.4,-50.0	chi angles: 295.4				
A 460		GLN	0.7	-	Favored (73.73%) General / -58.3,-36.8	Favored (38.7%) *tp40* chi angles: 186.2,63.3,78.1	0.09Å	-	-	-
A 461		ARG	0.67	-	Favored (83.86%) General / -57.4,-43.7	Favored (36.3%) *ttt90* chi angles: 189.1,196.3,177.3,90	0.09Å	-	-	-
A 462		GLY	0.75	-	Favored (56.82%) Glycine / -71.7,-41.3	-	-	-	-	-
A 463		LEU	0.72	0.41Å HD13 with A 294 ILE O	Favored (85.14%) General / -60.8,-38.4	Favored (54.2%) *mt* chi angles: 300.8,169.6	0.06Å	-	-	-
A 464		ALA	0.75	-	Favored (52.47%) General / -69.3,-9.3	-	0.05Å	-	-	-
A 465		LYS	0.69	-	Favored (57.37%) General / -92.2,1.2	Favored (72.9%) *mmtt* chi angles: 298.3,295.9,182.6,181.2	0.05Å	-	-	-
A 466		LEU	0.71	-	Favored (99.55%) General / -62.5,-43.1	Favored (32.5%) *tp* chi angles: 185.6,55.9	0.04Å	-	-	-
A 467		MET	0.69	-	Favored (34.08%) General / -103.3,13.3	Favored (98%) *mmm* chi angles: 293.3,301.8,293.2	0.03Å	-	-	-
A 468		THR	0.7	-	Favored (10.66%) General / -99.2,167.5	Favored (47.7%) *p* chi angles: 66.1	0.15Å	-	-	-
A 469		GLU	0.67	-	Favored (42.36%) Pre-Pro / -83.2,139.9	Favored (89.3%) *tt0* chi angles: 184,173.8,5.1	0.05Å	-	-	-
A 470		PRO	0.7	-	Favored (20.62%) Trans-Pro / -63.4,127.7	Favored (20.1%) *Cg_exo* chi angles: 342.4,31.9,326.5	0.05Å	-	-	-
A 471		VAL	0.67	-	Favored (23.02%) Ile or Val / -119.9,108.3	Favored (10.9%) *t* chi angles: 189.7	0.14Å	-	-	-
A 472		ALA	0.61	0.41Å HA with A 486 LYS HD3	Favored (66.13%) General / -65.4,-50.2	-	0.21Å	-	OUTLIER(S) worst is N-CA-CB: 4.5 σ	-
					Favored					

#	Alt	Res	High B	Clash > 0.4Å	Ramachandran	Rotamer	Cβ deviation	Bond lengths	Bond angles	Cis Peptides
A 473		GLU	0.66	-	(67.23%) General / -66.3,-26.3	Favored (3.1%) *pt0* chi angles: 50.7,200.1,68.7	0.23Å	-	OUTLIER(S) worst is C-CA-CB: 4.6 σ	-
A 474		ASP	0.65	-	Allowed (0.62%) General / -96.2,-72.9	Favored (17.1%) *m-30* chi angles: 310.1,319.9	0.11Å	-	-	-
			Avg: 0.70	Clashscore: 10.3	Outliers: 12 of 650	Poor rotamers: 11 of 577	Outliers: 10 of 629	Outliers: 1 of 652	Outliers: 75 of 652	Non-Trans: 1 of 651
A 475		GLU	0.66	-	Favored (2.52%) General / -67.5,107.6	Favored (5.1%) *mm-30* chi angles: 301.5,259.2,5.9	0.12Å	-	OUTLIER(S) worst is CG-CD-OE1: 4.9 σ	-
A 476		VAL	0.69	-	Favored (10.52%) Ile or Val / -98.5,1.1	Favored (26.9%) *m* chi angles: 299	0.08Å	-	-	-
A 477		HIS	0.51	-	Favored (57.95%) General / -78.9,-7.4	Favored (36.7%) *p-80* chi angles: 75.3,287.1	0.05Å	-	OUTLIER(S) worst is ND1-CG-CD2: 4.2 σ	-
A 478		LYS	0.5	0.55Å HZ2 with A 182 ILE HA	Favored (15.28%) General / -74.4,-51.3	Favored (72%) *mmtt* chi angles: 295.5,292.4,184.3,176.8	0.08Å	-	-	-
A 479		LEU	0.49	-	Favored (8.28%) Pre-Pro / -156.7,136.2	Favored (43.1%) *tp* chi angles: 185,60.8	0.07Å	-	-	-
A 480		PRO	0.53	-	Favored (16.53%) Trans-Pro / -76.7,-6.0	Favored (85.8%) *Cg_exo* chi angles: 333.8,35.3,328.8	0.08Å	-	-	-
A 481		HIS	0.51	-	Favored (6.14%) General / -112.4,173.9	Favored (52.8%) *m-70* chi angles: 280.4,281.1	0.12Å	-	OUTLIER(S) worst is ND1-CG-CD2: 4.0 σ	-
A 482		TYR	0.72	0.54Å CZ with A 526 GLY HA2	Allowed (0.35%) General / -32.8,121.1	Favored (85.5%) *t80* chi angles: 176.5,73.6	0.09Å	-	-	-
A 483		GLU	0.73	-	Favored (28.72%) General / -102.2,114.5	Favored (17.9%) *mt-10* chi angles: 297.5,199.4,64.5	0.07Å	-	OUTLIER(S) worst is C-N-CA: 4.6 σ	-
A 484		ILE	0.74	0.49Å HD12 with A 534 TYR CZ	Favored (83.72%) Ile or Val / -57.7,-48.3	OUTLIER (0.2%) chi angles: 38,185	0.11Å	-	-	-
				0.57Å	Favored	Allowed (0.8%)			OUTLIER(S)	

A 485		GLN	0.7	HG3 with A 183 LEU HD22	(86.89%) General / -63.5,-46.3	*pm20* chi angles: 51,273.9,338.9	0.08Å	-	worst is O-C-N: 4.0 σ	-
A 486		LYS	0.71	0.41Å HD3 with A 472 ALA HA	Favored (56.17%) General / -88.4,0.5	Favored (85.6%) *mttt* chi angles: 289.9,177,172.1,188.4	0.20Å	-	-	-
A 487		ASN	0.75	-	Allowed (1.08%) General / 39.5,62.9	Favored (64.5%) *t0* chi angles: 196.3,34.7	0.22Å	-	-	-
A 488		ARG	0.72	-	Favored (17.71%) General / -147.3,131.8	Favored (59.9%) *ttp80* chi angles: 187.4,187.7,61.7,94.6	0.06Å	-	-	-
A 489		ILE	0.75	-	Favored (13.91%) Ile or Val / -100.4,143.5	Favored (13.2%) *pt* chi angles: 52,177	0.08Å	-	-	-
A 490		GLN	0.75	-	Favored (51.35%) General / -126.4,133.0	Favored (47.7%) *tt0* chi angles: 191.2,175.9,318	0.11Å	-	-	-
A 491		VAL	0.76	0.44Å HB with A 496 LEU HD21	Favored (32%) Pre-Pro / -123.2,108.4	Favored (14.2%) *m* chi angles: 291.7	0.15Å	-	-	-
A 492		PRO	0.76	-	Favored (93.78%) Trans-Pro / -62.5,143.9	Favored (42.8%) *Cg_exo* chi angles: 338.2,26.6,338.5	0.08Å	-	-	-
A 493		ILE	0.71	0.43Å HD13 with A 370 HIS HB2	Favored (18.8%) Ile or Val / -52.9,-33.1	Favored (49.4%) *mt* chi angles: 287.3,174.3	0.10Å	-	-	-
A 494		THR	0.75	-	Favored (52.28%) General / -55.8,-27.6	Favored (42.3%) *p* chi angles: 55	0.08Å	-	-	-
**#**	**Alt**	**Res**	**High B**	**Clash > 0.4Å**	**Ramachandran**	**Rotamer**	**Cβ deviation**	**Bond lengths**	**Bond angles**	**Cis Peptides**
			Avg: 0.70	Clashscore: 10.3	Outliers: 12 of 650	Poor rotamers: 11 of 577	Outliers: 10 of 629	Outliers: 1 of 652	Outliers: 75 of 652	Non-Trans: 1 of 651
A 495		PHE	0.72	-	Favored (12.25%) General / -99.3,-30.4	Favored (98.4%) *m-80* chi angles: 298,96.1	0.04Å	-	OUTLIER(S) worst is CA-CB-CG: 6.8 σ	-
A 496		LEU	0.71	0.45Å O with A 502 TRP HB3	Favored (86.58%) General / -64.7,-45.4	Favored (52.1%) *mt* chi angles: 304.7,174	0.03Å	-	-	-
A					Favored	Favored (31.5%)			OUTLIER(S)	

497		GLN	0.51	-	(55.6%)<br>General /<br>-93.4,3.4	*mm110*<br>chi angles:<br>289.8,297.7,101.6	0.17Å	-	worst is C-N-CA: 4.0 σ	-
A<br>498		ALA	0.58	0.67Å<br>HB2 with A<br>87 LEU<br>HD11	Favored<br>(22.02%)<br>General /<br>-62.7,124.8	-	0.26Å	-	OUTLIER(S)<br>worst is N-CA-CB: 5.1 σ	-
A<br>499		ARG	0.52	-	Favored<br>(37.7%)<br>General /<br>-70.3,-6.8	Favored (97.4%)<br>*mtt180*<br>chi angles:<br>292.6,184.3,179.5,173.4	0.17Å	-	-	-
A<br>500		PHE	0.5	0.52Å<br>HB2 with A<br>83 TYR CE1	Favored<br>(17.19%)<br>General /<br>-94.3,-25.1	Favored (91.3%) *m-80*<br>chi angles: 292.5,99.4	0.03Å	-	-	-
A<br>501		PHE	0.71	0.49Å<br>CE2 with A<br>518 LEU<br>HD11	Allowed<br>(0.15%)<br>General /<br>-94.2,-99.4	Favored (5.5%) *m-10*<br>chi angles: 282,343.4	0.20Å	-	OUTLIER(S)<br>worst is CA-CB-CG: 4.8 σ	-
A<br>502		TRP	0.71	0.46Å<br>CZ3 with A<br>385 THR<br>HG21	Favored<br>(28.08%)<br>General /<br>-95.7,142.4	Favored (40.9%) *t-100*<br>chi angles: 176.1,272.4	0.10Å	-	-	-
A<br>503		ASP	0.72	-	Favored<br>(7.31%)<br>Pre-Pro /<br>-148.3,129.9	Favored (9%) *t0*<br>chi angles: 182.7,302.7	0.14Å	-	OUTLIER(S)<br>worst is CA-C-N: 5.3 σ	-
A<br>504		PRO	0.77	-	Favored<br>(48.74%)<br>Trans-Pro /<br>-66.3,-26.8	Favored (59.5%)<br>*Cg_endo*<br>chi angles:<br>26.4,323.8,31.8	0.06Å	-	-	-
A<br>505		ALA	0.75	-	Favored<br>(58.7%)<br>General /<br>-76.3,-19.0	-	0.07Å	-	-	-
A<br>506		TYR	0.71	-	Favored<br>(95.44%)<br>Pre-Pro /<br>-63.6,147.6	Favored (9.6%) *m-10*<br>chi angles: 281.5,4.8	0.11Å	-	-	-
A<br>507		PRO	0.77	-	Favored<br>(11.18%)<br>Trans-Pro /<br>-45.6,138.0	Favored (20.8%)<br>*Cg_exo*<br>chi angles:<br>342.1,28.6,331.4	0.08Å	-	-	-
A<br>508		ASN	0.76	-	Favored<br>(75.09%)<br>General /<br>-57.5,-38.7	Favored (78.7%) *m-40*<br>chi angles: 289.1,316.7	0.03Å	-	OUTLIER(S)<br>worst is CA-CB-CG: 6.0 σ	-
A<br>509		GLY	0.8	-	Favored<br>(56.22%)<br>Glycine /<br>-54.3,-35.8	-	-	-	-	-
A<br>510		LEU	0.78	-	Favored<br>(32.85%)<br>General /<br>-73.9,-48.1	Favored (95.9%) *mt*<br>chi angles: 294.3,172.2	0.11Å	-	-	-

A 511		LYS	0.77	-	Favored (94.12%) General / -60.9,-40.7	Favored (54.8%) *mtmt* chi angles: 293.8,191.7,293.3,187.1	0.03Å	-	-	-
A 512		TYR	0.78	-	Favored (86.26%) General / -66.7,-38.1	Favored (55.8%) *m-80* chi angles: 285,102.1	0.08Å	-	-	-
A 513		ALA	0.8	-	Favored (63.8%) General / -69.3,-23.5	-	0.11Å	-	-	-
A 514		THR	0.77	0.63Å HA with A 634 LEU HD11	Allowed (0.17%) General / -92.5,-90.9	Favored (50.9%) *p* chi angles: 56.4	0.18Å	-	-	-
**#**	**Alt**	**Res**	**High B**	**Clash > 0.4Å**	**Ramachandran**	**Rotamer**	**Cβ deviation**	**Bond lengths**	**Bond angles**	**Cis Peptides**
			Avg: 0.70	Clashscore: 10.3	Outliers: 12 of 650	Poor rotamers: 11 of 577	Outliers: 10 of 629	Outliers: 1 of 652	Outliers: 75 of 652	Non-Trans: 1 of 651
A 515		LEU	0.78	0.64Å HD21 with A 382 LEU CD1	Favored (82.8%) General / -67.4,-37.2	Favored (61.3%) *tp* chi angles: 181.1,62.9	0.08Å	-	-	-
A 516		GLY	0.81	-	Favored (35.59%) Glycine / -49.5,-43.2	-	-	-	-	-
A 517		VAL	0.81	-	Favored (96.99%) Ile or Val / -62.5,-46.2	Favored (85.9%) *t* chi angles: 177.4	0.07Å	-	-	-
A 518		LEU	0.79	0.49Å HD11 with A 501 PHE CE2	Favored (96.52%) General / -61.4,-41.0	Favored (50.2%) *mt* chi angles: 296,165.1	0.06Å	-	-	-
A 519		LEU	0.8	-	Favored (95.78%) General / -61.2,-45.1	Favored (38.5%) *mt* chi angles: 283.7,172.1	0.13Å	-	-	-
A 520		ALA	0.81	0.46Å HB1 with A 589 ALA HA	Favored (95.65%) General / -64.9,-41.4	-	0.06Å	-	-	-
A 521		ARG	0.77	-	Favored (77.13%) General / -58.0,-39.0	Favored (82.9%) *ttp80* chi angles: 183.2,183,60.9,79.2	0.05Å	-	-	-
A 522		GLN	0.78	-	Favored (84.01%) General / -67.8,-40.5	Favored (54.9%) *mm-40* chi angles: 297.7,296.8,347.8	0.09Å	-	-	-
					Favored	Favored (53.6%) *mtt*			OUTLIER(S)	

#	Alt	Res	High B	Clash > 0.4Å	Ramachandran	Rotamer	Cβ deviation	Bond lengths	Bond angles	Cis Peptides
A 523		MET	0.78	-	(96.75%) General / -60.4,-43.2	chi angles: 287.4,181.7,190.6	0.15Å	-	worst is CG-SD-CE: 6.2 σ	-
A 524		LEU	0.8	0.42Å HD21 with A 588 GLY HA3	Favored (70.99%) General / -66.1,-30.5	Allowed (0.7%) *mp* chi angles: 268.7,83.7	0.07Å	-	-	-
A 525		HIS	0.78	-	Favored (16.47%) General / -62.3,-11.7	Favored (8.2%) *m-70* chi angles: 276.7,313.4	0.03Å	-	OUTLIER(S) worst is CB-CG-ND1: 4.1 σ	-
A 526		GLY	0.79	0.54Å HA2 with A 482 TYR CZ	Favored (80.01%) Glycine / -75.9,-5.4	-	-	-	-	-
A 527		PHE	0.77	0.43Å HB3 with A 686 TYR CZ	Allowed (1%) General / -145.3,2.9	Favored (100%) *m-80* chi angles: 297.5,272.7	0.14Å	-	-	-
A 528		ASP	0.78	-	OUTLIER (0.02)%) General / -60.3,-173.4	Favored (2.2%) *p0* chi angles: 85.7,3.5	0.33Å	-	-	-
A 529		GLY	0.78	-	Favored (62.25%) Glycine / -61.9,-22.3	-	-	-	-	-
A 530		VAL	0.74	-	Favored (6.25%) Ile or Val / -95.4,-55.3	Favored (81.4%) *t* chi angles: 176.6	0.04Å	-	-	-
A 531		GLY	0.77	-	Favored (74.97%) Glycine / -68.3,-23.6	-	-	-	-	-
A 532		ARG	0.71	0.47Å NH2 with A 549 GLU OE2	Favored (63.48%) General / -69.0,-15.4	Favored (30.5%) *ptt90* chi angles: 75.5,162.8,186,83.6	0.12Å	-	-	-
A 533		ARG	0.7	-	Favored (43.84%) General / -85.0,2.3	Favored (95.5%) *mtt-85* chi angles: 288.5,179.9,177.6,272.7	0.08Å	-	-	-
A 534		TYR	0.7	0.49Å CZ with A 484 ILE HD12	Favored (55.81%) General / -118.9,130.6	Favored (50%) *m-80* chi angles: 299.8,258.8	0.16Å	-	-	-
**#**	**Alt**	**Res**	**High B**	**Clash > 0.4Å**	**Ramachandran**	**Rotamer**	**Cβ deviation**	**Bond lengths**	**Bond angles**	**Cis Peptides**
			Avg: 0.70	Clashscore: 10.3	Outliers: 12 of 650	Poor rotamers: 11 of 577	Outliers: 10 of 629	Outliers: 1 of 652	Outliers: 75 of 652	Non-Trans: 1 of 651
A 535		ASP	0.74	-	Allowed (0.06%) General /	Favored (21.6%) *p0* chi angles: 75.5,191.9	0.30Å	-	-	-

					-64.2,-172.1					
A 536		ALA	0.77	-	Favored (62.26%) General / -71.5,-18.7	-	0.09Å	-	-	-
A 537		TYR	0.69	-	Favored (44.91%) General / -97.3,-1.3	Favored (86.2%) *m-80* chi angles: 290.7,97.7	0.10Å	-	-	-
A 538		GLY	0.73	0.45Å HA3 with A 485 GLN O	Favored (55.35%) Glycine / 97.5,9.2	-	-	-	-	-
A 539		TYR	0.65	-	Favored (47.68%) General / -101.5,131.3	Favored (6.5%) *m-80* chi angles: 286.9,61.8	0.12Å	-	-	-
A 540		LYS	0.7	-	Favored (4.23%) General / -74.5,101.7	Favored (8.6%) *ttpp* chi angles: 195.9,180.9,71.1,71.7	0.06Å	-	-	-
A 541		ASN	0.67	-	Allowed (1.25%) General / -171.4,139.2	Favored (41.8%) *t0* chi angles: 194,339	0.08Å	-	-	-
A 542		ASN	0.69	-	Favored (4.74%) General / -68.0,112.0	Favored (79.7%) *m-40* chi angles: 297.3,338.6	0.08Å	-	-	-
A 543		TRP	0.68	-	Favored (14.74%) General / -111.9,-3.9	Favored (3.7%) *p-90* chi angles: 53.4,295.1	0.10Å	-	-	-
A 544		TRP	0.72	-	Favored (20.9%) General / -112.8,157.8	Favored (47.3%) *m-10* chi angles: 296.2,354.7	0.10Å	-	-	-
A 545		ASP	0.76	-	Favored (48.62%) General / -62.9,147.9	Favored (41.3%) *m-30* chi angles: 292.4,3.2	0.07Å	-	-	-
A 546		HIS	0.68	-	Favored (67.31%) General / -56.8,-34.0	Favored (86.3%) *t70* chi angles: 183.3,71.8	0.02Å	-	OUTLIER(S) worst is ND1-CG-CD2: 4.3 σ	-
A 547		THR	0.73	-	Favored (88.99%) General / -58.5,-43.4	Favored (98.7%) *m* chi angles: 300.2	0.03Å	-	-	-
A 548		SER	0.77	-	Favored (77.29%) General / -69.7,-39.1	Favored (11.5%) *m* chi angles: 307.6	0.04Å	-	-	-
A		GLU	0.74	0.47Å OE2 with A	Favored (99.58%)	Favored (46.8%) *mt-10*	0.07Å	-	-	-

549				532 ARG NH2	General / -62.3,-43.1	chi angles: 292.9,198.4,341.4				
A 550		SER	0.78	-	Favored (85.76%) General / -64.0,-37.0	Favored (9.7%) *p* chi angles: 82.4	0.21Å	-	-	-
A 551		SER	0.77	-	Favored (93.8%) General / -64.2,-39.3	Favored (19.9%) *m* chi angles: 287.6	0.10Å	-	-	-
A 552		TYR	0.75	0.47Å CZ with A 685 LEU HD21	Favored (85.78%) General / -61.3,-47.5	Favored (34.1%) *t80* chi angles: 178.8,98.2	0.07Å	-	-	-
A 553		SER	0.78	-	Favored (82.27%) General / -62.1,-36.6	Favored (86.6%) *p* chi angles: 67.9	0.17Å	-	-	-
A 554		ARG	0.72	-	Favored (86.26%) General / -59.6,-40.1	Favored (90.8%) *mtm-85* chi angles: 286.2,197.8,286.7,277.9	0.08Å	-	-	-
**#**	**Alt**	**Res**	**High B**	**Clash > 0.4Å**	**Ramachandran**	**Rotamer**	**Cβ deviation**	**Bond lengths**	**Bond angles**	**Cis Peptides**
			Avg: 0.70	Clashscore: 10.3	Outliers: 12 of 650	Poor rotamers: 11 of 577	Outliers: 10 of 629	Outliers: 1 of 652	Outliers: 75 of 652	Non-Trans: 1 of 651
A 555		ARG	0.71	-	Favored (93.97%) General / -65.2,-42.1	Favored (13.2%) *mmp80* chi angles: 299.5,289.8,81.2,73.5	0.09Å	-	-	-
A 556		THR	0.78	-	Favored (80.42%) General / -61.4,-36.5	Favored (66.5%) *p* chi angles: 62.9	0.18Å	-	-	-
A 557		GLN	0.73	-	Favored (65.2%) General / -56.1,-33.5	Favored (97.9%) *mt0* chi angles: 293.3,176.2,329.2	0.14Å	-	-	-
A 558		CYS	0.77	-	Favored (86.76%) General / -59.2,-40.7	Favored (15.9%) *t* chi angles: 192.3	0.06Å	-	-	-
A 559		PHE	0.71	-	Favored (56%) General / -78.0,-18.3	Favored (29.9%) *m-80* chi angles: 303.8,77.9	0.14Å	-	-	-
A 560		GLN	0.7	0.41Å HA with A 576 ASP HB2	Favored (8.91%) General / -68.9,-56.1	Favored (93.4%) *mt0* chi angles: 294,178.6,342.9	0.09Å	-	-	-
A 561		GLU	0.68	-	Favored (86.34%) General / -57.9,-45.5	Favored (61.8%) *mt-10* chi angles: 293.5,157.5,357.7	0.08Å	-	-	-

#	Alt	Res	High B	Clash > 0.4Å	Ramachandran	Rotamer	Cβ deviation	Bond lengths	Bond angles	Cis Peptides
A 562		GLN	0.68	-	Favored (78.8%) General / -58.7,-38.6	Favored (36.8%) *tt0* chi angles: 169.4,183.1,5.5	0.07Å	-	-	-
A 563		TYR	0.66	0.55Å CZ with A 583 ILE HG23	Favored (42.78%) General / -97.0,9.2	Favored (23.3%) *m-80* chi angles: 285.2,69.2	0.10Å	-	-	-
A 564		ALA	0.66	-	Allowed (1.33%) General / -128.0,-51.5	-	0.12Å	-	-	-
A 565		ILE	0.46	-	Favored (6.75%) Ile or Val / -68.1,115.7	Favored (88.2%) *mt* chi angles: 298.2,168.4	0.05Å	-	-	-
A 566		PHE	0.36	-	Allowed (0.39%) General / 82.7,15.4	Favored (13.8%) *m-80* chi angles: 307.9,319	0.15Å	-	-	-
A 567		VAL	0.54	-	Favored (51.22%) Ile or Val / -129.0,120.2	Allowed (2%) *t* chi angles: 194.3	0.03Å	-	-	-
A 568		GLU	0.65	-	Favored (31.49%) General / -156.2,167.1	Favored (25.4%) *pt0* chi angles: 60.4,181.8,358.9	0.02Å	-	-	-
A 569		TYR	0.68	-	Favored (30.98%) General / -137.7,131.2	Favored (21.7%) *p90* chi angles: 51.3,79.3	0.09Å	-	-	-
A 570		LYS	0.65	-	Favored (18.15%) General / 48.6,41.9	Favored (18.8%) *mmtm* chi angles: 303.5,292.8,188,314.9	0.10Å	-	-	-
A 571		GLY	0.64	-	Favored (74.15%) Glycine / 70.9,26.3	-	-	-	-	-
A 572		LYS	0.67	-	Favored (19.57%) Pre-Pro / -139.7,138.1	Favored (96.7%) *mttt* chi angles: 296.6,183.6,178.2,184.5	0.06Å	-	-	-
A 573		PRO	0.68	-	Favored (63.69%) Trans-Pro / -69.2,156.8	Favored (77.4%) *Cg_endo* chi angles: 28.6,322.5,32.3	0.08Å	-	-	-
A 574		VAL	0.66	-	Favored (26.65%) Ile or Val / -95.6,136.4	Favored (4.5%) *t* chi angles: 192	0.10Å	-	-	-
**#**	**Alt**	**Res**	**High B**	**Clash > 0.4Å**	**Ramachandran**	**Rotamer**	**Cβ deviation**	**Bond lengths**	**Bond angles**	**Cis Peptides**

			Avg: 0.70	Clashscore: 10.3	Outliers: 12 of 650	Poor rotamers: 11 of 577	Outliers: 10 of 629	Outliers: 1 of 652	Outliers: 75 of 652	Non-Trans: 1 of 651
A 575		GLN	0.65	-	Favored (17.32%) General / -93.4,105.2	Favored (98.1%) *mm-40* chi angles: 295.3,301.5,305.2	0.08Å	-	-	-
A 576		ASP	0.69	0.41Å HB2 with A 560 GLN HA	Favored (74.28%) General / -64.8,-48.5	Favored (10.9%) *p0* chi angles: 60.5,297.2	0.17Å	-	OUTLIER(S) worst is N-CA-CB: 4.5 σ	-
A 577		LYS	0.69	-	Favored (96.18%) General / -60.6,-44.6	Favored (97.9%) *mttt* chi angles: 291.1,184.9,177.3,177.1	0.04Å	-	-	-
A 578		ASP	0.69	-	Favored (84.97%) General / -64.5,-36.7	Favored (2.3%) *m-30* chi angles: 300,12.9	0.07Å	-	-	-
A 579		LEU	0.7	0.46Å HD22 with A 649 LEU HB2	Favored (40.81%) General / -93.1,-9.1	Favored (91.2%) *mt* chi angles: 299,177.9	0.10Å	-	-	-
A 580		LEU	0.72	-	Favored (34.02%) General / -48.4,-43.5	Favored (51.9%) *tp* chi angles: 176.5,56.3	0.03Å	-	-	-
A 581		ARG	0.72	-	Favored (91.44%) General / -58.9,-44.8	Favored (44.8%) *ptt-90* chi angles: 67.2,181.4,182,284.9	0.10Å	-	-	-
A 582		ARG	0.71	-	Favored (54.31%) General / -77.2,-29.7	Favored (98%) *mtt180* chi angles: 289,179.8,178.8,174.2	0.03Å	-	-	-
A 583		ILE	0.72	0.55Å HG23 with A 563 TYR CZ	Favored (87.57%) Ile or Val / -61.3,-48.7	Favored (70.5%) *mt* chi angles: 289,171.3	0.13Å	-	-	-
A 584		VAL	0.78	-	Favored (91.72%) Ile or Val / -59.3,-44.2	Favored (59.1%) *t* chi angles: 170.7	0.02Å	-	-	-
A 585		ALA	0.78	-	Favored (81.22%) General / -60.7,-48.6	-	0.06Å	-	-	-
A 586		ASP	0.77	-	Favored (96.64%) General / -60.9,-44.7	Favored (79.1%) *m-30* chi angles: 284.5,177.2	0.03Å	-	-	-
A 587		ASN	0.75	-	Favored (3.63%) General /	Favored (10.5%) *m-40*	0.09Å	-	OUTLIER(S) worst is CA-CB-CG: 11.3	-

#	Alt	Res	High B	Clash > 0.4Å	Ramachandran	Rotamer	Cβ deviation	Bond lengths	Bond angles	Cis Peptides
					-61.9,-60.4	chi angles: 270.2,270.7			σ	
A 588		GLY	0.79	0.42Å HA3 with A 524 LEU HD21	Favored (82.48%) Glycine / -78.0,-14.3	-	-	-	-	-
A 589		ALA	0.79	0.46Å HA with A 520 ALA HB1	Favored (97.14%) General / -62.3,-44.2	-	0.11Å	-	-	-
A 590		LEU	0.78	-	Favored (89.36%) General / -59.8,-46.4	Favored (7%) *tp* chi angles: 179,81.1	0.10Å	-	-	-
A 591		ASP	0.78	-	Favored (93.61%) General / -62.1,-45.4	Favored (96.8%) *m-30* chi angles: 289.9,343.7	0.14Å	-	-	-
A 592		ILE	0.8	-	Favored (95.03%) Ile or Val / -63.1,-46.8	Allowed (0.6%) *mm* chi angles: 298,330.5	0.04Å	-	-	-
A 593		ALA	0.82	-	Favored (95.32%) General / -60.9,-41.0	-	0.06Å	-	-	-
A 594		TYR	0.8	-	Favored (78.47%) General / -65.0,-47.1	Favored (31.5%) *t80* chi angles: 191.6,64.9	0.05Å	-	-	-
**#**	**Alt**	**Res**	**High B**	**Clash > 0.4Å**	**Ramachandran**	**Rotamer**	**Cβ deviation**	**Bond lengths**	**Bond angles**	**Cis Peptides**
			Avg: 0.70	Clashscore: 10.3	Outliers: 12 of 650	Poor rotamers: 11 of 577	Outliers: 10 of 629	Outliers: 1 of 652	Outliers: 75 of 652	Non-Trans: 1 of 651
A 595		ARG	0.78	0.52Å HG3 with A 392 VAL HG13	Favored (78.85%) General / -63.7,-34.9	Allowed (1.6%) *tpp80* chi angles: 162.1,61.5,83.4,110.6	0.24Å	-	-	-
A 596		ALA	0.82	0.47Å HA with A 392 VAL HG21	Favored (96.62%) General / -63.7,-40.4	-	0.03Å	-	-	-
A 597		TYR	0.78	0.42Å CZ with A 601 LEU HD11	Favored (93.23%) General / -62.3,-39.5	Favored (33.7%) *t80* chi angles: 182.6,55.1	0.12Å	-	-	-
A 598		GLN	0.73	-	Favored (77.61%) General / -66.5,-34.3	Favored (23.2%) *mm110* chi angles: 302.4,316.3,118.1	0.12Å	-	-	-
A 599		GLN	0.72	-	Favored (81.81%) General / -66.6,-44.5	Favored (40.9%) *mm-40* chi angles: 290.3,292.7,357.4	0.08Å	-	-	-

A 600		TRP	0.66	0.47Å O with A 610 ILE HG23	Favored (74.37%) General / -61.9,-33.5	Favored (36.8%) *t-100* chi angles: 181.7,276.9	0.12Å	-	-	-
A 601		LEU	0.63	0.42Å HA with A 610 ILE O	Favored (3.15%) General / -106.9,35.0	Favored (6.5%) *mt* chi angles: 283.4,190.3	0.05Å	-	-	-
A 602		LYS	0.59	0.56Å O with A 610 ILE HG22	Favored (54.73%) General / -63.4,133.8	Favored (83.4%) *tttt* chi angles: 188.2,178.3,182.5,181.8	0.07Å	-	-	-
A 603		ASN	0.43	-	Favored (65.41%) General / -59.7,-26.3	Favored (33.7%) *p0* chi angles: 64.2,331.9	0.14Å	-	-	-
A 604		ALA	0.47	-	Favored (48.13%) General / -94.8,-3.9	-	0.13Å	-	-	-
A 605		ALA	0.37	0.43Å HB3 with A 610 ILE HG21	Favored (13.08%) General / -89.1,167.5	-	0.24Å	-	OUTLIER(S) worst is N-CA-CB: 5.8 σ	-
A 606		GLU	0.36	-	Favored (63.86%) General / -74.2,-34.5	Favored (66%) *mt-10* chi angles: 305.3,181.6,349.2	0.18Å	-	OUTLIER(S) worst is N-CA-CB: 4.9 σ	-
A 607		THR	0.37	-	Favored (31.29%) Pre-Pro / -89.5,135.1	Favored (88.7%) *m* chi angles: 298.4	0.03Å	-	OUTLIER(S) worst is CA-C-N: 4.1 σ	-
A 608		PRO	0.58	-	Favored (88.28%) Trans-Pro / -58.5,-29.2	Favored (91.1%) *Cg_exo* chi angles: 331,33.5,334.6	0.07Å	-	-	-
A 609		VAL	0.6	-	Favored (22.02%) Ile or Val / -60.1,-21.4	Allowed (1.5%) *m* chi angles: 312.1	0.12Å	-	-	-
A 610		ILE	0.53	0.56Å HG22 with A 602 LYS O	Allowed (1.04%) Ile or Val / -84.1,-64.4	Allowed (1.7%) *mm* chi angles: 307.1,276.6	0.08Å	-	OUTLIER(S) worst is CA-C-N: 6.0 σ	-
A 611		TYR	0.43	0.45Å C with A 610 ILE O	Favored (72.14%) General / -56.0,-39.6	Favored (8.4%) *t80* chi angles: 165.6,53.3	0.03Å	-	-	-
A 612		GLN	0.61	-	Allowed (0.08%) General / -177.5,113.5	Favored (37%) *tt0* chi angles: 179.4,170.7,72	0.17Å	-	-	-
A		ARG	0.64	-	Favored (47.31%)	Favored (81.2%) *mtp85*	0.13Å	-	-	-

#	Alt	Res	High B	Clash > 0.4Å	Ramachandran	Rotamer	Cβ deviation	Bond lengths	Bond angles	Cis Peptides
613					General / -64.8,150.5	chi angles: 294.9,181,67.3,92				
A 614		GLU	0.64	-	Favored (2.37%) General / -51.4,152.8	Favored (32.2%) *mt-10* chi angles: 305.5,171.5,307.1	0.07Å	-	-	-
**#**	**Alt**	**Res**	**High B**	**Clash > 0.4Å**	**Ramachandran**	**Rotamer**	**Cβ deviation**	**Bond lengths**	**Bond angles**	**Cis Peptides**
			Avg: 0.70	Clashscore: 10.3	Outliers: 12 of 650	Poor rotamers: 11 of 577	Outliers: 10 of 629	Outliers: 1 of 652	Outliers: 75 of 652	Non-Trans: 1 of 651
A 615		ARG	0.68	-	Favored (17.85%) General / -88.0,160.5	Favored (80.6%) *mtm180* chi angles: 296.5,180.4,293.3,185.7	0.02Å	-	-	-
A 616		LEU	0.71	-	Favored (62.35%) Pre-Pro / -81.6,161.2	Favored (4.4%) *mt* chi angles: 299.3,201.7	0.07Å	-	OUTLIER(S) worst is CA-C-N: 4.3 σ	-
A 617		PRO	0.69	-	OUTLIER (0.01)%) Trans-Pro / -106.7,-45.6	Favored (58.5%) *Cg_endo* chi angles: 26.3,341.2,3.7	0.04Å	-	-	-
A 618		LEU	0.67	-	Favored (54.56%) General / -77.8,-19.7	Favored (86%) *mt* chi angles: 290.4,173.1	0.18Å	-	-	-
A 619		LEU	0.69	-	Favored (52.48%) General / -69.9,144.5	Favored (35.9%) *mt* chi angles: 284.1,163	0.05Å	-	-	-
A 620		ASP	0.7	-	Favored (6.06%) General / -97.9,25.4	Favored (53.5%) *m-30* chi angles: 295.1,300.1	0.08Å	-	-	-
A 621		HIS	0.72	-	Favored (50.25%) General / -125.8,129.8	Favored (61.2%) *m90* chi angles: 303.1,82.6	0.08Å	-	-	-
A 622		ASP	0.77	-	Favored (32.96%) General / -71.2,160.4	Favored (93.8%) *m-30* chi angles: 288.4,340.5	0.06Å	-	-	-
A 623		HIS	0.72	-	Favored (80.86%) General / -60.3,-37.5	Favored (42.5%) *p90* chi angles: 57.8,82.4	0.24Å	-	OUTLIER(S) worst is ND1-CG-CD2: 4.6 σ	-
A 624		ASN	0.76	-	Favored (85.71%) General / -59.7,-39.7	Favored (58.7%) *m-40* chi angles: 295.3,353.2	0.09Å	-	-	-
A		GLN	0.75	-	Favored (79.29%)	Favored (66.1%) *mt0*	0.03Å	-	-	-

#	Alt	Res	High B	Clash > 0.4Å	Ramachandran	Rotamer	Cβ deviation	Bond lengths	Bond angles	Cis Peptides
625					General / -69.0,-39.7	chi angles: 301.2,176.8,292.9				
A 626		ILE	0.79	-	Favored (55.3%) Ile or Val / -55.8,-39.2	Favored (58.4%) *mt* chi angles: 286.9,169.7	0.05Å	-	-	-
A 627		PHE	0.78	-	Favored (22.67%) General / -52.7,-55.4	Favored (46.3%) *t80* chi angles: 175.9,273.1	0.04Å	-	-	-
A 628		TYR	0.78	-	Favored (93.11%) General / -64.8,-39.1	Favored (42.3%) *m-80* chi angles: 291.5,120.8	0.05Å	-	-	-
A 629		LEU	0.78	-	Favored (86.41%) General / -63.9,-46.1	Favored (20.2%) *tp* chi angles: 188.4,54.6	0.04Å	-	-	-
A 630		GLY	0.8	-	Favored (92.12%) Glycine / -63.2,-35.5	-	-	-	-	-
A 631		TYR	0.78	-	Favored (78.4%) General / -59.3,-49.3	Favored (32.4%) *t80* chi angles: 163.6,71.5	0.08Å	-	-	-
A 632		ALA	0.78	-	Favored (85.82%) General / -64.3,-37.0	-	0.06Å	-	-	-
A 633		GLN	0.73	-	Favored (62.18%) General / -71.8,-15.4	Favored (69.6%) *mt0* chi angles: 292.8,189.6,340.1	0.13Å	-	-	-
A 634		LEU	0.75	0.63Å HD11 with A 514 THR HA	Favored (85.77%) General / -64.0,-37.0	Favored (75.8%) *mt* chi angles: 300.7,173.7	0.16Å	-	-	-
**#**	**Alt**	**Res**	**High B**	**Clash > 0.4Å**	**Ramachandran**	**Rotamer**	**Cβ deviation**	**Bond lengths**	**Bond angles**	**Cis Peptides**
			Avg: 0.70	Clashscore: 10.3	Outliers: 12 of 650	Poor rotamers: 11 of 577	Outliers: 10 of 629	Outliers: 1 of 652	Outliers: 75 of 652	Non-Trans: 1 of 651
A 635		TYR	0.74	-	Favored (44.18%) General / -94.4,7.8	Favored (73.2%) *m-80* chi angles: 288.1,98.7	0.04Å	-	-	-
A 636		CYS	0.79	-	Favored (6.8%) General / -58.7,119.3	Favored (80.2%) *m* chi angles: 296.1	0.10Å	-	-	-
A 637		THR	0.76	0.47Å HG21 with A 651 GLU HB2	Favored (30.48%) General / -155.4,153.5	Favored (11.8%) *t* chi angles: 188	0.03Å	-	OUTLIER(S) worst is CA-CB-OG1: 4.3 σ	-

A 638		ASP	0.71	-	Favored (45.4%) General / -132.5,157.2	Favored (18.6%) *m-30* chi angles: 286,294.1	0.14Å	-	OUTLIER(S) worst is CA-CB-CG: 4.8 σ	-
A 639		TYR	0.65	-	Favored (31.45%) Pre-Pro / -156.7,159.3	Allowed (1%) *p90* chi angles: 43.9,61.1	0.15Å	-	-	-
A 640		PRO	0.71	0.42Å HG2 with A 79 THR HB	Favored (80.35%) Trans-Pro / -60.5,150.6	Favored (9.8%) *Cg_exo* chi angles: 347.1,28.8,326.4	0.08Å	-	-	-
A 641		GLU	0.69	-	Favored (72.78%) General / -60.6,-33.5	Favored (12.8%) *tt0* chi angles: 196.1,193.5,309	0.06Å	-	-	-
A 642		THR	0.68	-	Favored (73.12%) General / -66.6,-47.3	Favored (57.1%) *p* chi angles: 57.1	0.06Å	-	-	-
A 643		VAL	0.68	-	Favored (82.11%) Ile or Val / -59.7,-40.4	Favored (14.1%) *t* chi angles: 163.2	0.04Å	-	-	-
A 644		GLU	0.68	-	Favored (81.85%) General / -59.0,-39.4	Favored (42.1%) *tt0* chi angles: 192,174.4,27.5	0.05Å	-	-	-
A 645		ARG	0.59	-	Favored (58.81%) General / -76.1,-9.7	Favored (62.4%) *mtt90* chi angles: 296.7,176.9,179.3,105.7	0.14Å	-	-	-
A 646		PHE	0.59	-	Favored (26.64%) General / -105.7,0.8	Favored (23.6%) *m-10* chi angles: 295.2,330.4	0.04Å	-	-	-
A 647		ASP	0.5	-	Favored (25.56%) General / -153.2,147.3	Favored (38.9%) *t0* chi angles: 192.5,354.8	0.04Å	-	-	-
A 648		GLU	0.44	-	Favored (25.39%) General / -106.1,0.4	Favored (38.1%) *mp0* chi angles: 292.5,75.1,29.5	0.07Å	-	-	-
A 649		LEU	0.55	0.46Å HB2 with A 579 LEU HD22	OUTLIER (0.08)%) Pre-Pro / -97.7,76.8	Favored (17%) *tp* chi angles: 190.6,55.2	0.04Å	-	OUTLIER(S) worst is CA-C-N: 5.7 σ	-
A 650		PRO	0.7	-	Favored (17.65%) Trans-Pro / -77.4,138.4	Favored (23.8%) *Cg_endo* chi angles: 36.3,321.1,27.5	0.03Å	-	-	-
A 651		GLU	0.71	0.47Å HB2 with A 637 THR	Favored (5.85%) General /	Favored (3.3%) *mp0* chi angles: 295.6,84,303.4	0.12Å	-	-	-

#	Alt	Res	High B	Clash > 0.4Å	Ramachandran	Rotamer	Cβ deviation	Bond lengths	Bond angles	Cis Peptides
				HG21	-46.0,-36.7					
A 652		GLN	0.71	-	Favored (83.45%) General / -58.4,-40.8	Favored (16%) *pt0* chi angles: 77,181.2,318.1	0.14Å	-	-	-
A 653		LEU	0.71	-	Favored (41.8%) General / -79.4,-27.0	Favored (59.7%) *mt* chi angles: 291.1,164.3	0.14Å	-	-	-
A 654		ARG	0.69	-	Favored (86.22%) General / -58.4,-42.0	Favored (34.4%) *mtp180* chi angles: 302.4,182.9,78.4,162.1	0.06Å	-	-	-
**#**	**Alt**	**Res**	**High B**	**Clash > 0.4Å**	**Ramachandran**	**Rotamer**	**Cβ deviation**	**Bond lengths**	**Bond angles**	**Cis Peptides**
			Avg: 0.70	Clashscore: 10.3	Outliers: 12 of 650	Poor rotamers: 11 of 577	Outliers: 10 of 629	Outliers: 1 of 652	Outliers: 75 of 652	Non-Trans: 1 of 651
A 655		VAL	0.77	-	Favored (3.25%) Ile or Val / -62.5,-60.1	Favored (58.8%) *t* chi angles: 170.6	0.06Å	-	-	-
A 656		ASN	0.71	-	Favored (97.21%) General / -64.1,-41.6	Favored (92.7%) *m-40* chi angles: 288,331.3	0.04Å	-	-	-
A 657		THR	0.71	-	Favored (70.43%) General / -69.8,-44.2	Favored (44.7%) *p* chi angles: 66.7	0.06Å	-	-	-
A 658		ALA	0.76	-	Favored (83.09%) General / -59.3,-48.0	-	0.02Å	-	-	-
A 659		LEU	0.76	-	Favored (87.71%) General / -66.2,-38.3	Favored (87.4%) *mt* chi angles: 299.3,175.5	0.10Å	-	-	-
A 660		SER	0.77	-	Favored (65.15%) General / -66.5,-18.1	Favored (19.3%) *m* chi angles: 287.2	0.10Å	-	-	-
A 661		ASN	0.74	0.74Å ND2 with A 683 CYS H	Favored (45.07%) General / -93.5,-7.1	Favored (31.4%) *m110* chi angles: 292.7,122.4	0.01Å	-	OUTLIER(S) worst is CA-CB-CG: 6.4 σ	-
A 662		SER	0.78	-	Favored (25.84%) General / -93.6,113.7	Favored (36.3%) *t* chi angles: 174.5	0.05Å	-	-	-
A 663		GLN	0.74	-	Favored (88.33%) General / -61.5,-38.7	Favored (84.2%) *tp40* chi angles: 182.7,64.4,44.7	0.05Å	-	-	-

A 664		GLN	0.76	-	Favored (90.92%) General / -63.7,-38.4	Favored (97.9%) *mm-40* chi angles: 296.3,297.4,308.7	0.09Å	-	-	-
A 665		PHE	0.78	-	Favored (77.03%) General / -59.2,-49.6	Favored (68.6%) *t80* chi angles: 170,259.9	0.04Å	-	-	-
A 666		ALA	0.8	-	Favored (90.68%) General / -64.4,-38.3	-	0.10Å	-	-	-
A 667		ASN	0.79	-	Favored (94.35%) General / -61.1,-45.5	Favored (34.3%) *m-40* chi angles: 291,5.7	0.10Å	-	-	-
A 668		ALA	0.8	-	Favored (50.46%) General / -78.8,-20.2	-	0.03Å	-	-	-
A 669		TYR	0.78	-	Favored (29.69%) General / -106.4,5.6	Favored (22.3%) *m-80* chi angles: 275.1,96.2	0.06Å	-	-	-
A 670		GLY	0.82	-	Favored (52.37%) Glycine / 51.3,45.6	-	-	-	-	-
A 671		CYS	0.82	-	Favored (21.35%) General / -71.8,122.8	Favored (54.1%) *m* chi angles: 302.6	0.08Å	-	-	-
A 672		SER	0.79	-	Favored (8.59%) General / -57.1,155.4	Favored (53.2%) *m* chi angles: 292.1	0.16Å	-	OUTLIER(S) worst is C-N-CA: 4.9 σ	-
A 673		ARG	0.71	-	OUTLIER (0)%) General / -34.5,-21.7	Favored (52.3%) *ptt-90* chi angles: 65.5,186,181.8,274.9	0.05Å	-	-	-
A 674		GLU	0.7	-	Allowed (0.27%) General / -157.7,30.2	Favored (7%) *pt0* chi angles: 59.5,194.4,50.1	0.10Å	-	-	-
**#**	**Alt**	**Res**	**High B**	**Clash > 0.4Å**	**Ramachandran**	**Rotamer**	**Cβ deviation**	**Bond lengths**	**Bond angles**	**Cis Peptides**
			Avg: 0.70	Clashscore: 10.3	Outliers: 12 of 650	Poor rotamers: 11 of 577	Outliers: 10 of 629	Outliers: 1 of 652	Outliers: 75 of 652	Non-Trans: 1 of 651
A 675		GLU	0.71	-	Favored (4.57%) General / -56.1,157.5	Favored (73.3%) *mm-30* chi angles: 300.5,294.5,321.1	0.10Å	-	-	-
					Favored	Favored (73.5%)				

A 676		LYS	0.69	-	(43.87%) General / -51.8,-35.2	*mttt* chi angles: 287.8,174.4,180.3,161.1	0.08Å	-	-	-
A 677		LEU	0.72	0.40Å HD11 with A 60 PHE N	Favored (25.24%) General / -97.0,14.5	Favored (74.7%) *mt* chi angles: 301.9,180.9	0.15Å	-	-	-
A 678		ASN	0.74	-	Allowed (1.9%) General / -136.2,54.4	Favored (37.3%) *t0* chi angles: 196.3,14.7	0.09Å	-	-	-
A 679		ALA	0.69	-	Favored (49.87%) General / -55.2,134.6	-	0.04Å	-	-	-
A 680		ARG	0.65	-	Favored (83.2%) General / -68.0,-40.6	Favored (91.7%) *mtt-85* chi angles: 295.6,178.7,192.5,278.4	0.03Å	-	-	-
A 681		PHE	0.64	-	Favored (2.64%) General / -75.7,81.1	Favored (96.2%) *m-80* chi angles: 293.3,96.2	0.05Å	-	-	-
A 682		LYS	0.69	-	Favored (34.43%) General / -90.3,134.3	Favored (28.4%) *mttp* chi angles: 278.4,170.6,168.4,70.7	0.04Å	-	-	-
A 683		CYS	0.78	0.74Å H with A 661 ASN ND2	Favored (50.13%) General / -103.1,128.0	Favored (38.4%) *m* chi angles: 305	0.03Å	-	-	-
A 684		THR	0.76	-	Favored (43.81%) General / -132.8,133.8	Favored (33%) *m* chi angles: 292.9	0.12Å	-	OUTLIER(S) worst is OG1-CB-CG2: 5.2 σ	-
A 685		LEU	0.77	0.47Å HD21 with A 552 TYR CZ	Allowed (0.61%) General / -132.9,-68.2	Allowed (1.1%) *tp* chi angles: 179.6,35.9	0.07Å	-	-	-
A 686		TYR	0.78	0.43Å CZ with A 527 PHE HB3	-	Favored (67.5%) *m-80* chi angles: 288.6,105.2	0.08Å	-	-	-

Figure S4

```
### targetp v1.1 prediction results ##################################
Number of query sequences:  12
Cleavage site predictions not included.
Using NON-PLANT networks.

Name                  Len            mTP     SP  other  Loc  RC
----------------------------------------------------------------------
DmelNepl15            686          0.025  0.953  0.067   S    1
DanaGF18283           691          0.026  0.921  0.078   S    1
DereGG12480           686          0.022  0.964  0.060   S    1
DgriGH18312           682          0.022  0.963  0.048   S    1
DmojGI100776          685          0.032  0.938  0.067   S    1
DperCG23771           685          0.065  0.943  0.021   S    1
DpseGA18384           687          0.030  0.942  0.050   S    1
DsecGM23611           686          0.020  0.964  0.066   S    1
DsimGD18421           686          0.023  0.958  0.064   S    1
DvirGJ14375           685          0.029  0.926  0.079   S    1
DwilGK11619           688          0.152  0.806  0.050   S    2
DyakGE24002           686          0.029  0.951  0.064   S    1
----------------------------------------------------------------------
cutoff                             0.000  0.000  0.000
```

Figure S5

A *D. simulans*

```
# DsimGD18421 Length: 686
# DsimGD18421 Number of predicted TMHs:  0
# DsimGD18421 Exp number of AAs in TMHs: 8.73464
# DsimGD18421 Exp number, first 60 AAs:  8.73063
# DsimGD18421 Total prob of N-in:        0.39619
DsimGD18421     TMHMM2.0        outside      1   686
```

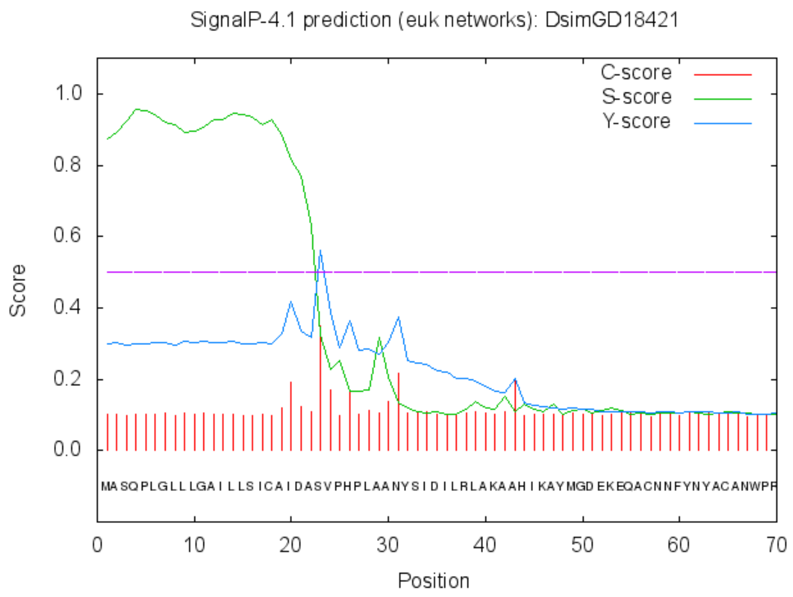

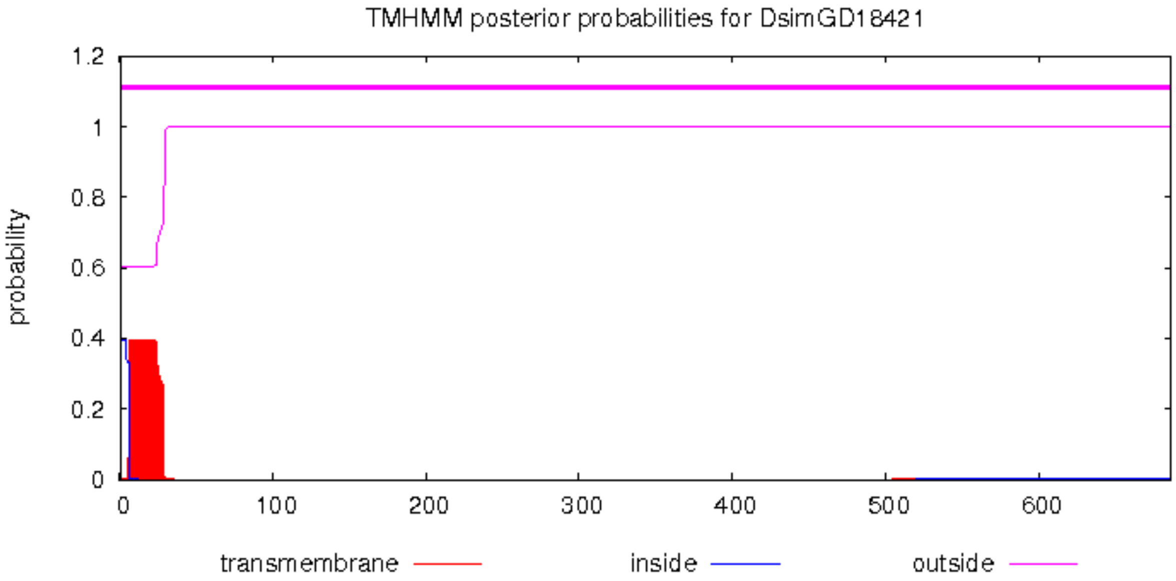


```
# Measure  Position  Value    Cutoff   signal peptide?
  max. C     23      0.351
  max. Y     23      0.560
  max. S      4      0.955
  mean S      1-22   0.895
       D      1-22   0.741   0.450   YES
Name=DsimGD18421        SP='YES' Cleavage site between pos. 22 and 23: IDA-SV D=0.741 D-cutoff=0.450 Networks=SignalP-noTM
```

B *D. melanogaster*

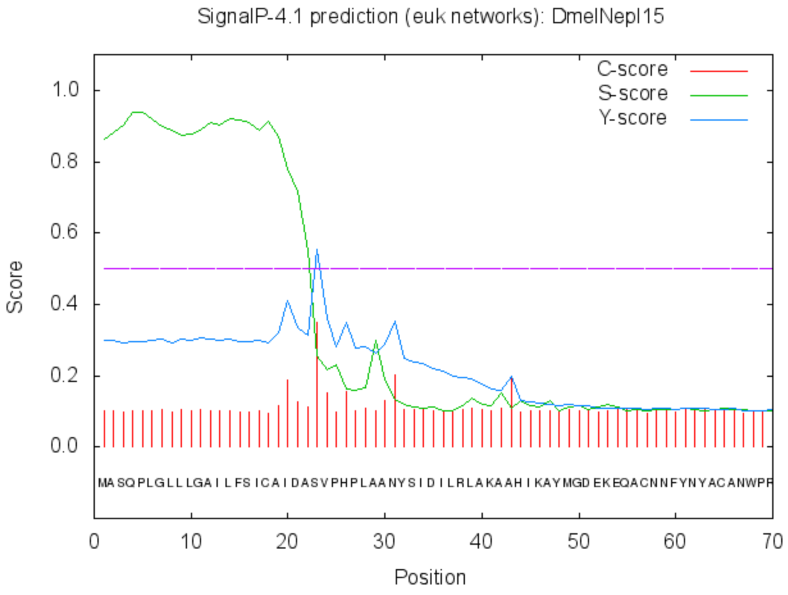


```
# DmelNepl15 Length: 686
# DmelNepl15 Number of predicted TMHs:  0
# DmelNepl15 Exp number of AAs in TMHs: 10.01779
# DmelNepl15 Exp number, first 60 AAs:  10.01377
# DmelNepl15 Total prob of N-in:        0.45443
# DmelNepl15 POSSIBLE N-term signal sequence
DmelNepl15      TMHMM2.0        outside      1   686
```

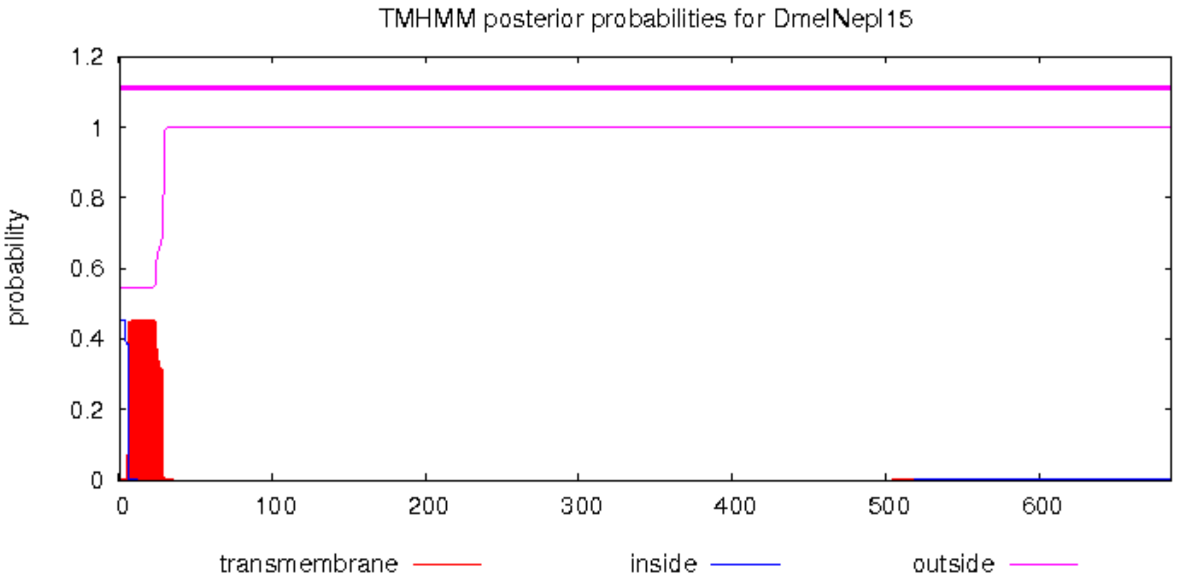


```
# Measure  Position  Value    Cutoff   signal peptide?
  max. C    23       0.351
  max. Y    23       0.553
  max. S     4       0.939
  mean S     1-22    0.871
       D     1-22    0.724   0.450   YES
Name=DmelNepl15 SP='YES' Cleavage site between pos. 22 and 23: IDA-SV D=0.724 D-cutoff=0.450 Networks=SignalP-noTM
```

C *D. sechellia*

```
# DsecGM23611 Length: 686
# DsecGM23611 Number of predicted TMHs:  1
# DsecGM23611 Exp number of AAs in TMHs: 17.06833
# DsecGM23611 Exp number, first 60 AAs:  17.06431
# DsecGM23611 Total prob of N-in:        0.77093
# DsecGM23611 POSSIBLE N-term signal sequence
DsecGM23611     TMHMM2.0        inside       1      6
DsecGM23611     TMHMM2.0        TMhelix      7     29
DsecGM23611     TMHMM2.0        outside     30    686
```

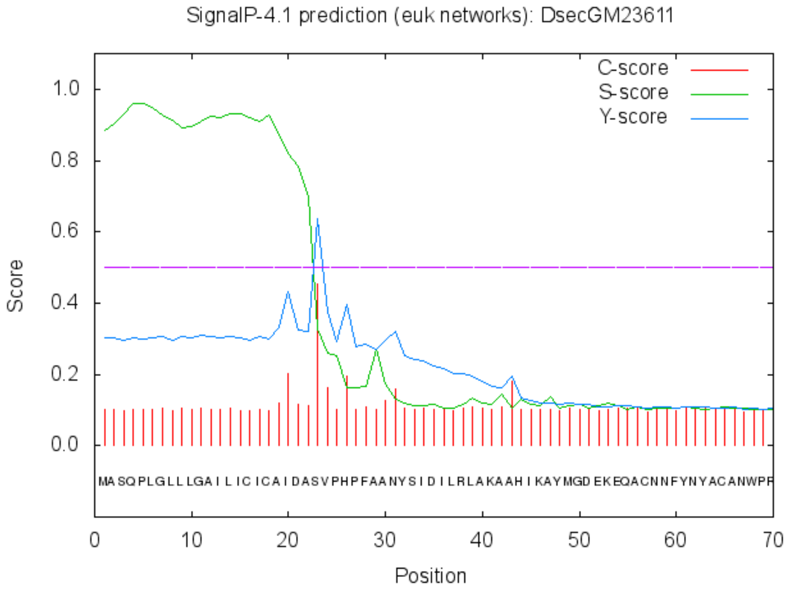

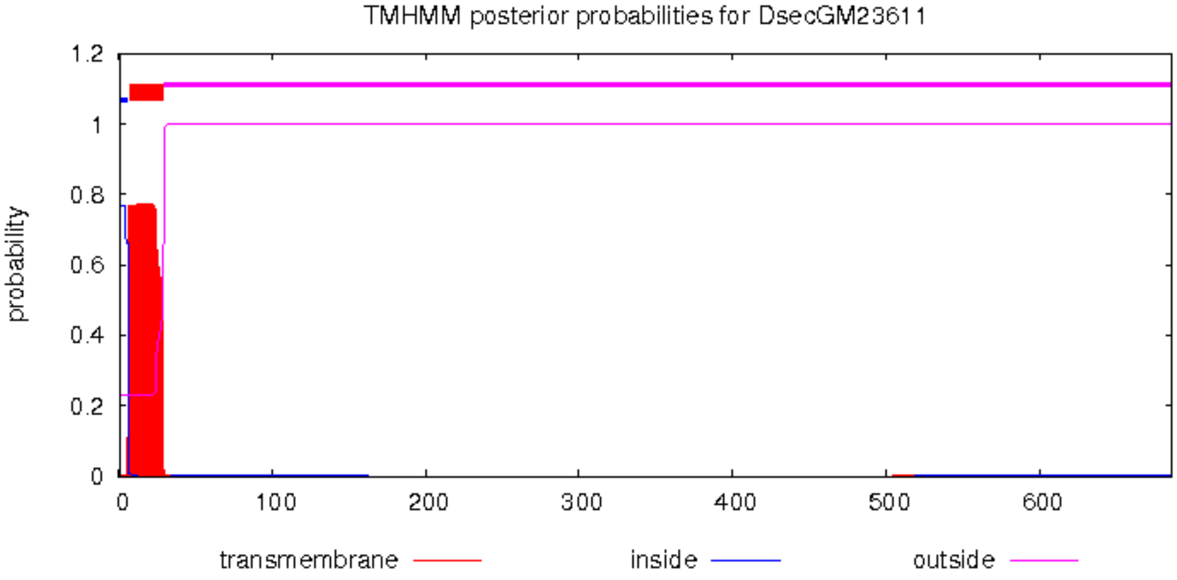


```
# Measure  Position  Value    Cutoff   signal peptide?
  max. C     23      0.453
  max. Y     23      0.638
  max. S      4      0.961
  mean S      1-22   0.899
       D      1-22   0.779   0.450   YES
Name=DsecGM23611        SP='YES' Cleavage site between pos. 22 and 23: IDA-SV D=0.779 D-cutoff=0.450 Networks=SignalP-noTM
```

D *D. yakuba*

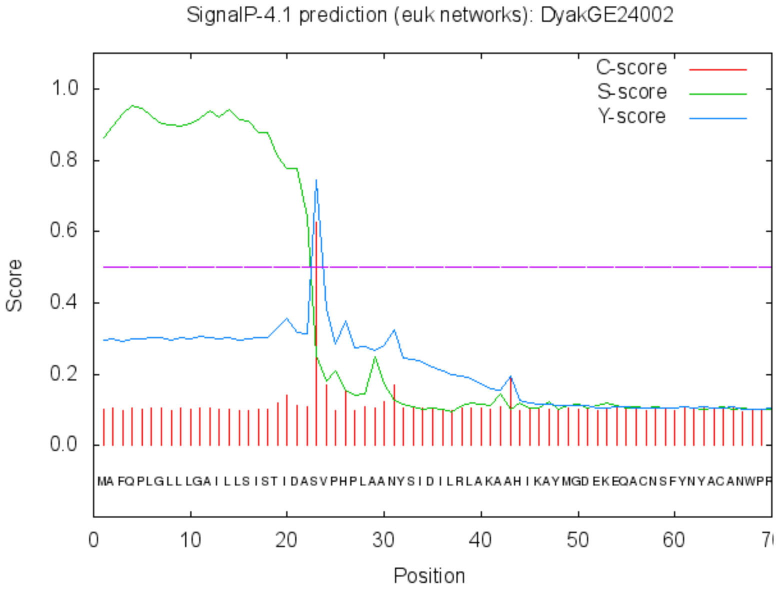


```
# DyakGE24002 Length: 686
# DyakGE24002 Number of predicted TMHs:  0
# DyakGE24002 Exp number of AAs in TMHs: 2.07931
# DyakGE24002 Exp number, first 60 AAs:  2.07366
# DyakGE24002 Total prob of N-in:        0.09455
DyakGE24002     TMHMM2.0        outside      1   686
```

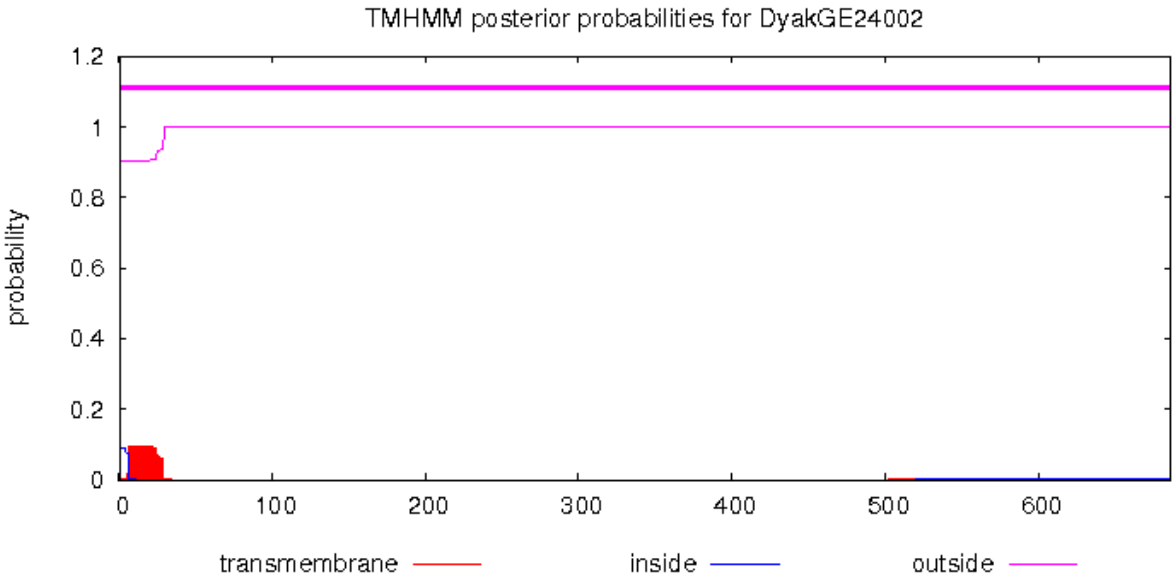


```
# Measure  Position  Value    Cutoff   signal peptide?
  max. C    23       0.626
  max. Y    23       0.743
  max. S     4       0.953
  mean S     1-22    0.882
       D     1-22    0.818   0.450   YES
Name=DyakGE24002       SP='YES' Cleavage site between pos. 22 and 23: IDA-SV D=0.818 D-cutoff=0.450 Networks=SignalP-noTM
```

E *D. erecta*

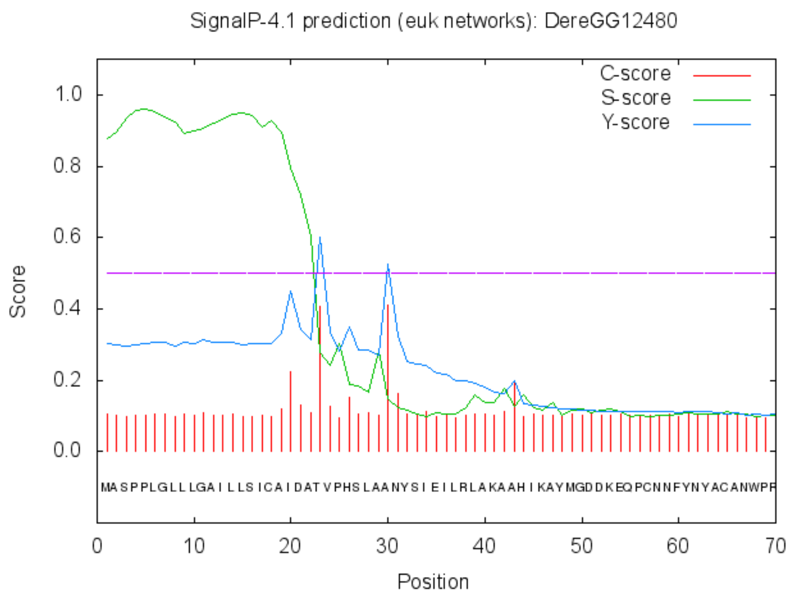


```
# DereGG12480 Length: 686
# DereGG12480 Number of predicted TMHs:  0
# DereGG12480 Exp number of AAs in TMHs: 7.82669
# DereGG12480 Exp number, first 60 AAs:  7.8224
# DereGG12480 Total prob of N-in:        0.35328
DereGG12480     TMHMM2.0        outside      1   686
```

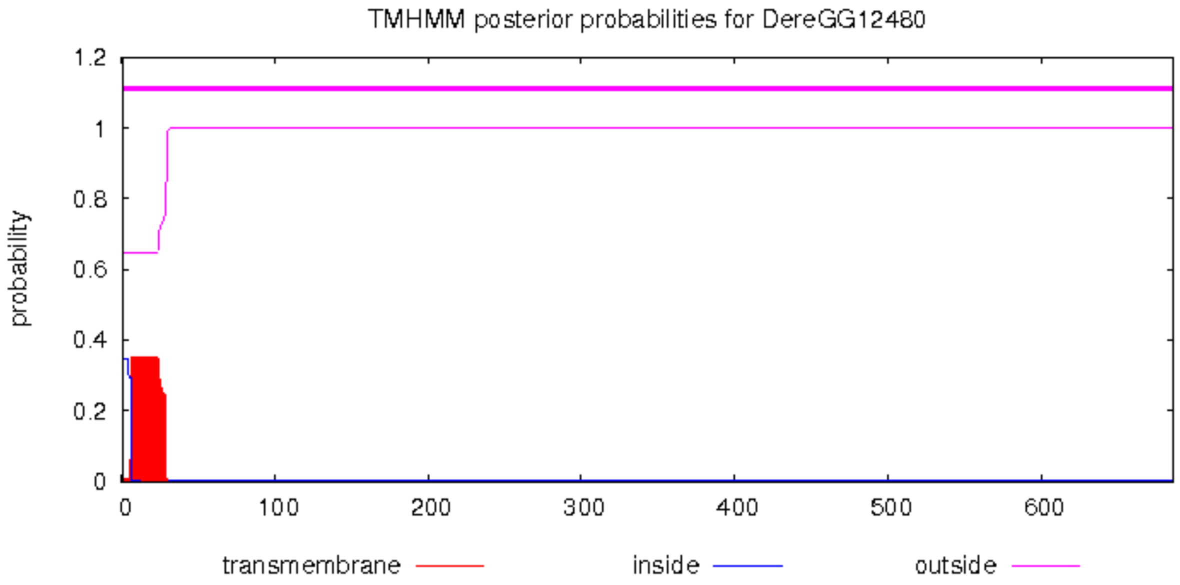


```
# Measure  Position  Value    Cutoff   signal peptide?
  max. C    30       0.410
  max. Y    23       0.602
  max. S     5       0.961
  mean S     1-22    0.894
       D     1-22    0.760   0.450   YES
Name=DereGG12480        SP='YES' Cleavage site between pos. 22 and 23: IDA-TV D=0.760 D-cutoff=0.450 Networks=SignalP-noTM
```

F *D. ananassae*

```
# DanaGF18283 Length: 691
# DanaGF18283 Number of predicted TMHs:  0
# DanaGF18283 Exp number of AAs in TMHs: 1.62112
# DanaGF18283 Exp number, first 60 AAs:  1.60732
# DanaGF18283 Total prob of N-in:        0.07534
DanaGF18283     TMHMM2.0        outside      1   691
```

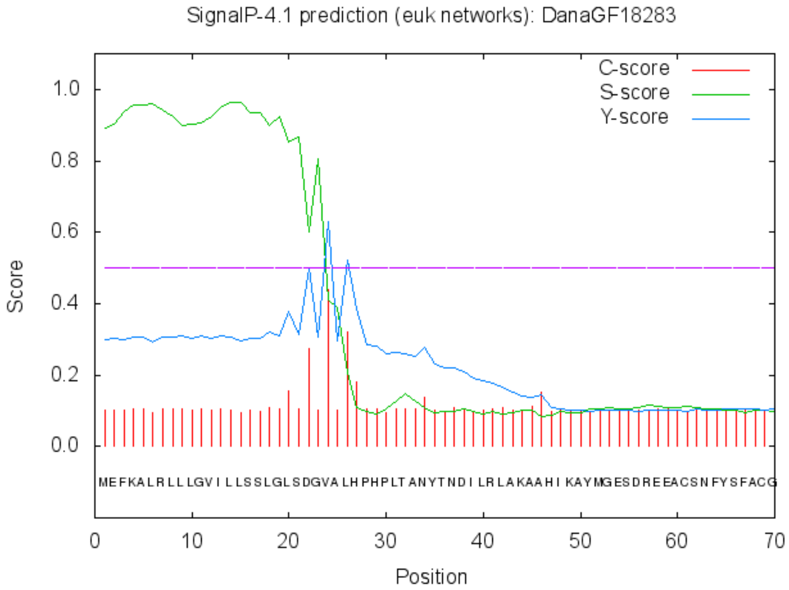

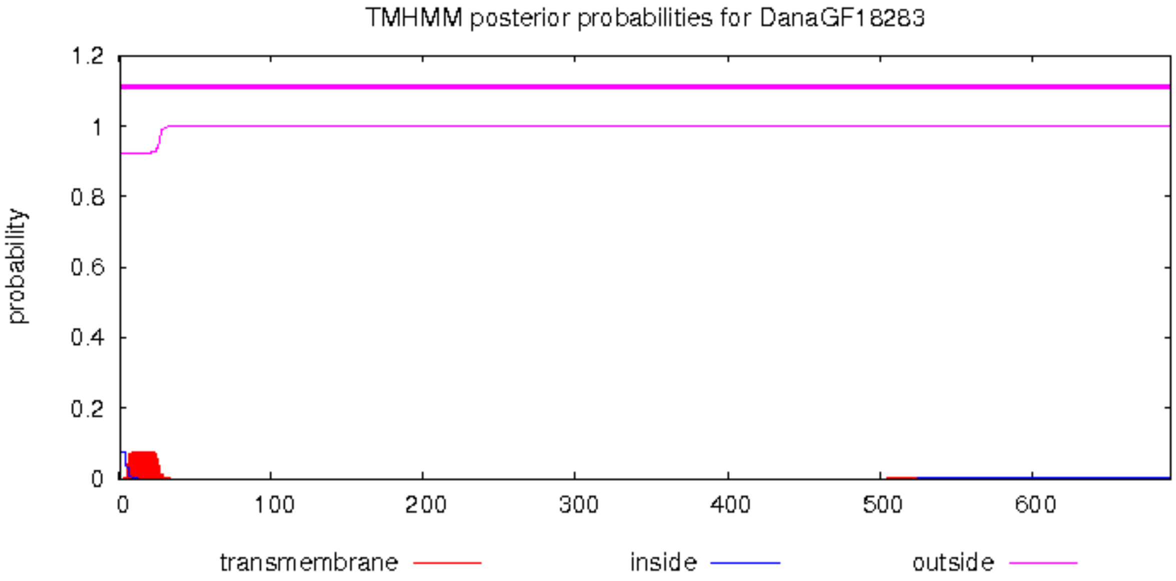


```
# Measure  Position  Value    Cutoff   signal peptide?
  max. C    24       0.440
  max. Y    24       0.631
  max. S    14       0.963
  mean S     1-23    0.904
       D     1-23    0.778   0.450   YES
Name=DanaGF18283       SP='YES' Cleavage site between pos. 23 and 24: SDG-VA D=0.778 D-cutoff=0.450 Networks=SignalP-noTM
```

G *D. pseudoobscura*

```
# DpseGA18384 Length: 687
# DpseGA18384 Number of predicted TMHs:  1
# DpseGA18384 Exp number of AAs in TMHs: 16.95645
# DpseGA18384 Exp number, first 60 AAs:  16.94895
# DpseGA18384 Total prob of N-in:        0.77556
# DpseGA18384 POSSIBLE N-term signal sequence
DpseGA18384     TMHMM2.0        inside       1     4
DpseGA18384     TMHMM2.0        TMhelix      5    27
DpseGA18384     TMHMM2.0        outside     28   687
```

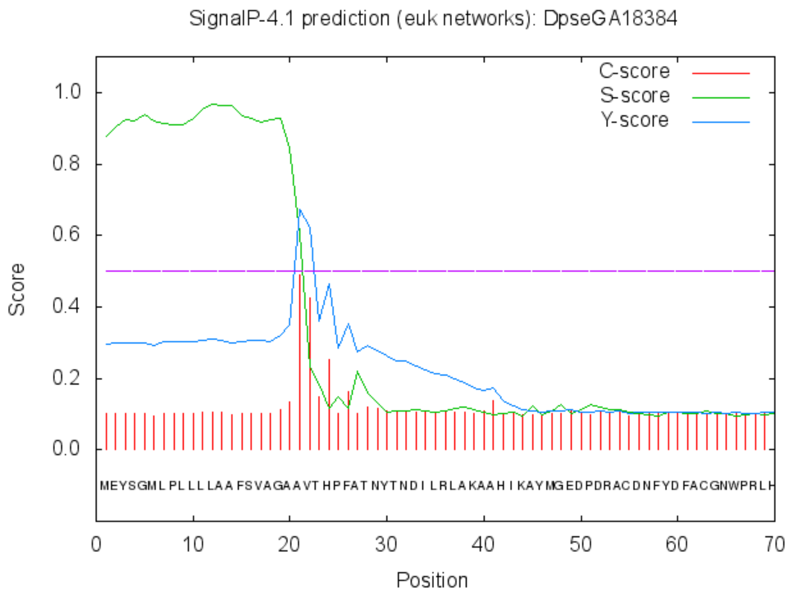

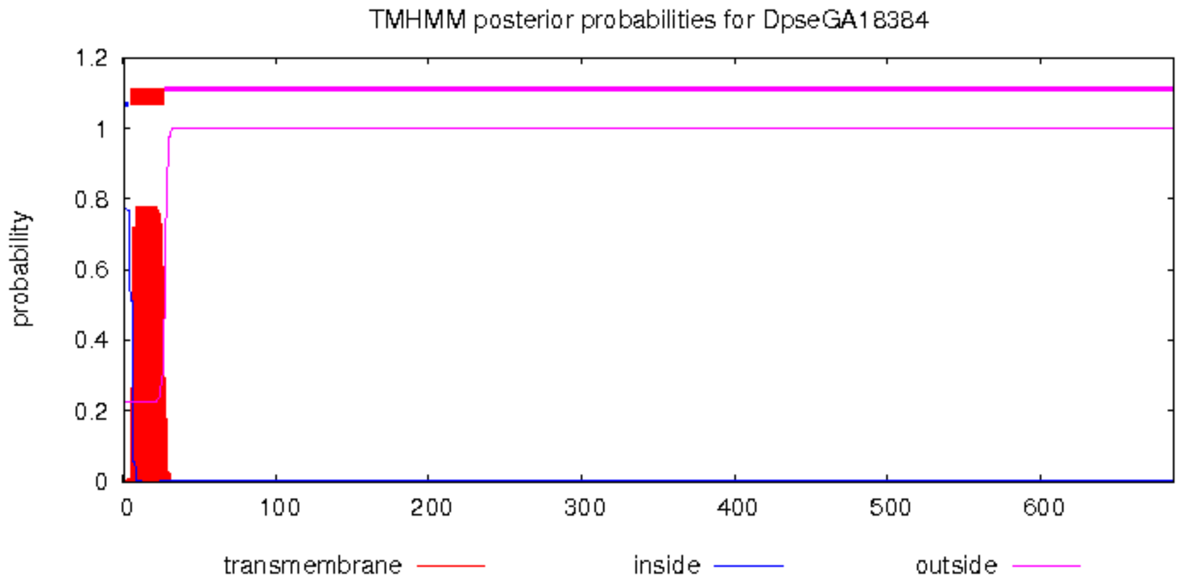


```
# Measure  Position  Value    Cutoff   signal peptide?
  max. C    21       0.491
  max. Y    21       0.672
  max. S    12       0.966
  mean S     1-20    0.924
       D     1-20    0.808   0.450    YES
Name=DpseGA18384        SP='YES' Cleavage site between pos. 20 and 21: AGA-AV D=0.808 D-cutoff=0.450 Networks=SignalP-noTM
```

H *D. persimilis*

SignalP-4.1 prediction (euk networks): DperCG23771

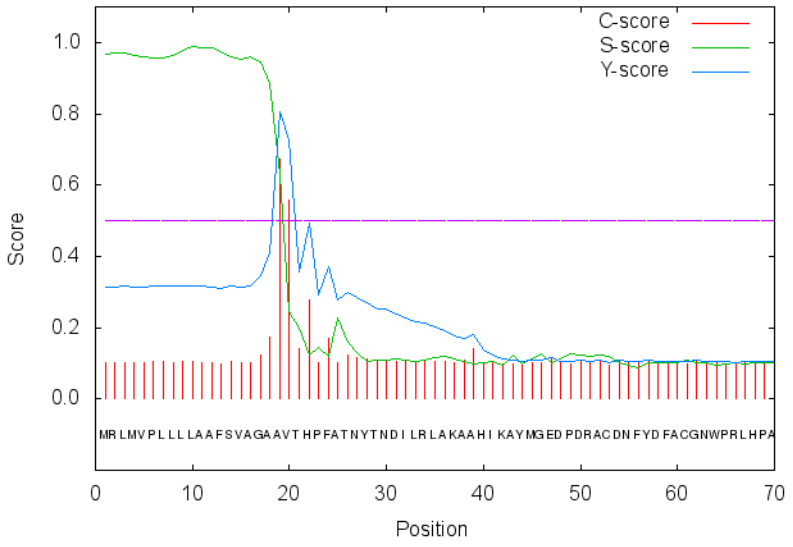


```
# Measure  Position  Value    Cutoff   signal peptide?
  max. C    19       0.672
  max. Y    19       0.805
  max. S    10       0.987
  mean S     1-18    0.962
       D     1-18    0.890   0.450    YES
Name=DperCG23771        SP='YES' Cleavage site between pos. 18 and 19: AGA-AV D=0.890 D-cutoff=0.450 Networks=SignalP-noTM
```

```
# DperCG23771 Length: 685
# DperCG23771 Number of predicted TMHs:  0
# DperCG23771 Exp number of AAs in TMHs: 14.87673
# DperCG23771 Exp number, first 60 AAs:  14.87032
# DperCG23771 Total prob of N-in:        0.70823
# DperCG23771 POSSIBLE N-term signal sequence
DperCG23771     TMHMM2.0        outside      1   685
```

TMHMM posterior probabilities for DperCG23771

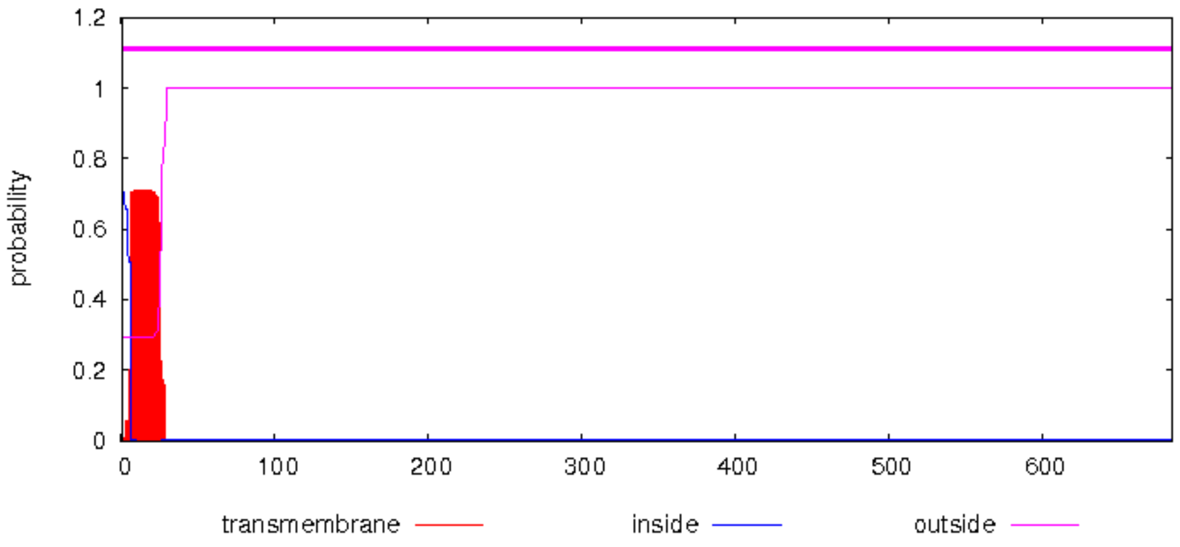

I *D. virilis*

```
# DvirGJ14375 Length: 685
# DvirGJ14375 Number of predicted TMHs:  0
# DvirGJ14375 Exp number of AAs in TMHs: 4.76205000000000001
# DvirGJ14375 Exp number, first 60 AAs:  4.74994
# DvirGJ14375 Total prob of N-in:        0.22688
DvirGJ14375     TMHMM2.0        outside      1   685
```

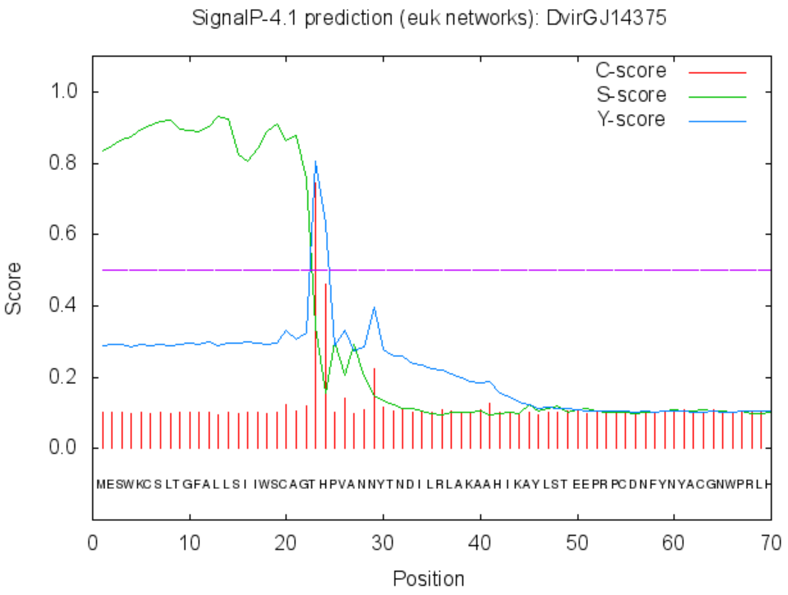

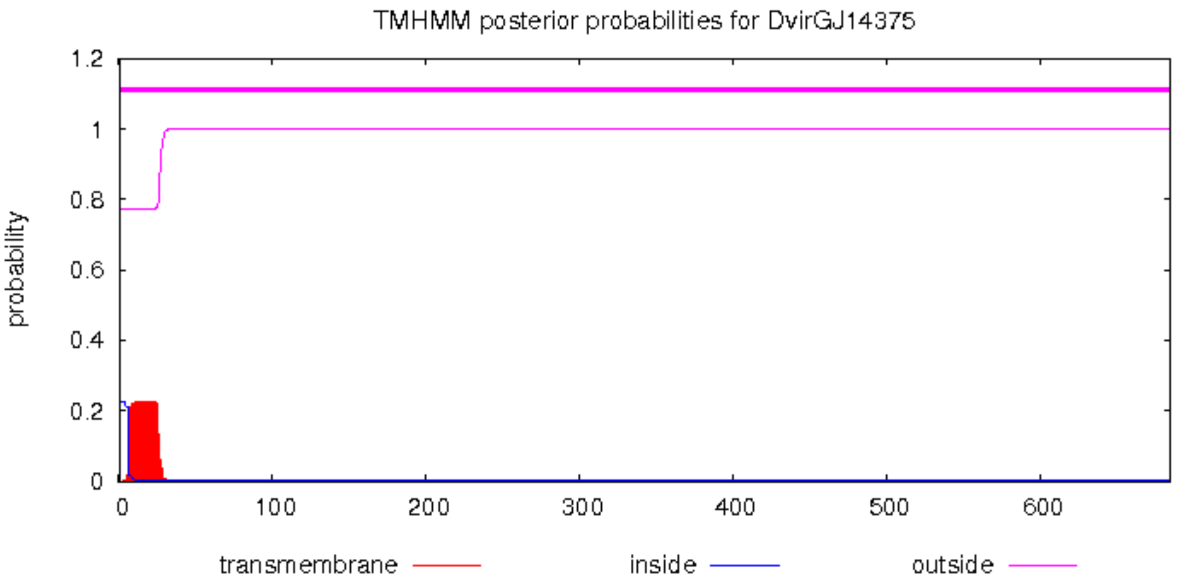


```
# Measure  Position  Value    Cutoff   signal peptide?
  max. C     23       0.743
  max. Y     23       0.806
  max. S     13       0.933
  mean S      1-22    0.875
       D      1-22    0.843   0.450    YES
Name=DvirGJ14375        SP='YES' Cleavage site between pos. 22 and 23: CAG-TH D=0.843 D-cutoff=0.450 Networks=SignalP-noTM
```

J *D. mojavensis*

```
# DmojGI100776 Length: 685
# DmojGI100776 Number of predicted TMHs:  0
# DmojGI100776 Exp number of AAs in TMHs: 14.74749
# DmojGI100776 Exp number, first 60 AAs:  14.73325
# DmojGI100776 Total prob of N-in:        0.70722
# DmojGI100776 POSSIBLE N-term signal sequence
DmojGI100776    TMHMM2.0        outside      1   685
```

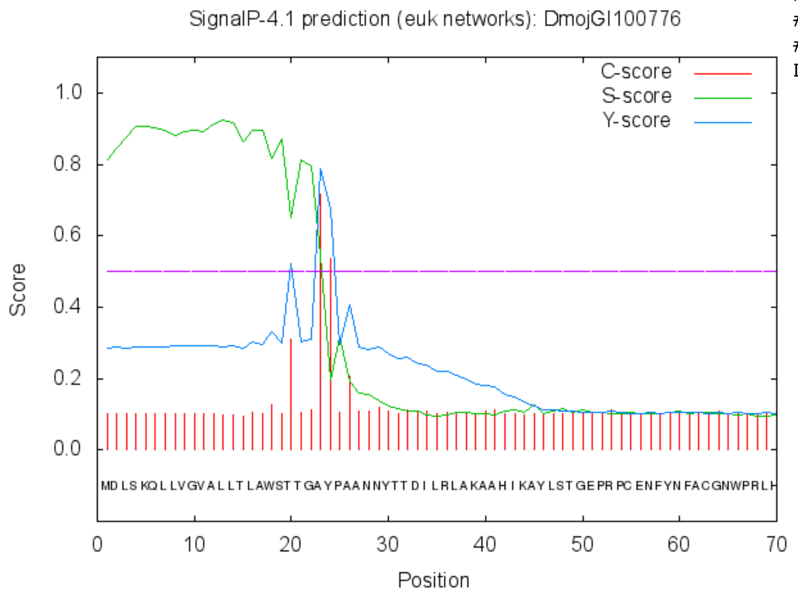

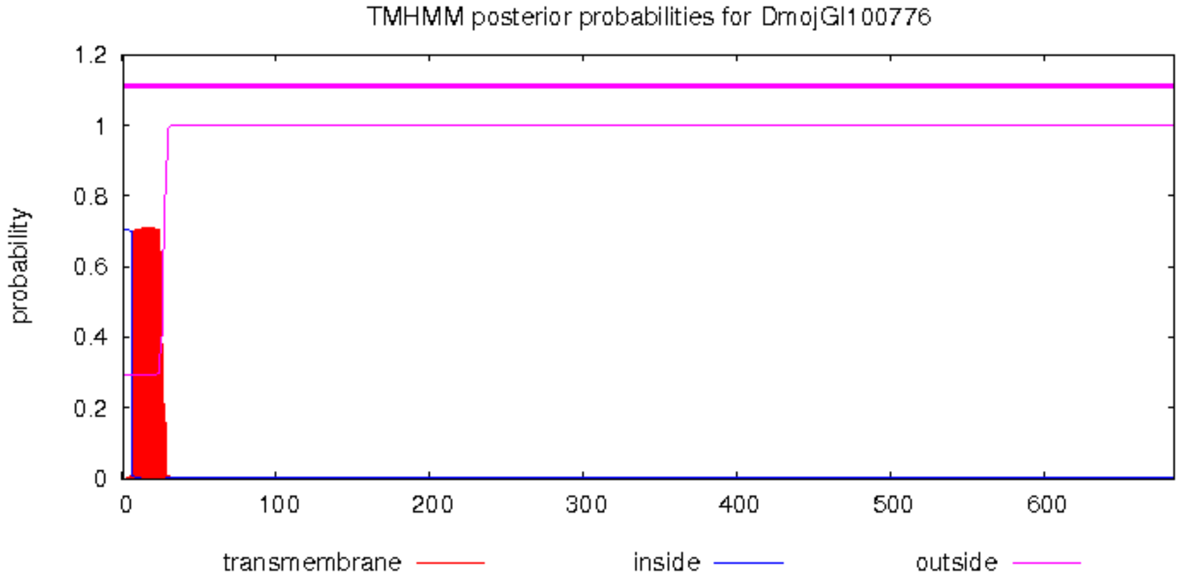


```
# Measure  Position  Value    Cutoff   signal peptide?
  max. C    23       0.715
  max. Y    23       0.787
  max. S    13       0.924
  mean S     1-22    0.866
       D     1-22    0.830   0.450    YES
Name=DmojGI100776       SP='YES' Cleavage site between pos. 22 and 23: TTG-AY D=0.830 D-cutoff=0.450 Networks=SignalP-noTM
```

K *D. grimshawi*

```
# DgriGH18312 Length: 682
# DgriGH18312 Number of predicted TMHs:  0
# DgriGH18312 Exp number of AAs in TMHs: 8.91620999999999998
# DgriGH18312 Exp number, first 60 AAs:  8.90922
# DgriGH18312 Total prob of N-in:        0.41164
DgriGH18312     TMHMM2.0        outside      1   682
```

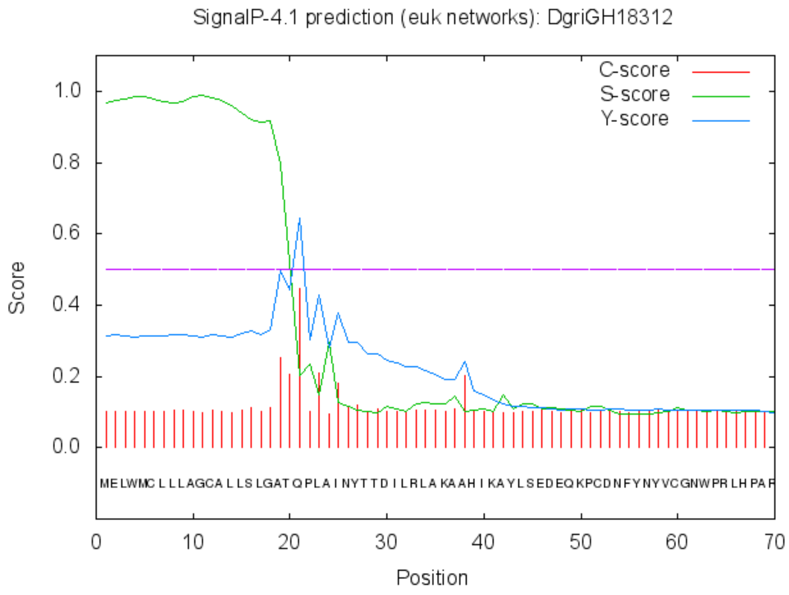

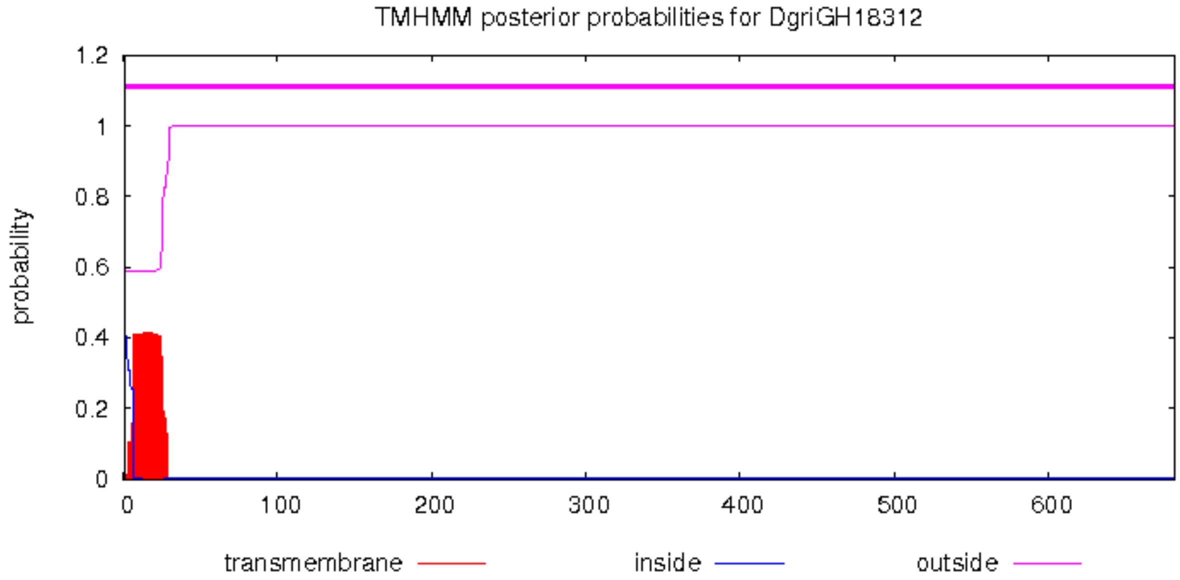


```
# Measure  Position  Value    Cutoff   signal peptide?
  max. C    21       0.445
  max. Y    21       0.645
  max. S    11       0.987
  mean S     1-20    0.934
       D     1-20    0.801   0.450   YES
Name=DgriGH18312       SP='YES' Cleavage site between pos. 20 and 21: GAT-QP D=0.801 D-cutoff=0.450 Networks=SignalP-noTM
```

L *D. willistoni*

```
# DwilGK11619 Length: 688
# DwilGK11619 Number of predicted TMHs:  0
# DwilGK11619 Exp number of AAs in TMHs: 0.05176
# DwilGK11619 Exp number, first 60 AAs:  0.04132
# DwilGK11619 Total prob of N-in:        0.00209
DwilGK11619     TMHMM2.0        outside      1   688
```

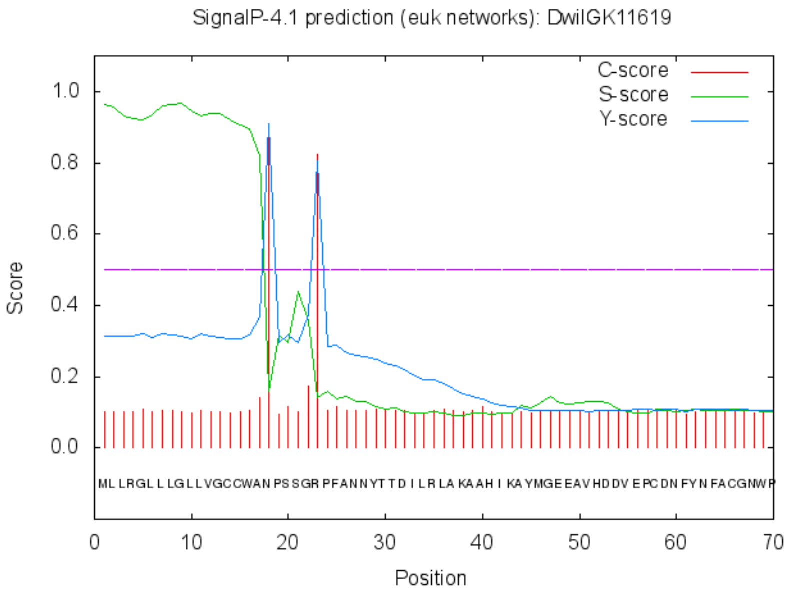

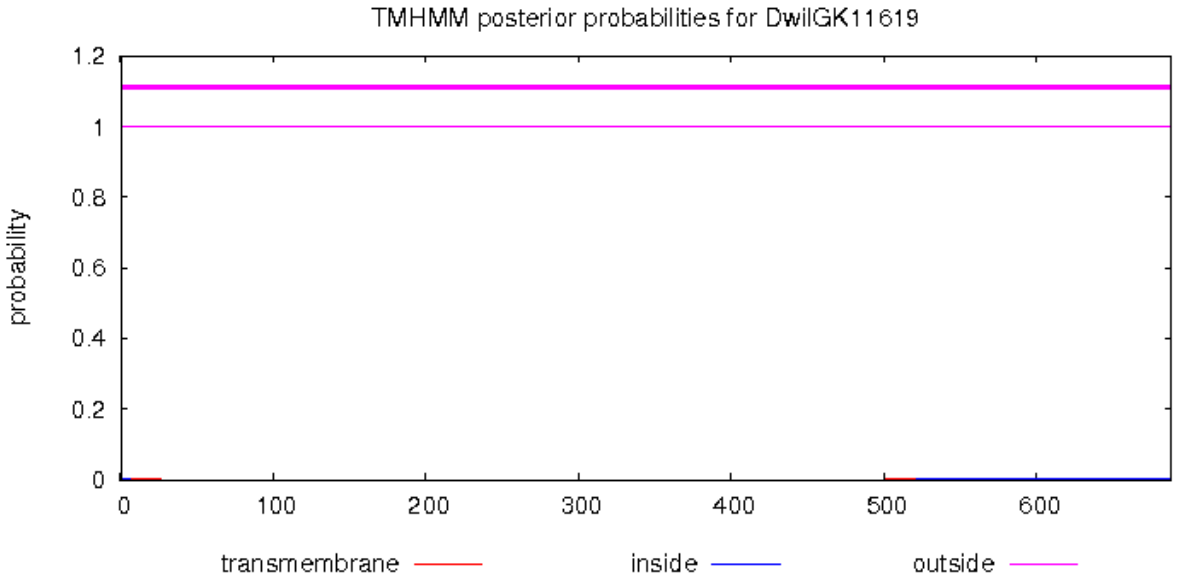


```
# Measure  Position  Value    Cutoff   signal peptide?
  max. C     18       0.884
  max. Y     18       0.911
  max. S      9       0.966
  mean S      1-17    0.930
       D      1-17    0.921   0.450    YES
Name=DwilGK11619        SP='YES' Cleavage site between pos. 17 and 18: CWA-NP D=0.921 D-cutoff=0.450 Networks=SignalP-noTM
```

Figure S6

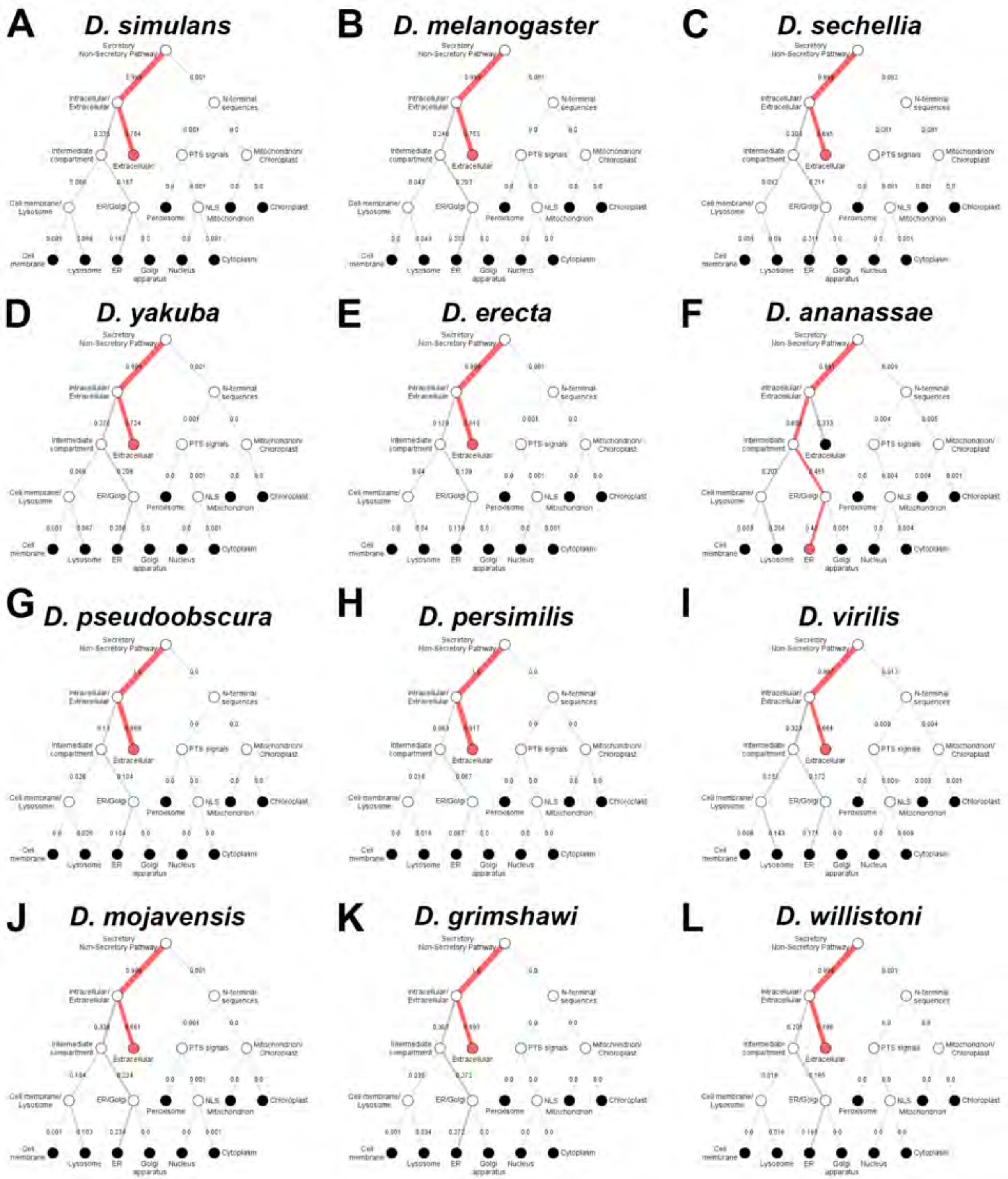
A
D. simulans
B
D. melanogaster
C
D. sechellia
D
D. yakuba
E
D. erecta
F
D. ananassae
G
D. pseudoobscura
H
D. persimilis
I
D. virilis
J
D. mojavensis
K
D. grimshawi
L
D. willistoni

Figure S7

A

Tissue Description	Mean Affy2 Probeset Expression Value	Expression Levels
Larval Central Nervous System	60.175	low expression
Larval Midgut	3.2	no expression
Larval Hindgut	16.4	low expression
Larval Malpighian Tubules	0.9	no expression
Larval Fat Body	193.5	moderate expression
Larval Salivary Gland	3.6	no expression
Larval Trachea	18.2	low expression
Larval Carcass	43.675	low expression
Adult Head	646.8	high expression
Adult Eye	523.7	high expression
Adult Brain	101.9	moderate expression
Adult Thoracic-Abdominal Ganglion	663	high expression
Adult Crop	26.8	low expression
Adult Midgut	1.3	no expression
Adult Hindgut	75.1	low expression
Adult Malpighian Tubules	8.4	no expression
Adult Fat Body	1135.2	very high expression
Adult Salivary Gland	12.2	low expression
Adult Heart	641.525	high expression
Adult Virgin Female Spermatheca	1221.5	very high expression
Adult Inseminated Female Spermatheca	898	high expression
Adult Ovary	0.7	no expression
Adult Testis	2	no expression
Adult Male Accessory Gland	60.2	low expression
Adult Carcass	773.2	moderate expression

B

Gene FPKMs and Enrichments ? ☑ SDs ☑ Whole Body ☑ Male v. Female

	Adult Male		Adult Female		Male v. Female		Larval	
Tissue	FPKM	Enrich...	FPKM	Enrich...	M/F	p value	FPKM	Enrich...
Head	35 ± 4.4	1.6	30 ± 7.4	6.1	1.15	n.s.		
Eye	57 ± 4.3	2.7	52 ± 2.1	10	1.10	n.s.		
Brain / CNS	6.4 ± 0.8	0.3	6.1 ± 0.6	1.2	1.04	n.s.	3.0 ± 0.6	0.8
Thoracicoabdominal g...	45 ± 8.0	2.1	35 ± 1.7	7.0	1.30	n.s.		
Crop	3.5 ± 2.6	0.2	3.9 ± 0.3	0.8	0.91	n.s.		
Midgut	1.0 ± 0.3	0.0	0.2 ± 0.1	0.0	1.00	p < 0.05	0.2 ± 0.1	0.1
Hindgut	1.9 ± 0.5	0.1	4.8 ± 1.1	1.0	0.42	p < 0.05	0.4 ± 0.1	0.1
Malpighian Tubules	0.8 ± 0.2	0.0	0.9 ± 0.2	0.2	1.00	n.s.	0.4 ± 0.2	0.1
Fat body	36 ± 1.4	1.7	22 ± 2.9	4.5	1.60	p < 0.01	35 ± 11	9.2
Salivary gland	6.3 ± 0.2	0.3	2.0 ± 0.8	0.4	3.1	n.s.	1.3 ± 0.3	0.3
Heart	pending	—	pending	—				
Trachea							0.8 ± 0.3	0.2
Ovary			0.3 ± 0.1	0.1				
Virgin Spermatheca			8.9 ± 2.6	1.8				
Mated Spermatheca			14 ± 2.9	2.8				
Testis	1.4 ± 0.3	0.1						
Accessory glands	3.9 ± 0.4	0.2						
Carcass	42 ± 3.9	2.0	31 ± 8.3	6.3	1.34	n.s.	2.8 ± 0.4	0.7
Rectal pad	14 ± 0.8	0.7	14 ± 0.5	2.8	1.05	n.s.		
Whole body	21 ± 1.4		5.0 ± 1.1		4.3	p < 0.01	3.8 ± 0.6	

Figure S8

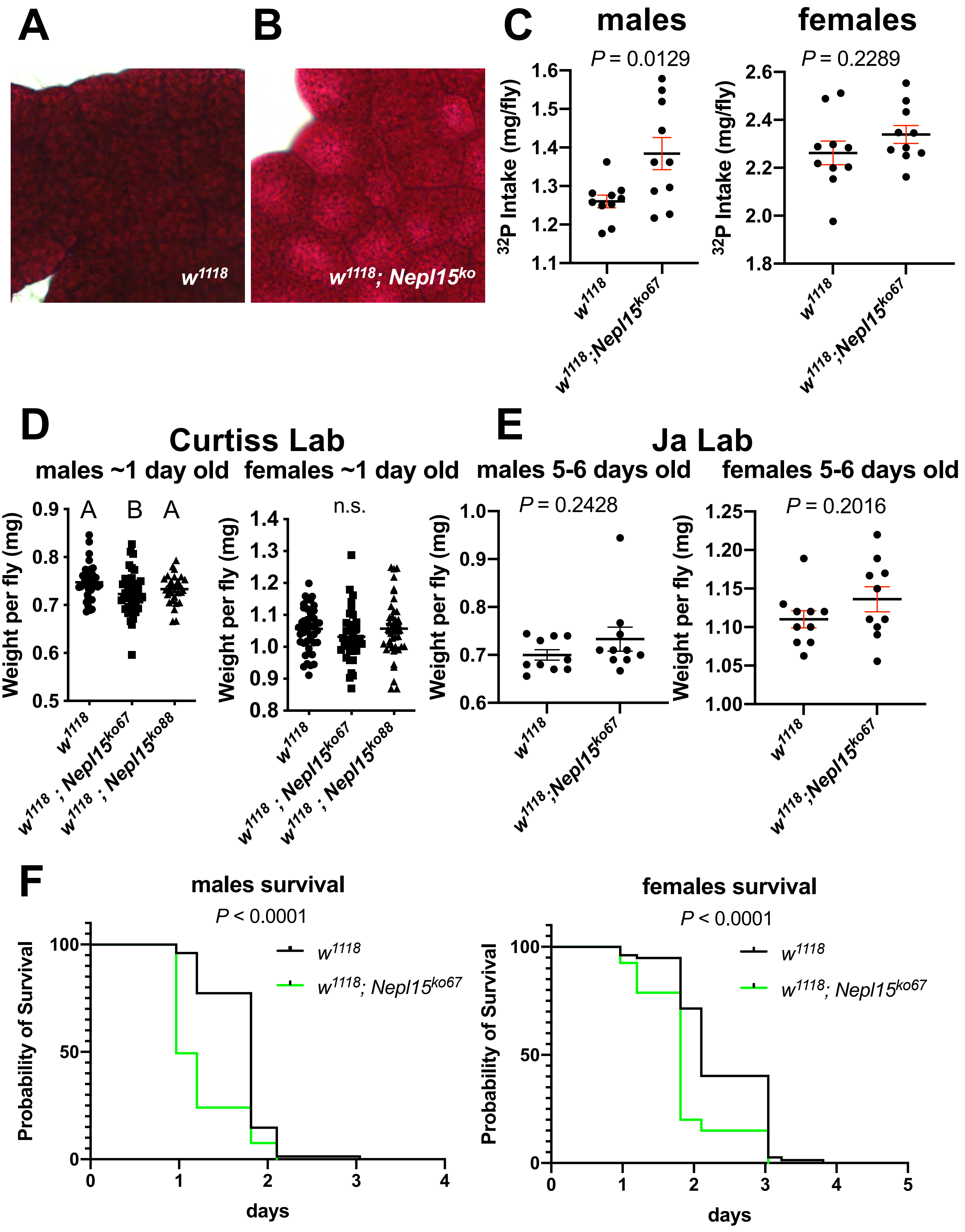

A
B
C
w1118
w1118; Nepl15ko
males
females
P = 0.0129
P = 0.2289
32P Intake (mg/fly)
w1118
w1118;Nepl15ko67
D
Curtiss Lab
males ~1 day old
females ~1 day old
A B A
n.s.
Weight per fly (mg)
w1118 ; Nepl15ko67
w1118 ; Nepl15ko88
E
Ja Lab
males 5-6 days old
females 5-6 days old
P = 0.2428
P = 0.2016
F
males survival
females survival
P < 0.0001
Probability of Survival
w1118; Nepl15ko67
days

Figure S9

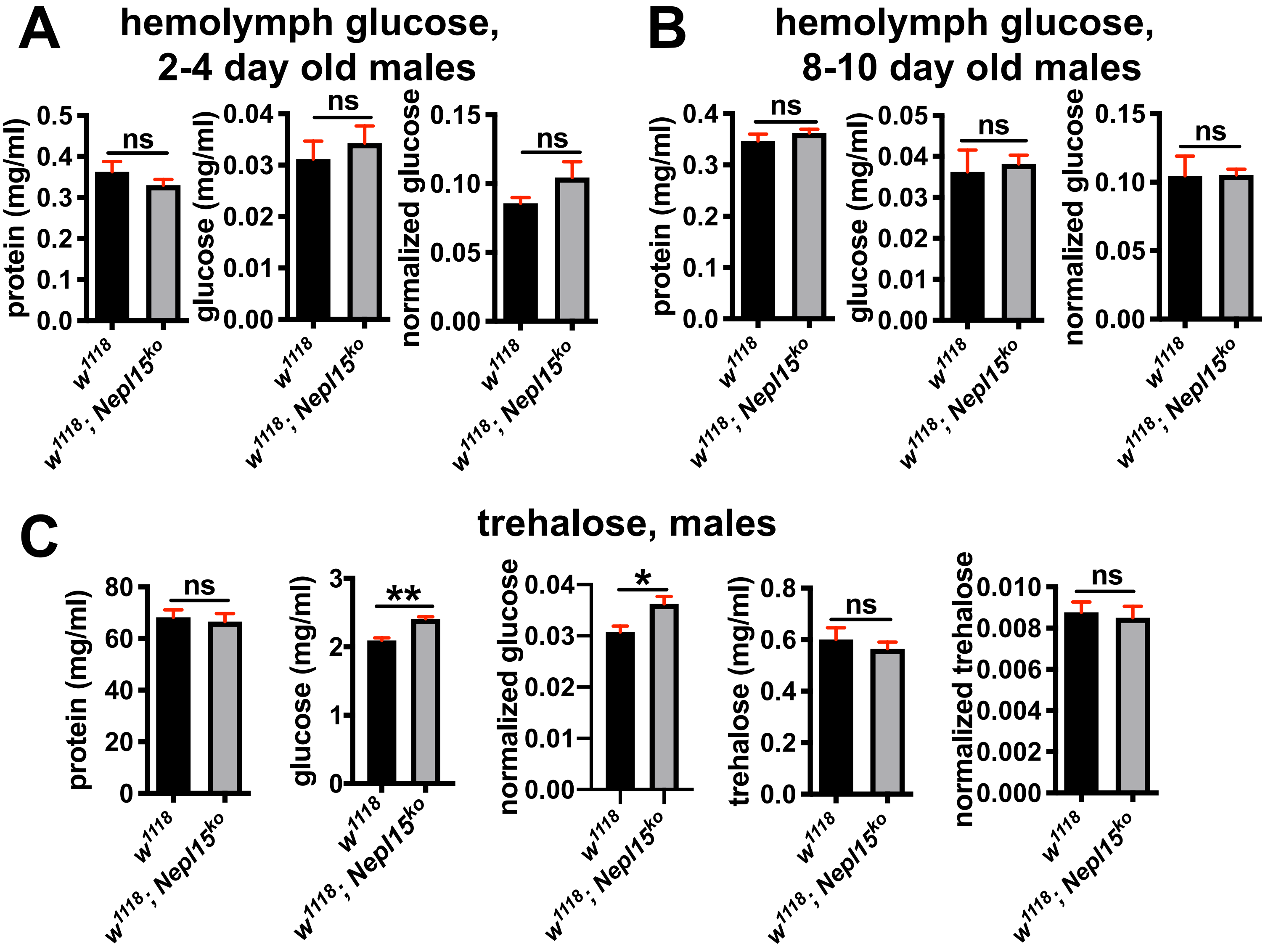
A
hemolymph glucose, 2-4 day old males
B
hemolymph glucose, 8-10 day old males
C
trehalose, males
protein (mg/ml)
glucose (mg/ml)
normalized glucose
trehalose (mg/ml)
normalized trehalose
ns
**
*
w1118
w1118; Nepl15ko

Figure S10

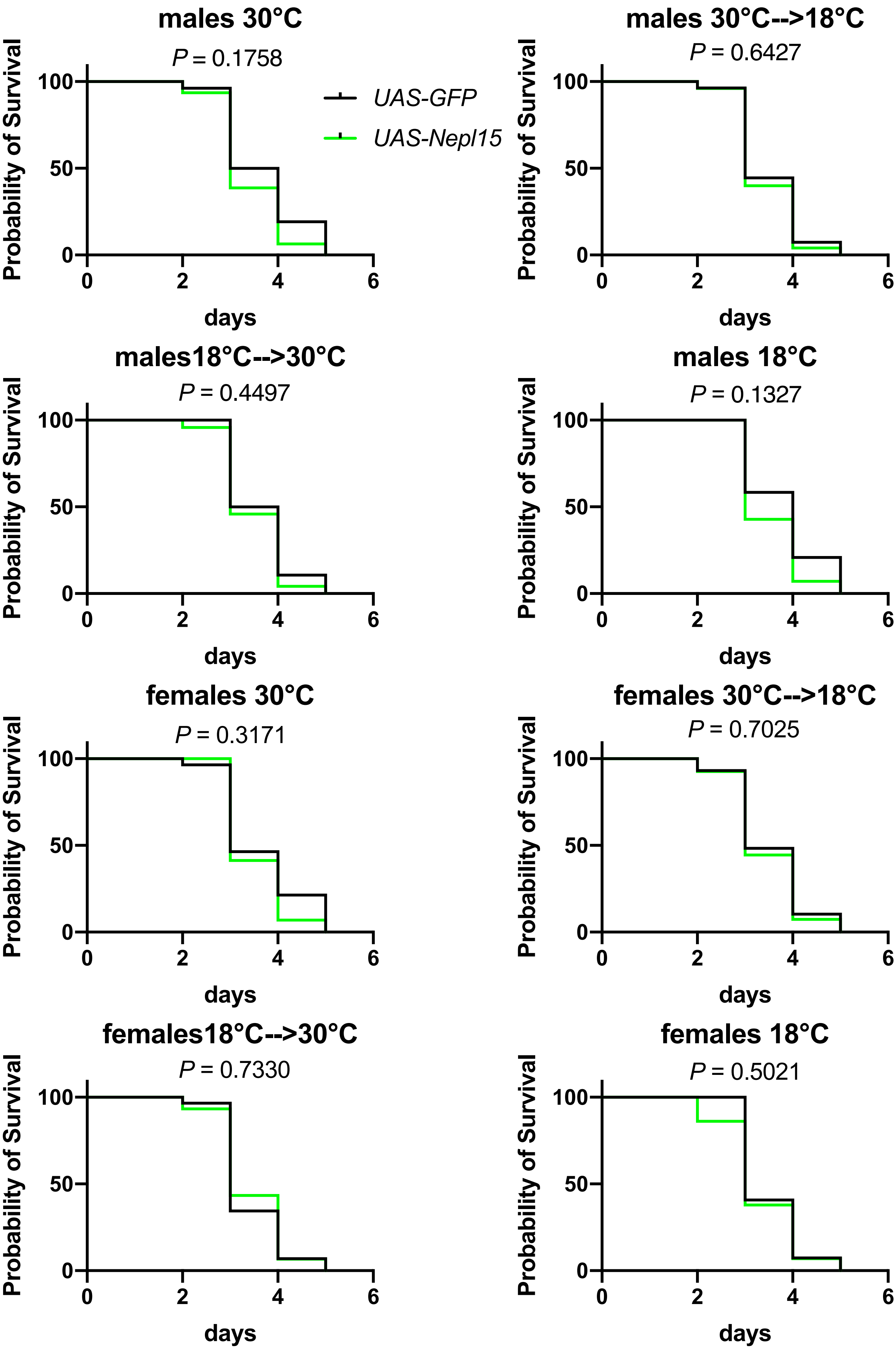
males 30°C
P = 0.1758
UAS-GFP
UAS-Nepl15
males 30°C-->18°C
P = 0.6427
males18°C-->30°C
P = 0.4497
males 18°C
P = 0.1327
females 30°C
P = 0.3171
females 30°C-->18°C
P = 0.7025
females18°C-->30°C
P = 0.7330
females 18°C
P = 0.5021
Probability of Survival
days
0
50
100
0
2
4
6
