# Supplementary material for: The Drosophila melanogaster Neprilysin Nepl15 is involved in lipid and carbohydrate storage
Source: Sci Rep. 2021 Jan 22;11:2099. doi: 10.1038/s41598-021-81165-z (PMC7822871; doi:10.1038/s41598-021-81165-z)
Supplement: Supplementary file 2 — Supplementary Legends. [file 41598_2021_81165_MOESM2_ESM.docx]

**The *Drosophila melanogaster* Neprilysin Nepl15 is involved in lipid and carbohydrate storage**

Surya Banerjee, Christine Woods, Micheal Burnett, Scarlet J. Park, William Ja, Jennifer Curtiss

**Supplemental Figure S1.** Alignments of the predicted *Dm*Nepl15 protein sequence to those of *Hs*Neprilysin and *Hs*ECE1, as well as to the Nepl15 orthologs from the *Drosophila* genomes sequenced by the *Drosophila* 12 Genomes Consortium. Secondary structure elements of domains 1, 2 and 3 of *Hs*Neprilysin, based on a model of HsNeprilysin in complex with the phosphoramidon inhibitor [1], are indicated in magenta, blueberry and turquoise, respectively. Colored boxes indicating an alpha helix are labeled with sequential letters (e.g. alpha helix A of domain 1 is listed as A1, etc.). Open boxes indicating a beta sheet are labeled with roman numerals (e.g. beta sheet I of domain 1 is listed as I1, etc.). Known (*Hs*Neprilysin and *Hs*ECE1) or probable (Nepl15) cysteines involved in di-sulfide bonds are labeled with yellow boxes.

As with other M13 family members [1, 2], the percent identity between *Dm*Nepl15, *Hs*Neprilysin and *Hs*ECE1 is greatest at the C-terminus. For instance, whereas alignment of full-length protein sequences reveals 24%, 24% and 39% identity for *Dm*Nepl15 compared to *Hs*ECE-1, *Dm*Nepl15 compared to *Hs*Neprilysin and *Hs*ECE-1 compared to *Hs*Neprilysin, respectively, alignments of the ~200 most C-terminal amino acids reveals 35%, 33% and 59% identity for *Dm*Nepl15 compared to *Hs*ECE-1, *Dm*Nepl15 compared to *Hs*Neprilysin and *Hs*ECE-1 compared to *Hs*Neprilysin, respectively. This C-terminal region contains most of the amino acids that play key roles in the catalytic site (boxed, see main text for details).

**Supplemental Figure S2.** (A-C) Data from SwissModel for three Nepl15 homology models. To the right in each panel is the alignment used in the homology model with a QMEAN color scheme in which the regions with best alignment are colored blue and the regions with worst alignment are colored red. To the left in each panel are SwissModel’s analytics of model quality. (A) Model 01 is a *Dm*Nepl15 homology model based on a model of a crystal structure of *Hs*Neprilysin in complex with the active metabolite of the drug sacubitril, LBQ657 (5mjy) [3]; GMQE score of 0.62; QMEAN score -3.81 (acceptable). (B) Model 02 is a *Dm*Nepl15 homology model based on a model of a crystal structure of *Hs*Neprilysin in complex with the phosphoramidon inhibitor (1dmt) [1]; GMQE score of 0.63; QMEAN score -3.03 (acceptable). (C) Model 03 is a *Dm*Nepl15 homology model based on a model of a crystal structure of *Hs*ECE-1 in complex with the phosphoramidon inhibitor (3dwb) [4]; GMQE score of 0.62; QMEAN score -3.74 (acceptable).

**Supplemental Figure S3.** Results of the MolProbity analysis of the 3 *Dm*Nepl15 homology models. (A) Model 01 is a *Dm*Nepl15 homology model based on a model of a crystal structure of *Hs*Neprilysin in complex with the active metabolite of the drug sacubitril, LBQ657 (5mjy) [3]. MolProbity Clashscore, all atoms: 1.84 (99^th^ percentile); MolProbity score 1.53 (94^th^ percentile). (B) Model 02 is a *Dm*Nepl15 homology model based on a model of a crystal structure of *Hs*Neprilysin in complex with the phosphoramidon inhibitor (1dmt) [1]; MolProbity Clashscore, all atoms: 9.96 (71^st^ percentile); MolProbity score 2.07 (72^nd^ percentile). (C) Model 03 is a *Dm*Nepl15 homology model based on a model of a crystal structure of *Hs*ECE-1 in complex with the phosphoramidon inhibitor (3dwb) [4]; MolProbity Clashscore, all atoms: 10.3 (70^th^ percentile); MolProbity score 2.24 (62^nd^ percentile).

**Supplemental Figure S4.** *Drosophila* Nepl15 orthologs are predicted to be secreted. Analysis of the *Dm*Nepl15 predicted protein sequences using TargetP 1.1 [5] reveals that for all 12 orthologs the location with the highest score is the Secretory Pathway with a Reliability Class (RC) of 1 (difference between the highest and the second highest output scores > 0.800) or 2 (difference between the highest and the second highest output scores of between 0.800 and 0.600). Len: sequence length; mTP: mitochondrial targeting peptide; SP: signal peptide Loc: Prediction of localization; S: Secretory pathway, i.e. the sequence contains SP, a signal peptide.

**Supplemental Figure S5.** (A-K) Analysis of the predicted *Dm*Nepl15 protein sequence as well as that of the Nepl15 orthologs from the *Drosophila* genomes sequenced by the *Drosophila* 12 Genomes Consortium [6] suggests that all have a signal peptide or a transmembrane helix. (LEFT) Analysis with SignalP 4.1 [7, 8]. All 12 *Drosophila* Nepl15 orthologs received Discrimination (D) scores that are well above the default threshold of 0.450), suggesting that all have a signal peptide. Plots of C-, S- and Y-scores versus the position of the first 70 amino acids in the predicted protein reveal the predicted cleavage sites according to the following. Max. C: the positions with the highest raw cleavage (C) site scores; values are high at the position immediately after a predicted cleavage site. Max. S: the position with the highest signal (S) peptide score; values are high at positions within signal peptides. Max. Y: the Y score is the combined cleavage site score, which is the geometric average of the C-score and the S-score; values are highest for C-score peaks where the slope of the S-score is steepest, allowing for choosing among multiple cleavage site possibilities. Mean S: the average S-score from position 1 to the position immediately before the maximal Y-score. D: the discrimination score, which is a weighted average of the mean S and the Max. Y scores, and is used to discriminate signal peptides from non-signal peptides. (RIGHT) Analysis with TMHMM 2.0 [9, 10]. Length: the length of the protein sequence. Number of predicted TMHs the number of predicted transmembrane helices. Exp number of AAs in TMHs: the expected number of amino acids in transmembrane helices (values >18 indicate a probable transmembrane protein or a signal peptide). Exp number, first 60 AAs: the expected number of amino acids in transmembrane helices in the first 60 amino acids in the protein; values greater than 10 indicate that the predicted N-terminal transmembrane helix could be a signal peptide. Total prob of N-in: the total probability that the protein N-terminus is on the cytoplasmic side of the membrane. TMHMM2.0: prediction of which amino acids are inside (i.e. in the cytoplasm), in a TMhelix (transmembrane helix) or outside (i.e. extracellular). Plots show the probability for a particular amino acid in the predicted protein sequence to be to be inside, in a transmembrane helix, or outside. The line at the top of each plot (between 1 and 1.2) is a plot of the predicted location of each amino acid in the predicted sequence. (NOT SHOWN) TMHMM 2.0 analysis of the *Drosophila* Nepl15 orthologs’ predicted protein sequence minus the predicted signal peptide/transmembrane helix indicates that there are no transmembrane helices C-terminal to the signal peptide/transmembrane helix in any of the predicted protein sequences.

**Supplemental Figure S6.** All of the *Drosophila* Nepl15 orthologs are predicted to be extracellular, soluble proteins, except for the *D. ananassae* ortholog, which is predicted to be a soluble endoplasmic reticulum protein. (A-L) DeepLoc-1.0 [11] was used to generate hierarchical trees of sorting pathways resulting in prediction of subcellular localization.

**Supplemental Figure S7.** (A) FlyAtlas Anatomical Expression Data (microarray data) [12] downloaded from FlyBase version FB2019_04. (B) FlyAtlas 2 [13] RNAseq data downloaded from the FlyAtlas 2 website (<http://flyatlas.gla.ac.uk/FlyAtlas2/index.html>) on September 14, 2019.

**Supplemental Figure S8.** (A, B) Brightfield images of fat bodies from larvae of the indicated genotypes. Darker staining indicates more polysaccharides. (C) Food intake measured by food labeling by radioactive tracer. (n = 10 for both males and females of both genotypes) (D,E) Fly weights for males and females of the indicated ages and genotypes determined in the Curtiss lab (D) and in the Ja lab (E). For panel D, males: n = 40 for *w^1118^*, n = 44 for *w^1118^; Nepl15^67^*, n = 28 for *w^1118^; Nepl15^88^*. For panel D, females: n = 44 for *w^1118^*, n = 38 for *w^1118^; Nepl15^67^*, n = 41 for *w^1118^; Nepl15^88^*. For panel E n = 10 for both males and females of both genotypes. Values in C and E were analyzed using an unpaired t test; *P* values are indicated on the graphs. Values in D were analyzed using a one-way ANOVA and were assigned to statistical groups using Tukey’s multiple comparison test. Groups sharing at least one letter are not significantly different; groups not sharing any letter are significantly different (*P* < 0.05). Error bars in C-E) represent the standard error of mean (SEM). (F) Survival curves for starvation assays for males (left) and females (right) of the indicated genotypes. (n = 74 for *w^1118^* males, n = 79 for *w^1118^; Nepl15^67^* males, n = 77 for *w^1118^* females, n = 80 for *w^1118^; Nepl15^67^* females. Values were analyzed using a Log-rank test, and the *P* values are indicated on the graphs.

**Supplemental Figure S9.** Stored carbohydrates, but not circulating carbohydrates are affected by *Nepl15^ko^*. (A,B) Bright-field images of Periodic Acid Schiff stained fat bodies from wild-type and *Nepl15^ko^* larvae. (C,D) Hemolymph protein concentration (left), hemolymph glucose concentration (center) and glucose concentration normalized to protein concentration (right) for adult 2-4 day old males (C) and adult 8-10 day old males (D) of the indicated genotypes. (E) Whole body protein concentration (left), glucose concentration (center left), glucose concentration normalized to protein concentration (center), trehalose concentration (center right). And trehalose concentration normalized to protein concentration (right) for adult males of the indicated genotypes. Values were analyzed using an unpaired t test. *, ** and *** indicate P < 0.05, P < 0.01 and P < 0.001, respectively. ns = non-significant. Error bars represent the standard error of mean (SEM). n = 3 biological replicates with 3 technical replicates each for all panels.

**Supplemental Figure S10.** Survival curves for starvation assays for males (left) and females (right) of the indicated genotypes and temperature regimens used in the TARGET experiments. Values were analyzed using a Log-rank test, and the *P* values are indicated on the graphs. (30$^{\circ}$C males: n = 26 for *UAS-GFP*, n = 31 for *UAS-Nepl15*) (30$^{\circ}$C 🡪 18$^{\circ}$C males: n = 27 for *UAS-GFP*, n = 25 for *UAS-Nepl15*) (18$^{\circ}$C 🡪 30$^{\circ}$C males: n = 28 for *UAS-GFP*, n = 24 for *UAS-Nepl15*) (18$^{\circ}$C males: n = 24 for *UAS-GFP*, n = 28 for *UAS-Nepl15*) (30$^{\circ}$C females: n = 28 for *UAS-GFP*, n = 29 for *UAS-Nepl15*) (30$^{\circ}$C 🡪 18$^{\circ}$C females: n = 29 for *UAS-GFP*, n = 27 for *UAS-Nepl15*) (18$^{\circ}$C 🡪 30$^{\circ}$C females: n = 29 for *UAS-GFP*, n = 39 for *UAS-Nepl15*) (18$^{\circ}$C females: n = 27 for *UAS-GFP*, n = 29 for *UAS-Nepl15*)

**REFERENCES**

1. Oefner, C., et al., *Structure of human neutral endopeptidase (Neprilysin) complexed with phosphoramidon.* J Mol Biol, 2000. **296**(2): p. 341-9.

2. Tiraboschi, G., et al., *A three-dimensional construction of the active site (region 507-749) of human neutral endopeptidase (EC.3.4.24.11).* Protein Eng, 1999. **12**(2): p. 141-9.

3. Schiering, N., et al., *Structure of neprilysin in complex with the active metabolite of sacubitril.* Sci Rep, 2016. **6**: p. 27909.

4. Schulz, H., et al., *Structure of human endothelin-converting enzyme I complexed with phosphoramidon.* J Mol Biol, 2009. **385**(1): p. 178-87.

5. Emanuelsson, O., et al., *Predicting subcellular localization of proteins based on their N-terminal amino acid sequence.* J Mol Biol, 2000. **300**(4): p. 1005-16.

6. Clark, A.G., et al., *Evolution of genes and genomes on the Drosophila phylogeny.* Nature, 2007. **450**(7167): p. 203-18.

7. Petersen, T.N., et al., *SignalP 4.0: discriminating signal peptides from transmembrane regions.* Nat Methods, 2011. **8**(10): p. 785-6.

8. Nielsen, H., *Predicting Secretory Proteins with SignalP.* Methods Mol Biol, 2017. **1611**: p. 59-73.

9. Sonnhammer, E.L., G. von Heijne, and A. Krogh, *A hidden Markov model for predicting transmembrane helices in protein sequences.* Proc Int Conf Intell Syst Mol Biol, 1998. **6**: p. 175-82.

10. Krogh, A., et al., *Predicting transmembrane protein topology with a hidden Markov model: application to complete genomes.* J Mol Biol, 2001. **305**(3): p. 567-80.

11. Almagro Armenteros, J.J., et al., *DeepLoc: prediction of protein subcellular localization using deep learning.* Bioinformatics, 2017. **33**(21): p. 3387-3395.

12. Chintapalli, V.R., J. Wang, and J.A. Dow, *Using FlyAtlas to identify better Drosophila melanogaster models of human disease.* Nat Genet, 2007. **39**(6): p. 715-20.

13. Leader, D.P., et al., *FlyAtlas 2: a new version of the Drosophila melanogaster expression atlas with RNA-Seq, miRNA-Seq and sex-specific data.* Nucleic Acids Res, 2018. **46**(D1): p. D809-D815.
